# The Worldwide Variation in Avian Clutch Size across Species and Space

**DOI:** 10.1371/journal.pbio.0060303

**Published:** 2008-12-09

**Authors:** Walter Jetz, Cagan H Sekercioglu, Katrin Böhning-Gaese

**Affiliations:** 1 Division of Biological Sciences, University of California San Diego, La Jolla, California, United States of America; 2 Center for Conservation Biology, Department of Biology, Stanford University, Stanford, California, United States of America; 3 Institut für Zoologie, Abteilung Ökologie, Johannes Gutenberg-Universität, Mainz Germany; University of Oxford, United Kingdom

## Abstract

Traits such as clutch size vary markedly across species and environmental gradients but have usually been investigated from either a comparative or a geographic perspective, respectively. We analyzed the global variation in clutch size across 5,290 bird species, excluding brood parasites and pelagic species. We integrated intrinsic (morphological, behavioural), extrinsic (environmental), and phylogenetic effects in a combined model that predicts up to 68% of the interspecific variation in clutch size. We then applied the same species-level model to predict mean clutch size across 2,521 assemblages worldwide and found that it explains the observed eco-geographic pattern very well. Clutches are consistently largest in cavity nesters and in species occupying seasonal environments, highlighting the importance of offspring and adult mortality that is jointly expressed in intrinsic and extrinsic correlates. The findings offer a conceptual bridge between macroecology and comparative biology and provide a global and integrative understanding of the eco-geographic and cross-species variation in a core life-history trait.

## Author Summary

Why do some bird species lay only one egg in their nest, and others ten? The clutch size of birds is one of the best-studied life-history traits of animals. Nevertheless, research has so far focused either on a comparative approach, relating clutch size to other biological traits of the species, such as body weight; or on a macroecological approach, testing how environmental factors, such as seasonality, influence clutch size. We used the most comprehensive dataset on clutch size ever compiled, including 5,290 species, and combined it with data on the biology and the environment of these species. This approach enabled us to merge comparative and macroecological methods and to test biological and environmental factors together in one analysis. With this approach, we are able to explain a major proportion of the global variation in clutch size and also to predict with high confidence the average clutch size of a bird assemblage on earth. For example, cavity nesters, such as woodpeckers, have larger clutches than open-nesting species; and species in seasonal environments, especially at northern latitudes, have larger clutches than tropical birds. The findings offer a bridge between macroecology and comparative biology, and provide a global and integrative understanding of a core life-history trait.

## Abstract 3

Why do some bird species lay only one egg in their nest, and others ten? An analysis combining comparative and macroecological approaches across more than half of all bird species explains the global variation in this trait with high confidence.

## Introduction

There is enormous variation in life-history among species and across regions, which ecologists have long sought to explain [1–3]. One trait of particular interest is the number of eggs laid per nest (clutch size) by birds, which is central to avian reproductive effort and probably the best-recorded animal life-history trait. The causes of its substantial variation have fascinated behavioural, ecological, and evolutionary biologists for more than 60 years [2,3], but remain highly debated [4–6]. Life-history theory aims to discover the factors that determine intra- and inter-specific variation in life-history traits. This discipline has recently benefited from observational and experimental studies that have quantified important trade-offs, reaction norms, and phenotypic plasticity of the variation within populations and species [7,8]. However, this work is limited in its ability to explain the tremendous interspecific and geographic variation in life-histories—from warblers to raptors, and from the tropics to the poles. Inspired by David Lack's original observations [3], comparative biologists have documented that clutch size tends to be conserved within clades and often co-varies with intrinsic (biological) attributes such as body size, nestling development, and nest type. At the same time, others have pointed to extrinsic (environmental) influences on clutch size with respect to latitude [1,2], gradients of resource availability, and seasonality [4,9,10], and between biogeographic regions. Lack [3,11] hypothesised that clutch size may be determined by food abundance during the breeding period, per se, and that northern species have large clutches because daylight periods during the breeding season are longer than those in the tropics. In contrast, seasonality of food abundance is suggested to be linked to clutch size by two alternative mechanisms. Classical life-history theory predicts that high seasonality in the temperate regions, causing high adult mortality, will lead to the evolution of high investment in current reproduction and large clutch sizes because the likelihood to survive until the next breeding season is low [5]. Alternatively, Ashmole [9] argued that high adult mortality in the temperate regions reduces population density, increases per-individual resource availability in the breeding season, and allows temperate birds to nourish large clutches [4,12].

Critically, like these suggested extrinsic drivers, clades and their intrinsic traits that may affect clutch size are also not randomly distributed along environmental gradients or realms. Consequently, separate viewpoints focusing on just intrinsic or extrinsic drivers have limited the unification and generalization of our understanding of life-history variation. Here we present an analysis that integrates these perspectives and we assess the variation in clutch size across species and assemblages worldwide. We compiled information on clutch size and other intrinsic (body mass, migratory behavior, development mode, nest type, diet) and extrinsic attributes (latitude, temperature, precipitation, net primary productivity, seasonality, and realm) for a total of 5,290 species of landbirds. This allows us to develop and test a first global model of clutch size that integrates existing viewpoints of life-history variation.

## Results/Discussion

**Figure 1:**
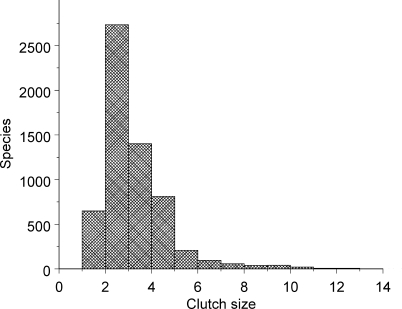
Global Variation in Species Clutch Size Shown are mean clutch sizes of all 5,290 species of landbirds in the analysis (six species with clutch sizes > 14 not illustrated).

While clutch sizes vary over a large range, more than half of all birds lay 2 or 3 eggs (mode: 2, median: 2.8; Figure 1). The right-skewed frequency distribution indicates that from a global perspective, the large clutch sizes of northern temperate bird species—few in numbers, but most frequently studied in life-history research [5,13,14]—are in fact unusual. This highlights the importance for a perspective that extends to the tropics [15]. We find that a key intrinsic factor that distinguishes clades in their typical clutch size is the mode of development [16–19]. Precocial species, with their more mobile offspring, have much larger clutches (*x̄* = 4.49, *N* = 864 species) than altricial species (*x̄* = 2.85, *N* = 4,426; *t* = 21.73, *p* < 0.001). This may be the result of the shorter and less intensive parental care required by precocial young, thus reducing the fitness costs of additional offspring and allowing parents to raise larger clutches [18,19]. Development mode is phylogenetically highly conserved (in our dataset there is no altricial versus precocial variation below the family level), and we note that its consequences and associated selection pressures likely influence other intrinsic effects on clutch size.

**Figure 2:**
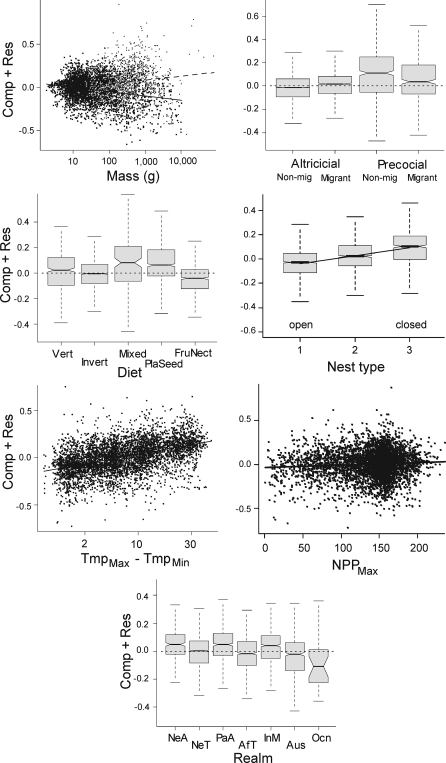
Partial Residual Plots for the Combined Intrinsic-Extrinsic Model of Clutch Size This plot (also called component + residual plot, response: Comp+Res) illustrates the relationship between a predictor and the response given other predictors in the model (specifically, it is a plot of *r_i_* + b*x_i_* versus *x_i_*_,_ where *r_i_* is the ordinary residual for the *i*-th observation, *x_i_* is the *i*-th observation and b is the regression coefficient estimate). For Mass, the solid line and solid symbols refer to altricial species, the dashed line and open symbols to precocial species. To visualize the interaction with Precocial, we excluded that variable for the two interacting predictors (Mass, Migrant) in the calculation of *r_i_*. Abbreviations in Diet: Vert, vertebrates; Invert, invertebrates; Mixed, omnivore; PlaSee, plants and seeds; FruNect, fruits and nectar. In Realm: NeA, Nearctic; NeT, Neotropics; PaA, Palearctic; AfT, Afrotropics; InM, IndoMalaya; Aus, Australasia; Ocn, Oceania. In Migrant: Non-mig, non-migrant. For further details, see Table 1 (cross-species model Both).

Because of the prevalent influence of development mode and its high collinearity with other potential predictors of clutch size (Table S1), we assessed all intrinsic effects in combination (for single-predictor results, see Table S2). A previously noted negative effect of body mass on clutch size [20] is only weakly borne out across the global avifauna for altricial species (Table 1 and Figure 2). From a global perspective, altricial migrants have larger clutches than nonmigrants, especially when extrinsic effects are not accounted for (see below), which is different from studies that do not include tropical species [17,21,22]. Another strong intrinsic determinant of clutch size is nest type [23–25]. Cavity nesters, which are naturally exposed to lower rates of nest predation, tend to have larger clutch sizes than open nesters, and species with half-open nests are in between (Figure 2). Finally, clutch size also varies by diet [3], with granivores and omnivores laying larger clutches than frugivores and nectarivores.

**Table 1:**
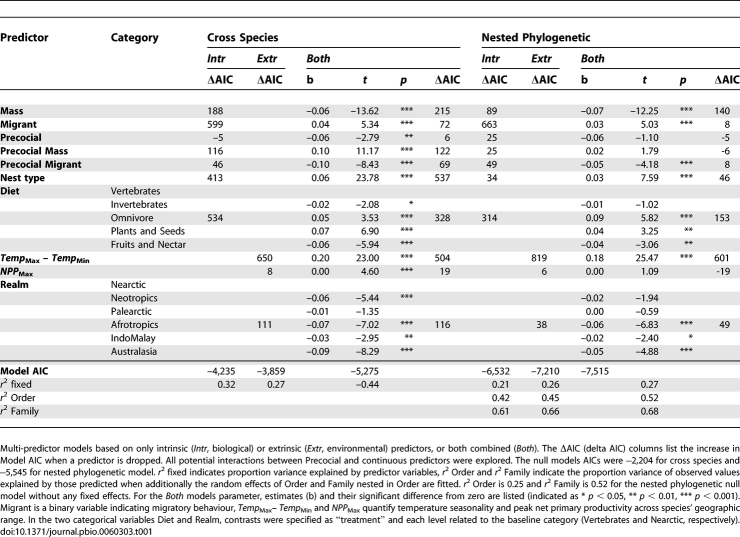
Integrated Models of Clutch Size across 5,290 Bird Species

A second suite of constraints on life histories arises from extrinsic factors characterizing the environment of species. One popular “catch-all” surrogate is latitude, which captures much of the global environmental variation because energy availability becomes more seasonal and is usually reduced at higher latitudes. An increase in clutch size toward the poles has long been noted [1,2] and is confirmed by our data (Table S1). The extensive geographical coverage of our data allows us to disentangle the various environmental trends underlying latitude. We use environmental information integrated across the global breeding distribution of each species to quantify the average extrinsic conditions characterizing its broad-scale niche. Specifically, we evaluate the seasonal difference between summer and winter temperatures (*Temp*_Max_ – *Temp*_Min_, averaged over 3-mo periods), which emerges as the strongest extrinsic predictor: clutch sizes are smallest in species inhabiting relatively aseasonal environments and increase linearly with temperature seasonality (Figure 2). When this seasonality is controlled for, energy availability in the breeding season (*NPP*_Max_) has a very weak positive effect on clutch size. This supports the idea that seasonality in resource conditions has a much stronger effect on clutch size than the absolute level of resources in the breeding season [4,9,10,26]. Even though fine-scale variation in productivity may limit *NPP*_Max_ as estimate of per-individual energy availability during the breeding season, the consistently weak trend over a wide range of environments offers little support for Lack's original hypothesis [3,11]. Finally, after accounting for these two environmental variables, only a limited biogeographic signal (variable *Realm*) remains: birds of Australasia, the Afrotropics, and especially Oceania tend to have smaller clutch sizes than birds of other regions. While the strong deviation in Oceania may arise from a potential over-sampling of species with large clutches (data were available for only 17% of species, compared to 57% elsewhere), the life-history strategies of island taxa may be partially shaped by higher population densities and elevated intraspecific competition [27,28].

**Figure 3:**
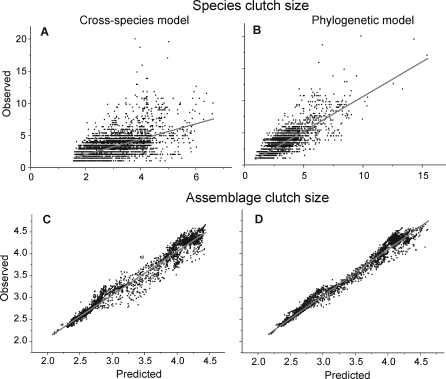
Cross-Species and Cross-Assemblage Model Fits Fit between the observed clutch size and that predicted by the combined intrinsic/extrinsic multi-predictor model *(*Both, Table 1) across 5,290 species (A and B) and 2,521 grid cell assemblages of 220 × 220-km size [(C and D) for geometric mean of assemblage clutch size]. Fits were evaluated for the cross-species model (A and C) and the nested phylogenetic model (B and D). Least squares fits are shown. For details, see text and Table 1. The spatial patterns of the assemblage predictions are illustrated in Figure 4.

Intrinsic and extrinsic life-history determinants do not act in isolation, and our analytical approach allows us to assess their respective contribution in combination. In the joint model, all five intrinsic and three extrinsic predictors so far discussed emerged as significant (Table 1 and Figure 2). All extrinsic and most of the intrinsic variables continue to have very strong effects. This is not true for migratory tendency and diet, which are closely tied to climatic conditions (Table S1). After accounting for temperature seasonality in the combined model, these variables retain relatively little residual effect. This suggests that the larger clutches of altricial migrants arise at least in part from their occupying high-latitude, seasonal environments. By themselves, the pure intrinsic and extrinsic models account for a substantial amount of cross-species variation in clutch size (32% and 27%, respectively). The combined model explains 44% and predicts the absolute variation well (Figure 3A; *x*_observed_ = −0.28 + 1.14 (s.e. = 0.018)*x*_predicted_; *F*_1,5288_ = 3,914).

**Figure 4:**
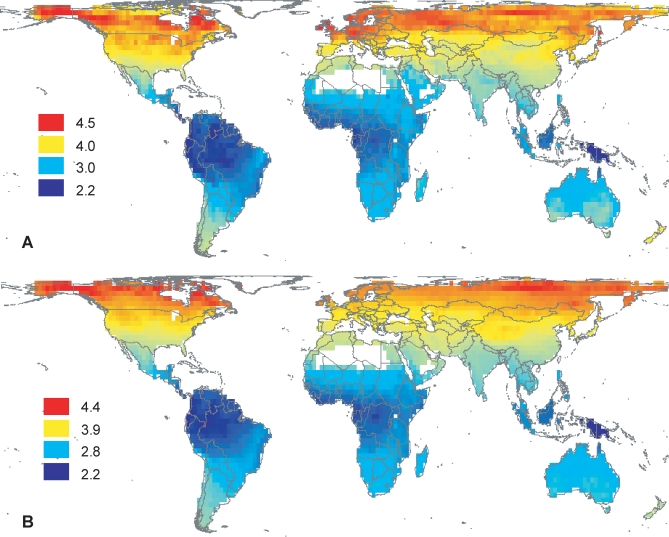
Geographic Variation in Assemblage Clutch Sizes, Observed and Predicted Shown are observed (A) and predicted (B) geographic patterns of the geometric mean clutch size across 5,290 bird species in 2,521 grid cell assemblages of 220 × 220-m size for the cross-species model. Only assemblages with >30 species illustrated. For other details, see Figure 3.

Different lineages may evolve fundamentally different morphological, physiological, and ecological niches and as a consequence exhibit conservatism in both life-history traits and their intrinsic and extrinsic correlates [29]. Over 90% of the variation in key life-history traits in birds occurs at the level of families and higher [30]. We therefore tested the ability of our combined model to predict variability of clutch size in a phylogenetic nested model that takes into account the order and family membership of the species. The results confirm the strong phylogenetic constraints on many intrinsic predictors of clutch size. After accounting for clade membership, the relative strength of the intrinsic compared to the extrinsic model in the cross-species analysis is reversed. Under phylogenetic control, the extrinsic portion of the model offers stronger predictions (*r* ^2^ = 0.26) than the more phylogenetically conserved intrinsic part (*r* ^2^ = 0.21), and the latter does not improve overall model fit as much (delta Akaike information criterion (AIC) of 305 and 983 to full model, respectively). Extrinsic predictors appear orthogonal to phylogeny and the outstanding importance of temperature seasonality is confirmed. As expected, biogeographic realm membership, which is tightly linked to clade-specific biogeographic history, loses importance in the phylogenetic model. Accounting for the phylogenetic variation at the order and family level explains substantial additional variation, increasing *r* ^2^ to 0.68 (Figure 3B).

The geographic context of our data allows us to test the ability of a comparative analysis to predict a global eco-geographic pattern [31]. Specifically, we evaluate how well predictions for each species from our combined cross-species and phylogenetic model fit the observed average (geometric mean) clutch size in 2,521 bird assemblages of 220 × 220-km size. This provides a test of whether the proposed integration of intrinsic and extrinsic factors is able to recreate observed geographic gradients. Observed average clutch sizes across assemblages show a remarkably strong geographic gradient from an average of 4.5 eggs at the high northern latitudes to just over two eggs in the tropics (Figure 4A). We find that our combined cross-species model successfully predicts this geographic pattern (Figure 3C), with a slope almost indistinguishable from 1 (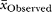 = 0.092 + 0.99 (s.e. = 0.004) 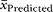 ; *r* ^2^ = 0.97, *F=* 75,680). The phylogenetic nested model provides an even better fit (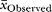 = −0.09 + 1.05 (s.e. = 0.003) 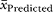 ; *r* ^2^ = 0.98, *F* = 129,800). This exceptional match is confirmed by a visual inspection of the geographic patterns (Figure 4B). It illustrates the statistical strength that assemblage attributes, averaged across species, can achieve even when underlying detectable trends across species are weaker. Geographic trends in the attributes of assemblages (e.g., mean assemblage clutch size) are affected by species' different geographic range sizes, as wide-ranging species occur in a disproportionate number of assemblages and thereby dominate geographic patterns [32]. Geographic trait patterns based on assemblage averages therefore carry a signal of both trait and range size variation across space. Consequently, models of eco-geographic patterns confound correlates of trait variation with correlates of species distributions and range size (and their respective patterns of spatial autocorrelation). For an understanding of potential extrinsic determinants of trait variation, we therefore advocate the use of a comparative approach for biological inference. Additionally testing whether a model can predict geographic patterns allows validation and bridges to the eco-geographic perspective.

Our findings on 56% of the world's landbirds empirically support recent theoretical work that highlighted the importance of food seasonality via adult mortality on clutch size [26,33]. Highly seasonal environments can cause increased adult mortality [34], e.g., because birds have to survive low temperatures and resource conditions in situ or because they have to migrate, which carries risks and costs. Additional effects on population density and, indirectly, per-individual resource availability in the breeding season, then combine to make seasonality of resources the predominant driver of clutch size variation across geographic gradients [26,33]. The significance of mortality, in this case mostly of offspring, for the evolution of clutch size is also expressed in the most important intrinsic determinant: nest type. Closed-nesters are subject to much smaller rates of nest predation or loss [23,24], and nest safety may influence clutch size through clutch size–dependent nest predation [35] or the effect of chick survival on adult density [4,9]. Nest type is phylogenetically conserved (its importance decreases strongly when phylogeny is addressed, Table 1), and it is clearly an intrinsic attribute. But its importance may itself be modulated by extrinsic constraints connected to mortality, such as nest predation pressure. This illustrates yet further the strong link between environmental conditions and the evolution and geographic distribution of biological traits such as nest type, which in turn affect life-history traits. Intricate disruptions of such trait associations may arise from climate change and its differential consequences for extrinsic vs. intrinsic determinants.

This study confirms many of the previously asserted correlates of clutch size, but moreover demonstrates how life-history traits are jointly determined by the interplay of intrinsic biological traits, the phylogenetic affinities, and the environment of a species. Understanding these interactions is vital for gauging broad-scale life-history consequences of future climate change and their potential impacts on biodiversity. Our findings call for a combination of traditional cross-species comparative analyses with spatial and macroecological approaches to gain a more integrative, conceptual understanding of life-history variation. Using this approach offers a compelling integration of the intrinsic and extrinsic determinants of trait variation that help understand long-noted eco-geographic patterns and critical linkages in a world of change.

## Materials and Methods

### Data.

We obtained the minimum and maximum clutch size data for 5,290 landbird species from a range of literature sources (see Tables S4–S6 and [36] for detailed overview). In this compilation, we did not include brood parasites, as their clutch size is difficult to define (female birds usually spread a large number of eggs over many host nests) and is obviously exposed to very different selection pressures. We also excluded predominately pelagic and marine species, because the environmental data in the analysis (see below) prevent a straightforward comparison with predominantly terrestrial species. We calculated the species-typical clutch size as the geometric mean of the typical minimum and maximum clutch size (for an evaluation of intraspecific variation, see below). For the same species, we compiled data on species-typical values of potential intrinsic determinants (development mode, body mass, migratory behavior, nest type, diet) from the literature (see Tables S4–S6 for details). We classified species into precocial (newly born young are relatively mobile, covered in feathers, and independent) and altricial (newly born young are relatively immobile, naked, and usually require care and feeding by the parents). Mass information (body mass in grams) was compiled from a variety of sources and averaged across up to four sources, and, if they differed, across sexes. Diet data came from the dataset described in [36]. Species dietary preferences were first recorded across nine major diet categories, and species were subsequently assigned to one out of five primary diets (vertebrates, invertebrates, fruits or nectar, other plant material or seeds, and omnivore). We were able to compile data on nest type data for 2,816 species in the analysis and for all 1,293 genera. Based on these data, we scored nest type according to levels of nest cover as follows: 1, open (e.g., no nest, cup, scrape, saucer, platform); 2, half-open nest (e.g., pendant, sphere, dome, pouch, crevice); 3, closed (cavity, burrow). This nest cover score only showed minor variation within genera, and across the 2,816 species with data the average genus score was an adequate surrogate for the species-level score (Nest type(genus) = −0.00 + 1.00 × nest type(species). *r* ^2^ = 0.90, *F*_1,2814_ = 26,740). We therefore used genus-typical nest type scores for all species lacking data. We acknowledge that this may inflate the type I error for this variable in our nonphylogenetic analysis. We assessed the migratory tendency of species (Migrant) and classified them into non-migrants (non-migrants, only altitudinal and local migrants) and migrants (inter- and intra-continental) following [37] and [38].

We used the extent of occurrence maps of breeding ranges derived from a variety of literature sources (for details see Figure S1, Table S4, and [39]) (for justification of grain size see [40]) to characterize the broad-scale environmental attributes of species. We calculated the centroid of the range in shapefile format to derive the average absolute latitude of a species' range location (Abs. Latitude). We extracted range map occurrences across a 55 × 55 km^2^ equal area grid (cylindrical equal area projection), which we then linked to an environmental dataset extracted across the same grid at 0.01° spatial resolution. Temperature (°C) and precipitation (mm) data came from University of East Anglia's Climatic Research Unit gridded climatology 1961–1990 dataset [41] at native 10-min resolution. We determined average annual temperature (*Temp*_Avg_), and total annual precipitation (*Prec*_Total_). For seasonality of temperature, we used average temperature of the coldest and warmest three months across all years (*Temp*_Min_, *Temp*_Max_) and calculated the annual temperature range (*Temp*_Max_ *– Temp*_Min_). In order to achieve a representative estimate of both total and seasonal net primary productivity across the second half of the 20th century, we used the output for above-ground NPP (g Carbon m^−2^) from a recently developed global productivity model [42] based on the Lund-Potsdam-Jena dynamic global vegetation model, including land-use [43]. We averaged model output across 1961–1990 and in the analysis used total annual NPP (*NPP*_Total_), NPP of the most productive three months (*NPP*_Max_), and a ratio characterizing seasonality in NPP (1 *–* (*NPP*_Min_ */NPP*_Max_)). We use NPP as a broad-scale, general proxy for food abundance [44], with *NPP*_Total_ reflecting average food abundance during the year, *NPP*_Max_ being food abundance in the breeding season, and (1 – (*NPP*_Min_/*NPP*_Max_)) mirroring seasonal variation in food abundance. Both tropical and temperate birds have been shown to most likely breed in the months with highest NPP [26]. For biogeographic realm, we determined the realm that contains the majority of a species breeding range. We followed the regionalization originally given by [45] as spatially implemented by the World Wildlife Fund (WWF) [46], excluding the Antarctic region.

Our taxonomy and phylogenetic placement of families and orders follows [37], with several updates (see [39] and Tables S4–S6 for details). Following [37], we excluded from the analysis brood parasites and species that forage predominately in pelagic and marine waters during the breeding season, resulting in a global list of 9,391 bird species. Data were not available for all predictor variables for some species, which therefore had to be excluded. The restricted final dataset with full information consists of 5,290 species, i.e., 56% of all qualifying species worldwide (for a list of species used in the analysis see Table S4). Global representation of qualifying species across realms ranged from high in Nearctic (95%) and Palearctic (80%), to medium in Indomalaya (57%) and Aftrotropics (71%), to low in Australasia (49%) and Neotropics (45%), and poor in Oceania (16%).

### Analysis.

We log_10_-transformed mean clutch size. Visual inspection indicated that this sufficiently stabilized model residuals. We transformed *Mass* and *Prec*_Total_ as log_10_(*x*), *Temp*_Avg_ as log_10_(*x* + 100), and *Temp*_Max_ – *Temp*_Min_ as log_10_(*x* + 1). Predictor variables were mostly weakly correlated, although 8 out of 65 variable combinations reached Spearman rank correlations ≥ 0.75 (Table S1). We first ran single-predictor cross-species linear models to test the influence of each predictor variables on log-transformed clutch size (Table S2), and we then used AIC values to guide variable selection for multi-predictor models. Given the different selection pressures on clutch size between precocial and altricial birds, we analyzed single predictor effects separately for these two groups and examined interactions between the factorial variable precocial and all other continuous predictors. We built the multi-predictor model starting with the variable that had the lowest AIC value in the single predictor models and sequentially added the next-strongest predictor.

We first developed multi-predictor models of the selected intrinsic and extrinsic variables separately by sequentially adding the best-performing variables, and then combined both sets of predictors in a joint model (the “Both” model, Table 1). We only included variables in the final model if the improvement in AIC they provided to the combined model was over ten. In a second set of analyses, we built nested phylogenetic models to address the strong phylogenetic signal in clutch size variation. Specifically, we fitted a linear mixed effects model using the function lme in the nlme library version 3.1–83 run in R 2.5.1. We used the taxonomic ranks Order and Family in [37] to assign nested clade membership and fitted Order alone and Family nested in Order as random effects in addition to the fixed effects selected in the across-species multi-predictor model (see above). We evaluated model fits using AIC and *r* ^2^, which was calculated as the fit between observed and predicted clutch size across effect types.

To investigate the spatial patterns in clutch size across assemblages, we compiled 2,521 lists of bird species found in 220 × 220-km grid cells across the globe. Grid cells with less than 50% land and under 30 bird species with clutch size data were excluded. For each grid cell assemblage, we calculated the geometric mean clutch size of its members, as observed and as predicted by the cross-species and nested phylogenetic multi-predictor models.

### Intraspecific variation.

Our analysis is based on species-typical intrinsic and extrinsic attributes that are integrated or averaged across all the individual and population level observations available and across the entire geographic range. This collapses the intraspecific variation to one single value, but has the advantage that values are buffered against between-site variation and potential population-level idiosyncrasies. We considered whether the substantial intraspecific variation in clutch size, which our analysis misses, may potentially bias observed patterns. Intraspecific clutch size variation may occur within individuals (within and between years) and between individuals (within and among populations). While a full global analysis at the various levels of within-species variation of clutch size would be desirable, the data do not allow it. However, we believe that natural selection on clutch size occurs at all listed levels of organization, and effects across species should be consistent and comparable to effects within species. Potential biases may arise if intrinsic or extrinsic within-species gradients were drastically different to those at the across-species level. This issue should increase in importance toward wide-ranging species as (i) their larger geographic spread would likely result in increased trait variance and (ii) average intrinsic and extrinsic attributes would be representative for a successively smaller subset of populations. Lacking standardized data on the intraspecific variance in clutch size across a representative set of species, we use as proxy the range of clutch sizes recorded for a species in the literature standardized by the mean (range to mean ratio). We find that thus-measured clutch size variance shows a weak, but significant increase with geographic range size: (log_10_-transformed ratio + 1) = −0.06 + 0.03 log_10_(geographic range size); *r* ^2^ = 0.05, *F*_1,5288_ = 263.6, *p* < 0.001). To examine the sensitivity of the results of our study to this variation, we selected the roughly half of all species for which the range-to-mean ratio was under 0.28, i.e., the species with either no recorded intraspecific variation (2,102 species), those with a mean clutch size of 4 that varied at most by one egg (276 species), and those with a mean clutch size of 7 that varied at most by two eggs (33 species). We then repeated our combined model (Table S3). All core results for this subset of species are broadly similar to those observed for all species. We conclude that the trends in our study should be robust to potential biases from intraspecific variation.

## Supporting Information

Figure S1 Data Sources for Geographic Range Maps (60 KB PDF)

Table S1 Cross-Correlation Matrix of Variables in the Analysis across all 5,290 Species Data Points (63 KB PDF)

Table S2 Single-Predictor Intrinsic and Extrinsic Effects on Clutch Size across Precocial Species and Altricial Species (15 KB PDF)

Table S3 Combined Models of Avian Clutch Size across the Approximately Half of Bird Species in the Analysis with Least Clutch Size Variation (13 KB PDF)

Table S4 List of Species and Data Sources Used for Geographic Distributions and Data on Clutch Sizes and Intrinsic Variables (562 KB PDF)

Table S5 List of Main Literature Sources Consulted, with Abbreviations (14 KB PDF)

Table S6 List of All Literature Sources Consulted (186 KB DOC)

## Abbreviations

AIC: Akaike information criterion
