# Supplementary material for: The Worldwide Variation in Avian Clutch Size across Species and Space
Source: PLoS Biol. 2008 Dec 9;6(12):e303. doi: 10.1371/journal.pbio.0060303 (PMC2596859; doi:10.1371/journal.pbio.0060303)
Supplement: Supplementary file 1 [file pbio.0060303.sg001.pdf]

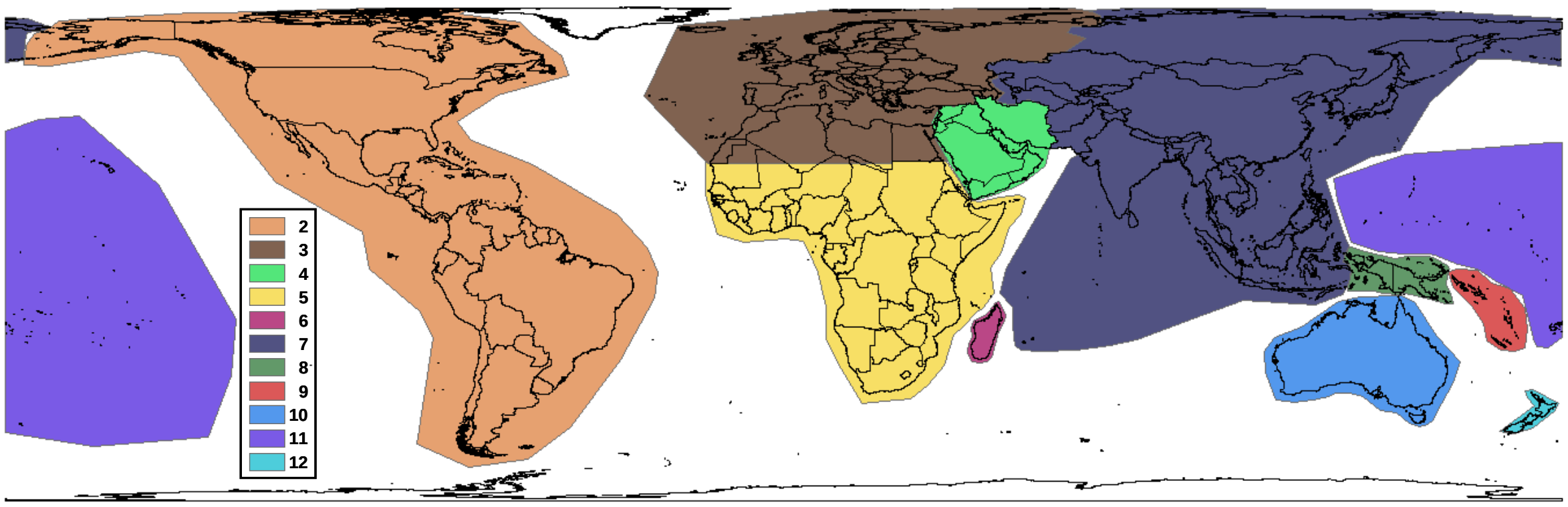

Fig. S1. Data sources for geographic range maps. A map illustrating the core references used for geographic sub-regions. Select additional sources such as island species lists were used.

Non-Passerines (not illustrated, global):

(1)

Hoyo, J. d., A. Elliott, and J. Sargatal 1992. Handbook of the birds of the world. Volume 1: Ostrich to Ducks. Lynx Edicions, Barcelona.

Elliott, A., J. Sargatal, and J. d. Hoyo 1994. Handbook of the birds of the world. Volume 2: New World Vultures to Guineafowl. Lynx Edicions, Barcelona.

Hoyo, J. d., A. Elliott, and J. Sargatal 1996. Handbook of the birds of the world. Volume 3: Hoatzin to Auks. Lynx Edicions, Barcelona.

Hoyo, J. d., A. Elliott, and J. Sargatal 1997. Handbook of the birds of the world. Volume 4: Sandgrouse to Cuckoos. Lynx Edicions, Barcelona.

Hoyo, J. d., A. Elliott, and J. Sargatal 1999. Handbook of the birds of the world. Volume 5: Barn Owls to Hummingbirds. Lynx Edicions, Barcelona.

Hoyo, J. d., A. Elliott, and J. Sargatal 2001. Handbook of the birds of the world. Volume 6: Mousebirds to Hornbills. Lynx Edicions, Barcelona.

Hoyo, J. d., A. Elliott, and J. Sargatal 2002. Handbook of the birds of the world. Volume 7: Jacamars to Woodpeckers. Lynx Edicions, Barcelona.

Passerines:

(2)

Ridgely, R. S., Allnutt, T. F., Brooks, T., McNicol, D. K., Mehlman, D. W., Young, B. E. & Zook, J. R. 2003. Digital Distribution Maps of the Birds of the Western Hemisphere, version 1.0. Arlington, Virginia, USA: NatureServe.

(3)

Snow, D., Perrins, C. M., Gillmor, R., Vincent, D. F. & Wilson, M. G. 1998. The birds of the Western Palearctic. Oxford: Oxford University Press. 2 Volumes.

Cramp, S. 1988. Handbook of the birds of Europe, the Middle East and North Africa : the birds of the Western Palearctic. Volume V, Tyrant flycatchers to thrushes. Oxford University Press, Oxford.

Cramp, S., and D. J. Brooks 1992. Handbook of the birds of Europe, the Middle East and North Africa : the birds of the Western Palearctic. Volume VI, Warblers. Oxford University Press, Oxford.

Cramp, S., C. M. Perrins, D. J. Brooks, and E. Dunn 1993. Handbook of the birds of Europe, the Middle East and North Africa : the birds of the Western Palearctic. Volume VII, Flycatchers to Shrikes Oxford University Press, Oxford.

Cramp, S., C. M. Perrins, D. J. Brooks, and E. Dunn 1994. Handbook of the birds of Europe, the Middle East and North Africa : the birds of the Western Palearctic. Volume VIII, Crows to finches Oxford University Press, Oxford.

Cramp, S., C. M. Perrins, D. J. Brooks, and E. Dunn 1994. Handbook of the birds of Europe, the Middle East and North Africa : the birds of the Western Palearctic. Volume IX, Buntings and new world warblers. Oxford University Press, Oxford.

(4)

Hèue, F. 1970. Les oiseaux du Proche et du Moyen Orient, de la Mâediterranâee aux contreforts de l'Himalaya. Paris: Editions. N. Boubee & Cie. 948 p.

Porter, R. F., Christensen, S. & Schiermacker-Hansen, P. 1996. Field guide to the birds of the Middle East. London. San Diego: Poyser; Academic Press. 460 p.

(5)

Keith, S., E. K. Urban, and C. H. Fry 1992. The Birds of Africa. Vol. 4: Broadbills to Chats. Academic Press, London & New York.

Urban, E. K., C. H. Fry, and S. Keith 1997. The Birds of Africa. Vol. 5: Thrushes to Puffback Flycatcher. Academic Press, London & New York.6. Langrand, O. 1990 Guide to the birds of Madagascar. New Haven, CT: Yale University Press. 364 p.

Fry, C. H., S. Keith, and E. K. Urban 2000. The Birds of Africa. Vol. 6: Picathartes to Oxpeckers. Academic Press, London & New York.

Fry, C. H., and S. Keith 2004. The Birds of Africa. Vol. 7: Sparrows to Buntings. Academic Press, London & New York.

(6)

Langrand, O. 1990. Guide to the birds of Madagascar. New Haven, CT: Yale University Press. 364 p.

(7)

Ding, T.-S. 2005. Distribution of Breeding Birds in East Asia (http://asianbird.zo.ntu.edu.tw/) (National University of Taiwan, Taiwan.

Ding, T.-S., Yuan, H.-W., Geng, S., Koh, C.-N. & Lee, P.-F. 2006. Macro-scale bird species richness patterns of the East Asian mainland and islands: energy, area and isolation. Journal of Biogeography 33, 683-693.

Flint, V. E., N. Bourso-Leland, and A. A. Kuznetsov 1984. A Field Guide to the Birds of Russia and Adjacent Territories. Princeton University Press, Princeton NJ.

Sinclair, I., and O. Langrand 1998. Birds of the Indian Ocean Islands. Struik Publishers, Cape Town.

(8)

Beehler, B. M., Pratt, T. K. & Zimmerman, D. A. 1986. Birds of New Guinea. Princeton, N.J.: Princeton University Press. 293 p.

Coates, B. J. 1985. The birds of Papua New Guinea : including the Bismarck Archipelago and Bougainville. Volume I. Alderley, Qld., Australia: Dove Publications. 464 p.

Coates, B. J. 1990. The birds of Papua New Guinea : including the Bismarck Archipelago and Bougainville. Volume II. Alderley, Qld., Australia: Dove Publications. 576 p.

(9)

Doughty, C. 1999. Birds of the Solomons, Vanuatu & New Caledonia. London: Christopher Helm, A &C Black. 206 p.

(10)

Simpson, K. & Day, N. 2004. Birds of Australia: Princeton University Press. 382 p.

(11)

Pratt, D., Bruner, P. L. & Berrett, D. G. 1987. A field guide to the birds of Hawaii and the tropical Pacific. Princeton: Princeton University Press. 409 p.

(12)

Bull PC, Gaze PD, Robertson CJR, Zealand OSoN 1985. The atlas of bird distribution in New Zealand. Wellington North, N.Z.: Ornithological Society of New Zealand. 296 p.

Heather, B., Robertson, H. & Onley, D. 2005. Field Guide to the Birds of New Zealand: Penguin Books Ltd. 432p.
