# Supplementary material for: The Worldwide Variation in Avian Clutch Size across Species and Space
Source: PLoS Biol. 2008 Dec 9;6(12):e303. doi: 10.1371/journal.pbio.0060303 (PMC2596859; doi:10.1371/journal.pbio.0060303)
Supplement: Supplementary file 2 [file pbio.0060303.st001.pdf]

Table S1: Cross-correlation matrix of variables in the analysis across all 5,290 species data points (Spearman rank correlations). Only continuous and categorical variables with 2 factors levels included. Correlations ≥ 0.75 are marked in bold. For variable abbreviations see Methods.

	Clutch size	*Precocial*	*Mass*	*Nest type*	*Migrant*	*Abs. Latitude*	*$Temp_{Avg}$*	*$Prec_{Total}$*	*NPP_{Total}*	*NPP_{Max}*	*$1 - (NPP_{Min} / NPP_{Max})$*	*$Temp_{Max} - Temp_{Min}$*
Clutch size		0.24	0.08	0.13	0.33	0.47	-0.39	-0.38	-0.43	-0.16	-0.46	0.52
Precocial	0.24		0.45	-0.20	0.17	0.16	-0.13	-0.16	-0.16	-0.07	-0.17	0.17
Mass	0.08	0.45		-0.03	0.07	0.08	-0.01	-0.08	-0.10	-0.08	-0.09	0.11
Nest type	0.13	-0.20	-0.03		-0.22	-0.15	0.16	0.11	0.15	0.05	0.12	-0.16
Migrant	0.33	0.17	0.07	-0.15		0.51	-0.51	-0.41	-0.41	-0.12	-0.46	0.55
Abs. Latitude	0.47	0.16	0.08	0.16	0.51		**-0.76**	-0.63	-0.69	-0.25	-0.73	**0.90**
$Temp_{Avg}$	-0.39	-0.13	-0.01	0.11	-0.51	**-0.76**		0.64	0.59	0.21	0.63	-0.75
$Prec_{Total}$	-0.38	-0.16	-0.08	0.13	-0.41	-0.63	0.64		**0.94**	0.62	**0.87**	**-0.78**
NPP_{Total}	-0.43	-0.16	-0.10	0.05	-0.41	-0.69	0.59	**0.94**		0.70	**0.90**	**-0.83**
NPP_{Max}	-0.16	-0.07	-0.08	0.12	-0.12	-0.25	0.21	0.62	0.70		0.38	-0.40
$1 - (NPP_{Min} / NPP_{Max})$	-0.46	-0.17	-0.09	-0.16	-0.46	-0.73	0.63	**0.87**	**0.90**	0.38		**-0.84**
$Temp_{Max} - Temp_{Min}$	0.52	0.17	0.11	-0.08	0.55	**0.90**	-0.75	**-0.78**	**-0.83**	-0.40	**-0.84**	
