# Supplementary material for: The Worldwide Variation in Avian Clutch Size across Species and Space
Source: PLoS Biol. 2008 Dec 9;6(12):e303. doi: 10.1371/journal.pbio.0060303 (PMC2596859; doi:10.1371/journal.pbio.0060303)
Supplement: Supplementary file 3 [file pbio.0060303.st002.pdf]

Table S2: Single predictor intrinsic and extrinsic effects on clutch size across precocial species (N = 864) and altricial species (N = 4,426). Cross-species models are linear models, Nested phylogenetic models are mixed effects models with each predictor as fixed effect and Order and Family (nested in Order) as random effects representing clade membership. Within each subgroup, all AIC values are comparable and best models are marked in bold. For other details see Table 1.

	**Cross-species**								**Nested phylogenetic**											
	**precocial**				**altricial**				**precocial**						**altricial**					
	b	t	AIC	r^2	b	t	AIC	r^2	b	t	AIC	r^2 fixed	r^2 Order	r^2 Family	b	t	AIC	r^2 fixed	r^2 Order	r^2 Family
Null			114	0.00			-3029				-659		0.42	0.66			-4923		0.09	0.39
intrinsic																				
Mass	0.09	6.44	75	0.05	-0.04	-9.14	-3134	0.02	-0.01	-0.84	-652	0.05	0.43	0.66	-0.05	-8.34	-4981	0.02	0.02	0.40
Nest type	0.06	4.67	94	0.02	0.04	14.03	-3242	0.04	-0.04	-4.13	-667	0.02	0.42	0.67	0.02	3.74	-4924	0.04	0.04	0.40
Diet			**-118**	**0.24**			-3501	0.10			**-711**	**0.09**	**0.47**	**0.69**			-5134	0.09	0.09	0.43
Migrant	0.02	1.30	114	0.00	**0.15**	**24.92**	**-3632**	**0.12**	0.07	5.39	-679	0.00	0.44	0.67	**0.14**	**26.87**	**-5581**	**0.12**	**0.12**	**0.48**
extrinsic																				
Abs. Latitude	0.00	8.24	50	0.07	0.01	38.22	-4314	0.25	0.00	8.99	-720	0.07	0.46	0.69	0.01	39.54	-6242	0.25	0.26	0.55
$Temp_{Avg}$	-1.36	-7.33	63	0.06	-2.82	-33.04	-4027	0.20	-0.99	-6.53	-697	0.06	0.45	0.68	-2.36	-31.75	-5826	0.20	0.20	0.51
$Prec_{Total}$	-0.12	-4.34	97	0.02	-0.20	-26.04	-3682	0.13	-0.18	-9.35	-734	0.02	0.44	0.69	-0.17	-25.26	-5509	0.13	0.13	0.47
NPP_{Total}	0.00	-5.52	86	0.03	0.00	-30.16	-3877	0.17	0.00	-10.35	-740	0.03	0.45	0.70	0.00	-29.39	-5687	0.17	0.17	0.49
NPP_{Max}	0.00	-0.13	115	0.00	0.00	-9.84	-3145	0.02	0.00	-3.46	-653	0.00	0.42	0.66	0.00	-10.61	-5012	0.02	0.02	0.41
$1 - (NPP_{Min} / NPP_{Max})$	0.25	6.60	73	0.05	0.30	34.22	-4090	0.21	0.30	11.96	-784	0.05	0.46	0.71	-0.24	-31.33	-5800	0.21	0.21	0.50
$Temp_{Max} - Temp_{Min}$	**0.21**	**9.33**	**32**	**0.09**	**0.25**	**42.29**	**-4553**	**0.29**	**0.21**	**13.67**	**-819**	**0.09**	**0.49**	**0.72**	**0.23**	**42.69**	**-6438**	**0.29**	**0.29**	**0.57**
Realm			37	0.10			-4094	0.21			-685	0.08	0.45	0.69			-5737	0.20	0.20	0.50
