# Supplementary material for: The Worldwide Variation in Avian Clutch Size across Species and Space
Source: PLoS Biol. 2008 Dec 9;6(12):e303. doi: 10.1371/journal.pbio.0060303 (PMC2596859; doi:10.1371/journal.pbio.0060303)
Supplement: Supplementary file 4 [file pbio.0060303.st003.pdf]

Table S3: Combined models of avian clutch size across the approximately half of bird species in the analysis with least clutch size variation (N = 2,411). The cross species null model AIC was -715 and the nested phylogenetic null model AIC (with only the effects of Order and Family fitted) was -2186. For other details see Table 1.

		**Cross species**				**Nested phylogenetic**			
		**All**				**All**			
		b	t		AIC	b	t		AIC
Mass		-0.09	-14.51	***	202	-0.09	-9.90	***	81
Migrant		0.03	2.49	*	29	0.03	2.80	**	-7
Precocial		-0.03	-0.75		-1	-0.02	-0.25		-6
Precocial | *Mass*		0.10	7.00	***	47	0.05	2.65	**	-1
Precocial | *Migrant*		-0.10	-5.73	***	69	-0.05	-2.69	**	-1
Nest type		0.07	15.25	***	222	0.02	3.70	***	3
	Vertebrates								
	Invertebrates	-0.03	-1.91			-0.01	-0.60		
Diet	*Omnivore*	0.05	1.92		140	0.13	4.58	***	77
	Plants & Seeds	0.07	4.05	***		0.06	2.77	**	
	Fruits & Nectar	-0.07	-4.01	***		-0.03	-1.49		
$Temp_{Max}$ - $Temp_{Min}$		0.21	16.49	***	257	0.19	17.02	***	264
NPP_{Max}		0.00	5.15	***	25	0.00	1.53		17
	Nearctic								
	Neotropics	-0.10	-6.25	***		-0.03	-2.12	*	
	Palearctic	-0.04	-2.19	*		-0.01	-1.03		
Realm	*Afrotropics*	-0.11	-6.25	***	87	-0.07	-5.12	***	0
	IndoMalay	-0.08	-4.27	**		-0.05	-3.35	*	
	Australasia	-0.15	-8.59	***		-0.08	-5.05	***	
	Oceania	-0.22	-4.50	***		-0.15	-3.93	***	
Model AIC			-2261				-3016		
r^2 fixed			0.48				0.41		
r^2 Order							0.53		
r^2 Family							0.69		
