# Supplementary material for: The Worldwide Variation in Avian Clutch Size across Species and Space
Source: PLoS Biol. 2008 Dec 9;6(12):e303. doi: 10.1371/journal.pbio.0060303 (PMC2596859; doi:10.1371/journal.pbio.0060303)
Supplement: Supplementary file 5 [file pbio.0060303.st004.pdf]

Table S4

A list of the species in the analysis, their geometric mean clutch size (Clutch), sources used for their geographic distributions (Range) and for data on clutch sizes and intrinsic variables (Traits). For details on source abbreviations see Fig. S1 and Table S5.

Order	Family	*Species*	Clutch	Range	Traits
Struthioniformes	Struthionidae	*Struthio camelus*	7.4	1	HBW1
Struthioniformes	Rheidae	*Rhea americana*	19.7	1	HBW1
Struthioniformes	Rheidae	*Rhea pennata*	17.3	1	HBW1
Struthioniformes	Casuariidae	*Casuarius casuarius*	3.9	1	HBW1
Struthioniformes	Casuariidae	*Casuarius bennetti*	3.9	1	HBW1
Struthioniformes	Casuariidae	*Dromaius novaehollandiae*	8.7	1	HBW1
Tinamiformes	Tinamidae	*Tinamus tao*	4.2	1	HBW1
Tinamiformes	Tinamidae	*Tinamus solitarius*	9.2	1	HBW1
Tinamiformes	Tinamidae	*Tinamus osgoodi*	2.0	1	HBW1
Tinamiformes	Tinamidae	*Tinamus major*	4.2	1	HBW1
Tinamiformes	Tinamidae	*Tinamus guttatus*	5.5	1	HBW1
Tinamiformes	Tinamidae	*Nothocercus bonapartei*	4.5	1	HBW1
Tinamiformes	Tinamidae	*Crypturellus cinereus*	2.0	1	HBW1
Tinamiformes	Tinamidae	*Crypturellus soui*	2.0	1	HBW1
Tinamiformes	Tinamidae	*Crypturellus obsoletus*	4.5	1	HBW1
Tinamiformes	Tinamidae	*Crypturellus cinnamomeus*	3.0	1	HBW1
Tinamiformes	Tinamidae	*Crypturellus undulatus*	4.5	1	HBW1
Tinamiformes	Tinamidae	*Crypturellus transfasciatus*	2.6	1	HBW1
Tinamiformes	Tinamidae	*Crypturellus strigulosus*	4.0	1	HBW1
Tinamiformes	Tinamidae	*Crypturellus boucardi*	3.7	1	HBW1
Tinamiformes	Tinamidae	*Crypturellus erythropus*	3.0	1	HBW1
Tinamiformes	Tinamidae	*Crypturellus noctivagus*	4.0	1	HBW1
Tinamiformes	Tinamidae	*Crypturellus variegatus*	1.0	1	HBW1
Tinamiformes	Tinamidae	*Crypturellus parvirostris*	4.5	1	HBW1
Tinamiformes	Tinamidae	*Crypturellus tataupa*	4.5	1	HBW1
Tinamiformes	Tinamidae	*Rhynchotus rufescens*	2.2	1	HBW1
Tinamiformes	Tinamidae	*Nothoprocta ornata*	6.0	1	HBW1
Tinamiformes	Tinamidae	*Nothoprocta pentlandii*	6.3	1	HBW1
Tinamiformes	Tinamidae	*Nothoprocta cinerascens*	9.4	1	HBW1
Tinamiformes	Tinamidae	*Nothoprocta perdicaria*	6.3	1	HBW1
Tinamiformes	Tinamidae	*Nothura darwinii*	4.9	1	HBW1
Tinamiformes	Tinamidae	*Nothura maculosa*	4.9	1	HBW1
Tinamiformes	Tinamidae	*Nothura boraquira*	4.0	1	HBW1
Tinamiformes	Tinamidae	*Eudromia elegans*	5.5	1	HBW1
Tinamiformes	Tinamidae	*Tinamotis pentlandii*	5.3	1	HBW1
Tinamiformes	Tinamidae	*Tinamotis ingoufi*	11.0	1	HBW1
Craciformes	Cracidae	*Ortalis vetula*	3.0	1	HBW2
Craciformes	Cracidae	*Ortalis cinereiceps*	3.0	1	HBW2
Craciformes	Cracidae	*Ortalis garrula*	3.0	1	HBW2
Craciformes	Cracidae	*Ortalis ruficauda*	3.5	1	HBW2
Craciformes	Cracidae	*Ortalis erythroptera*	3.0	1	HBW2
Craciformes	Cracidae	*Ortalis wagleri*	3.0	1	HBW2
Craciformes	Cracidae	*Ortalis canicollis*	3.5	1	HBW2
Craciformes	Cracidae	*Ortalis leucogastra*	2.4	1	HBW2
Craciformes	Cracidae	*Ortalis motmot*	3.0	1	HBW2
Craciformes	Cracidae	*Ortalis superciliaris*	2.4	1	HBW2
Craciformes	Cracidae	*Pipile pipile*	2.0	1	HBW2
Craciformes	Cracidae	*Pipile cumanensis*	3.0	1	HBW2
Craciformes	Cracidae	*Pipile jacutinga*	2.4	1	HBW2
Craciformes	Cracidae	*Crax rubra*	2.0	1	HBW2
Craciformes	Cracidae	*Crax daubentoni*	2.0	1	HBW2
Craciformes	Cracidae	*Crax alector*	2.0	1	HBW2
Craciformes	Cracidae	*Crax globulosa*	2.0	1	HBW2
Craciformes	Cracidae	*Crax fasciolata*	2.0	1	HBW2
Craciformes	Cracidae	*Crax blumenbachii*	2.0	1	HBW2

Order	Family	*Species*	Clutch	Range	Traits
Craciformes	Megapodiidae	*Alectura lathami*	12.0	1	HBW2
Craciformes	Megapodiidae	*Aepypodius arfakianus*	20.0	1	HBW2
Craciformes	Megapodiidae	*Leipoa ocellata*	12.8	1	HBW2
Galliformes	Phasianidae	*Lerwa lerwa*	3.2	1	HBW2
Galliformes	Phasianidae	*Ammoperdix griseogularis*	7.3	1	HBW2
Galliformes	Phasianidae	*Ammoperdix heyi*	5.9	1	HBW2
Galliformes	Phasianidae	*Alectoris barbara*	11.0	1	HBW2
Galliformes	Phasianidae	*Alectoris melanocephala*	6.3	1	HBW2
Galliformes	Phasianidae	*Alectoris chukar*	9.2	1	HBW2
Galliformes	Phasianidae	*Alectoris philbyi*	6.3	1	HBW2
Galliformes	Phasianidae	*Alectoris graeca*	10.6	1	HBW2
Galliformes	Phasianidae	*Alectoris rufa*	12.0	1	HBW2
Galliformes	Phasianidae	*Francolinus francolinus*	9.2	1	HBW2
Galliformes	Phasianidae	*Francolinus pictus*	4.9	1	HBW2
Galliformes	Phasianidae	*Francolinus pintadeanus*	4.5	1	HBW2
Galliformes	Phasianidae	*Francolinus pondicerianus*	7.3	1	HBW2
Galliformes	Phasianidae	*Francolinus gularis*	3.5	1	HBW2
Galliformes	Phasianidae	*Peliperdix coqui*	4.5	1	HBW2
Galliformes	Phasianidae	*Peliperdix albogularis*	6.0	1	HBW2
Galliformes	Phasianidae	*Peliperdix schlegelii*	3.2	1	HBW2
Galliformes	Phasianidae	*Peliperdix lathami*	2.0	1	HBW2
Galliformes	Phasianidae	*Peliperdix sephaena*	6.0	1	HBW2
Galliformes	Phasianidae	*Scleroptila streptophorus*	4.5	1	HBW2
Galliformes	Phasianidae	*Scleroptila finschi*	5.0	1	HBW2
Galliformes	Phasianidae	*Scleroptila africanus*	4.9	1	HBW2
Galliformes	Phasianidae	*Scleroptila levaillantii*	6.0	1	HBW2
Galliformes	Phasianidae	*Scleroptila shelleyi*	3.9	1	HBW2
Galliformes	Phasianidae	*Scleroptila levaillantoides*	6.3	1	HBW2
Galliformes	Phasianidae	*Pternistis nahani*	4.0	1	HBW2
Galliformes	Phasianidae	*Pternistis hartlaubi*	5.7	1	HBW2
Galliformes	Phasianidae	*Pternistis bicalcaratus*	6.0	1	HBW2
Galliformes	Phasianidae	*Pternistis icterorhynchus*	6.9	1	HBW2
Galliformes	Phasianidae	*Pternistis adspersus*	6.3	1	HBW2
Galliformes	Phasianidae	*Pternistis capensis*	6.9	1	HBW2
Galliformes	Phasianidae	*Pternistis hildebrandti*	6.0	1	HBW2
Galliformes	Phasianidae	*Pternistis natalensis*	5.0	1	HBW2
Galliformes	Phasianidae	*Pternistis ahantensis*	4.9	1	HBW2
Galliformes	Phasianidae	*Pternistis squamatus*	6.0	1	HBW2
Galliformes	Phasianidae	*Pternistis leucoscepus*	5.0	1	HBW2
Galliformes	Phasianidae	*Pternistis rufopictus*	4.5	1	HBW2
Galliformes	Phasianidae	*Pternistis afer*	5.2	1	HBW2
Galliformes	Phasianidae	*Pternistis swainsonii*	5.7	1	HBW2
Galliformes	Phasianidae	*Pternistis erckelii*	6.3	1	HBW2
Galliformes	Phasianidae	*Pternistis castaneicollis*	5.5	1	HBW2
Galliformes	Phasianidae	*Pternistis jacksoni*	3.0	1	HBW2
Galliformes	Phasianidae	*Perdix perdix*	16.0	1	HBW2
Galliformes	Phasianidae	*Perdix dauurica*	19.0	1	HBW2
Galliformes	Phasianidae	*Perdix hodgsoniae*	8.9	1	HBW2
Galliformes	Phasianidae	*Rhizothera longirostris*	3.2	1	HBW2
Galliformes	Phasianidae	*Coturnix coturnix*	10.2	1	HBW2
Galliformes	Phasianidae	*Coturnix japonica*	9.5	1	HBW2
Galliformes	Phasianidae	*Coturnix pectoralis*	7.5	1	HBW2
Galliformes	Phasianidae	*Coturnix coromandelica*	4.9	1	HBW2
Galliformes	Phasianidae	*Coturnix delegorguei*	5.7	1	HBW2
Galliformes	Phasianidae	*Coturnix ypsilophora*	8.8	1	HBW2
Galliformes	Phasianidae	*Coturnix adansonii*	5.2	1	HBW2
Galliformes	Phasianidae	*Coturnix chinensis*	5.3	1	HBW2
Galliformes	Phasianidae	*Perdicula asiatica*	5.5	1	HBW2
Galliformes	Phasianidae	*Perdicula argoondah*	5.5	1	HBW2
Galliformes	Phasianidae	*Perdicula erythrorhyncha*	5.3	1	HBW2
Galliformes	Phasianidae	*Perdicula manipurensis*	4.0	1	HBW2
Galliformes	Phasianidae	*Arborophila torqueola*	3.9	1	HBW2
Galliformes	Phasianidae	*Arborophila rufogularis*	4.5	1	HBW2

Order	Family	*Species*	Clutch	Range	Traits
Galliformes	Phasianidae	*Arborophila atrogularis*	4.5	1	HBW2
Galliformes	Phasianidae	*Arborophila crudigularis*	6.9	1	HBW2
Galliformes	Phasianidae	*Arborophila mandellii*	4.0	1	HBW2
Galliformes	Phasianidae	*Arborophila rufipectus*	5.5	1	HBW2
Galliformes	Phasianidae	*Arborophila orientalis*	2.8	1	HBW2
Galliformes	Phasianidae	*Arborophila javanica*	4.0	1	HBW2
Galliformes	Phasianidae	*Arborophila gingica*	5.9	1	HBW2
Galliformes	Phasianidae	*Arborophila ardens*	2.4	1	HBW2
Galliformes	Phasianidae	*Arborophila chloropus*	3.0	1	HBW2
Galliformes	Phasianidae	*Caloperdix oculea*	8.9	1	HBW2
Galliformes	Phasianidae	*Rollulus rouloul*	5.5	1	HBW2
Galliformes	Phasianidae	*Ptilopachus petrosus*	4.9	1	HBW2
Galliformes	Phasianidae	*Bambusicola fytchii*	4.9	1	HBW2
Galliformes	Phasianidae	*Bambusicola thoracica*	4.6	1	HBW2
Galliformes	Phasianidae	*Galloperdix spadicea*	3.2	1	HBW2
Galliformes	Phasianidae	*Galloperdix lunulata*	3.5	1	HBW2
Galliformes	Phasianidae	*Galloperdix bicalcarata*	2.0	1	HBW2
Galliformes	Phasianidae	*Ithaginis cruentus*	3.7	1	HBW2
Galliformes	Phasianidae	*Tragopan melanocephalus*	4.2	1	HBW2
Galliformes	Phasianidae	*Tragopan satyra*	2.4	1	HBW2
Galliformes	Phasianidae	*Tragopan blythii*	3.2	1	HBW2
Galliformes	Phasianidae	*Tragopan temminckii*	3.9	1	HBW2
Galliformes	Phasianidae	*Tragopan caboti*	3.9	1	HBW2
Galliformes	Phasianidae	*Lophophorus impejanus*	3.9	1	HBW2
Galliformes	Phasianidae	*Lophophorus sclateri*	5.0	1	HBW2
Galliformes	Phasianidae	*Lophophorus lhuysii*	3.9	1	HBW2
Galliformes	Phasianidae	*Gallus gallus*	6.0	1	HBW2
Galliformes	Phasianidae	*Gallus sonneratii*	4.5	1	HBW2
Galliformes	Phasianidae	*Gallus lafayetii*	2.0	1	HBW2
Galliformes	Phasianidae	*Gallus varius*	3.5	1	HBW2
Galliformes	Phasianidae	*Lophura leucomelanos*	7.3	1	HBW2
Galliformes	Phasianidae	*Lophura nycthemera*	6.5	1	HBW2
Galliformes	Phasianidae	*Lophura edwardsi*	5.3	1	HBW2
Galliformes	Phasianidae	*Lophura swinhoii*	5.7	1	HBW2
Galliformes	Phasianidae	*Lophura inornata*	2.0	1	HBW2
Galliformes	Phasianidae	*Lophura erythrophthalma*	4.2	1	HBW2
Galliformes	Phasianidae	*Lophura ignita*	5.7	1	HBW2
Galliformes	Phasianidae	*Lophura diardi*	5.7	1	HBW2
Galliformes	Phasianidae	*Lophura bulweri*	1.0	1	HBW2
Galliformes	Phasianidae	*Crossoptilon crossoptilon*	6.0	1	HBW2
Galliformes	Phasianidae	*Crossoptilon mantchuricum*	7.5	1	HBW2
Galliformes	Phasianidae	*Crossoptilon auritum*	8.0	1	HBW2
Galliformes	Phasianidae	*Phasianus colchicus*	9.9	1	HBW2
Galliformes	Phasianidae	*Chrysolophus pictus*	7.7	1	HBW2
Galliformes	Phasianidae	*Chrysolophus amherstiae*	6.5	1	HBW2
Galliformes	Phasianidae	*Polyplectron chalcurum*	2.0	1	HBW2
Galliformes	Phasianidae	*Polyplectron inopinatum*	2.0	1	HBW2
Galliformes	Phasianidae	*Polyplectron germaini*	1.4	1	HBW2
Galliformes	Phasianidae	*Polyplectron bicalcaratum*	2.0	1	HBW2
Galliformes	Phasianidae	*Polyplectron malacense*	1.0	1	HBW2
Galliformes	Phasianidae	*Polyplectron schleiermacheri*	1.0	1	HBW2
Galliformes	Phasianidae	*Polyplectron emphanum*	2.0	1	HBW2
Galliformes	Phasianidae	*Argusianus argus*	2.0	1	HBW2
Galliformes	Phasianidae	*Afropavo congensis*	2.4	1	HBW2
Galliformes	Phasianidae	*Falcipennis falcipennis*	7.7	1	HBW2
Galliformes	Phasianidae	*Falcipennis canadensis*	5.5	1	HBW2
Galliformes	Phasianidae	*Dendragapus obscurus*	6.9	1	HBW2
Galliformes	Phasianidae	*Lagopus lagopus*	9.4	1	HBW2
Galliformes	Phasianidae	*Lagopus mutus*	6.3	1	HBW2
Galliformes	Phasianidae	*Lagopus leucurus*	5.5	1	HBW2
Galliformes	Phasianidae	*Tetrao tetrix*	8.0	1	HBW2
Galliformes	Phasianidae	*Tetrao mlokosiewiczi*	6.0	1	HBW2
Galliformes	Phasianidae	*Tetrao urogallus*	7.3	1	HBW2

Order	Family	*Species*	Clutch	Range	Traits
Galliformes	Phasianidae	*Tetrao parvirostris*	6.5	1	HBW2
Galliformes	Phasianidae	*Bonasa bonasia*	8.8	1	HBW2
Galliformes	Phasianidae	*Bonasa sewerzowi*	6.3	1	HBW2
Galliformes	Phasianidae	*Bonasa umbellus*	11.0	1	HBW2
Galliformes	Phasianidae	*Centrocercus urophasianus*	7.5	1	HBW2
Galliformes	Phasianidae	*Tympanuchus phasianellus*	12.0	1	HBW2
Galliformes	Phasianidae	*Tympanuchus cupido*	10.2	1	HBW2
Galliformes	Phasianidae	*Tympanuchus pallidicinctus*	11.0	1	HBW2
Galliformes	Phasianidae	*Meleagris gallopavo*	11.4	1	HBW2
Galliformes	Phasianidae	*Meleagris ocellata*	12.0	1	HBW2
Galliformes	Numididae	*Numida meleagris*	8.5	1	HBW2
Galliformes	Numididae	*Guttera plumifera*	9.5	1	HBW2
Galliformes	Numididae	*Guttera pucherani*	4.5	1	HBW2
Galliformes	Numididae	*Acryllium vulturinum*	14.0	1	HBW2
Galliformes	Odontophoridae	*Dendrortyx barbatus*	3.5	1	HBW2
Galliformes	Odontophoridae	*Dendrortyx macroura*	4.0	1	HBW2
Galliformes	Odontophoridae	*Dendrortyx leucophrys*	4.5	1	HBW2
Galliformes	Odontophoridae	*Oreortyx pictus*	9.5	1	HBW2
Galliformes	Odontophoridae	*Callipepla squamata*	13.0	1	HBW2
Galliformes	Odontophoridae	*Callipepla douglasii*	9.8	1	HBW2
Galliformes	Odontophoridae	*Callipepla californica*	14.9	1	HBW2
Galliformes	Odontophoridae	*Callipepla gambelii*	13.0	1	HBW2
Galliformes	Odontophoridae	*Philortyx fasciatus*	5.5	1	HBW2
Galliformes	Odontophoridae	*Colinus virginianus*	8.0	1	HBW2
Galliformes	Odontophoridae	*Colinus cristatus*	11.3	1	HBW2
Galliformes	Odontophoridae	*Odontophorus gujanensis*	4.0	1	HBW2
Galliformes	Odontophoridae	*Odontophorus capueira*	5.0	1	HBW2
Galliformes	Odontophoridae	*Odontophorus melanotis*	4.0	1	HBW2
Galliformes	Odontophoridae	*Odontophorus columbianus*	6.0	1	HBW2
Galliformes	Odontophoridae	*Odontophorus leucolaemus*	5.0	1	HBW2
Galliformes	Odontophoridae	*Cyrtonyx montezumae*	11.0	1	HBW2
Anseriformes	Anhimidae	*Anhima cornuta*	3.9	1	HBW1
Anseriformes	Anhimidae	*Chauna chavaria*	3.9	1	HBW1
Anseriformes	Anhimidae	*Chauna torquata*	3.9	1	HBW1
Anseriformes	Anseranatidae	*Anseranas semipalmata*	7.4	1	HBW1
Anseriformes	Dendrocygnidae	*Dendrocygna guttata*	11.0	1	HBW1
Anseriformes	Dendrocygnidae	*Dendrocygna eytoni*	10.6	1	HBW1
Anseriformes	Dendrocygnidae	*Dendrocygna bicolor*	9.8	1	HBW1
Anseriformes	Dendrocygnidae	*Dendrocygna arcuata*	9.5	1	HBW1
Anseriformes	Dendrocygnidae	*Dendrocygna javanica*	9.2	1	HBW1
Anseriformes	Dendrocygnidae	*Dendrocygna viduata*	7.2	1	HBW1
Anseriformes	Dendrocygnidae	*Dendrocygna arborea*	8.5	1	HBW1
Anseriformes	Dendrocygnidae	*Dendrocygna autumnalis*	13.9	1	HBW1
Anseriformes	Anatidae	*Oxyura jamaicensis*	7.7	1	HBW1
Anseriformes	Anatidae	*Oxyura leucocephala*	6.3	1	HBW1
Anseriformes	Anatidae	*Oxyura maccoa*	5.7	1	HBW1
Anseriformes	Anatidae	*Oxyura vittata*	3.9	1	HBW1
Anseriformes	Anatidae	*Oxyura australis*	5.5	1	HBW1
Anseriformes	Anatidae	*Biziura lobata*	2.4	1	HBW1
Anseriformes	Anatidae	*Stictonetta naevosa*	7.1	1	HBW1
Anseriformes	Anatidae	*Cygnus olor*	5.9	1	HBW1
Anseriformes	Anatidae	*Cygnus atratus*	5.5	1	HBW1
Anseriformes	Anatidae	*Cygnus melanocorypha*	5.7	1	HBW1
Anseriformes	Anatidae	*Cygnus cygnus*	4.5	1	HBW1
Anseriformes	Anatidae	*Cygnus buccinator*	5.7	1	HBW1
Anseriformes	Anatidae	*Cygnus columbianus*	3.9	1	HBW1
Anseriformes	Anatidae	*Coscoroba coscoroba*	5.3	1	HBW1
Anseriformes	Anatidae	*Anser cygnoides*	5.5	1	HBW1
Anseriformes	Anatidae	*Anser brachyrhynchus*	3.9	1	HBW1
Anseriformes	Anatidae	*Anser fabalis*	4.9	1	HBW1
Anseriformes	Anatidae	*Anser albifrons*	5.5	1	HBW1
Anseriformes	Anatidae	*Anser erythropus*	4.9	1	HBW1
Anseriformes	Anatidae	*Anser anser*	4.9	1	HBW1

Order	Family	*Species*	Clutch	Range	Traits
Anseriformes	Anatidae	*Anser indicus*	4.9	1	HBW1
Anseriformes	Anatidae	*Anser caerulescens*	4.5	1	HBW1
Anseriformes	Anatidae	*Anser rossii*	4.5	1	HBW1
Anseriformes	Anatidae	*Anser canagica*	5.0	1	HBW1
Anseriformes	Anatidae	*Branta sandvicensis*	3.9	1	HBW1
Anseriformes	Anatidae	*Branta canadensis*	5.3	1	HBW1
Anseriformes	Anatidae	*Branta leucopsis*	4.5	1	HBW1
Anseriformes	Anatidae	*Branta bernicla*	3.9	1	HBW1
Anseriformes	Anatidae	*Branta ruficollis*	6.5	1	HBW1
Anseriformes	Anatidae	*Cereopsis novaehollandiae*	4.2	1	HBW1
Anseriformes	Anatidae	*Sarkidiornis melanotos*	11.0	1	HBW1
Anseriformes	Anatidae	*Tadorna ferruginea*	8.5	1	HBW1
Anseriformes	Anatidae	*Tadorna cana*	10.0	1	HBW1
Anseriformes	Anatidae	*Tadorna tadornoides*	8.9	1	HBW1
Anseriformes	Anatidae	*Tadorna variegata*	8.9	1	HBW1
Anseriformes	Anatidae	*Tadorna tadorna*	8.9	1	HBW1
Anseriformes	Anatidae	*Tadorna radjah*	8.5	1	HBW1
Anseriformes	Anatidae	*Malacorhynchus membranaceu*	6.9	1	HBW1
Anseriformes	Anatidae	*Neochen jubata*	7.7	1	HBW1
Anseriformes	Anatidae	*Chloephaga melanoptera*	7.1	1	HBW1
Anseriformes	Anatidae	*Chloephaga picta*	6.3	1	HBW1
Anseriformes	Anatidae	*Chloephaga hybrida*	4.6	1	HBW1
Anseriformes	Anatidae	*Chloephaga poliocephala*	4.9	1	HBW1
Anseriformes	Anatidae	*Chloephaga rubidiceps*	6.3	1	HBW1
Anseriformes	Anatidae	*Merganetta armata*	3.5	1	HBW1
Anseriformes	Anatidae	*Pteronetta hartlaubii*	8.8	1	HBW1
Anseriformes	Anatidae	*Cairina moschata*	11.0	1	HBW1
Anseriformes	Anatidae	*Cairina scutulata*	8.8	1	HBW1
Anseriformes	Anatidae	*Aix sponsa*	11.6	1	HBW1
Anseriformes	Anatidae	*Aix galericulata*	10.4	1	HBW1
Anseriformes	Anatidae	*Nettapus pulchellus*	9.8	1	HBW1
Anseriformes	Anatidae	*Nettapus coromandelianus*	9.8	1	HBW1
Anseriformes	Anatidae	*Nettapus auritus*	8.5	1	HBW1
Anseriformes	Anatidae	*Callonetta leucophrys*	8.5	1	HBW1
Anseriformes	Anatidae	*Chenonetta jubata*	9.4	1	HBW1
Anseriformes	Anatidae	*Amazonetta brasiliensis*	6.9	1	HBW1
Anseriformes	Anatidae	*Anas specularis*	4.9	1	HBW1
Anseriformes	Anatidae	*Anas capensis*	8.1	1	HBW1
Anseriformes	Anatidae	*Anas strepera*	9.8	1	HBW1
Anseriformes	Anatidae	*Anas falcata*	7.3	1	HBW1
Anseriformes	Anatidae	*Anas penelope*	8.5	1	HBW1
Anseriformes	Anatidae	*Anas americana*	7.9	1	HBW1
Anseriformes	Anatidae	*Anas sibilatrix*	6.3	1	HBW1
Anseriformes	Anatidae	*Anas sparsa*	5.7	1	HBW1
Anseriformes	Anatidae	*Anas rubripes*	9.2	1	HBW1
Anseriformes	Anatidae	*Anas platyrhynchos*	10.8	1	HBW1
Anseriformes	Anatidae	*Anas poecilorhyncha*	7.9	1	HBW1
Anseriformes	Anatidae	*Anas superciliosa*	8.9	1	HBW1
Anseriformes	Anatidae	*Anas luzonica*	10.0	1	HBW1
Anseriformes	Anatidae	*Anas undulata*	6.9	1	HBW1
Anseriformes	Anatidae	*Anas melleri*	7.1	1	HBW1
Anseriformes	Anatidae	*Anas discors*	9.4	1	HBW1
Anseriformes	Anatidae	*Anas cyanoptera*	10.4	1	HBW1
Anseriformes	Anatidae	*Anas smithii*	7.7	1	HBW1
Anseriformes	Anatidae	*Anas platalea*	6.3	1	HBW1
Anseriformes	Anatidae	*Anas rhynchotis*	9.9	1	HBW1
Anseriformes	Anatidae	*Anas clypeata*	9.9	1	HBW1
Anseriformes	Anatidae	*Anas bernieri*	4.5	1	HBW1
Anseriformes	Anatidae	*Anas gibberifrons*	7.5	1	HBW1
Anseriformes	Anatidae	*Anas castanea*	8.4	1	HBW1
Anseriformes	Anatidae	*Anas aucklandica*	5.5	1	HBW1
Anseriformes	Anatidae	*Anas bahamensis*	7.7	1	HBW1
Anseriformes	Anatidae	*Anas erythrorhyncha*	7.7	1	HBW1

Order	Family	*Species*	Clutch	Range	Traits
Anseriformes	Anatidae	*Anas flavirostris*	6.3	1	HBW1
Anseriformes	Anatidae	*Anas acuta*	7.9	1	HBW1
Anseriformes	Anatidae	*Anas georgica*	6.3	1	HBW1
Anseriformes	Anatidae	*Anas querquedula*	8.5	1	HBW1
Anseriformes	Anatidae	*Anas formosa*	7.3	1	HBW1
Anseriformes	Anatidae	*Anas crecca*	9.4	1	HBW1
Anseriformes	Anatidae	*Anas versicolor*	7.7	1	HBW1
Anseriformes	Anatidae	*Anas hottentota*	7.3	1	HBW1
Anseriformes	Anatidae	*Marmaronetta angustirostris*	9.9	1	HBW1
Anseriformes	Anatidae	*Netta rufina*	8.9	1	HBW1
Anseriformes	Anatidae	*Netta peposaca*	10.0	1	HBW1
Anseriformes	Anatidae	*Netta erythrophthalma*	8.7	1	HBW1
Anseriformes	Anatidae	*Aythya ferina*	8.9	1	HBW1
Anseriformes	Anatidae	*Aythya valisineria*	9.5	1	HBW1
Anseriformes	Anatidae	*Aythya americana*	9.0	1	HBW1
Anseriformes	Anatidae	*Aythya collaris*	8.9	1	HBW1
Anseriformes	Anatidae	*Aythya nyroca*	8.9	1	HBW1
Anseriformes	Anatidae	*Aythya baeri*	7.7	1	HBW1
Anseriformes	Anatidae	*Aythya australis*	10.8	1	HBW1
Anseriformes	Anatidae	*Aythya fuligula*	9.4	1	HBW1
Anseriformes	Anatidae	*Aythya novaeseelandiae*	5.7	1	HBW1
Anseriformes	Anatidae	*Aythya marila*	9.4	1	HBW1
Anseriformes	Anatidae	*Aythya affinis*	9.9	1	HBW1
Anseriformes	Anatidae	*Somateria mollissima*	4.9	1	HBW1
Anseriformes	Anatidae	*Somateria spectabilis*	4.5	1	HBW1
Anseriformes	Anatidae	*Somateria fischeri*	4.5	1	HBW1
Anseriformes	Anatidae	*Polysticta stelleri*	6.9	1	HBW1
Anseriformes	Anatidae	*Histrionicus histrionicus*	5.9	1	HBW1
Anseriformes	Anatidae	*Clangula hyemalis*	7.3	1	HBW1
Anseriformes	Anatidae	*Melanitta nigra*	6.9	1	HBW1
Anseriformes	Anatidae	*Melanitta perspicillata*	6.3	1	HBW1
Anseriformes	Anatidae	*Melanitta fusca*	7.9	1	HBW1
Anseriformes	Anatidae	*Bucephala clangula*	9.4	1	HBW1
Anseriformes	Anatidae	*Bucephala islandica*	9.4	1	HBW1
Anseriformes	Anatidae	*Bucephala albeola*	7.7	1	HBW1
Anseriformes	Anatidae	*Mergellus albellus*	7.9	1	HBW1
Anseriformes	Anatidae	*Lophodytes cucullatus*	11.0	1	HBW1
Anseriformes	Anatidae	*Mergus serrator*	8.9	1	HBW1
Anseriformes	Anatidae	*Mergus squamatus*	10.0	1	HBW1
Anseriformes	Anatidae	*Mergus merganser*	9.8	1	HBW1
Turniciformes	Turnicidae	*Turnix sylvatica*	3.7	1	HBW3
Turniciformes	Turnicidae	*Turnix maculosa*	4.0	1	HBW3
Turniciformes	Turnicidae	*Turnix hottentotta*	3.0	1	HBW3
Turniciformes	Turnicidae	*Turnix tanki*	4.0	1	HBW3
Turniciformes	Turnicidae	*Turnix suscitator*	4.2	1	HBW3
Turniciformes	Turnicidae	*Turnix nigricollis*	3.9	1	HBW3
Turniciformes	Turnicidae	*Turnix melanogaster*	3.5	1	HBW3
Turniciformes	Turnicidae	*Turnix castanota*	4.0	1	HBW3
Turniciformes	Turnicidae	*Turnix olivii*	3.5	1	HBW3
Turniciformes	Turnicidae	*Turnix varia*	3.5	1	HBW3
Turniciformes	Turnicidae	*Turnix everetti*	2.4	1	HBW3
Turniciformes	Turnicidae	*Turnix pyrrhothorax*	4.0	1	HBW3
Turniciformes	Turnicidae	*Turnix velox*	3.5	1	HBW3
Turniciformes	Turnicidae	*Ortyxelos meiffrenii*	2.0	1	HBW3
Piciformes	Indicatoridae	*Indicator variegatus*	2.0	1	HBW7
Piciformes	Indicatoridae	*Indicator indicator*	4.0	1	HBW7
Piciformes	Indicatoridae	*Indicator minor*	2.8	1	HBW7
Piciformes	Indicatoridae	*Prodotiscus insignis*	2.0	1	HBW7
Piciformes	Indicatoridae	*Prodotiscus zambesiae*	2.0	1	HBW7
Piciformes	Indicatoridae	*Prodotiscus regulus*	5.5	1	HBW7
Piciformes	Picidae	*Jynx torquilla*	9.2	1	HBW7
Piciformes	Picidae	*Jynx ruficollis*	3.5	1	HBW7
Piciformes	Picidae	*Picumnus innominatus*	2.8	1	HBW7

Order	Family	*Species*	Clutch	Range	Traits
Piciformes	Picidae	*Picumnus lafresnayi*	2.0	1	HBW7
Piciformes	Picidae	*Picumnus minutissimus*	2.4	1	HBW7
Piciformes	Picidae	*Picumnus cirratus*	2.8	1	HBW7
Piciformes	Picidae	*Picumnus olivaceus*	2.4	1	HBW7
Piciformes	Picidae	*Sasia africana*	2.0	1	HBW7
Piciformes	Picidae	*Sasia abnormis*	2.4	1	HBW7
Piciformes	Picidae	*Sasia ochracea*	2.8	1	HBW7
Piciformes	Picidae	*Nesoctites micromegas*	2.8	1	HBW7
Piciformes	Picidae	*Melanerpes candidus*	3.5	1	HBW7
Piciformes	Picidae	*Melanerpes lewis*	6.5	1	HBW7
Piciformes	Picidae	*Melanerpes erythrocephalus*	5.3	1	HBW7
Piciformes	Picidae	*Melanerpes formicivorus*	4.9	1	HBW7
Piciformes	Picidae	*Melanerpes pucherani*	2.8	1	HBW7
Piciformes	Picidae	*Melanerpes chrysauchen*	3.5	1	HBW7
Piciformes	Picidae	*Melanerpes striatus*	4.9	1	HBW7
Piciformes	Picidae	*Melanerpes radiolatus*	3.9	1	HBW7
Piciformes	Picidae	*Melanerpes rubricapillus*	3.5	1	HBW7
Piciformes	Picidae	*Melanerpes uropygialis*	3.9	1	HBW7
Piciformes	Picidae	*Melanerpes carolinus*	4.9	1	HBW7
Piciformes	Picidae	*Melanerpes superciliaris*	4.5	1	HBW7
Piciformes	Picidae	*Melanerpes aurifrons*	4.5	1	HBW7
Piciformes	Picidae	*Melanerpes hoffmannii*	2.4	1	HBW7
Piciformes	Picidae	*Sphyrapicus varius*	5.3	1	HBW7
Piciformes	Picidae	*Sphyrapicus nuchalis*	4.9	1	HBW7
Piciformes	Picidae	*Sphyrapicus ruber*	5.3	1	HBW7
Piciformes	Picidae	*Sphyrapicus thyroideus*	4.9	1	HBW7
Piciformes	Picidae	*Xiphidiopicus percussus*	3.5	1	HBW7
Piciformes	Picidae	*Campethera punctuligera*	2.4	1	HBW7
Piciformes	Picidae	*Campethera nubica*	2.4	1	HBW7
Piciformes	Picidae	*Campethera bennettii*	3.0	1	HBW7
Piciformes	Picidae	*Campethera abingoni*	2.4	1	HBW7
Piciformes	Picidae	*Campethera notata*	2.8	1	HBW7
Piciformes	Picidae	*Campethera cailliautii*	2.4	1	HBW7
Piciformes	Picidae	*Campethera nivosa*	2.0	1	HBW7
Piciformes	Picidae	*Campethera caroli*	2.4	1	HBW7
Piciformes	Picidae	*Geocolaptes olivaceus*	3.0	1	HBW7
Piciformes	Picidae	*Dendropicos fuscescens*	2.4	1	HBW7
Piciformes	Picidae	*Dendropicos stierlingi*	2.0	1	HBW7
Piciformes	Picidae	*Dendropicos namaquus*	3.0	1	HBW7
Piciformes	Picidae	*Dendropicos goertae*	2.8	1	HBW7
Piciformes	Picidae	*Dendropicos griseocephalus*	2.4	1	HBW7
Piciformes	Picidae	*Dendropicos obsoletus*	2.0	1	HBW7
Piciformes	Picidae	*Dendrocopos moluccensis*	2.8	1	HBW7
Piciformes	Picidae	*Dendrocopos canicapillus*	4.9	1	HBW7
Piciformes	Picidae	*Dendrocopos kizuki*	5.9	1	HBW7
Piciformes	Picidae	*Dendrocopos minor*	5.5	1	HBW7
Piciformes	Picidae	*Dendrocopos auriceps*	3.9	1	HBW7
Piciformes	Picidae	*Dendrocopos macei*	3.2	1	HBW7
Piciformes	Picidae	*Dendrocopos atratus*	4.5	1	HBW7
Piciformes	Picidae	*Dendrocopos hyperythrus*	4.5	1	HBW7
Piciformes	Picidae	*Dendrocopos cathpharius*	2.8	1	HBW7
Piciformes	Picidae	*Dendrocopos darjellensis*	2.8	1	HBW7
Piciformes	Picidae	*Dendrocopos medius*	5.5	1	HBW7
Piciformes	Picidae	*Dendrocopos leucotos*	3.9	1	HBW7
Piciformes	Picidae	*Dendrocopos major*	5.9	1	HBW7
Piciformes	Picidae	*Dendrocopos syriacus*	4.5	1	HBW7
Piciformes	Picidae	*Dendrocopos leucopterus*	4.9	1	HBW7
Piciformes	Picidae	*Dendrocopos assimilis*	3.5	1	HBW7
Piciformes	Picidae	*Dendrocopos himalayensis*	3.9	1	HBW7
Piciformes	Picidae	*Picoides lignarius*	3.9	1	HBW7
Piciformes	Picidae	*Picoides mixtus*	4.0	1	HBW7
Piciformes	Picidae	*Picoides nuttallii*	4.5	1	HBW7
Piciformes	Picidae	*Picoides scalaris*	3.9	1	HBW7

Order	Family	*Species*	Clutch	Range	Traits
Piciformes	Picidae	*Picoides pubescens*	4.5	1	HBW7
Piciformes	Picidae	*Picoides borealis*	3.2	1	HBW7
Piciformes	Picidae	*Picoides stricklandi*	2.8	1	HBW7
Piciformes	Picidae	*Picoides villosus*	3.2	1	HBW7
Piciformes	Picidae	*Picoides albolarvatus*	4.5	1	HBW7
Piciformes	Picidae	*Picoides tridactylus*	4.2	1	HBW7
Piciformes	Picidae	*Picoides arcticus*	3.5	1	HBW7
Piciformes	Picidae	*Veniliornis fumigatus*	4.0	1	HBW7
Piciformes	Picidae	*Veniliornis sanguineus*	1.4	1	HBW7
Piciformes	Picidae	*Veniliornis kirkii*	2.4	1	HBW7
Piciformes	Picidae	*Piculus simplex*	2.8	1	HBW7
Piciformes	Picidae	*Piculus rubiginosus*	2.8	1	HBW7
Piciformes	Picidae	*Colaptes melanochloros*	4.0	1	HBW7
Piciformes	Picidae	*Colaptes auratus*	6.0	1	HBW7
Piciformes	Picidae	*Colaptes chrysoides*	3.9	1	HBW7
Piciformes	Picidae	*Colaptes fernandinae*	3.9	1	HBW7
Piciformes	Picidae	*Colaptes pitius*	4.9	1	HBW7
Piciformes	Picidae	*Colaptes rupicola*	2.8	1	HBW7
Piciformes	Picidae	*Colaptes campestris*	4.5	1	HBW7
Piciformes	Picidae	*Celeus brachyurus*	2.4	1	HBW7
Piciformes	Picidae	*Celeus castaneus*	3.5	1	HBW7
Piciformes	Picidae	*Celeus elegans*	3.0	1	HBW7
Piciformes	Picidae	*Celeus spectabilis*	1.0	1	HBW7
Piciformes	Picidae	*Dryocopus pileatus*	2.8	1	HBW7
Piciformes	Picidae	*Dryocopus lineatus*	2.4	1	HBW7
Piciformes	Picidae	*Dryocopus schulzi*	2.0	1	HBW7
Piciformes	Picidae	*Dryocopus javensis*	2.8	1	HBW7
Piciformes	Picidae	*Dryocopus martius*	3.9	1	HBW7
Piciformes	Picidae	*Campephilus rubricollis*	1.0	1	HBW7
Piciformes	Picidae	*Campephilus guatemalensis*	2.0	1	HBW7
Piciformes	Picidae	*Campephilus melanoleucos*	2.4	1	HBW7
Piciformes	Picidae	*Campephilus magellanicus*	2.0	1	HBW7
Piciformes	Picidae	*Picus mineaceus*	2.4	1	HBW7
Piciformes	Picidae	*Picus chlorolophus*	3.5	1	HBW7
Piciformes	Picidae	*Picus puniceus*	2.4	1	HBW7
Piciformes	Picidae	*Picus flavinucha*	2.8	1	HBW7
Piciformes	Picidae	*Picus mentalis*	2.4	1	HBW7
Piciformes	Picidae	*Picus vittatus*	3.5	1	HBW7
Piciformes	Picidae	*Picus xanthopygaeus*	3.9	1	HBW7
Piciformes	Picidae	*Picus squamatus*	4.9	1	HBW7
Piciformes	Picidae	*Picus awokera*	7.5	1	HBW7
Piciformes	Picidae	*Picus viridis*	6.3	1	HBW7
Piciformes	Picidae	*Picus erythropygius*	3.5	1	HBW7
Piciformes	Picidae	*Picus canus*	6.0	1	HBW7
Piciformes	Picidae	*Dinopium shorii*	2.4	1	HBW7
Piciformes	Picidae	*Dinopium javanense*	2.4	1	HBW7
Piciformes	Picidae	*Dinopium benghalense*	2.4	1	HBW7
Piciformes	Picidae	*Chrysocolaptes lucidus*	3.2	1	HBW7
Piciformes	Picidae	*Chrysocolaptes festivus*	1.7	1	HBW7
Piciformes	Picidae	*Blythipicus pyrrhotis*	2.4	1	HBW7
Piciformes	Picidae	*Reinwardtipicus validus*	1.4	1	HBW7
Piciformes	Picidae	*Meiglyptes tristis*	2.0	1	HBW7
Piciformes	Picidae	*Meiglyptes tukki*	2.0	1	HBW7
Piciformes	Picidae	*Hemicircus canente*	2.4	1	HBW7
Piciformes	Picidae	*Mulleripicus fulvus*	2.4	1	HBW7
Piciformes	Picidae	*Mulleripicus pulverulentus*	2.8	1	HBW7
Piciformes	Megalaimidae	*Psilopogon pyrolophus*	2.0	1	HBW7
Piciformes	Megalaimidae	*Megalaima virens*	3.5	1	HBW7
Piciformes	Megalaimidae	*Megalaima zeylanica*	2.8	1	HBW7
Piciformes	Megalaimidae	*Megalaima lineata*	2.8	1	HBW7
Piciformes	Megalaimidae	*Megalaima viridis*	2.8	1	HBW7
Piciformes	Megalaimidae	*Megalaima faiostricta*	2.0	1	HBW7
Piciformes	Megalaimidae	*Megalaima corvina*	2.0	1	HBW7

Order	Family	*Species*	Clutch	Range	Traits
Piciformes	Megalaimidae	*Megalaima chrysopogon*	2.0	1	HBW7
Piciformes	Megalaimidae	*Megalaima rafflesii*	2.0	1	HBW7
Piciformes	Megalaimidae	*Megalaima mystacophanos*	2.8	1	HBW7
Piciformes	Megalaimidae	*Megalaima javensis*	2.0	1	HBW7
Piciformes	Megalaimidae	*Megalaima flavifrons*	2.4	1	HBW7
Piciformes	Megalaimidae	*Megalaima franklinii*	3.2	1	HBW7
Piciformes	Megalaimidae	*Megalaima asiatica*	3.5	1	HBW7
Piciformes	Megalaimidae	*Megalaima armillaris*	2.0	1	HBW7
Piciformes	Megalaimidae	*Megalaima australis*	2.8	1	HBW7
Piciformes	Megalaimidae	*Megalaima rubricapilla*	2.4	1	HBW7
Piciformes	Megalaimidae	*Megalaima haemacephala*	2.8	1	HBW7
Piciformes	Megalaimidae	*Calorhamphus fuliginosus*	2.4	1	HBW7
Piciformes	Lybiidae	*Gymnobucco calvus*	3.0	1	HBW7
Piciformes	Lybiidae	*Gymnobucco bonapartei*	3.9	1	HBW7
Piciformes	Lybiidae	*Stactolaema leucotis*	3.5	1	HBW7
Piciformes	Lybiidae	*Stactolaema anchietae*	3.2	1	HBW7
Piciformes	Lybiidae	*Stactolaema whytii*	3.2	1	HBW7
Piciformes	Lybiidae	*Stactolaema olivacea*	4.2	1	HBW7
Piciformes	Lybiidae	*Pogoniulus scolopaceus*	2.8	1	HBW7
Piciformes	Lybiidae	*Pogoniulus coryphaeus*	3.0	1	HBW7
Piciformes	Lybiidae	*Pogoniulus leucomystax*	2.4	1	HBW7
Piciformes	Lybiidae	*Pogoniulus simplex*	2.4	1	HBW7
Piciformes	Lybiidae	*Pogoniulus atroflavus*	2.4	1	HBW7
Piciformes	Lybiidae	*Pogoniulus subsulphureus*	2.4	1	HBW7
Piciformes	Lybiidae	*Pogoniulus bilineatus*	3.0	1	HBW7
Piciformes	Lybiidae	*Pogoniulus chrysoconus*	2.4	1	HBW7
Piciformes	Lybiidae	*Pogoniulus pusillus*	3.0	1	HBW7
Piciformes	Lybiidae	*Tricholaema diademata*	2.8	1	HBW7
Piciformes	Lybiidae	*Tricholaema frontata*	2.4	1	HBW7
Piciformes	Lybiidae	*Tricholaema leucomelas*	2.0	1	HBW7
Piciformes	Lybiidae	*Tricholaema lacrymosa*	2.8	1	HBW7
Piciformes	Lybiidae	*Tricholaema melanocephala*	2.4	1	HBW7
Piciformes	Lybiidae	*Lybius vieilloti*	2.4	1	HBW7
Piciformes	Lybiidae	*Lybius leucocephalus*	2.8	1	HBW7
Piciformes	Lybiidae	*Lybius chaplini*	2.8	1	HBW7
Piciformes	Lybiidae	*Lybius guifsobalito*	3.0	1	HBW7
Piciformes	Lybiidae	*Lybius torquatus*	2.2	1	HBW7
Piciformes	Lybiidae	*Lybius melanopterus*	3.5	1	HBW7
Piciformes	Lybiidae	*Lybius minor*	3.5	1	HBW7
Piciformes	Lybiidae	*Lybius bidentatus*	2.8	1	HBW7
Piciformes	Lybiidae	*Lybius dubius*	2.0	1	HBW7
Piciformes	Lybiidae	*Lybius rolleti*	1.4	1	HBW7
Piciformes	Lybiidae	*Trachyphonus purpuratus*	2.8	1	HBW7
Piciformes	Lybiidae	*Trachyphonus vaillantii*	3.2	1	HBW7
Piciformes	Lybiidae	*Trachyphonus margaritatus*	4.2	1	HBW7
Piciformes	Lybiidae	*Trachyphonus erythrocephalus*	3.5	1	HBW7
Piciformes	Lybiidae	*Trachyphonus darnaudii*	2.8	1	HBW7
Piciformes	Ramphastidae	*Capito squamatus*	4.0	1	HBW7
Piciformes	Ramphastidae	*Capito niger*	3.5	1	HBW7
Piciformes	Ramphastidae	*Eubucco bourcierii*	3.2	1	HBW7
Piciformes	Ramphastidae	*Semnornis frantzii*	4.5	1	HBW7
Piciformes	Ramphastidae	*Semnornis ramphastinus*	2.4	1	HBW7
Piciformes	Ramphastidae	*Aulacorhynchus prasinus*	2.2	1	HBW7
Piciformes	Ramphastidae	*Aulacorhynchus haematopygus*	4.0	1	HBW7
Piciformes	Ramphastidae	*Pteroglossus viridis*	2.8	1	HBW7
Piciformes	Ramphastidae	*Pteroglossus azara*	2.8	1	HBW7
Piciformes	Ramphastidae	*Pteroglossus castanotis*	2.0	1	HBW7
Piciformes	Ramphastidae	*Pteroglossus aracari*	2.8	1	HBW7
Piciformes	Ramphastidae	*Pteroglossus torquatus*	3.2	1	HBW7
Piciformes	Ramphastidae	*Pteroglossus frantzii*	2.0	1	HBW7
Piciformes	Ramphastidae	*Andigena laminirostris*	2.4	1	HBW7
Piciformes	Ramphastidae	*Selenidera spectabilis*	2.8	1	HBW7
Piciformes	Ramphastidae	*Selenidera culik*	2.4	1	HBW7

Order	Family	*Species*	Clutch	Range	Traits
Piciformes	Ramphastidae	*Selenidera maculirostris*	2.4	1	HBW7
Piciformes	Ramphastidae	*Ramphastos sulfuratus*	2.0	1	HBW7
Piciformes	Ramphastidae	*Ramphastos vitellinus*	2.8	1	HBW7
Piciformes	Ramphastidae	*Ramphastos dicolorus*	2.8	1	HBW7
Piciformes	Ramphastidae	*Ramphastos ambiguus*	2.4	1	HBW7
Piciformes	Ramphastidae	*Ramphastos tucanus*	2.4	1	HBW7
Piciformes	Ramphastidae	*Ramphastos toco*	2.8	1	HBW7
Galbuliformes	Galbulidae	*Brachygalba goeringi*	4.0	1	HBW7
Galbuliformes	Galbulidae	*Brachygalba lugubris*	3.0	1	HBW7
Galbuliformes	Galbulidae	*Galbula albirostris*	2.0	1	HBW7
Galbuliformes	Galbulidae	*Galbula cyanicollis*	2.0	1	HBW7
Galbuliformes	Galbulidae	*Galbula ruficauda*	2.8	1	HBW7
Galbuliformes	Galbulidae	*Galbula leucogastra*	1.0	1	HBW7
Galbuliformes	Bucconidae	*Notharchus pectoralis*	3.0	1	HBW7
Galbuliformes	Bucconidae	*Notharchus tectus*	2.0	1	HBW7
Galbuliformes	Bucconidae	*Bucco tamatia*	2.0	1	HBW7
Galbuliformes	Bucconidae	*Nystalus chacuru*	2.8	1	HBW7
Galbuliformes	Bucconidae	*Nystalus striolatus*	4.0	1	HBW7
Galbuliformes	Bucconidae	*Nystalus maculatus*	2.4	1	HBW7
Galbuliformes	Bucconidae	*Hypnelus ruficollis*	3.0	1	HBW7
Galbuliformes	Bucconidae	*Malacoptila panamensis*	2.4	1	HBW7
Galbuliformes	Bucconidae	*Micromonacha lanceolata*	2.0	1	HBW7
Galbuliformes	Bucconidae	*Hapaloptila castanea*	2.0	1	HBW7
Galbuliformes	Bucconidae	*Monasa nigrifrons*	3.0	1	HBW7
Galbuliformes	Bucconidae	*Monasa morphoeus*	2.4	1	HBW7
Galbuliformes	Bucconidae	*Chelidoptera tenebrosa*	1.4	1	HBW7
Bucerotiformes	Bucerotidae	*Tockus hartlaubi*	4.0	1	HBW6
Bucerotiformes	Bucerotidae	*Tockus monteiri*	4.0	1	HBW6
Bucerotiformes	Bucerotidae	*Tockus erythrorhynchus*	3.7	1	HBW6
Bucerotiformes	Bucerotidae	*Tockus flavirostris*	2.4	1	HBW6
Bucerotiformes	Bucerotidae	*Tockus leucomelas*	3.5	1	HBW6
Bucerotiformes	Bucerotidae	*Tockus deckeni*	2.8	1	HBW6
Bucerotiformes	Bucerotidae	*Tockus alboterminatus*	3.2	1	HBW6
Bucerotiformes	Bucerotidae	*Tockus fasciatus*	4.0	1	HBW6
Bucerotiformes	Bucerotidae	*Tockus hemprichii*	3.0	1	HBW6
Bucerotiformes	Bucerotidae	*Tockus nasutus*	3.2	1	HBW6
Bucerotiformes	Bucerotidae	*Tockus pallidirostris*	4.5	1	HBW6
Bucerotiformes	Bucerotidae	*Anthracoceros coronatus*	2.8	1	HBW6
Bucerotiformes	Bucerotidae	*Anthracoceros albirostris*	1.7	1	HBW6
Bucerotiformes	Bucerotidae	*Anthracoceros malayanus*	2.4	1	HBW6
Bucerotiformes	Bucerotidae	*Buceros rhinoceros*	1.4	1	HBW6
Bucerotiformes	Bucerotidae	*Buceros bicornis*	2.0	1	HBW6
Bucerotiformes	Bucerotidae	*Buceros hydrocorax*	2.8	1	HBW6
Bucerotiformes	Bucerotidae	*Buceros vigil*	1.4	1	HBW6
Bucerotiformes	Bucerotidae	*Anorrhinus tickelli*	3.2	1	HBW6
Bucerotiformes	Bucerotidae	*Anorrhinus galeritus*	2.4	1	HBW6
Bucerotiformes	Bucerotidae	*Penelopides manillae*	4.2	1	HBW6
Bucerotiformes	Bucerotidae	*Penelopides panini*	2.4	1	HBW6
Bucerotiformes	Bucerotidae	*Penelopides exarhatus*	2.8	1	HBW6
Bucerotiformes	Bucerotidae	*Aceros nipalensis*	1.4	1	HBW6
Bucerotiformes	Bucerotidae	*Aceros corrugatus*	2.4	1	HBW6
Bucerotiformes	Bucerotidae	*Aceros waldeni*	2.4	1	HBW6
Bucerotiformes	Bucerotidae	*Aceros cassidix*	2.4	1	HBW6
Bucerotiformes	Bucerotidae	*Aceros undulatus*	1.7	1	HBW6
Bucerotiformes	Bucerotidae	*Aceros subruficollis*	1.7	1	HBW6
Bucerotiformes	Bucerotidae	*Aceros plicatus*	1.4	1	HBW6
Bucerotiformes	Bucerotidae	*Ceratogymna bucinator*	2.8	1	HBW6
Bucerotiformes	Bucerotidae	*Ceratogymna fistulator*	1.7	1	HBW6
Bucerotiformes	Bucerotidae	*Ceratogymna brevis*	1.4	1	HBW6
Bucerotiformes	Bucerotidae	*Ceratogymna subcylindricus*	2.0	1	HBW6
Bucerotiformes	Bucerotidae	*Ceratogymna atrata*	1.4	1	HBW6
Bucerotiformes	Bucorvidae	*Bucorvus abyssinicus*	2.0	1	HBW6
Bucerotiformes	Bucorvidae	*Bucorvus leadbeateri*	1.7	1	HBW6

Order	Family	*Species*	Clutch	Range	Traits
Upupiformes	Upupidae	*Upupa epops*	5.7	1	HBW6
Upupiformes	Phoeniculidae	*Phoeniculus purpureus*	3.2	1	HBW6
Upupiformes	Phoeniculidae	*Phoeniculus damarensis*	3.0	1	HBW6
Upupiformes	Phoeniculidae	*Phoeniculus somaliensis*	3.9	1	HBW6
Upupiformes	Phoeniculidae	*Phoeniculus bollei*	2.0	1	HBW6
Upupiformes	Rhinopomastidae	*Rhinopomastus cyanomelas*	2.8	1	HBW6
Upupiformes	Rhinopomastidae	*Rhinopomastus minor*	2.4	1	HBW6
Trogoniformes	Trogonidae	*Apaloderma narina*	2.0	1	HBW6
Trogoniformes	Trogonidae	*Apaloderma aequatoriale*	2.0	1	HBW6
Trogoniformes	Trogonidae	*Apaloderma vittatum*	2.4	1	HBW6
Trogoniformes	Trogonidae	*Trogon massena*	3.0	1	HBW6
Trogoniformes	Trogonidae	*Trogon melanurus*	2.4	1	HBW6
Trogoniformes	Trogonidae	*Trogon comptus*	3.5	1	HBW6
Trogoniformes	Trogonidae	*Trogon bairdii*	2.4	1	HBW6
Trogoniformes	Trogonidae	*Trogon viridis*	2.4	1	HBW6
Trogoniformes	Trogonidae	*Trogon citreolus*	2.8	1	HBW6
Trogoniformes	Trogonidae	*Trogon melanocephalus*	3.0	1	HBW6
Trogoniformes	Trogonidae	*Trogon mexicanus*	2.4	1	HBW6
Trogoniformes	Trogonidae	*Trogon elegans*	2.4	1	HBW6
Trogoniformes	Trogonidae	*Trogon collaris*	2.4	1	HBW6
Trogoniformes	Trogonidae	*Trogon personatus*	2.0	1	HBW6
Trogoniformes	Trogonidae	*Trogon rufus*	2.0	1	HBW6
Trogoniformes	Trogonidae	*Trogon curucui*	2.4	1	HBW6
Trogoniformes	Trogonidae	*Trogon violaceus*	2.4	1	HBW6
Trogoniformes	Trogonidae	*Harpactes reinwardtii*	1.4	1	HBW6
Trogoniformes	Trogonidae	*Harpactes fasciatus*	2.8	1	HBW6
Trogoniformes	Trogonidae	*Harpactes oreskios*	2.4	1	HBW6
Trogoniformes	Trogonidae	*Harpactes erythrocephalus*	2.8	1	HBW6
Coraciiformes	Coraciidae	*Coracias garrulus*	4.2	1	HBW6
Coraciiformes	Coraciidae	*Coracias abyssinica*	4.2	1	HBW6
Coraciiformes	Coraciidae	*Coracias caudata*	2.8	1	HBW6
Coraciiformes	Coraciidae	*Coracias spatulata*	2.8	1	HBW6
Coraciiformes	Coraciidae	*Coracias naevia*	2.8	1	HBW6
Coraciiformes	Coraciidae	*Coracias benghalensis*	3.9	1	HBW6
Coraciiformes	Coraciidae	*Eurystomus glaucurus*	2.4	1	HBW6
Coraciiformes	Coraciidae	*Eurystomus gularis*	2.4	1	HBW6
Coraciiformes	Coraciidae	*Eurystomus orientalis*	3.9	1	HBW6
Coraciiformes	Alcedinidae	*Alcedo hercules*	4.9	1	HBW6
Coraciiformes	Alcedinidae	*Alcedo atthis*	6.5	1	HBW6
Coraciiformes	Alcedinidae	*Alcedo semitorquata*	3.5	1	HBW6
Coraciiformes	Alcedinidae	*Alcedo quadribrachys*	4.9	1	HBW6
Coraciiformes	Alcedinidae	*Alcedo meninting*	4.6	1	HBW6
Coraciiformes	Alcedinidae	*Alcedo azurea*	5.3	1	HBW6
Coraciiformes	Alcedinidae	*Alcedo euryzona*	3.9	1	HBW6
Coraciiformes	Alcedinidae	*Alcedo cristata*	4.2	1	HBW6
Coraciiformes	Alcedinidae	*Alcedo vintsioides*	4.2	1	HBW6
Coraciiformes	Alcedinidae	*Alcedo leucogaster*	2.0	1	HBW6
Coraciiformes	Alcedinidae	*Alcedo coerulescens*	4.2	1	HBW6
Coraciiformes	Alcedinidae	*Alcedo pusilla*	3.9	1	HBW6
Coraciiformes	Alcedinidae	*Ceyx lepidus*	2.0	1	HBW6
Coraciiformes	Alcedinidae	*Ceyx erithacus*	4.6	1	HBW6
Coraciiformes	Alcedinidae	*Ispidina madagascariensis*	4.0	1	HBW6
Coraciiformes	Alcedinidae	*Ispidina picta*	4.2	1	HBW6
Coraciiformes	Alcedinidae	*Ispidina lecontei*	3.0	1	HBW6
Coraciiformes	Halcyonidae	*Lacedo pulchella*	3.2	1	HBW6
Coraciiformes	Halcyonidae	*Dacelo novaeguineae*	2.2	1	HBW6
Coraciiformes	Halcyonidae	*Dacelo leachii*	2.4	1	HBW6
Coraciiformes	Halcyonidae	*Dacelo gaudichaud*	2.0	1	HBW6
Coraciiformes	Halcyonidae	*Pelargopsis amauropterus*	3.5	1	HBW6
Coraciiformes	Halcyonidae	*Pelargopsis capensis*	3.2	1	HBW6
Coraciiformes	Halcyonidae	*Halcyon coromanda*	4.9	1	HBW6
Coraciiformes	Halcyonidae	*Halcyon badia*	2.4	1	HBW6
Coraciiformes	Halcyonidae	*Halcyon smyrnensis*	5.3	1	HBW6

Order	Family	*Species*	Clutch	Range	Traits
Coraciiformes	Halcyonidae	*Halcyon pileata*	4.5	1	HBW6
Coraciiformes	Halcyonidae	*Halcyon cyanoventris*	3.9	1	HBW6
Coraciiformes	Halcyonidae	*Halcyon leucocephala*	3.5	1	HBW6
Coraciiformes	Halcyonidae	*Halcyon senegalensis*	2.8	1	HBW6
Coraciiformes	Halcyonidae	*Halcyon senegaloides*	3.0	1	HBW6
Coraciiformes	Halcyonidae	*Halcyon malimbica*	2.8	1	HBW6
Coraciiformes	Halcyonidae	*Halcyon albiventris*	3.2	1	HBW6
Coraciiformes	Halcyonidae	*Halcyon chelicuti*	3.9	1	HBW6
Coraciiformes	Halcyonidae	*Todiramphus macleayii*	4.2	1	HBW6
Coraciiformes	Halcyonidae	*Todiramphus albonotatus*	2.4	1	HBW6
Coraciiformes	Halcyonidae	*Todiramphus leucopygius*	3.0	1	HBW6
Coraciiformes	Halcyonidae	*Todiramphus farquhari*	3.0	1	HBW6
Coraciiformes	Halcyonidae	*Todiramphus pyrrhopygia*	4.5	1	HBW6
Coraciiformes	Halcyonidae	*Todiramphus chloris*	3.2	1	HBW6
Coraciiformes	Halcyonidae	*Todiramphus saurophaga*	2.8	1	HBW6
Coraciiformes	Halcyonidae	*Todiramphus sanctus*	4.5	1	HBW6
Coraciiformes	Halcyonidae	*Actenoides concretus*	2.0	1	HBW6
Coraciiformes	Cerylidae	*Megaceryle maxima*	3.9	1	HBW6
Coraciiformes	Cerylidae	*Megaceryle lugubris*	4.5	1	HBW6
Coraciiformes	Cerylidae	*Megaceryle alcyon*	6.5	1	HBW6
Coraciiformes	Cerylidae	*Megaceryle torquata*	4.0	1	HBW6
Coraciiformes	Cerylidae	*Ceryle rudis*	4.5	1	HBW6
Coraciiformes	Cerylidae	*Chloroceryle amazona*	3.5	1	HBW6
Coraciiformes	Cerylidae	*Chloroceryle americana*	4.5	1	HBW6
Coraciiformes	Cerylidae	*Chloroceryle inda*	3.9	1	HBW6
Coraciiformes	Cerylidae	*Chloroceryle aenea*	3.5	1	HBW6
Coraciiformes	Nyctyornithidae	*Nyctyornis amictus*	3.9	1	HBW6
Coraciiformes	Meropidae	*Merops gularis*	2.0	1	HBW6
Coraciiformes	Meropidae	*Merops muelleri*	2.0	1	HBW6
Coraciiformes	Meropidae	*Merops bulocki*	2.8	1	HBW6
Coraciiformes	Meropidae	*Merops bullockoides*	3.2	1	HBW6
Coraciiformes	Meropidae	*Merops pusillus*	4.9	1	HBW6
Coraciiformes	Meropidae	*Merops variegatus*	2.4	1	HBW6
Coraciiformes	Meropidae	*Merops oreobates*	2.4	1	HBW6
Coraciiformes	Meropidae	*Merops hirundineus*	2.8	1	HBW6
Coraciiformes	Meropidae	*Merops breweri*	2.4	1	HBW6
Coraciiformes	Meropidae	*Merops albicollis*	5.3	1	HBW6
Coraciiformes	Meropidae	*Merops orientalis*	5.7	1	HBW6
Coraciiformes	Meropidae	*Merops boehmi*	2.8	1	HBW6
Coraciiformes	Meropidae	*Merops viridis*	4.2	1	HBW6
Coraciiformes	Meropidae	*Merops persicus*	5.7	1	HBW6
Coraciiformes	Meropidae	*Merops superciliosus*	4.9	1	HBW6
Coraciiformes	Meropidae	*Merops philippinus*	5.9	1	HBW6
Coraciiformes	Meropidae	*Merops ornatus*	4.6	1	HBW6
Coraciiformes	Meropidae	*Merops apiaster*	6.3	1	HBW6
Coraciiformes	Meropidae	*Merops leschenaulti*	5.7	1	HBW6
Coraciiformes	Meropidae	*Merops malimbicus*	2.0	1	HBW6
Coraciiformes	Meropidae	*Merops nubicus*	3.2	1	HBW6
Coliiformes	Coliidae	*Colius striatus*	2.4	1	HBW6
Coliiformes	Coliidae	*Colius leucocephalus*	3.0	1	HBW6
Coliiformes	Coliidae	*Colius castanotus*	3.0	1	HBW6
Coliiformes	Coliidae	*Colius colius*	3.0	1	HBW6
Coliiformes	Coliidae	*Urocolius macrourus*	2.4	1	HBW6
Coliiformes	Coliidae	*Urocolius indicus*	3.5	1	HBW6
Cuculiformes	Cuculidae	*Ceuthmochares aereus*	2.4	1	HBW4
Cuculiformes	Cuculidae	*Phaenicophaeus sumatranus*	2.0	1	HBW4
Cuculiformes	Cuculidae	*Phaenicophaeus tristis*	3.0	1	HBW4
Cuculiformes	Cuculidae	*Phaenicophaeus viridirostris*	2.0	1	HBW4
Cuculiformes	Cuculidae	*Phaenicophaeus leschenaultii*	2.4	1	HBW4
Cuculiformes	Cuculidae	*Phaenicophaeus chlorophaeus*	2.4	1	HBW4
Cuculiformes	Cuculidae	*Phaenicophaeus curvirostris*	2.4	1	HBW4
Cuculiformes	Cuculidae	*Phaenicophaeus pyrrhocephal*	2.4	1	HBW4
Cuculiformes	Cuculidae	*Carpococcyx renauldi*	2.8	1	HBW4

Order	Family	*Species*	Clutch	Range	Traits
Cuculiformes	Cuculidae	*Coua gigas*	3.0	1	HBW4
Cuculiformes	Cuculidae	*Coua coquereli*	2.0	1	HBW4
Cuculiformes	Cuculidae	*Coua serriana*	2.0	1	HBW4
Cuculiformes	Cuculidae	*Coua reynaudii*	2.0	1	HBW4
Cuculiformes	Cuculidae	*Coua cursor*	2.0	1	HBW4
Cuculiformes	Cuculidae	*Coua ruficeps*	2.0	1	HBW4
Cuculiformes	Cuculidae	*Coua cristata*	2.0	1	HBW4
Cuculiformes	Cuculidae	*Coua caerulea*	1.0	1	HBW4
Cuculiformes	Centropodidae	*Centropus violaceus*	3.0	1	HBW4
Cuculiformes	Centropodidae	*Centropus ateralbus*	2.0	1	HBW4
Cuculiformes	Centropodidae	*Centropus phasianinus*	3.9	1	HBW4
Cuculiformes	Centropodidae	*Centropus bernsteini*	2.0	1	HBW4
Cuculiformes	Centropodidae	*Centropus sinensis*	2.8	1	HBW4
Cuculiformes	Centropodidae	*Centropus andamanensis*	2.4	1	HBW4
Cuculiformes	Centropodidae	*Centropus nigrorufus*	1.7	1	HBW4
Cuculiformes	Centropodidae	*Centropus viridis*	3.0	1	HBW4
Cuculiformes	Centropodidae	*Centropus toulou*	2.4	1	HBW4
Cuculiformes	Centropodidae	*Centropus grillii*	4.2	1	HBW4
Cuculiformes	Centropodidae	*Centropus bengalensis*	2.8	1	HBW4
Cuculiformes	Centropodidae	*Centropus chlororhynchus*	2.4	1	HBW4
Cuculiformes	Centropodidae	*Centropus leucogaster*	2.0	1	HBW4
Cuculiformes	Centropodidae	*Centropus monachus*	3.0	1	HBW4
Cuculiformes	Centropodidae	*Centropus cupreicaudus*	2.8	1	HBW4
Cuculiformes	Centropodidae	*Centropus senegalensis*	3.2	1	HBW4
Cuculiformes	Centropodidae	*Centropus superciliosus*	3.9	1	HBW4
Cuculiformes	Coccyzidae	*Hyetornis pluvialis*	2.8	1	HBW4
Cuculiformes	Coccyzidae	*Hyetornis rufigularis*	2.0	1	HBW4
Cuculiformes	Coccyzidae	*Piaya cayana*	2.4	1	HBW4
Cuculiformes	Coccyzidae	*Piaya minuta*	2.0	1	HBW4
Cuculiformes	Coccyzidae	*Saurothera merlini*	2.4	1	HBW4
Cuculiformes	Coccyzidae	*Saurothera longirostris*	2.4	1	HBW4
Cuculiformes	Coccyzidae	*Saurothera vieilloti*	2.4	1	HBW4
Cuculiformes	Crotophagidae	*Crotophaga major*	2.4	1	HBW4
Cuculiformes	Crotophagidae	*Crotophaga ani*	2.9	1	HBW4
Cuculiformes	Crotophagidae	*Crotophaga sulcirostris*	3.5	1	HBW4
Cuculiformes	Neomorphidae	*Morococcyx erythropygus*	2.0	1	HBW4
Cuculiformes	Neomorphidae	*Geococcyx californianus*	3.5	1	HBW4
Cuculiformes	Neomorphidae	*Geococcyx velox*	2.4	1	HBW4
Cuculiformes	Neomorphidae	*Neomorphus geoffroyi*	1.0	1	HBW4
Psittaciformes	Cacatuidae	*Probosciger aterrimus*	1.0	1	HBW4
Psittaciformes	Cacatuidae	*Calyptorhynchus baudinii*	2.0	1	HBW4
Psittaciformes	Cacatuidae	*Calyptorhynchus latirostris*	1.4	1	HBW4
Psittaciformes	Cacatuidae	*Calyptorhynchus funereus*	2.0	1	HBW4
Psittaciformes	Cacatuidae	*Calyptorhynchus banksii*	1.0	1	HBW4
Psittaciformes	Cacatuidae	*Calyptorhynchus lathami*	1.0	1	HBW4
Psittaciformes	Cacatuidae	*Callocephalon fimbriatum*	2.0	1	HBW4
Psittaciformes	Cacatuidae	*Cacatua roseicapilla*	3.5	1	HBW4
Psittaciformes	Cacatuidae	*Cacatua leadbeateri*	3.2	1	HBW4
Psittaciformes	Cacatuidae	*Cacatua sulphurea*	2.4	1	HBW4
Psittaciformes	Cacatuidae	*Cacatua galerita*	2.4	1	HBW4
Psittaciformes	Cacatuidae	*Cacatua ophthalmica*	2.0	1	HBW4
Psittaciformes	Cacatuidae	*Cacatua moluccensis*	2.0	1	HBW4
Psittaciformes	Cacatuidae	*Cacatua alba*	2.4	1	HBW4
Psittaciformes	Cacatuidae	*Cacatua haematuropygia*	2.4	1	HBW4
Psittaciformes	Cacatuidae	*Cacatua sanguinea*	2.4	1	HBW4
Psittaciformes	Cacatuidae	*Cacatua pastinator*	2.4	1	HBW4
Psittaciformes	Cacatuidae	*Cacatua tenuirostris*	2.4	1	HBW4
Psittaciformes	Cacatuidae	*Nymphicus hollandicus*	4.6	1	HBW4
Psittaciformes	Psittacidae	*Eos squamata*	2.0	1	HBW4
Psittaciformes	Psittacidae	*Eos reticulata*	2.0	1	HBW4
Psittaciformes	Psittacidae	*Eos cyanogenia*	2.0	1	HBW4
Psittaciformes	Psittacidae	*Pseudeos fuscata*	2.0	1	HBW4
Psittaciformes	Psittacidae	*Trichoglossus ornatus*	2.0	1	HBW4

Order	Family	*Species*	Clutch	Range	Traits
Psittaciformes	Psittacidae	*Trichoglossus haematodus*	1.7	1	HBW4
Psittaciformes	Psittacidae	*Trichoglossus euteles*	3.0	1	HBW4
Psittaciformes	Psittacidae	*Trichoglossus flavoviridis*	2.0	1	HBW4
Psittaciformes	Psittacidae	*Trichoglossus johnstoniae*	2.0	1	HBW4
Psittaciformes	Psittacidae	*Trichoglossus chlorolepidotus*	2.0	1	HBW4
Psittaciformes	Psittacidae	*Psitteuteles versicolor*	3.2	1	HBW4
Psittaciformes	Psittacidae	*Psitteuteles iris*	2.0	1	HBW4
Psittaciformes	Psittacidae	*Psitteuteles goldiei*	2.0	1	HBW4
Psittaciformes	Psittacidae	*Phigys solitarius*	2.0	1	HBW4
Psittaciformes	Psittacidae	*Glossopsitta concinna*	2.0	1	HBW4
Psittaciformes	Psittacidae	*Glossopsitta pusilla*	3.9	1	HBW4
Psittaciformes	Psittacidae	*Glossopsitta porphyrocephala*	3.5	1	HBW4
Psittaciformes	Psittacidae	*Neopsittacus musschenbroekii*	2.0	1	HBW4
Psittaciformes	Psittacidae	*Neopsittacus pullicauda*	2.0	1	HBW4
Psittaciformes	Psittacidae	*Cyclopsitta diophthalma*	2.0	1	HBW4
Psittaciformes	Psittacidae	*Psittinus cyanurus*	3.0	1	HBW4
Psittaciformes	Psittacidae	*Prioniturus discurus*	3.0	1	HBW4
Psittaciformes	Psittacidae	*Eclectus roratus*	2.0	1	HBW4
Psittaciformes	Psittacidae	*Alisterus scapularis*	4.2	1	HBW4
Psittaciformes	Psittacidae	*Alisterus amboinensis*	2.0	1	HBW4
Psittaciformes	Psittacidae	*Aprosmictus erythropterus*	3.9	1	HBW4
Psittaciformes	Psittacidae	*Polytelis swainsonii*	4.9	1	HBW4
Psittaciformes	Psittacidae	*Polytelis anthopeplus*	4.9	1	HBW4
Psittaciformes	Psittacidae	*Polytelis alexandrae*	4.9	1	HBW4
Psittaciformes	Psittacidae	*Purpureicephalus spurius*	5.3	1	HBW4
Psittaciformes	Psittacidae	*Barnardius zonarius*	5.3	1	HBW4
Psittaciformes	Psittacidae	*Platycercus caledonicus*	4.9	1	HBW4
Psittaciformes	Psittacidae	*Platycercus elegans*	5.3	1	HBW4
Psittaciformes	Psittacidae	*Platycercus venustus*	2.8	1	HBW4
Psittaciformes	Psittacidae	*Platycercus adscitus*	3.9	1	HBW4
Psittaciformes	Psittacidae	*Platycercus eximius*	6.0	1	HBW4
Psittaciformes	Psittacidae	*Platycercus icterotis*	4.6	1	HBW4
Psittaciformes	Psittacidae	*Northiella haematogaster*	5.3	1	HBW4
Psittaciformes	Psittacidae	*Psephotus haematonotus*	5.7	1	HBW4
Psittaciformes	Psittacidae	*Psephotus varius*	5.3	1	HBW4
Psittaciformes	Psittacidae	*Psephotus dissimilis*	4.0	1	HBW4
Psittaciformes	Psittacidae	*Psephotus chrysopterygius*	4.2	1	HBW4
Psittaciformes	Psittacidae	*Neopsephotus bourkii*	4.2	1	HBW4
Psittaciformes	Psittacidae	*Neophema chrysostoma*	4.9	1	HBW4
Psittaciformes	Psittacidae	*Neophema elegans*	4.5	1	HBW4
Psittaciformes	Psittacidae	*Neophema petrophila*	4.5	1	HBW4
Psittaciformes	Psittacidae	*Neophema chrysogaster*	4.2	1	HBW4
Psittaciformes	Psittacidae	*Neophema pulchella*	4.5	1	HBW4
Psittaciformes	Psittacidae	*Neophema splendida*	3.9	1	HBW4
Psittaciformes	Psittacidae	*Lathamus discolor*	3.9	1	HBW4
Psittaciformes	Psittacidae	*Melopsittacus undulatus*	4.9	1	HBW4
Psittaciformes	Psittacidae	*Pezoporus wallicus*	3.5	1	HBW4
Psittaciformes	Psittacidae	*Pezoporus occidentalis*	4.0	1	HBW4
Psittaciformes	Psittacidae	*Coracopsis vasa*	3.0	1	HBW4
Psittaciformes	Psittacidae	*Coracopsis nigra*	2.4	1	HBW4
Psittaciformes	Psittacidae	*Psittacus erithacus*	2.4	1	HBW4
Psittaciformes	Psittacidae	*Poicephalus robustus*	4.0	1	HBW4
Psittaciformes	Psittacidae	*Poicephalus gulielmi*	2.8	1	HBW4
Psittaciformes	Psittacidae	*Poicephalus senegalus*	2.8	1	HBW4
Psittaciformes	Psittacidae	*Poicephalus meyeri*	2.8	1	HBW4
Psittaciformes	Psittacidae	*Poicephalus cryptoxanthus*	2.4	1	HBW4
Psittaciformes	Psittacidae	*Poicephalus rueppellii*	3.9	1	HBW4
Psittaciformes	Psittacidae	*Agapornis canus*	4.2	1	HBW4
Psittaciformes	Psittacidae	*Agapornis pullarius*	3.9	1	HBW4
Psittaciformes	Psittacidae	*Agapornis taranta*	5.0	1	HBW4
Psittaciformes	Psittacidae	*Agapornis roseicollis*	4.9	1	HBW4
Psittaciformes	Psittacidae	*Agapornis fischeri*	4.9	1	HBW4
Psittaciformes	Psittacidae	*Agapornis personatus*	4.9	1	HBW4

Order	Family	*Species*	Clutch	Range	Traits
Psittaciformes	Psittacidae	*Agapornis lilianae*	4.9	1	HBW4
Psittaciformes	Psittacidae	*Agapornis nigrigenis*	4.9	1	HBW4
Psittaciformes	Psittacidae	*Loriculus vernalis*	2.8	1	HBW4
Psittaciformes	Psittacidae	*Loriculus beryllinus*	2.4	1	HBW4
Psittaciformes	Psittacidae	*Loriculus philippensis*	3.0	1	HBW4
Psittaciformes	Psittacidae	*Loriculus galgulus*	3.0	1	HBW4
Psittaciformes	Psittacidae	*Loriculus aurantiifrons*	4.0	1	HBW4
Psittaciformes	Psittacidae	*Loriculus pusillus*	2.0	1	HBW4
Psittaciformes	Psittacidae	*Psittacula eupatria*	3.5	1	HBW4
Psittaciformes	Psittacidae	*Psittacula krameri*	3.5	1	HBW4
Psittaciformes	Psittacidae	*Psittacula echo*	2.8	1	HBW4
Psittaciformes	Psittacidae	*Psittacula himalayana*	3.9	1	HBW4
Psittaciformes	Psittacidae	*Psittacula finschii*	4.0	1	HBW4
Psittaciformes	Psittacidae	*Psittacula cyanocephala*	4.5	1	HBW4
Psittaciformes	Psittacidae	*Psittacula columboides*	4.5	1	HBW4
Psittaciformes	Psittacidae	*Psittacula calthropae*	2.8	1	HBW4
Psittaciformes	Psittacidae	*Psittacula alexandri*	3.5	1	HBW4
Psittaciformes	Psittacidae	*Psittacula longicauda*	2.4	1	HBW4
Psittaciformes	Psittacidae	*Anodorhynchus hyacinthinus*	2.4	1	HBW4
Psittaciformes	Psittacidae	*Anodorhynchus leari*	2.0	1	HBW4
Psittaciformes	Psittacidae	*Cyanopsitta spixii*	2.4	1	HBW4
Psittaciformes	Psittacidae	*Ara ararauna*	1.7	1	HBW4
Psittaciformes	Psittacidae	*Ara glaucogularis*	2.4	1	HBW4
Psittaciformes	Psittacidae	*Ara militaris*	2.4	1	HBW4
Psittaciformes	Psittacidae	*Ara ambigua*	2.4	1	HBW4
Psittaciformes	Psittacidae	*Ara macao*	2.0	1	HBW4
Psittaciformes	Psittacidae	*Ara chloropterus*	2.4	1	HBW4
Psittaciformes	Psittacidae	*Ara rubrogenys*	1.7	1	HBW4
Psittaciformes	Psittacidae	*Ara severa*	2.0	1	HBW4
Psittaciformes	Psittacidae	*Ara manilata*	2.0	1	HBW4
Psittaciformes	Psittacidae	*Ara maracana*	2.0	1	HBW4
Psittaciformes	Psittacidae	*Ara auricollis*	3.5	1	HBW4
Psittaciformes	Psittacidae	*Ara nobilis*	4.0	1	HBW4
Psittaciformes	Psittacidae	*Aratinga acuticaudata*	2.0	1	HBW4
Psittaciformes	Psittacidae	*Aratinga guarouba*	2.4	1	HBW4
Psittaciformes	Psittacidae	*Aratinga holochlora*	4.0	1	HBW4
Psittaciformes	Psittacidae	*Aratinga mitrata*	2.4	1	HBW4
Psittaciformes	Psittacidae	*Aratinga erythrogenys*	2.8	1	HBW4
Psittaciformes	Psittacidae	*Aratinga finschi*	2.8	1	HBW4
Psittaciformes	Psittacidae	*Aratinga leucophthalmus*	3.5	1	HBW4
Psittaciformes	Psittacidae	*Aratinga euops*	3.2	1	HBW4
Psittaciformes	Psittacidae	*Aratinga solstitialis*	4.0	1	HBW4
Psittaciformes	Psittacidae	*Aratinga jandaya*	3.0	1	HBW4
Psittaciformes	Psittacidae	*Aratinga weddellii*	3.5	1	HBW4
Psittaciformes	Psittacidae	*Aratinga nana*	3.9	1	HBW4
Psittaciformes	Psittacidae	*Aratinga canicularis*	3.9	1	HBW4
Psittaciformes	Psittacidae	*Aratinga aurea*	2.8	1	HBW4
Psittaciformes	Psittacidae	*Aratinga pertinax*	3.7	1	HBW4
Psittaciformes	Psittacidae	*Aratinga cactorum*	6.0	1	HBW4
Psittaciformes	Psittacidae	*Nandayus nenday*	4.0	1	HBW4
Psittaciformes	Psittacidae	*Ognorhynchus icterotis*	4.0	1	HBW4
Psittaciformes	Psittacidae	*Rhynchopsitta pachyrhyncha*	2.0	1	HBW4
Psittaciformes	Psittacidae	*Cyanoliseus patagonus*	2.8	1	HBW4
Psittaciformes	Psittacidae	*Pyrrhura cruentata*	2.8	1	HBW4
Psittaciformes	Psittacidae	*Pyrrhura frontalis*	5.0	1	HBW4
Psittaciformes	Psittacidae	*Pyrrhura perlata*	4.2	1	HBW4
Psittaciformes	Psittacidae	*Pyrrhura molinae*	3.0	1	HBW4
Psittaciformes	Psittacidae	*Pyrrhura picta*	4.5	1	HBW4
Psittaciformes	Psittacidae	*Pyrrhura leucotis*	6.7	1	HBW4
Psittaciformes	Psittacidae	*Pyrrhura melanura*	4.0	1	HBW4
Psittaciformes	Psittacidae	*Pyrrhura rupicola*	7.0	1	HBW4
Psittaciformes	Psittacidae	*Pyrrhura hoffmanni*	6.0	1	HBW4
Psittaciformes	Psittacidae	*Enicognathus ferrugineus*	5.7	1	HBW4

Order	Family	*Species*	Clutch	Range	Traits
Psittaciformes	Psittacidae	*Enicognathus leptorhynchus*	3.5	1	HBW4
Psittaciformes	Psittacidae	*Bolborhynchus aymara*	4.9	1	HBW4
Psittaciformes	Psittacidae	*Bolborhynchus aurifrons*	4.2	1	HBW4
Psittaciformes	Psittacidae	*Forpus cyanopygius*	3.0	1	HBW4
Psittaciformes	Psittacidae	*Forpus passerinus*	5.0	1	HBW4
Psittaciformes	Psittacidae	*Forpus xanthopterygius*	5.3	1	HBW4
Psittaciformes	Psittacidae	*Forpus conspicillatus*	2.4	1	HBW4
Psittaciformes	Psittacidae	*Forpus coelestis*	4.9	1	HBW4
Psittaciformes	Psittacidae	*Forpus xanthops*	4.2	1	HBW4
Psittaciformes	Psittacidae	*Brotogeris tirica*	4.0	1	HBW4
Psittaciformes	Psittacidae	*Brotogeris versicolurus*	4.5	1	HBW4
Psittaciformes	Psittacidae	*Brotogeris pyrrhopterus*	5.3	1	HBW4
Psittaciformes	Psittacidae	*Brotogeris jugularis*	5.3	1	HBW4
Psittaciformes	Psittacidae	*Brotogeris cyanoptera*	5.0	1	HBW4
Psittaciformes	Psittacidae	*Brotogeris chrysopterus*	3.5	1	HBW4
Psittaciformes	Psittacidae	*Touit batavica*	5.5	1	HBW4
Psittaciformes	Psittacidae	*Touit costaricensis*	2.4	1	HBW4
Psittaciformes	Psittacidae	*Touit purpurata*	3.9	1	HBW4
Psittaciformes	Psittacidae	*Pionites melanocephala*	2.0	1	HBW4
Psittaciformes	Psittacidae	*Pionites leucogaster*	2.0	1	HBW4
Psittaciformes	Psittacidae	*Pionopsitta pileata*	2.0	1	HBW4
Psittaciformes	Psittacidae	*Hapalopsittaca pyrrhops*	2.0	1	HBW4
Psittaciformes	Psittacidae	*Amazona leucocephala*	3.5	1	HBW4
Psittaciformes	Psittacidae	*Amazona collaria*	3.5	1	HBW4
Psittaciformes	Psittacidae	*Amazona ventralis*	2.8	1	HBW4
Psittaciformes	Psittacidae	*Amazona albifrons*	3.9	1	HBW4
Psittaciformes	Psittacidae	*Amazona xantholora*	4.0	1	HBW4
Psittaciformes	Psittacidae	*Amazona agilis*	2.8	1	HBW4
Psittaciformes	Psittacidae	*Amazona vittata*	2.8	1	HBW4
Psittaciformes	Psittacidae	*Amazona tucumana*	4.0	1	HBW4
Psittaciformes	Psittacidae	*Amazona pretrei*	2.8	1	HBW4
Psittaciformes	Psittacidae	*Amazona viridigenalis*	3.5	1	HBW4
Psittaciformes	Psittacidae	*Amazona finschi*	2.0	1	HBW4
Psittaciformes	Psittacidae	*Amazona autumnalis*	3.5	1	HBW4
Psittaciformes	Psittacidae	*Amazona dufresniana*	3.0	1	HBW4
Psittaciformes	Psittacidae	*Amazona rhodocorytha*	4.0	1	HBW4
Psittaciformes	Psittacidae	*Amazona brasiliensis*	3.5	1	HBW4
Psittaciformes	Psittacidae	*Amazona barbadensis*	3.2	1	HBW4
Psittaciformes	Psittacidae	*Amazona aestiva*	3.0	1	HBW4
Psittaciformes	Psittacidae	*Amazona ochrocephala*	2.4	1	HBW4
Psittaciformes	Psittacidae	*Amazona amazonica*	3.2	1	HBW4
Psittaciformes	Psittacidae	*Amazona farinosa*	3.0	1	HBW4
Psittaciformes	Psittacidae	*Amazona vinacea*	2.8	1	HBW4
Psittaciformes	Psittacidae	*Deroptyus accipitrinus*	2.4	1	HBW4
Psittaciformes	Psittacidae	*Triclaria malachitacea*	2.8	1	HBW4
Apodiformes	Apodidae	*Cypseloides niger*	1.0	1	HBW5
Apodiformes	Apodidae	*Cypseloides fumigatus*	1.0	1	HBW5
Apodiformes	Apodidae	*Cypseloides cherriei*	1.0	1	HBW5
Apodiformes	Apodidae	*Cypseloides cryptus*	1.0	1	HBW5
Apodiformes	Apodidae	*Streptoprocne rutila*	2.0	1	HBW5
Apodiformes	Apodidae	*Streptoprocne zonaris*	2.0	1	HBW5
Apodiformes	Apodidae	*Streptoprocne semicollaris*	2.0	1	HBW5
Apodiformes	Apodidae	*Collocalia esculenta*	1.4	1	HBW5
Apodiformes	Apodidae	*Aerodramus francicus*	2.0	1	HBW5
Apodiformes	Apodidae	*Aerodramus hirundinacea*	1.0	1	HBW5
Apodiformes	Apodidae	*Aerodramus spodiopygius*	1.4	1	HBW5
Apodiformes	Apodidae	*Aerodramus terraereginae*	1.0	1	HBW5
Apodiformes	Apodidae	*Aerodramus salanganus*	1.4	1	HBW5
Apodiformes	Apodidae	*Aerodramus vanikorensis*	1.4	1	HBW5
Apodiformes	Apodidae	*Zoonavena sylvatica*	3.9	1	HBW5
Apodiformes	Apodidae	*Telacanthura ussheri*	4.0	1	HBW5
Apodiformes	Apodidae	*Rhaphidura sabini*	2.4	1	HBW5
Apodiformes	Apodidae	*Neafrapus boehmi*	3.0	1	HBW5

Order	Family	*Species*	Clutch	Range	Traits
Apodiformes	Apodidae	*Hirundapus caudacutus*	3.7	1	HBW5
Apodiformes	Apodidae	*Hirundapus giganteus*	3.9	1	HBW5
Apodiformes	Apodidae	*Chaetura cinereiventris*	2.8	1	HBW5
Apodiformes	Apodidae	*Chaetura pelagica*	3.7	1	HBW5
Apodiformes	Apodidae	*Chaetura vauxi*	2.6	1	HBW5
Apodiformes	Apodidae	*Chaetura chapmani*	2.0	1	HBW5
Apodiformes	Apodidae	*Chaetura brachyura*	3.7	1	HBW5
Apodiformes	Apodidae	*Chaetura andrei*	3.5	1	HBW5
Apodiformes	Apodidae	*Aeronautes saxatalis*	4.2	1	HBW5
Apodiformes	Apodidae	*Cypsiurus parvus*	2.4	1	HBW5
Apodiformes	Apodidae	*Cypsiurus balasiensis*	1.7	1	HBW5
Apodiformes	Apodidae	*Tachymarptis melba*	2.0	1	HBW5
Apodiformes	Apodidae	*Tachymarptis aequatorialis*	1.4	1	HBW5
Apodiformes	Apodidae	*Apus apus*	2.0	1	HBW5
Apodiformes	Apodidae	*Apus unicolor*	2.0	1	HBW5
Apodiformes	Apodidae	*Apus niansae*	1.7	1	HBW5
Apodiformes	Apodidae	*Apus pallidus*	2.4	1	HBW5
Apodiformes	Apodidae	*Apus barbatus*	1.4	1	HBW5
Apodiformes	Apodidae	*Apus berliozi*	2.0	1	HBW5
Apodiformes	Apodidae	*Apus bradfieldi*	2.0	1	HBW5
Apodiformes	Apodidae	*Apus pacificus*	1.7	1	HBW5
Apodiformes	Apodidae	*Apus acuticauda*	2.8	1	HBW5
Apodiformes	Apodidae	*Apus affinis*	1.7	1	HBW5
Apodiformes	Apodidae	*Apus nipalensis*	2.2	1	HBW5
Apodiformes	Apodidae	*Apus horus*	2.0	1	HBW5
Apodiformes	Apodidae	*Apus caffer*	1.7	1	HBW5
Apodiformes	Apodidae	*Apus batesi*	2.0	1	HBW5
Apodiformes	Hemiprocnidae	*Hemiprocne mystacea*	1.0	1	HBW5
Trochiliformes	Trochilidae	*Glaucis aenea*	2.0	1	HBW5
Trochiliformes	Trochilidae	*Glaucis hirsuta*	2.0	1	HBW5
Trochiliformes	Trochilidae	*Threnetes niger*	2.0	1	HBW5
Trochiliformes	Trochilidae	*Threnetes ruckeri*	2.0	1	HBW5
Trochiliformes	Trochilidae	*Phaethornis yaruqui*	2.0	1	HBW5
Trochiliformes	Trochilidae	*Phaethornis guy*	2.0	1	HBW5
Trochiliformes	Trochilidae	*Phaethornis syrmatophorus*	2.0	1	HBW5
Trochiliformes	Trochilidae	*Phaethornis superciliosus*	2.0	1	HBW5
Trochiliformes	Trochilidae	*Phaethornis malaris*	2.0	1	HBW5
Trochiliformes	Trochilidae	*Phaethornis eurynome*	2.0	1	HBW5
Trochiliformes	Trochilidae	*Phaethornis hispidus*	2.0	1	HBW5
Trochiliformes	Trochilidae	*Phaethornis anthophilus*	2.0	1	HBW5
Trochiliformes	Trochilidae	*Phaethornis bourcieri*	2.0	1	HBW5
Trochiliformes	Trochilidae	*Phaethornis koepckeae*	2.0	1	HBW5
Trochiliformes	Trochilidae	*Phaethornis squalidus*	2.0	1	HBW5
Trochiliformes	Trochilidae	*Phaethornis augusti*	2.0	1	HBW5
Trochiliformes	Trochilidae	*Phaethornis pretrei*	2.0	1	HBW5
Trochiliformes	Trochilidae	*Phaethornis ruber*	2.0	1	HBW5
Trochiliformes	Trochilidae	*Phaethornis longuemareus*	2.0	1	HBW5
Trochiliformes	Trochilidae	*Phaethornis striigularis*	2.0	1	HBW5
Trochiliformes	Trochilidae	*Phaethornis idaliae*	2.0	1	HBW5
Trochiliformes	Trochilidae	*Eutoxeres aquila*	2.0	1	HBW5
Trochiliformes	Trochilidae	*Eutoxeres condamini*	2.0	1	HBW5
Trochiliformes	Trochilidae	*Ramphodon dohrnii*	2.0	1	HBW5
Trochiliformes	Trochilidae	*Doryfera johannae*	2.0	1	HBW5
Trochiliformes	Trochilidae	*Doryfera ludovicae*	2.0	1	HBW5
Trochiliformes	Trochilidae	*Phaeochroa cuvierii*	2.0	1	HBW5
Trochiliformes	Trochilidae	*Campylopterus largipennis*	2.0	1	HBW5
Trochiliformes	Trochilidae	*Campylopterus rufus*	2.0	1	HBW5
Trochiliformes	Trochilidae	*Campylopterus ensipennis*	2.0	1	HBW5
Trochiliformes	Trochilidae	*Eupetomena macroura*	2.0	1	HBW5
Trochiliformes	Trochilidae	*Melanotrochilus fuscus*	2.0	1	HBW5
Trochiliformes	Trochilidae	*Colibri thalassinus*	2.0	1	HBW5
Trochiliformes	Trochilidae	*Colibri serrirostris*	2.0	1	HBW5
Trochiliformes	Trochilidae	*Anthracothorax viridigula*	2.0	1	HBW5

Order	Family	*Species*	Clutch	Range	Traits
Trochiliformes	Trochilidae	*Anthracothorax prevostii*	2.0	1	HBW5
Trochiliformes	Trochilidae	*Anthracothorax nigricollis*	2.0	1	HBW5
Trochiliformes	Trochilidae	*Anthracothorax mango*	2.0	1	HBW5
Trochiliformes	Trochilidae	*Anthracothorax dominicus*	2.0	1	HBW5
Trochiliformes	Trochilidae	*Anthracothorax viridis*	2.0	1	HBW5
Trochiliformes	Trochilidae	*Avocettula recurvirostris*	2.0	1	HBW5
Trochiliformes	Trochilidae	*Eulampis holosericeus*	2.0	1	HBW5
Trochiliformes	Trochilidae	*Chrysolampis mosquitus*	2.0	1	HBW5
Trochiliformes	Trochilidae	*Orthorhyncus cristatus*	2.0	1	HBW5
Trochiliformes	Trochilidae	*Klais guimeti*	2.0	1	HBW5
Trochiliformes	Trochilidae	*Abeillia abeillei*	2.0	1	HBW5
Trochiliformes	Trochilidae	*Stephanoxis lalandi*	2.0	1	HBW5
Trochiliformes	Trochilidae	*Lophornis ornatus*	2.0	1	HBW5
Trochiliformes	Trochilidae	*Lophornis gouldii*	2.0	1	HBW5
Trochiliformes	Trochilidae	*Lophornis magnificus*	2.0	1	HBW5
Trochiliformes	Trochilidae	*Lophornis chalybeus*	2.0	1	HBW5
Trochiliformes	Trochilidae	*Lophornis pavoninus*	2.0	1	HBW5
Trochiliformes	Trochilidae	*Lophornis adorabilis*	2.0	1	HBW5
Trochiliformes	Trochilidae	*Discosura longicauda*	2.0	1	HBW5
Trochiliformes	Trochilidae	*Chlorestes notatus*	2.0	1	HBW5
Trochiliformes	Trochilidae	*Chlorostilbon mellisugus*	2.0	1	HBW5
Trochiliformes	Trochilidae	*Chlorostilbon aureoventris*	2.0	1	HBW5
Trochiliformes	Trochilidae	*Chlorostilbon ricordii*	2.0	1	HBW5
Trochiliformes	Trochilidae	*Chlorostilbon swainsonii*	2.0	1	HBW5
Trochiliformes	Trochilidae	*Chlorostilbon maugaeus*	2.0	1	HBW5
Trochiliformes	Trochilidae	*Chlorostilbon russatus*	2.0	1	HBW5
Trochiliformes	Trochilidae	*Chlorostilbon stenura*	2.0	1	HBW5
Trochiliformes	Trochilidae	*Chlorostilbon alice*	2.0	1	HBW5
Trochiliformes	Trochilidae	*Chlorostilbon poortmani*	2.0	1	HBW5
Trochiliformes	Trochilidae	*Cynanthus sordidus*	2.0	1	HBW5
Trochiliformes	Trochilidae	*Cynanthus latirostris*	2.0	1	HBW5
Trochiliformes	Trochilidae	*Thalurania fannyi*	2.0	1	HBW5
Trochiliformes	Trochilidae	*Thalurania watertonii*	2.0	1	HBW5
Trochiliformes	Trochilidae	*Thalurania glaucopis*	2.0	1	HBW5
Trochiliformes	Trochilidae	*Panterpe insignis*	2.0	1	HBW5
Trochiliformes	Trochilidae	*Damophila julie*	2.0	1	HBW5
Trochiliformes	Trochilidae	*Lepidopyga coeruleogularis*	2.0	1	HBW5
Trochiliformes	Trochilidae	*Hylocharis xantusii*	2.0	1	HBW5
Trochiliformes	Trochilidae	*Hylocharis leucotis*	2.0	1	HBW5
Trochiliformes	Trochilidae	*Hylocharis eliciae*	2.0	1	HBW5
Trochiliformes	Trochilidae	*Hylocharis sapphirina*	2.0	1	HBW5
Trochiliformes	Trochilidae	*Hylocharis cyanus*	2.0	1	HBW5
Trochiliformes	Trochilidae	*Hylocharis chrysura*	2.0	1	HBW5
Trochiliformes	Trochilidae	*Trochilus polytmus*	2.0	1	HBW5
Trochiliformes	Trochilidae	*Leucochloris albicollis*	2.0	1	HBW5
Trochiliformes	Trochilidae	*Polytmus guainumbi*	2.0	1	HBW5
Trochiliformes	Trochilidae	*Polytmus milleri*	2.0	1	HBW5
Trochiliformes	Trochilidae	*Polytmus theresiae*	2.0	1	HBW5
Trochiliformes	Trochilidae	*Leucippus fallax*	2.0	1	HBW5
Trochiliformes	Trochilidae	*Leucippus chlorocercus*	2.0	1	HBW5
Trochiliformes	Trochilidae	*Taphrospilus hypostictus*	2.0	1	HBW5
Trochiliformes	Trochilidae	*Amazilia viridicauda*	2.0	1	HBW5
Trochiliformes	Trochilidae	*Amazilia chionogaster*	2.0	1	HBW5
Trochiliformes	Trochilidae	*Amazilia candida*	2.0	1	HBW5
Trochiliformes	Trochilidae	*Amazilia chionopectus*	2.0	1	HBW5
Trochiliformes	Trochilidae	*Amazilia versicolor*	2.0	1	HBW5
Trochiliformes	Trochilidae	*Amazilia luciae*	2.0	1	HBW5
Trochiliformes	Trochilidae	*Amazilia fimbriata*	2.0	1	HBW5
Trochiliformes	Trochilidae	*Amazilia lactea*	2.0	1	HBW5
Trochiliformes	Trochilidae	*Amazilia amabilis*	2.0	1	HBW5
Trochiliformes	Trochilidae	*Amazilia rosenbergi*	2.0	1	HBW5
Trochiliformes	Trochilidae	*Amazilia boucardi*	2.0	1	HBW5
Trochiliformes	Trochilidae	*Amazilia franciae*	2.0	1	HBW5

Order	Family	*Species*	Clutch	Range	Traits
Trochiliformes	Trochilidae	*Amazilia leucogaster*	2.0	1	HBW5
Trochiliformes	Trochilidae	*Amazilia cyanocephala*	2.0	1	HBW5
Trochiliformes	Trochilidae	*Amazilia cyanifrons*	2.0	1	HBW5
Trochiliformes	Trochilidae	*Amazilia beryllina*	2.0	1	HBW5
Trochiliformes	Trochilidae	*Amazilia saucerrottei*	2.0	1	HBW5
Trochiliformes	Trochilidae	*Amazilia tobaci*	2.0	1	HBW5
Trochiliformes	Trochilidae	*Amazilia viridigaster*	2.0	1	HBW5
Trochiliformes	Trochilidae	*Amazilia edward*	2.0	1	HBW5
Trochiliformes	Trochilidae	*Amazilia rutila*	2.0	1	HBW5
Trochiliformes	Trochilidae	*Amazilia yucatanensis*	2.0	1	HBW5
Trochiliformes	Trochilidae	*Amazilia tzacatl*	2.0	1	HBW5
Trochiliformes	Trochilidae	*Amazilia castaneiventris*	2.0	1	HBW5
Trochiliformes	Trochilidae	*Amazilia amazilia*	2.0	1	HBW5
Trochiliformes	Trochilidae	*Amazilia viridifrons*	2.0	1	HBW5
Trochiliformes	Trochilidae	*Amazilia violiceps*	2.0	1	HBW5
Trochiliformes	Trochilidae	*Eupherusa cyanophrys*	2.0	1	HBW5
Trochiliformes	Trochilidae	*Eupherusa nigriventris*	2.0	1	HBW5
Trochiliformes	Trochilidae	*Elvira cupreiceps*	2.0	1	HBW5
Trochiliformes	Trochilidae	*Aphantochroa cirrochloris*	2.0	1	HBW5
Trochiliformes	Trochilidae	*Lampornis clemenciae*	2.0	1	HBW5
Trochiliformes	Trochilidae	*Lampornis amethystinus*	2.0	1	HBW5
Trochiliformes	Trochilidae	*Lampornis castaneoventris*	2.0	1	HBW5
Trochiliformes	Trochilidae	*Lamprolaima rhami*	2.0	1	HBW5
Trochiliformes	Trochilidae	*Adelomyia melanogenys*	2.0	1	HBW5
Trochiliformes	Trochilidae	*Phlogophilus hemileucurus*	2.0	1	HBW5
Trochiliformes	Trochilidae	*Clytolaema rubricauda*	2.0	1	HBW5
Trochiliformes	Trochilidae	*Heliodoxa xanthogonys*	2.0	1	HBW5
Trochiliformes	Trochilidae	*Heliodoxa schreibersii*	2.0	1	HBW5
Trochiliformes	Trochilidae	*Heliodoxa aurescens*	2.0	1	HBW5
Trochiliformes	Trochilidae	*Heliodoxa rubinoides*	2.0	1	HBW5
Trochiliformes	Trochilidae	*Heliodoxa leadbeateri*	2.0	1	HBW5
Trochiliformes	Trochilidae	*Eugenes fulgens*	2.0	1	HBW5
Trochiliformes	Trochilidae	*Topaza pella*	2.0	1	HBW5
Trochiliformes	Trochilidae	*Oreotrochilus chimborazo*	2.0	1	HBW5
Trochiliformes	Trochilidae	*Oreotrochilus estella*	2.0	1	HBW5
Trochiliformes	Trochilidae	*Oreotrochilus leucopleurus*	2.0	1	HBW5
Trochiliformes	Trochilidae	*Oreotrochilus melanogaster*	2.0	1	HBW5
Trochiliformes	Trochilidae	*Urochroa bougueri*	2.0	1	HBW5
Trochiliformes	Trochilidae	*Patagona gigas*	2.0	1	HBW5
Trochiliformes	Trochilidae	*Aglaeactis cupripennis*	2.0	1	HBW5
Trochiliformes	Trochilidae	*Lafresnaya lafresnayi*	2.0	1	HBW5
Trochiliformes	Trochilidae	*Pterophanes cyanopterus*	2.0	1	HBW5
Trochiliformes	Trochilidae	*Coeligena coeligena*	2.0	1	HBW5
Trochiliformes	Trochilidae	*Coeligena wilsoni*	2.0	1	HBW5
Trochiliformes	Trochilidae	*Coeligena torquata*	2.0	1	HBW5
Trochiliformes	Trochilidae	*Coeligena lutetiae*	2.0	1	HBW5
Trochiliformes	Trochilidae	*Sephanoides sephaniodes*	2.0	1	HBW5
Trochiliformes	Trochilidae	*Boissonneaua flavescens*	2.0	1	HBW5
Trochiliformes	Trochilidae	*Boissonneaua matthewsii*	2.0	1	HBW5
Trochiliformes	Trochilidae	*Boissonneaua jardini*	2.0	1	HBW5
Trochiliformes	Trochilidae	*Heliangelus mavors*	2.0	1	HBW5
Trochiliformes	Trochilidae	*Heliangelus amethysticollis*	2.0	1	HBW5
Trochiliformes	Trochilidae	*Heliangelus strophianus*	2.0	1	HBW5
Trochiliformes	Trochilidae	*Heliangelus exortis*	2.0	1	HBW5
Trochiliformes	Trochilidae	*Heliangelus viola*	2.0	1	HBW5
Trochiliformes	Trochilidae	*Heliangelus regalis*	2.0	1	HBW5
Trochiliformes	Trochilidae	*Eriocnemis nigrivestis*	2.0	1	HBW5
Trochiliformes	Trochilidae	*Eriocnemis vestitus*	2.0	1	HBW5
Trochiliformes	Trochilidae	*Eriocnemis godini*	2.0	1	HBW5
Trochiliformes	Trochilidae	*Eriocnemis luciani*	2.0	1	HBW5
Trochiliformes	Trochilidae	*Eriocnemis cupreoventris*	2.0	1	HBW5
Trochiliformes	Trochilidae	*Eriocnemis mosquera*	2.0	1	HBW5
Trochiliformes	Trochilidae	*Eriocnemis glaucopoides*	2.0	1	HBW5

Order	Family	*Species*	Clutch	Range	Traits
Trochiliformes	Trochilidae	*Eriocnemis derbyi*	2.0	1	HBW5
Trochiliformes	Trochilidae	*Haplophaedia aureliae*	2.0	1	HBW5
Trochiliformes	Trochilidae	*Haplophaedia lugens*	2.0	1	HBW5
Trochiliformes	Trochilidae	*Urosticte benjamini*	2.0	1	HBW5
Trochiliformes	Trochilidae	*Urosticte ruficrissa*	2.0	1	HBW5
Trochiliformes	Trochilidae	*Ocreatus underwoodii*	2.0	1	HBW5
Trochiliformes	Trochilidae	*Lesbia nuna*	2.0	1	HBW5
Trochiliformes	Trochilidae	*Sappho sparganura*	2.0	1	HBW5
Trochiliformes	Trochilidae	*Ramphomicron microrhynchum*	2.0	1	HBW5
Trochiliformes	Trochilidae	*Aglaiocercus kingi*	2.0	1	HBW5
Trochiliformes	Trochilidae	*Aglaiocercus coelestis*	2.0	1	HBW5
Trochiliformes	Trochilidae	*Augastes lumachellus*	2.0	1	HBW5
Trochiliformes	Trochilidae	*Augastes scutatus*	2.0	1	HBW5
Trochiliformes	Trochilidae	*Augastes geoffroyi*	2.0	1	HBW5
Trochiliformes	Trochilidae	*Heliothryx barroti*	2.0	1	HBW5
Trochiliformes	Trochilidae	*Heliothryx aurita*	2.0	1	HBW5
Trochiliformes	Trochilidae	*Heliactin bilophum*	2.0	1	HBW5
Trochiliformes	Trochilidae	*Heliomaster longirostris*	2.0	1	HBW5
Trochiliformes	Trochilidae	*Heliomaster squamosus*	2.0	1	HBW5
Trochiliformes	Trochilidae	*Heliomaster furcifer*	2.0	1	HBW5
Trochiliformes	Trochilidae	*Rhodopis vesper*	2.0	1	HBW5
Trochiliformes	Trochilidae	*Thaumastura cora*	2.0	1	HBW5
Trochiliformes	Trochilidae	*Philodice mitchellii*	2.0	1	HBW5
Trochiliformes	Trochilidae	*Doricha eliza*	2.0	1	HBW5
Trochiliformes	Trochilidae	*Calothorax lucifer*	2.0	1	HBW5
Trochiliformes	Trochilidae	*Calothorax pulcher*	2.0	1	HBW5
Trochiliformes	Trochilidae	*Archilochus colubris*	2.0	1	HBW5
Trochiliformes	Trochilidae	*Archilochus alexandri*	2.0	1	HBW5
Trochiliformes	Trochilidae	*Calypte anna*	2.0	1	HBW5
Trochiliformes	Trochilidae	*Calypte costae*	2.0	1	HBW5
Trochiliformes	Trochilidae	*Calliphlox evelynae*	2.0	1	HBW5
Trochiliformes	Trochilidae	*Calliphlox amethystina*	2.0	1	HBW5
Trochiliformes	Trochilidae	*Mellisuga helenae*	2.0	1	HBW5
Trochiliformes	Trochilidae	*Mellisuga minima*	2.0	1	HBW5
Trochiliformes	Trochilidae	*Stellula calliope*	2.0	1	HBW5
Trochiliformes	Trochilidae	*Myrtis fanny*	2.0	1	HBW5
Trochiliformes	Trochilidae	*Myrmia micrura*	2.0	1	HBW5
Trochiliformes	Trochilidae	*Selasphorus platycercus*	2.0	1	HBW5
Trochiliformes	Trochilidae	*Selasphorus rufus*	2.0	1	HBW5
Trochiliformes	Trochilidae	*Selasphorus sasin*	2.0	1	HBW5
Musophagiforme	Musophagidae	*Tauraco persa*	2.0	1	HBW4
Musophagiforme	Musophagidae	*Tauraco schuettii*	2.0	1	HBW4
Musophagiforme	Musophagidae	*Tauraco schalowi*	2.0	1	HBW4
Musophagiforme	Musophagidae	*Tauraco fischeri*	2.0	1	HBW4
Musophagiforme	Musophagidae	*Tauraco livingstonii*	2.0	1	HBW4
Musophagiforme	Musophagidae	*Tauraco corythaix*	2.4	1	HBW4
Musophagiforme	Musophagidae	*Tauraco bannermani*	2.0	1	HBW4
Musophagiforme	Musophagidae	*Tauraco macrorhynchus*	2.0	1	HBW4
Musophagiforme	Musophagidae	*Tauraco leucotis*	2.0	1	HBW4
Musophagiforme	Musophagidae	*Tauraco hartlaubi*	2.0	1	HBW4
Musophagiforme	Musophagidae	*Tauraco leucolophus*	2.0	1	HBW4
Musophagiforme	Musophagidae	*Musophaga johnstoni*	1.4	1	HBW4
Musophagiforme	Musophagidae	*Musophaga porphyreolopha*	2.4	1	HBW4
Musophagiforme	Musophagidae	*Musophaga violacea*	1.4	1	HBW4
Musophagiforme	Musophagidae	*Musophaga rossae*	1.4	1	HBW4
Musophagiforme	Musophagidae	*Corythaixoides concolor*	3.0	1	HBW4
Musophagiforme	Musophagidae	*Corythaixoides personatus*	2.4	1	HBW4
Musophagiforme	Musophagidae	*Corythaixoides leucogaster*	2.4	1	HBW4
Musophagiforme	Musophagidae	*Crinifer piscator*	2.4	1	HBW4
Musophagiforme	Musophagidae	*Crinifer zonurus*	2.4	1	HBW4
Musophagiforme	Musophagidae	*Corythaeola cristata*	2.0	1	HBW4
Strigiformes	Tytonidae	*Tyto tenebricosa*	1.4	1	HBW5
Strigiformes	Tytonidae	*Tyto multipunctata*	2.0	1	HBW5

Order	Family	*Species*	Clutch	Range	Traits
Strigiformes	Tytonidae	*Tyto novaehollandiae*	2.8	1	HBW5
Strigiformes	Tytonidae	*Tyto alba*	5.3	1	HBW5
Strigiformes	Tytonidae	*Tyto glaucops*	4.6	1	HBW5
Strigiformes	Tytonidae	*Tyto capensis*	3.2	1	HBW5
Strigiformes	Tytonidae	*Tyto longimembris*	4.9	1	HBW5
Strigiformes	Strigidae	*Otus sagittatus*	3.5	1	HBW5
Strigiformes	Strigidae	*Otus balli*	2.4	1	HBW5
Strigiformes	Strigidae	*Otus spilocephalus*	2.4	1	HBW5
Strigiformes	Strigidae	*Otus longicornis*	2.4	1	HBW5
Strigiformes	Strigidae	*Otus brucei*	4.9	1	HBW5
Strigiformes	Strigidae	*Otus flammeolus*	2.8	1	HBW5
Strigiformes	Strigidae	*Otus scops*	3.5	1	HBW5
Strigiformes	Strigidae	*Otus sunia*	4.2	1	HBW5
Strigiformes	Strigidae	*Otus senegalensis*	2.8	1	HBW5
Strigiformes	Strigidae	*Otus rutilus*	4.5	1	HBW5
Strigiformes	Strigidae	*Otus bakkamoena*	3.9	1	HBW5
Strigiformes	Strigidae	*Otus lettia*	3.9	1	HBW5
Strigiformes	Strigidae	*Otus lempiji*	3.9	1	HBW5
Strigiformes	Strigidae	*Otus semitorques*	3.9	1	HBW5
Strigiformes	Strigidae	*Otus megalotis*	2.0	1	HBW5
Strigiformes	Strigidae	*Otus leucotis*	2.4	1	HBW5
Strigiformes	Strigidae	*Otus kennicottii*	4.9	1	HBW5
Strigiformes	Strigidae	*Otus cooperi*	3.9	1	HBW5
Strigiformes	Strigidae	*Otus asio*	3.5	1	HBW5
Strigiformes	Strigidae	*Otus trichopsis*	3.5	1	HBW5
Strigiformes	Strigidae	*Otus choliba*	2.0	1	HBW5
Strigiformes	Strigidae	*Otus barbarus*	4.5	1	HBW5
Strigiformes	Strigidae	*Otus guatemalae*	3.0	1	HBW5
Strigiformes	Strigidae	*Otus vermiculatus*	3.0	1	HBW5
Strigiformes	Strigidae	*Otus lawrencii*	2.0	1	HBW5
Strigiformes	Strigidae	*Otus nudipes*	2.0	1	HBW5
Strigiformes	Strigidae	*Bubo virginianus*	1.7	1	HBW5
Strigiformes	Strigidae	*Bubo bubo*	2.8	1	HBW5
Strigiformes	Strigidae	*Bubo bengalensis*	3.2	1	HBW5
Strigiformes	Strigidae	*Bubo ascalaphus*	2.0	1	HBW5
Strigiformes	Strigidae	*Bubo capensis*	1.7	1	HBW5
Strigiformes	Strigidae	*Bubo africanus*	2.8	1	HBW5
Strigiformes	Strigidae	*Bubo nipalensis*	1.0	1	HBW5
Strigiformes	Strigidae	*Bubo lacteus*	1.4	1	HBW5
Strigiformes	Strigidae	*Bubo coromandus*	1.7	1	HBW5
Strigiformes	Strigidae	*Nyctea scandiaca*	3.9	1	HBW5
Strigiformes	Strigidae	*Scotopelia peli*	1.4	1	HBW5
Strigiformes	Strigidae	*Scotopelia bouvieri*	1.4	1	HBW5
Strigiformes	Strigidae	*Strix seloputo*	2.4	1	HBW5
Strigiformes	Strigidae	*Strix ocellata*	2.4	1	HBW5
Strigiformes	Strigidae	*Strix leptogrammica*	1.4	1	HBW5
Strigiformes	Strigidae	*Strix aluco*	3.2	1	HBW5
Strigiformes	Strigidae	*Strix butleri*	2.2	1	HBW5
Strigiformes	Strigidae	*Strix occidentalis*	2.0	1	HBW5
Strigiformes	Strigidae	*Strix varia*	3.2	1	HBW5
Strigiformes	Strigidae	*Strix hylophila*	2.4	1	HBW5
Strigiformes	Strigidae	*Strix rufipes*	2.4	1	HBW5
Strigiformes	Strigidae	*Strix uralensis*	2.8	1	HBW5
Strigiformes	Strigidae	*Strix nebulosa*	4.2	1	HBW5
Strigiformes	Strigidae	*Strix virgata*	1.4	1	HBW5
Strigiformes	Strigidae	*Strix nigrolineata*	1.4	1	HBW5
Strigiformes	Strigidae	*Strix woodfordii*	1.7	1	HBW5
Strigiformes	Strigidae	*Surnia ulula*	7.7	1	HBW5
Strigiformes	Strigidae	*Glaucidium passerinum*	5.3	1	HBW5
Strigiformes	Strigidae	*Glaucidium brodiei*	4.2	1	HBW5
Strigiformes	Strigidae	*Glaucidium perlatum*	3.0	1	HBW5
Strigiformes	Strigidae	*Glaucidium californicum*	3.7	1	HBW5
Strigiformes	Strigidae	*Glaucidium gnoma*	2.8	1	HBW5

Order	Family	*Species*	Clutch	Range	Traits
Strigiformes	Strigidae	*Glaucidium palmarum*	2.8	1	HBW5
Strigiformes	Strigidae	*Glaucidium sanchezi*	2.8	1	HBW5
Strigiformes	Strigidae	*Glaucidium griseiceps*	2.8	1	HBW5
Strigiformes	Strigidae	*Glaucidium brasilianum*	3.2	1	HBW5
Strigiformes	Strigidae	*Glaucidium tucumanum*	3.2	1	HBW5
Strigiformes	Strigidae	*Glaucidium nanum*	3.9	1	HBW5
Strigiformes	Strigidae	*Glaucidium siju*	3.5	1	HBW5
Strigiformes	Strigidae	*Glaucidium tephronotum*	2.8	1	HBW5
Strigiformes	Strigidae	*Glaucidium sjostedti*	2.0	1	HBW5
Strigiformes	Strigidae	*Glaucidium cuculoides*	3.9	1	HBW5
Strigiformes	Strigidae	*Glaucidium radiatum*	2.8	1	HBW5
Strigiformes	Strigidae	*Glaucidium capense*	2.4	1	HBW5
Strigiformes	Strigidae	*Glaucidium castaneum*	2.4	1	HBW5
Strigiformes	Strigidae	*Micrathene whitneyi*	2.2	1	HBW5
Strigiformes	Strigidae	*Athene noctua*	4.2	1	HBW5
Strigiformes	Strigidae	*Athene brama*	3.2	1	HBW5
Strigiformes	Strigidae	*Speotyto cunicularia*	8.1	1	HBW5
Strigiformes	Strigidae	*Aegolius funereus*	4.2	1	HBW5
Strigiformes	Strigidae	*Aegolius acadicus*	5.9	1	HBW5
Strigiformes	Strigidae	*Aegolius ridgwayi*	5.5	1	HBW5
Strigiformes	Strigidae	*Aegolius harrisii*	3.0	1	HBW5
Strigiformes	Strigidae	*Ninox rufa*	2.0	1	HBW5
Strigiformes	Strigidae	*Ninox strenua*	2.0	1	HBW5
Strigiformes	Strigidae	*Ninox connivens*	2.4	1	HBW5
Strigiformes	Strigidae	*Ninox boobook*	3.0	1	HBW5
Strigiformes	Strigidae	*Ninox novaeseelandiae*	2.0	1	HBW5
Strigiformes	Strigidae	*Ninox scutulata*	3.2	1	HBW5
Strigiformes	Strigidae	*Ninox superciliaris*	3.9	1	HBW5
Strigiformes	Strigidae	*Ninox theomacha*	2.0	1	HBW5
Strigiformes	Strigidae	*Asio stygius*	2.0	1	HBW5
Strigiformes	Strigidae	*Asio otus*	5.9	1	HBW5
Strigiformes	Strigidae	*Asio clamator*	3.0	1	HBW5
Strigiformes	Strigidae	*Asio flammeus*	7.1	1	HBW5
Strigiformes	Strigidae	*Asio capensis*	2.4	1	HBW5
Strigiformes	Aegothelidae	*Aegotheles cristatus*	3.5	1	HBW5
Strigiformes	Podargidae	*Podargus strigoides*	1.7	1	HBW5
Strigiformes	Podargidae	*Podargus papuensis*	1.0	1	HBW5
Strigiformes	Podargidae	*Podargus ocellatus*	1.0	1	HBW5
Strigiformes	Batrachostomidae	*Batrachostomus hodgsoni*	1.4	1	HBW5
Strigiformes	Batrachostomidae	*Batrachostomus javensis*	1.4	1	HBW5
Strigiformes	Steatornithidae	*Steatornis caripensis*	2.8	1	HBW5
Strigiformes	Eurostopodidae	*Eurostopodus argus*	1.0	1	HBW5
Strigiformes	Eurostopodidae	*Eurostopodus mystacalis*	1.0	1	HBW5
Strigiformes	Eurostopodidae	*Eurostopodus diabolicus*	1.0	1	HBW5
Strigiformes	Eurostopodidae	*Eurostopodus papuensis*	1.0	1	HBW5
Strigiformes	Eurostopodidae	*Eurostopodus archboldi*	1.0	1	HBW5
Strigiformes	Eurostopodidae	*Eurostopodus temminckii*	1.4	1	HBW5
Strigiformes	Eurostopodidae	*Eurostopodus macrotis*	1.0	1	HBW5
Strigiformes	Caprimulgidae	*Lurocalis semitorquatus*	1.0	1	HBW5
Strigiformes	Caprimulgidae	*Chordeiles pusillus*	1.0	1	HBW5
Strigiformes	Caprimulgidae	*Chordeiles rupestris*	1.4	1	HBW5
Strigiformes	Caprimulgidae	*Chordeiles acutipennis*	1.4	1	HBW5
Strigiformes	Caprimulgidae	*Chordeiles minor*	1.7	1	HBW5
Strigiformes	Caprimulgidae	*Chordeiles gundlachii*	1.4	1	HBW5
Strigiformes	Caprimulgidae	*Podager nacunda*	1.4	1	HBW5
Strigiformes	Caprimulgidae	*Nyctidromus albicollis*	1.4	1	HBW5
Strigiformes	Caprimulgidae	*Phalaenoptilus nuttallii*	2.0	1	HBW5
Strigiformes	Caprimulgidae	*Nyctiphrynus mcleodii*	2.0	1	HBW5
Strigiformes	Caprimulgidae	*Nyctiphrynus ocellatus*	2.0	1	HBW5
Strigiformes	Caprimulgidae	*Caprimulgus carolinensis*	2.0	1	HBW5
Strigiformes	Caprimulgidae	*Caprimulgus rufus*	1.4	1	HBW5
Strigiformes	Caprimulgidae	*Caprimulgus cubanensis*	1.4	1	HBW5
Strigiformes	Caprimulgidae	*Caprimulgus salvini*	2.0	1	HBW5

Order	Family	*Species*	Clutch	Range	Traits
Strigiformes	Caprimulgidae	*Caprimulgus badius*	2.0	1	HBW5
Strigiformes	Caprimulgidae	*Caprimulgus ridgwayi*	2.0	1	HBW5
Strigiformes	Caprimulgidae	*Caprimulgus vociferus*	1.4	1	HBW5
Strigiformes	Caprimulgidae	*Caprimulgus noctitherus*	1.4	1	HBW5
Strigiformes	Caprimulgidae	*Caprimulgus saturatus*	1.0	1	HBW5
Strigiformes	Caprimulgidae	*Caprimulgus longirostris*	1.4	1	HBW5
Strigiformes	Caprimulgidae	*Caprimulgus cayennensis*	1.4	1	HBW5
Strigiformes	Caprimulgidae	*Caprimulgus candicans*	2.0	1	HBW5
Strigiformes	Caprimulgidae	*Caprimulgus maculicaudus*	2.0	1	HBW5
Strigiformes	Caprimulgidae	*Caprimulgus parvulus*	2.0	1	HBW5
Strigiformes	Caprimulgidae	*Caprimulgus anthonyi*	1.4	1	HBW5
Strigiformes	Caprimulgidae	*Caprimulgus nigrescens*	1.0	1	HBW5
Strigiformes	Caprimulgidae	*Caprimulgus ruficollis*	1.4	1	HBW5
Strigiformes	Caprimulgidae	*Caprimulgus indicus*	1.4	1	HBW5
Strigiformes	Caprimulgidae	*Caprimulgus europaeus*	1.4	1	HBW5
Strigiformes	Caprimulgidae	*Caprimulgus fraenatus*	1.4	1	HBW5
Strigiformes	Caprimulgidae	*Caprimulgus rufigena*	1.4	1	HBW5
Strigiformes	Caprimulgidae	*Caprimulgus aegyptius*	1.4	1	HBW5
Strigiformes	Caprimulgidae	*Caprimulgus nubicus*	1.4	1	HBW5
Strigiformes	Caprimulgidae	*Caprimulgus eximius*	2.0	1	HBW5
Strigiformes	Caprimulgidae	*Caprimulgus madagascariensis*	2.0	1	HBW5
Strigiformes	Caprimulgidae	*Caprimulgus macrurus*	1.4	1	HBW5
Strigiformes	Caprimulgidae	*Caprimulgus manillensis*	1.4	1	HBW5
Strigiformes	Caprimulgidae	*Caprimulgus donaldsoni*	2.0	1	HBW5
Strigiformes	Caprimulgidae	*Caprimulgus pectoralis*	1.4	1	HBW5
Strigiformes	Caprimulgidae	*Caprimulgus poliocephalus*	2.0	1	HBW5
Strigiformes	Caprimulgidae	*Caprimulgus natalensis*	2.0	1	HBW5
Strigiformes	Caprimulgidae	*Caprimulgus inornatus*	2.0	1	HBW5
Strigiformes	Caprimulgidae	*Caprimulgus stellatus*	2.0	1	HBW5
Strigiformes	Caprimulgidae	*Caprimulgus affinis*	1.4	1	HBW5
Strigiformes	Caprimulgidae	*Caprimulgus tristigma*	2.0	1	HBW5
Strigiformes	Caprimulgidae	*Caprimulgus pulchellus*	1.4	1	HBW5
Strigiformes	Caprimulgidae	*Caprimulgus enarratus*	2.0	1	HBW5
Strigiformes	Caprimulgidae	*Caprimulgus batesi*	1.0	1	HBW5
Strigiformes	Caprimulgidae	*Caprimulgus climacurus*	2.0	1	HBW5
Strigiformes	Caprimulgidae	*Caprimulgus clarus*	2.0	1	HBW5
Strigiformes	Caprimulgidae	*Caprimulgus fossii*	1.4	1	HBW5
Strigiformes	Caprimulgidae	*Macrodipteryx longipennis*	1.4	1	HBW5
Strigiformes	Caprimulgidae	*Macrodipteryx vexillarius*	1.4	1	HBW5
Strigiformes	Caprimulgidae	*Hydropsalis climacocerca*	2.0	1	HBW5
Strigiformes	Caprimulgidae	*Hydropsalis brasiliana*	2.0	1	HBW5
Columbiformes	Columbidae	*Columba livia*	2.0	1	HBW4
Columbiformes	Columbidae	*Columba rupestris*	2.0	1	HBW4
Columbiformes	Columbidae	*Columba leuconota*	2.0	1	HBW4
Columbiformes	Columbidae	*Columba guinea*	2.0	1	HBW4
Columbiformes	Columbidae	*Columba albitorques*	2.0	1	HBW4
Columbiformes	Columbidae	*Columba oenas*	2.0	1	HBW4
Columbiformes	Columbidae	*Columba oliviae*	1.0	1	HBW4
Columbiformes	Columbidae	*Columba eversmanni*	2.0	1	HBW4
Columbiformes	Columbidae	*Columba palumbus*	2.0	1	HBW4
Columbiformes	Columbidae	*Columba bollii*	1.0	1	HBW4
Columbiformes	Columbidae	*Columba junoniae*	1.4	1	HBW4
Columbiformes	Columbidae	*Columba unicincta*	1.0	1	HBW4
Columbiformes	Columbidae	*Columba sjostedti*	1.0	1	HBW4
Columbiformes	Columbidae	*Columba arquatrix*	1.0	1	HBW4
Columbiformes	Columbidae	*Columba hodgsonii*	1.0	1	HBW4
Columbiformes	Columbidae	*Columba albinucha*	1.0	1	HBW4
Columbiformes	Columbidae	*Columba pulchricollis*	1.4	1	HBW4
Columbiformes	Columbidae	*Columba elphinstonii*	1.0	1	HBW4
Columbiformes	Columbidae	*Columba torringtoni*	1.0	1	HBW4
Columbiformes	Columbidae	*Columba punicea*	1.0	1	HBW4
Columbiformes	Columbidae	*Columba argentina*	1.0	1	HBW4
Columbiformes	Columbidae	*Columba janthina*	1.0	1	HBW4

Order	Family	*Species*	Clutch	Range	Traits
Columbiformes	Columbidae	*Columba vitiensis*	1.0	1	HBW4
Columbiformes	Columbidae	*Columba leucomela*	1.0	1	HBW4
Columbiformes	Columbidae	*Columba leucocephala*	2.0	1	HBW4
Columbiformes	Columbidae	*Columba speciosa*	2.0	1	HBW4
Columbiformes	Columbidae	*Columba squamosa*	1.4	1	HBW4
Columbiformes	Columbidae	*Columba corensis*	1.0	1	HBW4
Columbiformes	Columbidae	*Columba picazuro*	1.0	1	HBW4
Columbiformes	Columbidae	*Columba maculosa*	1.4	1	HBW4
Columbiformes	Columbidae	*Columba fasciata*	1.4	1	HBW4
Columbiformes	Columbidae	*Columba cayennensis*	1.0	1	HBW4
Columbiformes	Columbidae	*Columba flavirostris*	1.0	1	HBW4
Columbiformes	Columbidae	*Columba inornata*	1.4	1	HBW4
Columbiformes	Columbidae	*Columba subvinacea*	1.0	1	HBW4
Columbiformes	Columbidae	*Columba nigrirostris*	1.0	1	HBW4
Columbiformes	Columbidae	*Columba iriditorques*	1.0	1	HBW4
Columbiformes	Columbidae	*Columba delegorguei*	2.0	1	HBW4
Columbiformes	Columbidae	*Columba mayeri*	2.0	1	HBW4
Columbiformes	Columbidae	*Aplopelia larvata*	2.0	1	HBW4
Columbiformes	Columbidae	*Streptopelia picturata*	2.0	1	HBW4
Columbiformes	Columbidae	*Streptopelia turtur*	2.0	1	HBW4
Columbiformes	Columbidae	*Streptopelia hypopyrrha*	2.0	1	HBW4
Columbiformes	Columbidae	*Streptopelia lugens*	2.0	1	HBW4
Columbiformes	Columbidae	*Streptopelia orientalis*	2.0	1	HBW4
Columbiformes	Columbidae	*Streptopelia senegalensis*	2.0	1	HBW4
Columbiformes	Columbidae	*Streptopelia chinensis*	2.4	1	HBW4
Columbiformes	Columbidae	*Streptopelia decipiens*	2.0	1	HBW4
Columbiformes	Columbidae	*Streptopelia vinacea*	2.0	1	HBW4
Columbiformes	Columbidae	*Streptopelia capicola*	2.0	1	HBW4
Columbiformes	Columbidae	*Streptopelia tranquebarica*	2.0	1	HBW4
Columbiformes	Columbidae	*Streptopelia semitorquata*	2.0	1	HBW4
Columbiformes	Columbidae	*Streptopelia decaocto*	2.0	1	HBW4
Columbiformes	Columbidae	*Streptopelia roseogrisea*	2.0	1	HBW4
Columbiformes	Columbidae	*Streptopelia reichenowi*	2.0	1	HBW4
Columbiformes	Columbidae	*Streptopelia bitorquata*	2.0	1	HBW4
Columbiformes	Columbidae	*Macropygia unchall*	1.4	1	HBW4
Columbiformes	Columbidae	*Macropygia emiliana*	1.0	1	HBW4
Columbiformes	Columbidae	*Macropygia amboinensis*	1.0	1	HBW4
Columbiformes	Columbidae	*Macropygia phasianella*	1.0	1	HBW4
Columbiformes	Columbidae	*Macropygia ruficeps*	1.0	1	HBW4
Columbiformes	Columbidae	*Macropygia nigrirostris*	1.0	1	HBW4
Columbiformes	Columbidae	*Macropygia mackinlayi*	1.4	1	HBW4
Columbiformes	Columbidae	*Turtur abyssinicus*	2.0	1	HBW4
Columbiformes	Columbidae	*Turtur chalcospilos*	2.0	1	HBW4
Columbiformes	Columbidae	*Turtur afer*	2.0	1	HBW4
Columbiformes	Columbidae	*Turtur tympanistria*	2.0	1	HBW4
Columbiformes	Columbidae	*Turtur brehmeri*	1.4	1	HBW4
Columbiformes	Columbidae	*Oena capensis*	2.0	1	HBW4
Columbiformes	Columbidae	*Chalcophaps stephani*	2.0	1	HBW4
Columbiformes	Columbidae	*Phaps chalcoptera*	2.0	1	HBW4
Columbiformes	Columbidae	*Phaps elegans*	2.0	1	HBW4
Columbiformes	Columbidae	*Phaps histrionica*	2.0	1	HBW4
Columbiformes	Columbidae	*Geophaps lophotes*	2.0	1	HBW4
Columbiformes	Columbidae	*Geophaps plumifera*	2.0	1	HBW4
Columbiformes	Columbidae	*Geophaps smithii*	2.0	1	HBW4
Columbiformes	Columbidae	*Geophaps scripta*	2.0	1	HBW4
Columbiformes	Columbidae	*Petrophassa albipennis*	2.0	1	HBW4
Columbiformes	Columbidae	*Petrophassa rufipennis*	2.0	1	HBW4
Columbiformes	Columbidae	*Geopelia cuneata*	2.0	1	HBW4
Columbiformes	Columbidae	*Geopelia striata*	2.0	1	HBW4
Columbiformes	Columbidae	*Geopelia humeralis*	2.0	1	HBW4
Columbiformes	Columbidae	*Leucosarcia melanoleuca*	2.0	1	HBW4
Columbiformes	Columbidae	*Zenaida macroura*	2.0	1	HBW4
Columbiformes	Columbidae	*Zenaida auriculata*	2.0	1	HBW4

Order	Family	*Species*	Clutch	Range	Traits
Columbiformes	Columbidae	*Zenaida aurita*	2.0	1	HBW4
Columbiformes	Columbidae	*Zenaida asiatica*	2.0	1	HBW4
Columbiformes	Columbidae	*Zenaida galapagoensis*	2.0	1	HBW4
Columbiformes	Columbidae	*Columbina inca*	2.0	1	HBW4
Columbiformes	Columbidae	*Columbina squammata*	2.0	1	HBW4
Columbiformes	Columbidae	*Columbina passerina*	2.0	1	HBW4
Columbiformes	Columbidae	*Columbina minuta*	2.0	1	HBW4
Columbiformes	Columbidae	*Columbina talpacoti*	2.0	1	HBW4
Columbiformes	Columbidae	*Columbina picui*	2.0	1	HBW4
Columbiformes	Columbidae	*Columbina cruziana*	2.0	1	HBW4
Columbiformes	Columbidae	*Claravis pretiosa*	2.0	1	HBW4
Columbiformes	Columbidae	*Claravis godefrida*	2.0	1	HBW4
Columbiformes	Columbidae	*Leptotila verreauxi*	2.0	1	HBW4
Columbiformes	Columbidae	*Leptotila megalura*	2.0	1	HBW4
Columbiformes	Columbidae	*Leptotila rufaxilla*	1.4	1	HBW4
Columbiformes	Columbidae	*Leptotila jamaicensis*	2.0	1	HBW4
Columbiformes	Columbidae	*Leptotila cassini*	2.0	1	HBW4
Columbiformes	Columbidae	*Geotrygon lawrencii*	1.0	1	HBW4
Columbiformes	Columbidae	*Geotrygon carrikeri*	1.0	1	HBW4
Columbiformes	Columbidae	*Geotrygon caniceps*	1.4	1	HBW4
Columbiformes	Columbidae	*Geotrygon versicolor*	2.0	1	HBW4
Columbiformes	Columbidae	*Geotrygon veraguensis*	2.0	1	HBW4
Columbiformes	Columbidae	*Geotrygon albifacies*	1.4	1	HBW4
Columbiformes	Columbidae	*Geotrygon chiriquensis*	2.0	1	HBW4
Columbiformes	Columbidae	*Geotrygon linearis*	2.0	1	HBW4
Columbiformes	Columbidae	*Geotrygon frenata*	1.0	1	HBW4
Columbiformes	Columbidae	*Geotrygon chrysia*	2.0	1	HBW4
Columbiformes	Columbidae	*Geotrygon mystacea*	1.4	1	HBW4
Columbiformes	Columbidae	*Geotrygon violacea*	2.0	1	HBW4
Columbiformes	Columbidae	*Geotrygon montana*	2.0	1	HBW4
Columbiformes	Columbidae	*Caloenas nicobarica*	1.0	1	HBW4
Columbiformes	Columbidae	*Gallicolumba luzonica*	2.0	1	HBW4
Columbiformes	Columbidae	*Gallicolumba platenae*	2.0	1	HBW4
Columbiformes	Columbidae	*Gallicolumba criniger*	1.0	1	HBW4
Columbiformes	Columbidae	*Gallicolumba rufigula*	1.0	1	HBW4
Columbiformes	Columbidae	*Gallicolumba jobiensis*	2.0	1	HBW4
Columbiformes	Columbidae	*Gallicolumba stairi*	1.4	1	HBW4
Columbiformes	Columbidae	*Gallicolumba sanctaecrucis*	1.0	1	HBW4
Columbiformes	Columbidae	*Gallicolumba beccarii*	1.0	1	HBW4
Columbiformes	Columbidae	*Trugon terrestris*	1.0	1	HBW4
Columbiformes	Columbidae	*Otidiphaps nobilis*	1.0	1	HBW4
Columbiformes	Columbidae	*Treron fulvicollis*	2.0	1	HBW4
Columbiformes	Columbidae	*Treron griseicauda*	2.0	1	HBW4
Columbiformes	Columbidae	*Treron phoenicoptera*	2.0	1	HBW4
Columbiformes	Columbidae	*Treron waalia*	1.4	1	HBW4
Columbiformes	Columbidae	*Treron calva*	2.0	1	HBW4
Columbiformes	Columbidae	*Treron australis*	2.0	1	HBW4
Columbiformes	Columbidae	*Treron apicauda*	2.0	1	HBW4
Columbiformes	Columbidae	*Treron sphenura*	1.4	1	HBW4
Columbiformes	Columbidae	*Treron sieboldii*	2.0	1	HBW4
Columbiformes	Columbidae	*Treron formosae*	2.0	1	HBW4
Columbiformes	Columbidae	*Ptilinopus fischeri*	1.0	1	HBW4
Columbiformes	Columbidae	*Ptilinopus jambu*	1.0	1	HBW4
Columbiformes	Columbidae	*Ptilinopus leclancheri*	1.0	1	HBW4
Columbiformes	Columbidae	*Ptilinopus bernsteinii*	1.0	1	HBW4
Columbiformes	Columbidae	*Ptilinopus magnificus*	1.0	1	HBW4
Columbiformes	Columbidae	*Ptilinopus perlatus*	1.0	1	HBW4
Columbiformes	Columbidae	*Ptilinopus tannensis*	1.0	1	HBW4
Columbiformes	Columbidae	*Ptilinopus aurantiifrons*	1.0	1	HBW4
Columbiformes	Columbidae	*Ptilinopus superbus*	1.0	1	HBW4
Columbiformes	Columbidae	*Ptilinopus perousii*	1.4	1	HBW4
Columbiformes	Columbidae	*Ptilinopus coronulatus*	1.0	1	HBW4
Columbiformes	Columbidae	*Ptilinopus pulchellus*	1.0	1	HBW4

Order	Family	*Species*	Clutch	Range	Traits
Columbiformes	Columbidae	*Ptilinopus regina*	1.0	1	HBW4
Columbiformes	Columbidae	*Ptilinopus greyii*	1.0	1	HBW4
Columbiformes	Columbidae	*Ptilinopus rivoli*	2.0	1	HBW4
Columbiformes	Columbidae	*Ptilinopus solomonensis*	1.0	1	HBW4
Columbiformes	Columbidae	*Ptilinopus viridis*	1.0	1	HBW4
Columbiformes	Columbidae	*Ptilinopus iozonus*	1.0	1	HBW4
Columbiformes	Columbidae	*Ptilinopus insolitus*	1.0	1	HBW4
Columbiformes	Columbidae	*Ptilinopus melanospila*	1.0	1	HBW4
Columbiformes	Columbidae	*Ptilinopus victor*	1.0	1	HBW4
Columbiformes	Columbidae	*Ptilinopus luteovirens*	1.0	1	HBW4
Columbiformes	Columbidae	*Alectroenas madagascariensis*	1.0	1	HBW4
Columbiformes	Columbidae	*Ducula radiata*	1.0	1	HBW4
Columbiformes	Columbidae	*Ducula carola*	1.0	1	HBW4
Columbiformes	Columbidae	*Ducula aenea*	1.4	1	HBW4
Columbiformes	Columbidae	*Ducula pacifica*	1.0	1	HBW4
Columbiformes	Columbidae	*Ducula rubricera*	1.0	1	HBW4
Columbiformes	Columbidae	*Ducula pistrinaria*	1.0	1	HBW4
Columbiformes	Columbidae	*Ducula latrans*	1.0	1	HBW4
Columbiformes	Columbidae	*Ducula pinon*	1.0	1	HBW4
Columbiformes	Columbidae	*Ducula melanochroa*	1.0	1	HBW4
Columbiformes	Columbidae	*Ducula mullerii*	1.0	1	HBW4
Columbiformes	Columbidae	*Ducula badia*	1.0	1	HBW4
Columbiformes	Columbidae	*Ducula lacernulata*	1.0	1	HBW4
Columbiformes	Columbidae	*Ducula bicolor*	1.0	1	HBW4
Columbiformes	Columbidae	*Ducula spilorrhoa*	1.0	1	HBW4
Columbiformes	Columbidae	*Lopholaimus antarcticus*	1.0	1	HBW4
Columbiformes	Columbidae	*Gymnophaps albertisii*	1.0	1	HBW4
Gruiformes	Eurypygidae	*Eurypyga helias*	2.0	1	HBW3
Gruiformes	Otididae	*Tetrax tetrax*	3.5	1	HBW3
Gruiformes	Otididae	*Otis tarda*	2.4	1	HBW3
Gruiformes	Otididae	*Neotis denhami*	1.4	1	HBW3
Gruiformes	Otididae	*Neotis ludwigii*	2.0	1	HBW3
Gruiformes	Otididae	*Neotis nuba*	2.0	1	HBW3
Gruiformes	Otididae	*Neotis heuglinii*	2.0	1	HBW3
Gruiformes	Otididae	*Ardeotis arabs*	1.4	1	HBW3
Gruiformes	Otididae	*Ardeotis kori*	2.0	1	HBW3
Gruiformes	Otididae	*Ardeotis nigriceps*	1.0	1	HBW3
Gruiformes	Otididae	*Ardeotis australis*	1.4	1	HBW3
Gruiformes	Otididae	*Chlamydotis undulata*	2.4	1	HBW3
Gruiformes	Otididae	*Eupodotis savilei*	1.4	1	HBW3
Gruiformes	Otididae	*Eupodotis gindiana*	1.4	1	HBW3
Gruiformes	Otididae	*Eupodotis ruficrista*	1.4	1	HBW3
Gruiformes	Otididae	*Eupodotis afraoides*	1.4	1	HBW3
Gruiformes	Otididae	*Eupodotis afra*	1.4	1	HBW3
Gruiformes	Otididae	*Eupodotis rueppellii*	1.0	1	HBW3
Gruiformes	Otididae	*Eupodotis vigorsii*	1.0	1	HBW3
Gruiformes	Otididae	*Eupodotis humilis*	2.0	1	HBW3
Gruiformes	Otididae	*Eupodotis senegalensis*	1.7	1	HBW3
Gruiformes	Otididae	*Eupodotis caerulescens*	1.7	1	HBW3
Gruiformes	Otididae	*Eupodotis melanogaster*	1.4	1	HBW3
Gruiformes	Otididae	*Eupodotis bengalensis*	1.4	1	HBW3
Gruiformes	Otididae	*Eupodotis indica*	3.9	1	HBW3
Gruiformes	Gruidae	*Balearica pavonina*	2.4	1	HBW3
Gruiformes	Gruidae	*Balearica regulorum*	2.0	1	HBW3
Gruiformes	Gruidae	*Grus leucogeranus*	2.0	1	HBW3
Gruiformes	Gruidae	*Grus antigone*	2.4	1	HBW3
Gruiformes	Gruidae	*Grus rubicunda*	2.0	1	HBW3
Gruiformes	Gruidae	*Grus vipio*	2.0	1	HBW3
Gruiformes	Gruidae	*Grus canadensis*	2.0	1	HBW3
Gruiformes	Gruidae	*Grus virgo*	2.0	1	HBW3
Gruiformes	Gruidae	*Grus paradisea*	2.0	1	HBW3
Gruiformes	Gruidae	*Grus carunculatus*	2.0	1	HBW3
Gruiformes	Gruidae	*Grus grus*	2.0	1	HBW3

Order	Family	*Species*	Clutch	Range	Traits
Gruiformes	Gruidae	*Grus monacha*	2.0	1	HBW3
Gruiformes	Gruidae	*Grus americana*	2.0	1	HBW3
Gruiformes	Gruidae	*Grus nigricollis*	2.0	1	HBW3
Gruiformes	Gruidae	*Grus japonensis*	2.0	1	HBW3
Gruiformes	Heliornithidae	*Podica senegalensis*	2.4	1	HBW3
Gruiformes	Heliornithidae	*Heliopais personata*	5.9	1	HBW3
Gruiformes	Heliornithidae	*Heliornis fulica*	2.4	1	HBW3
Gruiformes	Psophiidae	*Psophia crepitans*	3.0	1	HBW3
Gruiformes	Psophiidae	*Psophia leucoptera*	3.0	1	HBW3
Gruiformes	Cariamidae	*Cariama cristata*	2.4	1	HBW3
Gruiformes	Rallidae	*Himantornis haematopus*	3.0	1	HBW3
Gruiformes	Rallidae	*Canirallus oculeus*	2.0	1	HBW3
Gruiformes	Rallidae	*Canirallus kioloides*	2.0	1	HBW3
Gruiformes	Rallidae	*Sarothrura pulchra*	2.0	1	HBW3
Gruiformes	Rallidae	*Sarothrura elegans*	3.9	1	HBW3
Gruiformes	Rallidae	*Sarothrura rufa*	2.4	1	HBW3
Gruiformes	Rallidae	*Sarothrura boehmi*	3.9	1	HBW3
Gruiformes	Rallidae	*Sarothrura affinis*	4.0	1	HBW3
Gruiformes	Rallidae	*Sarothrura insularis*	3.5	1	HBW3
Gruiformes	Rallidae	*Sarothrura ayresi*	3.0	1	HBW3
Gruiformes	Rallidae	*Coturnicops exquisitus*	3.5	1	HBW3
Gruiformes	Rallidae	*Coturnicops noveboracensis*	8.0	1	HBW3
Gruiformes	Rallidae	*Micropygia schomburgkii*	2.0	1	HBW3
Gruiformes	Rallidae	*Rallina rubra*	1.0	1	HBW3
Gruiformes	Rallidae	*Rallina forbesi*	4.5	1	HBW3
Gruiformes	Rallidae	*Rallina tricolor*	4.6	1	HBW3
Gruiformes	Rallidae	*Rallina canningi*	3.9	1	HBW3
Gruiformes	Rallidae	*Rallina fasciata*	4.2	1	HBW3
Gruiformes	Rallidae	*Rallina eurizonoides*	5.7	1	HBW3
Gruiformes	Rallidae	*Anurolimnas viridis*	1.7	1	HBW3
Gruiformes	Rallidae	*Laterallus melanophaius*	2.4	1	HBW3
Gruiformes	Rallidae	*Laterallus ruber*	4.2	1	HBW3
Gruiformes	Rallidae	*Laterallus albigularis*	3.2	1	HBW3
Gruiformes	Rallidae	*Laterallus exilis*	3.0	1	HBW3
Gruiformes	Rallidae	*Laterallus jamaicensis*	7.2	1	HBW3
Gruiformes	Rallidae	*Laterallus spilonotus*	4.2	1	HBW3
Gruiformes	Rallidae	*Laterallus leucopyrrhus*	2.4	1	HBW3
Gruiformes	Rallidae	*Gallirallus australis*	2.8	1	HBW3
Gruiformes	Rallidae	*Gallirallus torquatus*	3.5	1	HBW3
Gruiformes	Rallidae	*Gallirallus philippensis*	5.7	1	HBW3
Gruiformes	Rallidae	*Gallirallus rovianae*	2.4	1	HBW3
Gruiformes	Rallidae	*Gallirallus striatus*	6.7	1	HBW3
Gruiformes	Rallidae	*Rallus longirostris*	6.5	1	HBW3
Gruiformes	Rallidae	*Rallus elegans*	11.0	1	HBW3
Gruiformes	Rallidae	*Rallus limicola*	7.2	1	HBW3
Gruiformes	Rallidae	*Rallus antarcticus*	8.0	1	HBW3
Gruiformes	Rallidae	*Rallus aquaticus*	8.1	1	HBW3
Gruiformes	Rallidae	*Rallus caerulescens*	3.5	1	HBW3
Gruiformes	Rallidae	*Dryolimnas cuvieri*	3.5	1	HBW3
Gruiformes	Rallidae	*Crecopsis egregia*	5.2	1	HBW3
Gruiformes	Rallidae	*Crex crex*	9.8	1	HBW3
Gruiformes	Rallidae	*Rougetius rougetii*	4.5	1	HBW3
Gruiformes	Rallidae	*Aramides axillaris*	5.0	1	HBW3
Gruiformes	Rallidae	*Aramides cajanea*	4.6	1	HBW3
Gruiformes	Rallidae	*Aramides ypecaha*	4.5	1	HBW3
Gruiformes	Rallidae	*Aramides saracura*	4.5	1	HBW3
Gruiformes	Rallidae	*Amaurolimnas concolor*	4.0	1	HBW3
Gruiformes	Rallidae	*Amaurornis akool*	5.5	1	HBW3
Gruiformes	Rallidae	*Amaurornis moluccanus*	5.3	1	HBW3
Gruiformes	Rallidae	*Amaurornis phoenicurus*	6.0	1	HBW3
Gruiformes	Rallidae	*Amaurornis flavirostra*	3.0	1	HBW3
Gruiformes	Rallidae	*Amaurornis olivieri*	2.0	1	HBW3
Gruiformes	Rallidae	*Amaurornis bicolor*	6.3	1	HBW3

Order	Family	*Species*	Clutch	Range	Traits
Gruiformes	Rallidae	*Porzana parva*	7.9	1	HBW3
Gruiformes	Rallidae	*Porzana pusilla*	6.6	1	HBW3
Gruiformes	Rallidae	*Porzana porzana*	9.8	1	HBW3
Gruiformes	Rallidae	*Porzana fluminea*	4.6	1	HBW3
Gruiformes	Rallidae	*Porzana carolina*	11.0	1	HBW3
Gruiformes	Rallidae	*Porzana albicollis*	2.4	1	HBW3
Gruiformes	Rallidae	*Porzana fusca*	5.2	1	HBW3
Gruiformes	Rallidae	*Porzana paykullii*	6.7	1	HBW3
Gruiformes	Rallidae	*Porzana tabuensis*	3.5	1	HBW3
Gruiformes	Rallidae	*Porzana flaviventer*	3.9	1	HBW3
Gruiformes	Rallidae	*Porzana cinerea*	4.6	1	HBW3
Gruiformes	Rallidae	*Aenigmatolimnas marginalis*	4.5	1	HBW3
Gruiformes	Rallidae	*Neocrex erythrops*	4.6	1	HBW3
Gruiformes	Rallidae	*Pardirallus maculatus*	3.7	1	HBW3
Gruiformes	Rallidae	*Pardirallus nigricans*	3.0	1	HBW3
Gruiformes	Rallidae	*Pardirallus sanguinolentus*	4.9	1	HBW3
Gruiformes	Rallidae	*Eulabeornis castaneoventris*	4.5	1	HBW3
Gruiformes	Rallidae	*Gallicrex cinerea*	4.2	1	HBW3
Gruiformes	Rallidae	*Porphyrio porphyrio*	3.7	1	HBW3
Gruiformes	Rallidae	*Porphyrio mantelli*	2.0	1	HBW3
Gruiformes	Rallidae	*Porphyrio alleni*	4.9	1	HBW3
Gruiformes	Rallidae	*Porphyrio martinicus*	6.0	1	HBW3
Gruiformes	Rallidae	*Porphyrio flavirostris*	4.5	1	HBW3
Gruiformes	Rallidae	*Gallinula chloropus*	5.8	1	HBW3
Gruiformes	Rallidae	*Gallinula tenebrosa*	9.5	1	HBW3
Gruiformes	Rallidae	*Gallinula angulata*	5.2	1	HBW3
Gruiformes	Rallidae	*Gallinula melanops*	5.7	1	HBW3
Gruiformes	Rallidae	*Gallinula ventralis*	5.9	1	HBW3
Gruiformes	Rallidae	*Gallinula mortierii*	6.3	1	HBW3
Gruiformes	Rallidae	*Fulica cristata*	5.9	1	HBW3
Gruiformes	Rallidae	*Fulica atra*	7.7	1	HBW3
Gruiformes	Rallidae	*Fulica alai*	7.7	1	HBW3
Gruiformes	Rallidae	*Fulica americana*	9.5	1	HBW3
Gruiformes	Rallidae	*Fulica caribaea*	5.3	1	HBW3
Gruiformes	Rallidae	*Fulica leucoptera*	11.0	1	HBW3
Gruiformes	Rallidae	*Fulica ardesiaca*	4.5	1	HBW3
Gruiformes	Rallidae	*Fulica armillata*	4.0	1	HBW3
Gruiformes	Rallidae	*Fulica rufifrons*	4.2	1	HBW3
Gruiformes	Rallidae	*Fulica gigantea*	4.6	1	HBW3
Gruiformes	Rallidae	*Fulica cornuta*	3.9	1	HBW3
Gruiformes	Mesitornithidae	*Mesitornis variegata*	1.7	1	HBW3
Gruiformes	Mesitornithidae	*Mesitornis unicolor*	1.0	1	HBW3
Gruiformes	Mesitornithidae	*Monias benschi*	1.4	1	HBW3
Ciconiiformes	Pteroclidae	*Syrrhaptes tibetanus*	3.0	1	HBW4
Ciconiiformes	Pteroclidae	*Syrrhaptes paradoxus*	3.0	1	HBW4
Ciconiiformes	Pteroclidae	*Pterocles alchata*	3.0	1	HBW4
Ciconiiformes	Pteroclidae	*Pterocles namaqua*	3.0	1	HBW4
Ciconiiformes	Pteroclidae	*Pterocles exustus*	3.0	1	HBW4
Ciconiiformes	Pteroclidae	*Pterocles senegallus*	3.0	1	HBW4
Ciconiiformes	Pteroclidae	*Pterocles gutturalis*	3.0	1	HBW4
Ciconiiformes	Pteroclidae	*Pterocles orientalis*	3.0	1	HBW4
Ciconiiformes	Pteroclidae	*Pterocles coronatus*	3.0	1	HBW4
Ciconiiformes	Pteroclidae	*Pterocles personatus*	3.0	1	HBW4
Ciconiiformes	Pteroclidae	*Pterocles decoratus*	3.0	1	HBW4
Ciconiiformes	Pteroclidae	*Pterocles bicinctus*	2.4	1	HBW4
Ciconiiformes	Pteroclidae	*Pterocles quadricinctus*	2.4	1	HBW4
Ciconiiformes	Pteroclidae	*Pterocles indicus*	2.4	1	HBW4
Ciconiiformes	Pteroclidae	*Pterocles lichtensteinii*	2.4	1	HBW4
Ciconiiformes	Pteroclidae	*Pterocles burchelli*	3.0	1	HBW4
Ciconiiformes	Thinocoridae	*Attagis gayi*	4.0	1	HBW3
Ciconiiformes	Thinocoridae	*Attagis malouinus*	4.0	1	HBW3
Ciconiiformes	Thinocoridae	*Thinocorus orbignyianus*	4.0	1	HBW3
Ciconiiformes	Thinocoridae	*Thinocorus rumicivorus*	4.0	1	HBW3

Order	Family	*Species*	Clutch	Range	Traits
Ciconiiformes	Pedionomidae	*Pedionomus torquatus*	4.0	1	HBW3
Ciconiiformes	Scolopacidae	*Scolopax rusticola*	4.0	1	HBW3
Ciconiiformes	Scolopacidae	*Scolopax saturata*	2.0	1	HBW3
Ciconiiformes	Scolopacidae	*Scolopax minor*	4.0	1	HBW3
Ciconiiformes	Scolopacidae	*Gallinago solitaria*	4.0	1	HBW3
Ciconiiformes	Scolopacidae	*Gallinago hardwickii*	4.0	1	HBW3
Ciconiiformes	Scolopacidae	*Gallinago stenura*	4.0	1	HBW3
Ciconiiformes	Scolopacidae	*Gallinago megala*	4.0	1	HBW3
Ciconiiformes	Scolopacidae	*Gallinago media*	4.0	1	HBW3
Ciconiiformes	Scolopacidae	*Gallinago gallinago*	4.0	1	HBW3
Ciconiiformes	Scolopacidae	*Gallinago nigripennis*	2.4	1	HBW3
Ciconiiformes	Scolopacidae	*Gallinago macrodactyla*	3.0	1	HBW3
Ciconiiformes	Scolopacidae	*Gallinago paraguaiae*	2.8	1	HBW3
Ciconiiformes	Scolopacidae	*Gallinago nobilis*	2.0	1	HBW3
Ciconiiformes	Scolopacidae	*Gallinago undulata*	2.8	1	HBW3
Ciconiiformes	Scolopacidae	*Gallinago jamesoni*	2.0	1	HBW3
Ciconiiformes	Scolopacidae	*Gallinago stricklandii*	2.0	1	HBW3
Ciconiiformes	Scolopacidae	*Limosa limosa*	4.0	1	HBW3
Ciconiiformes	Scolopacidae	*Limosa haemastica*	4.0	1	HBW3
Ciconiiformes	Scolopacidae	*Limosa lapponica*	4.0	1	HBW3
Ciconiiformes	Scolopacidae	*Limosa fedoa*	4.0	1	HBW3
Ciconiiformes	Scolopacidae	*Numenius minutus*	4.0	1	HBW3
Ciconiiformes	Scolopacidae	*Numenius borealis*	3.5	1	HBW3
Ciconiiformes	Scolopacidae	*Numenius phaeopus*	4.0	1	HBW3
Ciconiiformes	Scolopacidae	*Numenius tahitiensis*	4.0	1	HBW3
Ciconiiformes	Scolopacidae	*Numenius tenuirostris*	4.0	1	HBW3
Ciconiiformes	Scolopacidae	*Numenius arquata*	4.0	1	HBW3
Ciconiiformes	Scolopacidae	*Numenius americanus*	4.0	1	HBW3
Ciconiiformes	Scolopacidae	*Numenius madagascariensis*	4.0	1	HBW3
Ciconiiformes	Scolopacidae	*Tringa erythropus*	4.0	1	HBW3
Ciconiiformes	Scolopacidae	*Tringa totanus*	4.0	1	HBW3
Ciconiiformes	Scolopacidae	*Tringa stagnatilis*	4.0	1	HBW3
Ciconiiformes	Scolopacidae	*Tringa nebularia*	4.0	1	HBW3
Ciconiiformes	Scolopacidae	*Tringa guttifer*	4.0	1	HBW3
Ciconiiformes	Scolopacidae	*Tringa melanoleuca*	4.0	1	HBW3
Ciconiiformes	Scolopacidae	*Tringa flavipes*	4.0	1	HBW3
Ciconiiformes	Scolopacidae	*Tringa solitaria*	4.0	1	HBW3
Ciconiiformes	Scolopacidae	*Tringa ochropus*	4.0	1	HBW3
Ciconiiformes	Scolopacidae	*Tringa glareola*	4.0	1	HBW3
Ciconiiformes	Scolopacidae	*Tringa cinerea*	4.0	1	HBW3
Ciconiiformes	Scolopacidae	*Tringa hypoleucos*	4.0	1	HBW3
Ciconiiformes	Scolopacidae	*Tringa macularia*	4.0	1	HBW3
Ciconiiformes	Scolopacidae	*Tringa brevipes*	4.0	1	HBW3
Ciconiiformes	Scolopacidae	*Tringa incana*	4.0	1	HBW3
Ciconiiformes	Scolopacidae	*Arenaria interpres*	4.0	1	HBW3
Ciconiiformes	Scolopacidae	*Arenaria melanocephala*	4.0	1	HBW3
Ciconiiformes	Scolopacidae	*Limnodromus griseus*	4.0	1	HBW3
Ciconiiformes	Scolopacidae	*Limnodromus scolopaceus*	4.0	1	HBW3
Ciconiiformes	Scolopacidae	*Limnodromus semipalmatus*	2.0	1	HBW3
Ciconiiformes	Scolopacidae	*Aphriza virgata*	4.0	1	HBW3
Ciconiiformes	Scolopacidae	*Calidris tenuirostris*	4.0	1	HBW3
Ciconiiformes	Scolopacidae	*Calidris canutus*	3.5	1	HBW3
Ciconiiformes	Scolopacidae	*Calidris alba*	4.0	1	HBW3
Ciconiiformes	Scolopacidae	*Calidris pusilla*	4.0	1	HBW3
Ciconiiformes	Scolopacidae	*Calidris mauri*	4.0	1	HBW3
Ciconiiformes	Scolopacidae	*Calidris minuta*	4.0	1	HBW3
Ciconiiformes	Scolopacidae	*Calidris ruficollis*	4.0	1	HBW3
Ciconiiformes	Scolopacidae	*Calidris temminckii*	4.0	1	HBW3
Ciconiiformes	Scolopacidae	*Calidris subminuta*	4.0	1	HBW3
Ciconiiformes	Scolopacidae	*Calidris minutilla*	4.0	1	HBW3
Ciconiiformes	Scolopacidae	*Calidris fuscicollis*	4.0	1	HBW3
Ciconiiformes	Scolopacidae	*Calidris bairdii*	4.0	1	HBW3
Ciconiiformes	Scolopacidae	*Calidris melanotos*	4.0	1	HBW3

Order	Family	*Species*	Clutch	Range	Traits
Ciconiiformes	Scolopacidae	*Calidris acuminata*	4.0	1	HBW3
Ciconiiformes	Scolopacidae	*Calidris maritima*	4.0	1	HBW3
Ciconiiformes	Scolopacidae	*Calidris ptilocnemis*	4.0	1	HBW3
Ciconiiformes	Scolopacidae	*Calidris alpina*	4.0	1	HBW3
Ciconiiformes	Scolopacidae	*Micropalama himantopus*	4.0	1	HBW3
Ciconiiformes	Scolopacidae	*Tryngites subruficollis*	3.5	1	HBW3
Ciconiiformes	Scolopacidae	*Limicola falcinellus*	4.0	1	HBW3
Ciconiiformes	Scolopacidae	*Philomachus pugnax*	4.0	1	HBW3
Ciconiiformes	Scolopacidae	*Phalaropus tricolor*	4.0	1	HBW3
Ciconiiformes	Scolopacidae	*Phalaropus lobatus*	4.0	1	HBW3
Ciconiiformes	Scolopacidae	*Phalaropus fulicaria*	4.0	1	HBW3
Ciconiiformes	Rostratulidae	*Rostratula benghalensis*	3.2	1	HBW3
Ciconiiformes	Rostratulidae	*Rostratula semicollaris*	2.0	1	HBW3
Ciconiiformes	Jacanidae	*Actophilornis africanus*	4.0	1	HBW3
Ciconiiformes	Jacanidae	*Actophilornis albinucha*	4.0	1	HBW3
Ciconiiformes	Jacanidae	*Microparra capensis*	3.0	1	HBW3
Ciconiiformes	Jacanidae	*Irediparra gallinacea*	4.0	1	HBW3
Ciconiiformes	Jacanidae	*Hydrophasianus chirurgus*	4.0	1	HBW3
Ciconiiformes	Jacanidae	*Metopidius indicus*	4.0	1	HBW3
Ciconiiformes	Jacanidae	*Jacana spinosa*	4.0	1	HBW3
Ciconiiformes	Jacanidae	*Jacana jacana*	4.0	1	HBW3
Ciconiiformes	Pluvianellidae	*Pluvianellus socialis*	2.0	1	HBW3
Ciconiiformes	Burhinidae	*Burhinus oedicnemus*	2.0	1	HBW3
Ciconiiformes	Burhinidae	*Burhinus senegalensis*	2.0	1	HBW3
Ciconiiformes	Burhinidae	*Burhinus vermiculatus*	2.0	1	HBW3
Ciconiiformes	Burhinidae	*Burhinus capensis*	2.0	1	HBW3
Ciconiiformes	Burhinidae	*Burhinus bistriatus*	2.0	1	HBW3
Ciconiiformes	Burhinidae	*Burhinus superciliaris*	2.0	1	HBW3
Ciconiiformes	Burhinidae	*Burhinus grallarius*	2.0	1	HBW3
Ciconiiformes	Burhinidae	*Burhinus recurvirostris*	1.4	1	HBW3
Ciconiiformes	Burhinidae	*Esacus neglectus*	1.0	1	HBW3
Ciconiiformes	Charadriidae	*Haematopus ostralegus*	3.2	1	HBW3
Ciconiiformes	Charadriidae	*Ibidorhyncha struthersii*	4.0	1	HBW3
Ciconiiformes	Charadriidae	*Cladorhynchus leucocephalus*	3.5	1	HBW3
Ciconiiformes	Charadriidae	*Recurvirostra avosetta*	3.5	1	HBW3
Ciconiiformes	Charadriidae	*Recurvirostra americana*	4.0	1	HBW3
Ciconiiformes	Charadriidae	*Recurvirostra novaehollandiae*	4.0	1	HBW3
Ciconiiformes	Charadriidae	*Recurvirostra andina*	4.0	1	HBW3
Ciconiiformes	Charadriidae	*Pluvialis apricaria*	4.0	1	HBW3
Ciconiiformes	Charadriidae	*Pluvialis fulva*	4.0	1	HBW3
Ciconiiformes	Charadriidae	*Pluvialis dominica*	4.0	1	HBW3
Ciconiiformes	Charadriidae	*Pluvialis squatarola*	4.0	1	HBW3
Ciconiiformes	Charadriidae	*Charadrius obscurus*	3.0	1	HBW3
Ciconiiformes	Charadriidae	*Charadrius hiaticula*	3.5	1	HBW3
Ciconiiformes	Charadriidae	*Charadrius semipalmatus*	3.5	1	HBW3
Ciconiiformes	Charadriidae	*Charadrius placidus*	4.0	1	HBW3
Ciconiiformes	Charadriidae	*Charadrius dubius*	4.0	1	HBW3
Ciconiiformes	Charadriidae	*Charadrius wilsonia*	3.0	1	HBW3
Ciconiiformes	Charadriidae	*Charadrius vociferus*	4.0	1	HBW3
Ciconiiformes	Charadriidae	*Charadrius thoracicus*	2.0	1	HBW3
Ciconiiformes	Charadriidae	*Charadrius pecuarius*	2.0	1	HBW3
Ciconiiformes	Charadriidae	*Charadrius tricollaris*	2.0	1	HBW3
Ciconiiformes	Charadriidae	*Charadrius forbesi*	2.4	1	HBW3
Ciconiiformes	Charadriidae	*Charadrius melodus*	4.0	1	HBW3
Ciconiiformes	Charadriidae	*Charadrius pallidus*	2.0	1	HBW3
Ciconiiformes	Charadriidae	*Charadrius alexandrinus*	3.0	1	HBW3
Ciconiiformes	Charadriidae	*Charadrius marginatus*	2.0	1	HBW3
Ciconiiformes	Charadriidae	*Charadrius ruficapillus*	2.0	1	HBW3
Ciconiiformes	Charadriidae	*Charadrius peronii*	3.0	1	HBW3
Ciconiiformes	Charadriidae	*Charadrius collaris*	2.0	1	HBW3
Ciconiiformes	Charadriidae	*Charadrius bicinctus*	3.0	1	HBW3
Ciconiiformes	Charadriidae	*Charadrius falklandicus*	3.0	1	HBW3
Ciconiiformes	Charadriidae	*Charadrius mongolus*	3.0	1	HBW3

Order	Family	*Species*	Clutch	Range	Traits
Ciconiiformes	Charadriidae	*Charadrius leschenaultii*	3.0	1	HBW3
Ciconiiformes	Charadriidae	*Charadrius asiaticus*	3.0	1	HBW3
Ciconiiformes	Charadriidae	*Charadrius montanus*	3.0	1	HBW3
Ciconiiformes	Charadriidae	*Charadrius modestus*	2.0	1	HBW3
Ciconiiformes	Charadriidae	*Charadrius australis*	3.0	1	HBW3
Ciconiiformes	Charadriidae	*Erythrogonys cinctus*	4.0	1	HBW3
Ciconiiformes	Charadriidae	*Eudromias morinellus*	3.0	1	HBW3
Ciconiiformes	Charadriidae	*Elseyornis melanops*	3.0	1	HBW3
Ciconiiformes	Charadriidae	*Vanellus vanellus*	4.0	1	HBW3
Ciconiiformes	Charadriidae	*Vanellus crassirostris*	2.8	1	HBW3
Ciconiiformes	Charadriidae	*Vanellus malarbaricus*	4.0	1	HBW3
Ciconiiformes	Charadriidae	*Vanellus tricolor*	4.0	1	HBW3
Ciconiiformes	Charadriidae	*Vanellus miles*	4.0	1	HBW3
Ciconiiformes	Charadriidae	*Vanellus armatus*	3.0	1	HBW3
Ciconiiformes	Charadriidae	*Vanellus spinosus*	2.8	1	HBW3
Ciconiiformes	Charadriidae	*Vanellus duvaucelii*	4.0	1	HBW3
Ciconiiformes	Charadriidae	*Vanellus tectus*	2.4	1	HBW3
Ciconiiformes	Charadriidae	*Vanellus melanocephalus*	4.0	1	HBW3
Ciconiiformes	Charadriidae	*Vanellus cinereus*	4.0	1	HBW3
Ciconiiformes	Charadriidae	*Vanellus indicus*	3.5	1	HBW3
Ciconiiformes	Charadriidae	*Vanellus albiceps*	2.8	1	HBW3
Ciconiiformes	Charadriidae	*Vanellus senegallus*	2.8	1	HBW3
Ciconiiformes	Charadriidae	*Vanellus lugubris*	3.0	1	HBW3
Ciconiiformes	Charadriidae	*Vanellus melanopterus*	3.0	1	HBW3
Ciconiiformes	Charadriidae	*Vanellus coronatus*	2.4	1	HBW3
Ciconiiformes	Charadriidae	*Vanellus superciliosus*	2.8	1	HBW3
Ciconiiformes	Charadriidae	*Vanellus gregarius*	3.2	1	HBW3
Ciconiiformes	Charadriidae	*Vanellus leucurus*	4.0	1	HBW3
Ciconiiformes	Charadriidae	*Vanellus cayanus*	2.0	1	HBW3
Ciconiiformes	Charadriidae	*Vanellus chilensis*	4.0	1	HBW3
Ciconiiformes	Charadriidae	*Vanellus resplendens*	4.0	1	HBW3
Ciconiiformes	Glareolidae	*Dromas ardeola*	1.0	1	HBW3
Ciconiiformes	Glareolidae	*Pluvianus aegyptius*	2.4	1	HBW3
Ciconiiformes	Glareolidae	*Rhinoptilus africanus*	1.0	1	HBW3
Ciconiiformes	Glareolidae	*Rhinoptilus chalcopterus*	2.4	1	HBW3
Ciconiiformes	Glareolidae	*Rhinoptilus cinctus*	2.0	1	HBW3
Ciconiiformes	Glareolidae	*Rhinoptilus bitorquatus*	2.0	1	HBW3
Ciconiiformes	Glareolidae	*Cursorius cursor*	2.0	1	HBW3
Ciconiiformes	Glareolidae	*Cursorius rufus*	2.0	1	HBW3
Ciconiiformes	Glareolidae	*Cursorius temminckii*	2.0	1	HBW3
Ciconiiformes	Glareolidae	*Cursorius coromandelicus*	2.4	1	HBW3
Ciconiiformes	Glareolidae	*Glareola pratincola*	3.0	1	HBW3
Ciconiiformes	Glareolidae	*Glareola maldivarum*	2.4	1	HBW3
Ciconiiformes	Glareolidae	*Glareola nordmanni*	3.5	1	HBW3
Ciconiiformes	Glareolidae	*Glareola ocularis*	2.0	1	HBW3
Ciconiiformes	Glareolidae	*Glareola nuchalis*	2.0	1	HBW3
Ciconiiformes	Glareolidae	*Glareola cinerea*	1.4	1	HBW3
Ciconiiformes	Glareolidae	*Glareola lactea*	2.4	1	HBW3
Ciconiiformes	Glareolidae	*Stiltia isabella*	2.0	1	HBW3
Ciconiiformes	Laridae	*Stercorarius longicaudus*	2.0	1	HBW3
Ciconiiformes	Laridae	*Rynchops niger*	3.5	1	HBW3
Ciconiiformes	Laridae	*Rynchops flavirostris*	2.4	1	HBW3
Ciconiiformes	Laridae	*Rynchops albicollis*	2.8	1	HBW3
Ciconiiformes	Laridae	*Larus modestus*	1.7	1	HBW3
Ciconiiformes	Laridae	*Larus delawarensis*	3.0	1	HBW3
Ciconiiformes	Laridae	*Larus californicus*	3.0	1	HBW3
Ciconiiformes	Laridae	*Larus cachinnans*	2.4	1	HBW3
Ciconiiformes	Laridae	*Larus armenicus*	3.0	1	HBW3
Ciconiiformes	Laridae	*Larus fuscus*	2.4	1	HBW3
Ciconiiformes	Laridae	*Larus ichthyaetus*	2.0	1	HBW3
Ciconiiformes	Laridae	*Larus brunnicephalus*	3.0	1	HBW3
Ciconiiformes	Laridae	*Larus cirrocephalus*	2.4	1	HBW3
Ciconiiformes	Laridae	*Larus novaehollandiae*	3.0	1	HBW3

Order	Family	*Species*	Clutch	Range	Traits
Ciconiiformes	Laridae	*Larus scopulinus*	2.0	1	HBW3
Ciconiiformes	Laridae	*Larus bulleri*	2.0	1	HBW3
Ciconiiformes	Laridae	*Larus maculipennis*	2.4	1	HBW3
Ciconiiformes	Laridae	*Larus ridibundus*	1.7	1	HBW3
Ciconiiformes	Laridae	*Larus genei*	2.4	1	HBW3
Ciconiiformes	Laridae	*Larus philadelphia*	3.0	1	HBW3
Ciconiiformes	Laridae	*Larus saundersi*	3.0	1	HBW3
Ciconiiformes	Laridae	*Larus serranus*	2.4	1	HBW3
Ciconiiformes	Laridae	*Larus melanocephalus*	2.4	1	HBW3
Ciconiiformes	Laridae	*Larus relictus*	2.4	1	HBW3
Ciconiiformes	Laridae	*Larus pipixcan*	3.0	1	HBW3
Ciconiiformes	Laridae	*Larus minutus*	2.4	1	HBW3
Ciconiiformes	Laridae	*Sterna aurantia*	3.0	1	HBW3
Ciconiiformes	Laridae	*Sterna hirundo*	2.0	1	HBW3
Ciconiiformes	Laridae	*Sterna paradisaea*	2.4	1	HBW3
Ciconiiformes	Laridae	*Sterna forsteri*	2.4	1	HBW3
Ciconiiformes	Laridae	*Sterna trudeaui*	3.0	1	HBW3
Ciconiiformes	Laridae	*Sterna superciliaris*	2.4	1	HBW3
Ciconiiformes	Laridae	*Sterna acuticauda*	3.0	1	HBW3
Ciconiiformes	Laridae	*Phaetusa simplex*	2.4	1	HBW3
Ciconiiformes	Accipitridae	*Pandion haliaetus*	3.0	1	HBW2
Ciconiiformes	Accipitridae	*Aviceda cuculoides*	2.0	1	HBW2
Ciconiiformes	Accipitridae	*Aviceda jerdoni*	2.4	1	HBW2
Ciconiiformes	Accipitridae	*Aviceda subcristata*	2.4	1	HBW2
Ciconiiformes	Accipitridae	*Aviceda leuphotes*	2.4	1	HBW2
Ciconiiformes	Accipitridae	*Chondrohierax uncinatus*	1.4	1	HBW2
Ciconiiformes	Accipitridae	*Pernis apivorus*	2.0	1	HBW2
Ciconiiformes	Accipitridae	*Pernis ptilorhyncus*	2.0	1	HBW2
Ciconiiformes	Accipitridae	*Pernis celebensis*	2.0	1	HBW2
Ciconiiformes	Accipitridae	*Lophoictinia isura*	3.0	1	HBW2
Ciconiiformes	Accipitridae	*Hamirostra melanosternon*	2.0	1	HBW2
Ciconiiformes	Accipitridae	*Elanoides forficatus*	1.4	1	HBW2
Ciconiiformes	Accipitridae	*Macheiramphus alcinus*	1.0	1	HBW2
Ciconiiformes	Accipitridae	*Gampsonyx swainsonii*	2.4	1	HBW2
Ciconiiformes	Accipitridae	*Elanus caeruleus*	3.5	1	HBW2
Ciconiiformes	Accipitridae	*Elanus axillaris*	3.5	1	HBW2
Ciconiiformes	Accipitridae	*Elanus leucurus*	3.9	1	HBW2
Ciconiiformes	Accipitridae	*Elanus scriptus*	4.5	1	HBW2
Ciconiiformes	Accipitridae	*Chelictinia riocourii*	4.0	1	HBW2
Ciconiiformes	Accipitridae	*Rostrhamus sociabilis*	2.4	1	HBW2
Ciconiiformes	Accipitridae	*Rostrhamus hamatus*	2.0	1	HBW2
Ciconiiformes	Accipitridae	*Harpagus bidentatus*	2.0	1	HBW2
Ciconiiformes	Accipitridae	*Harpagus diodon*	2.0	1	HBW2
Ciconiiformes	Accipitridae	*Ictinia mississippiensis*	2.0	1	HBW2
Ciconiiformes	Accipitridae	*Ictinia plumbea*	1.4	1	HBW2
Ciconiiformes	Accipitridae	*Milvus milvus*	2.0	1	HBW2
Ciconiiformes	Accipitridae	*Milvus migrans*	2.4	1	HBW2
Ciconiiformes	Accipitridae	*Haliastur sphenurus*	2.0	1	HBW2
Ciconiiformes	Accipitridae	*Haliastur indus*	1.4	1	HBW2
Ciconiiformes	Accipitridae	*Haliaeetus leucogaster*	2.0	1	HBW2
Ciconiiformes	Accipitridae	*Haliaeetus vocifer*	2.0	1	HBW2
Ciconiiformes	Accipitridae	*Haliaeetus vociferoides*	2.0	1	HBW2
Ciconiiformes	Accipitridae	*Haliaeetus leucoryphus*	2.4	1	HBW2
Ciconiiformes	Accipitridae	*Haliaeetus albicilla*	1.7	1	HBW2
Ciconiiformes	Accipitridae	*Haliaeetus leucocephalus*	2.0	1	HBW2
Ciconiiformes	Accipitridae	*Haliaeetus pelagicus*	1.7	1	HBW2
Ciconiiformes	Accipitridae	*Gypohierax angolensis*	1.0	1	HBW2
Ciconiiformes	Accipitridae	*Gypaetus barbatus*	1.4	1	HBW2
Ciconiiformes	Accipitridae	*Neophron percnopterus*	2.0	1	HBW2
Ciconiiformes	Accipitridae	*Necrosyrtes monachus*	1.0	1	HBW2
Ciconiiformes	Accipitridae	*Gyps africanus*	1.0	1	HBW2
Ciconiiformes	Accipitridae	*Gyps bengalensis*	1.0	1	HBW2
Ciconiiformes	Accipitridae	*Gyps indicus*	1.0	1	HBW2

Order	Family	*Species*	Clutch	Range	Traits
Ciconiiformes	Accipitridae	*Gyps rueppellii*	1.0	1	HBW2
Ciconiiformes	Accipitridae	*Gyps himalayensis*	1.0	1	HBW2
Ciconiiformes	Accipitridae	*Gyps fulvus*	1.0	1	HBW2
Ciconiiformes	Accipitridae	*Gyps coprotheres*	1.0	1	HBW2
Ciconiiformes	Accipitridae	*Aegypius monachus*	1.0	1	HBW2
Ciconiiformes	Accipitridae	*Torgos tracheliotus*	1.0	1	HBW2
Ciconiiformes	Accipitridae	*Trigonoceps occipitalis*	1.0	1	HBW2
Ciconiiformes	Accipitridae	*Circaetus gallicus*	1.0	1	HBW2
Ciconiiformes	Accipitridae	*Circaetus cinereus*	1.0	1	HBW2
Ciconiiformes	Accipitridae	*Circaetus fasciolatus*	1.0	1	HBW2
Ciconiiformes	Accipitridae	*Circaetus cinerascens*	1.0	1	HBW2
Ciconiiformes	Accipitridae	*Terathopius ecaudatus*	1.0	1	HBW2
Ciconiiformes	Accipitridae	*Circus aeruginosus*	4.2	1	HBW2
Ciconiiformes	Accipitridae	*Circus ranivorus*	3.5	1	HBW2
Ciconiiformes	Accipitridae	*Circus spilonotus*	4.6	1	HBW2
Ciconiiformes	Accipitridae	*Circus approximans*	3.5	1	HBW2
Ciconiiformes	Accipitridae	*Circus buffoni*	3.5	1	HBW2
Ciconiiformes	Accipitridae	*Circus assimilis*	3.0	1	HBW2
Ciconiiformes	Accipitridae	*Circus maurus*	3.5	1	HBW2
Ciconiiformes	Accipitridae	*Circus cyaneus*	4.2	1	HBW2
Ciconiiformes	Accipitridae	*Circus cinereus*	3.5	1	HBW2
Ciconiiformes	Accipitridae	*Circus macrourus*	4.5	1	HBW2
Ciconiiformes	Accipitridae	*Circus melanoleucos*	4.5	1	HBW2
Ciconiiformes	Accipitridae	*Circus pygargus*	3.9	1	HBW2
Ciconiiformes	Accipitridae	*Polyboroides typus*	2.0	1	HBW2
Ciconiiformes	Accipitridae	*Polyboroides radiatus*	2.0	1	HBW2
Ciconiiformes	Accipitridae	*Kaupifalco monogrammicus*	2.0	1	HBW2
Ciconiiformes	Accipitridae	*Melierax metabates*	1.4	1	HBW2
Ciconiiformes	Accipitridae	*Melierax poliopterus*	1.4	1	HBW2
Ciconiiformes	Accipitridae	*Melierax canorus*	2.0	1	HBW2
Ciconiiformes	Accipitridae	*Melierax gabar*	2.0	1	HBW2
Ciconiiformes	Accipitridae	*Accipiter trivirgatus*	2.0	1	HBW2
Ciconiiformes	Accipitridae	*Accipiter tachiro*	2.4	1	HBW2
Ciconiiformes	Accipitridae	*Accipiter badius*	2.8	1	HBW2
Ciconiiformes	Accipitridae	*Accipiter brevipes*	3.9	1	HBW2
Ciconiiformes	Accipitridae	*Accipiter soloensis*	3.5	1	HBW2
Ciconiiformes	Accipitridae	*Accipiter francesiae*	3.0	1	HBW2
Ciconiiformes	Accipitridae	*Accipiter novaehollandiae*	2.4	1	HBW2
Ciconiiformes	Accipitridae	*Accipiter fasciatus*	3.0	1	HBW2
Ciconiiformes	Accipitridae	*Accipiter haplochrous*	3.0	1	HBW2
Ciconiiformes	Accipitridae	*Accipiter rufitorques*	2.4	1	HBW2
Ciconiiformes	Accipitridae	*Accipiter poliocephalus*	1.0	1	HBW2
Ciconiiformes	Accipitridae	*Accipiter superciliosus*	1.7	1	HBW2
Ciconiiformes	Accipitridae	*Accipiter erythropus*	2.0	1	HBW2
Ciconiiformes	Accipitridae	*Accipiter minullus*	2.0	1	HBW2
Ciconiiformes	Accipitridae	*Accipiter gularis*	3.2	1	HBW2
Ciconiiformes	Accipitridae	*Accipiter cirrocephalus*	3.5	1	HBW2
Ciconiiformes	Accipitridae	*Accipiter madagascariensis*	3.0	1	HBW2
Ciconiiformes	Accipitridae	*Accipiter ovampensis*	3.0	1	HBW2
Ciconiiformes	Accipitridae	*Accipiter nisus*	4.2	1	HBW2
Ciconiiformes	Accipitridae	*Accipiter rufiventris*	3.0	1	HBW2
Ciconiiformes	Accipitridae	*Accipiter striatus*	4.5	1	HBW2
Ciconiiformes	Accipitridae	*Accipiter cooperii*	4.2	1	HBW2
Ciconiiformes	Accipitridae	*Accipiter gundlachi*	3.5	1	HBW2
Ciconiiformes	Accipitridae	*Accipiter bicolor*	1.7	1	HBW2
Ciconiiformes	Accipitridae	*Accipiter melanoleucus*	2.8	1	HBW2
Ciconiiformes	Accipitridae	*Accipiter gentilis*	3.5	1	HBW2
Ciconiiformes	Accipitridae	*Accipiter meyerianus*	3.0	1	HBW2
Ciconiiformes	Accipitridae	*Erythrotriorchis radiatus*	2.0	1	HBW2
Ciconiiformes	Accipitridae	*Butastur rufipennis*	1.7	1	HBW2
Ciconiiformes	Accipitridae	*Butastur teesa*	3.0	1	HBW2
Ciconiiformes	Accipitridae	*Butastur liventer*	2.4	1	HBW2
Ciconiiformes	Accipitridae	*Butastur indicus*	2.8	1	HBW2

Order	Family	Species	Clutch	Range	Traits
Ciconiiformes	Accipitridae	*Buteogallus aequinoctialis*	1.4	1	HBW2
Ciconiiformes	Accipitridae	*Buteogallus anthracinus*	1.7	1	HBW2
Ciconiiformes	Accipitridae	*Buteogallus urubitinga*	1.4	1	HBW2
Ciconiiformes	Accipitridae	*Buteogallus meridionalis*	1.0	1	HBW2
Ciconiiformes	Accipitridae	*Parabuteo unicinctus*	2.8	1	HBW2
Ciconiiformes	Accipitridae	*Buteo nitidus*	1.7	1	HBW2
Ciconiiformes	Accipitridae	*Buteo magnirostris*	1.4	1	HBW2
Ciconiiformes	Accipitridae	*Buteo lineatus*	3.2	1	HBW2
Ciconiiformes	Accipitridae	*Buteo ridgwayi*	2.0	1	HBW2
Ciconiiformes	Accipitridae	*Buteo platypterus*	2.4	1	HBW2
Ciconiiformes	Accipitridae	*Buteo leucorrhous*	2.4	1	HBW2
Ciconiiformes	Accipitridae	*Buteo brachyurus*	2.0	1	HBW2
Ciconiiformes	Accipitridae	*Buteo swainsoni*	2.4	1	HBW2
Ciconiiformes	Accipitridae	*Buteo galapagoensis*	1.7	1	HBW2
Ciconiiformes	Accipitridae	*Buteo albicaudatus*	2.0	1	HBW2
Ciconiiformes	Accipitridae	*Buteo polyosoma*	1.7	1	HBW2
Ciconiiformes	Accipitridae	*Buteo poecilochrous*	2.0	1	HBW2
Ciconiiformes	Accipitridae	*Buteo albonotatus*	2.0	1	HBW2
Ciconiiformes	Accipitridae	*Buteo solitarius*	2.4	1	HBW2
Ciconiiformes	Accipitridae	*Buteo jamaicensis*	2.4	1	HBW2
Ciconiiformes	Accipitridae	*Buteo buteo*	2.8	1	HBW2
Ciconiiformes	Accipitridae	*Buteo oreophilus*	2.4	1	HBW2
Ciconiiformes	Accipitridae	*Buteo brachypterus*	2.0	1	HBW2
Ciconiiformes	Accipitridae	*Buteo rufinus*	2.8	1	HBW2
Ciconiiformes	Accipitridae	*Buteo hemilasius*	2.8	1	HBW2
Ciconiiformes	Accipitridae	*Buteo regalis*	3.5	1	HBW2
Ciconiiformes	Accipitridae	*Buteo lagopus*	3.9	1	HBW2
Ciconiiformes	Accipitridae	*Buteo auguralis*	2.4	1	HBW2
Ciconiiformes	Accipitridae	*Buteo augur*	2.0	1	HBW2
Ciconiiformes	Accipitridae	*Buteo archeri*	1.4	1	HBW2
Ciconiiformes	Accipitridae	*Buteo rufofuscus*	2.0	1	HBW2
Ciconiiformes	Accipitridae	*Aquila pomarina*	2.0	1	HBW2
Ciconiiformes	Accipitridae	*Aquila clanga*	2.0	1	HBW2
Ciconiiformes	Accipitridae	*Aquila rapax*	2.0	1	HBW2
Ciconiiformes	Accipitridae	*Aquila nipalensis*	1.7	1	HBW2
Ciconiiformes	Accipitridae	*Aquila adalberti*	2.4	1	HBW2
Ciconiiformes	Accipitridae	*Aquila heliaca*	2.4	1	HBW2
Ciconiiformes	Accipitridae	*Aquila chrysaetos*	2.0	1	HBW2
Ciconiiformes	Accipitridae	*Aquila audax*	2.0	1	HBW2
Ciconiiformes	Accipitridae	*Aquila verreauxii*	2.0	1	HBW2
Ciconiiformes	Accipitridae	*Aquila wahlbergi*	1.0	1	HBW2
Ciconiiformes	Accipitridae	*Hieraaetus fasciatus*	1.4	1	HBW2
Ciconiiformes	Accipitridae	*Hieraaetus spilogaster*	2.0	1	HBW2
Ciconiiformes	Accipitridae	*Hieraaetus pennatus*	2.0	1	HBW2
Ciconiiformes	Accipitridae	*Hieraaetus morphnoides*	2.0	1	HBW2
Ciconiiformes	Accipitridae	*Hieraaetus ayresii*	1.0	1	HBW2
Ciconiiformes	Accipitridae	*Hieraaetus kienerii*	1.0	1	HBW2
Ciconiiformes	Accipitridae	*Polemaetus bellicosus*	1.0	1	HBW2
Ciconiiformes	Accipitridae	*Lophaetus occipitalis*	1.4	1	HBW2
Ciconiiformes	Accipitridae	*Stephanoaetus coronatus*	2.0	1	HBW2
Ciconiiformes	Sagittariidae	*Sagittarius serpentarius*	2.0	1	HBW2
Ciconiiformes	Falconidae	*Micrastur ruficollis*	2.4	1	HBW2
Ciconiiformes	Falconidae	*Micrastur semitorquatus*	2.4	1	HBW2
Ciconiiformes	Falconidae	*Caracara plancus*	2.0	1	HBW2
Ciconiiformes	Falconidae	*Polihierax semitorquatus*	3.0	1	HBW2
Ciconiiformes	Falconidae	*Microhierax caerulescens*	4.5	1	HBW2
Ciconiiformes	Falconidae	*Microhierax fringillarius*	4.5	1	HBW2
Ciconiiformes	Falconidae	*Falco berigora*	2.4	1	HBW2
Ciconiiformes	Falconidae	*Falco naumanni*	4.2	1	HBW2
Ciconiiformes	Falconidae	*Falco tinnunculus*	4.2	1	HBW2
Ciconiiformes	Falconidae	*Falco newtoni*	4.0	1	HBW2
Ciconiiformes	Falconidae	*Falco punctatus*	3.0	1	HBW2
Ciconiiformes	Falconidae	*Falco moluccensis*	4.0	1	HBW2

Order	Family	*Species*	Clutch	Range	Traits
Ciconiiformes	Falconidae	*Falco cenchroides*	3.9	1	HBW2
Ciconiiformes	Falconidae	*Falco sparverius*	4.9	1	HBW2
Ciconiiformes	Falconidae	*Falco rupicoloides*	3.5	1	HBW2
Ciconiiformes	Falconidae	*Falco alopex*	2.4	1	HBW2
Ciconiiformes	Falconidae	*Falco ardosiaceus*	3.9	1	HBW2
Ciconiiformes	Falconidae	*Falco dickinsoni*	3.0	1	HBW2
Ciconiiformes	Falconidae	*Falco chicquera*	3.5	1	HBW2
Ciconiiformes	Falconidae	*Falco vespertinus*	3.5	1	HBW2
Ciconiiformes	Falconidae	*Falco amurensis*	3.5	1	HBW2
Ciconiiformes	Falconidae	*Falco eleonorae*	2.4	1	HBW2
Ciconiiformes	Falconidae	*Falco concolor*	2.4	1	HBW2
Ciconiiformes	Falconidae	*Falco femoralis*	2.4	1	HBW2
Ciconiiformes	Falconidae	*Falco columbarius*	4.2	1	HBW2
Ciconiiformes	Falconidae	*Falco rufigularis*	2.8	1	HBW2
Ciconiiformes	Falconidae	*Falco deiroleucus*	1.7	1	HBW2
Ciconiiformes	Falconidae	*Falco subbuteo*	3.0	1	HBW2
Ciconiiformes	Falconidae	*Falco cuvierii*	3.0	1	HBW2
Ciconiiformes	Falconidae	*Falco severus*	2.8	1	HBW2
Ciconiiformes	Falconidae	*Falco longipennis*	2.4	1	HBW2
Ciconiiformes	Falconidae	*Falco novaeseelandiae*	2.8	1	HBW2
Ciconiiformes	Falconidae	*Falco hypoleucos*	2.4	1	HBW2
Ciconiiformes	Falconidae	*Falco subniger*	3.5	1	HBW2
Ciconiiformes	Falconidae	*Falco biarmicus*	3.5	1	HBW2
Ciconiiformes	Falconidae	*Falco jugger*	3.5	1	HBW2
Ciconiiformes	Falconidae	*Falco cherrug*	3.9	1	HBW2
Ciconiiformes	Falconidae	*Falco rusticolus*	3.5	1	HBW2
Ciconiiformes	Falconidae	*Falco mexicanus*	3.9	1	HBW2
Ciconiiformes	Falconidae	*Falco peregrinus*	2.8	1	HBW2
Ciconiiformes	Falconidae	*Falco fasciinucha*	2.8	1	HBW2
Ciconiiformes	Podicipedidae	*Rollandia rolland*	1.7	1	HBW1
Ciconiiformes	Podicipedidae	*Rollandia microptera*	2.0	1	HBW1
Ciconiiformes	Podicipedidae	*Tachybaptus ruficollis*	3.7	1	HBW1
Ciconiiformes	Podicipedidae	*Tachybaptus novaehollandiae*	4.5	1	HBW1
Ciconiiformes	Podicipedidae	*Tachybaptus pelzelnii*	3.5	1	HBW1
Ciconiiformes	Podicipedidae	*Tachybaptus dominicus*	4.9	1	HBW1
Ciconiiformes	Podicipedidae	*Podilymbus podiceps*	5.3	1	HBW1
Ciconiiformes	Podicipedidae	*Poliocephalus poliocephalus*	3.9	1	HBW1
Ciconiiformes	Podicipedidae	*Poliocephalus rufopectus*	2.4	1	HBW1
Ciconiiformes	Podicipedidae	*Podiceps major*	3.9	1	HBW1
Ciconiiformes	Podicipedidae	*Podiceps grisegena*	4.5	1	HBW1
Ciconiiformes	Podicipedidae	*Podiceps cristatus*	3.9	1	HBW1
Ciconiiformes	Podicipedidae	*Podiceps auritus*	4.5	1	HBW1
Ciconiiformes	Podicipedidae	*Podiceps nigricollis*	3.5	1	HBW1
Ciconiiformes	Podicipedidae	*Podiceps occipitalis*	2.0	1	HBW1
Ciconiiformes	Podicipedidae	*Podiceps taczanowskii*	1.7	1	HBW1
Ciconiiformes	Podicipedidae	*Podiceps gallardoi*	2.0	1	HBW1
Ciconiiformes	Podicipedidae	*Aechmophorus occidentalis*	3.5	1	HBW1
Ciconiiformes	Podicipedidae	*Aechmophorus clarkii*	3.5	1	HBW1
Ciconiiformes	Anhingidae	*Anhinga anhinga*	3.9	1	HBW1
Ciconiiformes	Anhingidae	*Anhinga melanogaster*	3.9	1	HBW1
Ciconiiformes	Phalacrocoracida	*Phalacrocorax africanus*	3.5	1	HBW1
Ciconiiformes	Phalacrocoracida	*Phalacrocorax pygmeus*	4.9	1	HBW1
Ciconiiformes	Phalacrocoracida	*Phalacrocorax niger*	3.9	1	HBW1
Ciconiiformes	Phalacrocoracida	*Phalacrocorax melanoleucos*	3.9	1	HBW1
Ciconiiformes	Phalacrocoracida	*Phalacrocorax brasilianus*	3.5	1	HBW1
Ciconiiformes	Phalacrocoracida	*Phalacrocorax auritus*	3.5	1	HBW1
Ciconiiformes	Phalacrocoracida	*Phalacrocorax fuscicollis*	4.2	1	HBW1
Ciconiiformes	Phalacrocoracida	*Phalacrocorax varius*	2.2	1	HBW1
Ciconiiformes	Phalacrocoracida	*Phalacrocorax sulcirostris*	3.5	1	HBW1
Ciconiiformes	Phalacrocoracida	*Phalacrocorax carbo*	3.5	1	HBW1
Ciconiiformes	Ardeidae	*Tigrisoma mexicanum*	1.7	1	HBW1
Ciconiiformes	Ardeidae	*Tigrisoma lineatum*	3.0	1	HBW1
Ciconiiformes	Ardeidae	*Tigriornis leucolophus*	1.0	1	HBW1

Order	Family	*Species*	Clutch	Range	Traits
Ciconiiformes	Ardeidae	*Cochlearius cochlearia*	2.8	1	HBW1
Ciconiiformes	Ardeidae	*Ixobrychus involucris*	3.5	1	HBW1
Ciconiiformes	Ardeidae	*Ixobrychus minutus*	4.2	1	HBW1
Ciconiiformes	Ardeidae	*Ixobrychus sinensis*	4.6	1	HBW1
Ciconiiformes	Ardeidae	*Ixobrychus exilis*	3.7	1	HBW1
Ciconiiformes	Ardeidae	*Ixobrychus eurhythmus*	4.2	1	HBW1
Ciconiiformes	Ardeidae	*Ixobrychus cinnamomeus*	3.9	1	HBW1
Ciconiiformes	Ardeidae	*Ixobrychus sturmii*	3.5	1	HBW1
Ciconiiformes	Ardeidae	*Ixobrychus flavicollis*	4.2	1	HBW1
Ciconiiformes	Ardeidae	*Botaurus lentiginosus*	3.7	1	HBW1
Ciconiiformes	Ardeidae	*Botaurus pinnatus*	2.4	1	HBW1
Ciconiiformes	Ardeidae	*Botaurus stellaris*	4.6	1	HBW1
Ciconiiformes	Ardeidae	*Botaurus poiciloptilus*	4.2	1	HBW1
Ciconiiformes	Ardeidae	*Agamia agami*	2.8	1	HBW1
Ciconiiformes	Ardeidae	*Nycticorax nycticorax*	3.9	1	HBW1
Ciconiiformes	Ardeidae	*Nycticorax caledonicus*	3.2	1	HBW1
Ciconiiformes	Ardeidae	*Gorsachius leuconotus*	3.2	1	HBW1
Ciconiiformes	Ardeidae	*Gorsachius goisagi*	3.5	1	HBW1
Ciconiiformes	Ardeidae	*Gorsachius melanolophus*	3.9	1	HBW1
Ciconiiformes	Ardeidae	*Syrigma sibilatrix*	3.5	1	HBW1
Ciconiiformes	Ardeidae	*Egretta rufescens*	3.5	1	HBW1
Ciconiiformes	Ardeidae	*Egretta vinaceigula*	2.4	1	HBW1
Ciconiiformes	Ardeidae	*Egretta ardesiaca*	2.8	1	HBW1
Ciconiiformes	Ardeidae	*Egretta tricolor*	3.5	1	HBW1
Ciconiiformes	Ardeidae	*Egretta novaehollandiae*	3.9	1	HBW1
Ciconiiformes	Ardeidae	*Egretta garzetta*	3.5	1	HBW1
Ciconiiformes	Ardeidae	*Egretta thula*	3.2	1	HBW1
Ciconiiformes	Ardeidae	*Egretta eulophotes*	3.2	1	HBW1
Ciconiiformes	Ardeidae	*Ardea cinerea*	3.9	1	HBW1
Ciconiiformes	Ardeidae	*Ardea herodias*	3.7	1	HBW1
Ciconiiformes	Ardeidae	*Ardea cocoi*	2.8	1	HBW1
Ciconiiformes	Ardeidae	*Ardea pacifica*	3.5	1	HBW1
Ciconiiformes	Ardeidae	*Ardea melanocephala*	3.5	1	HBW1
Ciconiiformes	Ardeidae	*Ardea humbloti*	3.0	1	HBW1
Ciconiiformes	Ardeidae	*Ardea goliath*	3.0	1	HBW1
Ciconiiformes	Ardeidae	*Ardea insignis*	4.0	1	HBW1
Ciconiiformes	Ardeidae	*Ardea sumatrana*	2.0	1	HBW1
Ciconiiformes	Ardeidae	*Ardea purpurea*	4.0	1	HBW1
Ciconiiformes	Ardeidae	*Ardea picata*	2.8	1	HBW1
Ciconiiformes	Ardeidae	*Ardea alba*	3.9	1	HBW1
Ciconiiformes	Ardeidae	*Ardea intermedia*	3.5	1	HBW1
Ciconiiformes	Ardeidae	*Ardeola ralloides*	3.7	1	HBW1
Ciconiiformes	Ardeidae	*Ardeola grayii*	3.9	1	HBW1
Ciconiiformes	Ardeidae	*Ardeola bacchus*	4.0	1	HBW1
Ciconiiformes	Ardeidae	*Ardeola speciosa*	3.2	1	HBW1
Ciconiiformes	Ardeidae	*Ardeola idae*	2.8	1	HBW1
Ciconiiformes	Ardeidae	*Ardeola rufiventris*	2.0	1	HBW1
Ciconiiformes	Scopidae	*Scopus umbretta*	4.2	1	HBW1
Ciconiiformes	Phoenicopteridae	*Phoenicopterus ruber*	1.0	1	HBW1
Ciconiiformes	Phoenicopteridae	*Phoenicopterus chilensis*	1.0	1	HBW1
Ciconiiformes	Phoenicopteridae	*Phoenicopterus minor*	1.0	1	HBW1
Ciconiiformes	Phoenicopteridae	*Phoenicopterus andinus*	1.0	1	HBW1
Ciconiiformes	Phoenicopteridae	*Phoenicopterus jamesi*	1.0	1	HBW1
Ciconiiformes	Threskiornithidae	*Eudocimus albus*	2.4	1	HBW1
Ciconiiformes	Threskiornithidae	*Eudocimus ruber*	2.0	1	HBW1
Ciconiiformes	Threskiornithidae	*Phimosus infuscatus*	3.5	1	HBW1
Ciconiiformes	Threskiornithidae	*Plegadis falcinellus*	3.5	1	HBW1
Ciconiiformes	Threskiornithidae	*Plegadis chihi*	3.0	1	HBW1
Ciconiiformes	Threskiornithidae	*Plegadis ridgwayi*	2.0	1	HBW1
Ciconiiformes	Threskiornithidae	*Theristicus caerulescens*	2.4	1	HBW1
Ciconiiformes	Threskiornithidae	*Theristicus caudatus*	2.8	1	HBW1
Ciconiiformes	Threskiornithidae	*Theristicus melanopis*	2.4	1	HBW1
Ciconiiformes	Threskiornithidae	*Bostrychia hagedash*	2.4	1	HBW1

Order	Family	*Species*	Clutch	Range	Traits
Ciconiiformes	Threskiornithidae	*Bostrychia carunculata*	2.4	1	HBW1
Ciconiiformes	Threskiornithidae	*Bostrychia olivacea*	3.0	1	HBW1
Ciconiiformes	Threskiornithidae	*Bostrychia rara*	2.0	1	HBW1
Ciconiiformes	Threskiornithidae	*Geronticus eremita*	2.8	1	HBW1
Ciconiiformes	Threskiornithidae	*Geronticus calvus*	1.7	1	HBW1
Ciconiiformes	Threskiornithidae	*Lophotibis cristata*	3.0	1	HBW1
Ciconiiformes	Threskiornithidae	*Threskiornis aethiopicus*	2.4	1	HBW1
Ciconiiformes	Threskiornithidae	*Threskiornis melanocephalus*	2.8	1	HBW1
Ciconiiformes	Threskiornithidae	*Threskiornis molucca*	2.0	1	HBW1
Ciconiiformes	Threskiornithidae	*Threskiornis spinicollis*	3.2	1	HBW1
Ciconiiformes	Threskiornithidae	*Pseudibis papillosa*	2.8	1	HBW1
Ciconiiformes	Threskiornithidae	*Pseudibis davisoni*	2.8	1	HBW1
Ciconiiformes	Threskiornithidae	*Pseudibis gigantea*	2.4	1	HBW1
Ciconiiformes	Threskiornithidae	*Nipponia nippon*	3.0	1	HBW1
Ciconiiformes	Threskiornithidae	*Platalea leucorodia*	3.5	1	HBW1
Ciconiiformes	Threskiornithidae	*Platalea regia*	3.0	1	HBW1
Ciconiiformes	Threskiornithidae	*Platalea alba*	2.4	1	HBW1
Ciconiiformes	Threskiornithidae	*Platalea minor*	4.9	1	HBW1
Ciconiiformes	Threskiornithidae	*Platalea flavipes*	3.0	1	HBW1
Ciconiiformes	Threskiornithidae	*Ajaia ajaja*	2.4	1	HBW1
Ciconiiformes	Pelecanidae	*Balaeniceps rex*	1.7	1	HBW1
Ciconiiformes	Pelecanidae	*Pelecanus onocrotalus*	1.7	1	HBW1
Ciconiiformes	Pelecanidae	*Pelecanus rufescens*	1.7	1	HBW1
Ciconiiformes	Pelecanidae	*Pelecanus crispus*	2.0	1	HBW1
Ciconiiformes	Pelecanidae	*Pelecanus philippensis*	3.5	1	HBW1
Ciconiiformes	Pelecanidae	*Pelecanus conspicillatus*	2.0	1	HBW1
Ciconiiformes	Pelecanidae	*Pelecanus erythrorhynchos*	2.0	1	HBW1
Ciconiiformes	Pelecanidae	*Pelecanus occidentalis*	2.4	1	HBW1
Ciconiiformes	Ciconiidae	*Coragyps atratus*	2.0	1	HBW2
Ciconiiformes	Ciconiidae	*Cathartes aura*	2.0	1	HBW2
Ciconiiformes	Ciconiidae	*Gymnogyps californianus*	1.0	1	HBW2
Ciconiiformes	Ciconiidae	*Mycteria americana*	3.2	1	HBW1
Ciconiiformes	Ciconiidae	*Mycteria cinerea*	2.0	1	HBW1
Ciconiiformes	Ciconiidae	*Mycteria ibis*	2.4	1	HBW1
Ciconiiformes	Ciconiidae	*Mycteria leucocephala*	3.2	1	HBW1
Ciconiiformes	Ciconiidae	*Anastomus oscitans*	3.2	1	HBW1
Ciconiiformes	Ciconiidae	*Anastomus lamelligerus*	3.2	1	HBW1
Ciconiiformes	Ciconiidae	*Ciconia nigra*	3.5	1	HBW1
Ciconiiformes	Ciconiidae	*Ciconia abdimii*	2.4	1	HBW1
Ciconiiformes	Ciconiidae	*Ciconia episcopus*	2.8	1	HBW1
Ciconiiformes	Ciconiidae	*Ciconia stormi*	2.0	1	HBW1
Ciconiiformes	Ciconiidae	*Ciconia maguari*	2.8	1	HBW1
Ciconiiformes	Ciconiidae	*Ciconia ciconia*	2.6	1	HBW1
Ciconiiformes	Ciconiidae	*Ciconia boyciana*	3.5	1	HBW1
Ciconiiformes	Ciconiidae	*Ephippiorhynchus asiaticus*	3.2	1	HBW1
Ciconiiformes	Ciconiidae	*Ephippiorhynchus senegalensis*	2.2	1	HBW1
Ciconiiformes	Ciconiidae	*Jabiru mycteria*	3.2	1	HBW1
Ciconiiformes	Ciconiidae	*Leptoptilos javanicus*	2.8	1	HBW1
Ciconiiformes	Ciconiidae	*Leptoptilos crumeniferus*	2.0	1	HBW1
Ciconiiformes	Ciconiidae	*Leptoptilos dubius*	2.8	1	HBW1
Ciconiiformes	Gaviidae	*Gavia stellata*	1.7	1	HBW1
Ciconiiformes	Gaviidae	*Gavia arctica*	1.7	1	HBW1
Ciconiiformes	Gaviidae	*Gavia immer*	1.7	1	HBW1
Ciconiiformes	Gaviidae	*Gavia adamsii*	2.0	1	HBW1
Passeriformes	Pittidae	*Pitta phayrei*	4.0	7	HBW8, Birds of Southeast Asia
Passeriformes	Pittidae	*Pitta nipalensis*	3.9	7	HBW8, Book of Indian Birds
Passeriformes	Pittidae	*Pitta soror*	3.0	7	HBW8, Birds of Southeast Asia
Passeriformes	Pittidae	*Pitta oatesi*	3.5	7	HBW8, Birds of Southeast Asia
Passeriformes	Pittidae	*Pitta caerulea*	2.4	7	HBW8, Birds of Southeast Asia
Passeriformes	Pittidae	*Pitta cyanea*	5.3	7	HBW8, Birds of Southeast Asia
Passeriformes	Pittidae	*Pitta guajana*	3.2	7	HBW8, Birds of Southeast Asia
Passeriformes	Pittidae	*Pitta elliotii*	2.8	7	HBW8, Birds of Southeast Asia
Passeriformes	Pittidae	*Pitta gurneyi*	3.9	7	HBW8, Birds of Southeast Asia

Order	Family	*Species*	Clutch	Range	Traits
Passeriformes	Pittidae	*Pitta baudii*	2.0	7	HBW8, PittasEtc
Passeriformes	Pittidae	*Pitta sordida*	3.2	7,8	HBW8, Birds of Southeast Asia, HBW8
Passeriformes	Pittidae	*Pitta maxima*	1.0	7	HBW8, PittasEtc
Passeriformes	Pittidae	*Pitta erythrogaster*	2.8	7,8,10	HBW8, Handbook of Australian, New Zeeland and Antarctic Birds, HB
Passeriformes	Pittidae	*Pitta arquata*	2.0	7	HBW8, PittasEtc
Passeriformes	Pittidae	*Pitta granatina*	2.0	7	HBW8, Birds of Southeast Asia
Passeriformes	Pittidae	*Pitta ussheri*	2.0	14	HBW8, Birds of Southeast Asia
Passeriformes	Pittidae	*Pitta venusta*	2.4	7	HBW8, PittasEtc
Passeriformes	Pittidae	*Pitta angolensis*	3.0	5	HBW8, BA
Passeriformes	Pittidae	*Pitta reichenowi*	2.4	5	HBW8, BA
Passeriformes	Pittidae	*Pitta brachyura*	4.9	7	HBW8, PittasEtc
Passeriformes	Pittidae	*Pitta nympha*	4.9	7	HBW8, Birds of Southeast Asia
Passeriformes	Pittidae	*Pitta moluccensis*	4.6	7	HBW8, Handbook of Australian, New Zeeland and Antarctic Birds
Passeriformes	Pittidae	*Pitta megarhyncha*	2.8	7	HBW8, Birds of Southeast Asia
Passeriformes	Pittidae	*Pitta elegans*	2.4	7	HBW8, PittasEtc
Passeriformes	Pittidae	*Pitta iris*	3.9	10	HBW8, Handbook of Australian, New Zeeland and Antarctic Birds
Passeriformes	Pittidae	*Pitta versicolor*	3.9	8,10	HBW8, Handbook of Australian, New Zeeland and Antarctic Birds
Passeriformes	Eurylaimidae	*Smithornis capensis*	2.4	5	HBW8, BA
Passeriformes	Eurylaimidae	*Smithornis sharpei*	2.0	5	HBW8, BA
Passeriformes	Eurylaimidae	*Smithornis rufolateralis*	2.0	5	HBW8, BA
Passeriformes	Eurylaimidae	*Corydon sumatranus*	2.8	7	HBW8, Birds of Southeast Asia
Passeriformes	Eurylaimidae	*Cymbirhynchus macrorhynchos*	2.8	7	HBW8, HBW8
Passeriformes	Eurylaimidae	*Eurylaimus javanicus*	2.4	7	HBW8, Birds of Southeast Asia
Passeriformes	Eurylaimidae	*Eurylaimus ochromalus*	2.4	7	HBW8, Birds of Southeast Asia
Passeriformes	Eurylaimidae	*Eurylaimus steerii*	1.0	7	HBW8, HBW8
Passeriformes	Eurylaimidae	*Serilophus lunatus*	3.7	7	HBW8, Birds of Southeast Asia
Passeriformes	Eurylaimidae	*Psarisomus dalhousiae*	5.7	7	HBW8, Birds of Southeast Asia
Passeriformes	Eurylaimidae	*Calyptomena viridis*	1.7	7	HBW8, Birds of Southeast Asia
Passeriformes	Eurylaimidae	*Calyptomena hosii*	2.8	7	HBW8, HBW8
Passeriformes	Eurylaimidae	*Calyptomena whiteheadi*	1.4	7	HBW8, HBW8
Passeriformes	Tyrannidae	*Mionectes striaticollis*	2.4	2	HBW9, BSA
Passeriformes	Tyrannidae	*Mionectes olivaceus*	2.4	2	HBW9, BSA, Birds of Costa Rica
Passeriformes	Tyrannidae	*Mionectes oleagineus*	3.2	2	HBW9, BSA, Birds of Venezuela, Birds of Costa Rica
Passeriformes	Tyrannidae	*Mionectes macconnelli*	3.0	2	HBW9, BSA, Birds of Venezuela
Passeriformes	Tyrannidae	*Mionectes rufiventris*	3.0	2	HBW9, BSA
Passeriformes	Tyrannidae	*Leptopogon amaurocephalus*	2.4	2	HBW9, BSA, Birds of Costa Rica
Passeriformes	Tyrannidae	*Leptopogon superciliaris*	2.0	2	HBW9, BSA, Birds of Venezuela, Birds of Costa Rica
Passeriformes	Tyrannidae	*Hemitriccus obsoletus*	2.0	2	HBW9, BSA, BHA
Passeriformes	Tyrannidae	*Hemitriccus striaticollis*	2.0	2	HBW9, BSA, BHA
Passeriformes	Tyrannidae	*Hemitriccus nidipendulus*	2.0	2	HBW9, BSA, BHA
Passeriformes	Tyrannidae	*Hemitriccus margaritaceivente*	1.7	2	HBW9, BSA, Birds of Venezuela
Passeriformes	Tyrannidae	*Hemitriccus granadensis*	2.0	2	HBW9, BSA, Birds of Venezuela
Passeriformes	Tyrannidae	*Hemitriccus rufigularis*	2.0	2	HBW9, BSA, BHA
Passeriformes	Tyrannidae	*Todirostrum plumbeiceps*	2.4	2	HBW9, BSA, BHA
Passeriformes	Tyrannidae	*Todirostrum sylvia*	2.0	2	HBW9, HBW9
Passeriformes	Tyrannidae	*Todirostrum maculatum*	1.4	2	HBW9, BSA, BHA
Passeriformes	Tyrannidae	*Todirostrum cinereum*	2.4	2	HBW9, BSA, Birds of Venezuela, Birds of Costa Rica
Passeriformes	Tyrannidae	*Todirostrum nigriceps*	1.0	2	HBW9, Birds of Venezuela, Birds of Costa Rica
Passeriformes	Tyrannidae	*Todirostrum chrysocrotaphum*	2.0	2	HBW9, BSA, BHA
Passeriformes	Tyrannidae	*Corythopis delalandi*	2.4	2	HBW8, BSA
Passeriformes	Tyrannidae	*Phyllomyias fasciatus*	2.0	2	HBW9, BSA
Passeriformes	Tyrannidae	*Phyllomyias virescens*	2.0	2	HBW9, BSA, Birds of Venezuela
Passeriformes	Tyrannidae	*Zimmerius vilissimus*	2.0	2	HBW9, BSA, Birds of Venezuela, Birds of Costa Rica
Passeriformes	Tyrannidae	*Camptostoma imberbe*	2.8	2	HBW9, Birds of Costa Rica
Passeriformes	Tyrannidae	*Camptostoma obsoletum*	2.4	2	HBW9, BSA, Birds of Venezuela, Birds of Costa Rica
Passeriformes	Tyrannidae	*Capsiempis flaveola*	2.0	2	HBW9, BSA, Birds of Venezuela, Birds of Costa Rica
Passeriformes	Tyrannidae	*Sublegatus arenarum*	2.0	2	HBW9, BSA
Passeriformes	Tyrannidae	*Sublegatus modestus*	2.0	2	HBW9, BSA, Birds of Venezuela, Birds of Costa Rica
Passeriformes	Tyrannidae	*Tyrannulus elatus*	1.4	2	HBW9, BSA, Birds of Costa Rica
Passeriformes	Tyrannidae	*Myiopagis cotta*	3.0	2	HBW9, Birds of the West Indies
Passeriformes	Tyrannidae	*Myiopagis viridicata*	2.0	2	HBW9, BSA, Birds of Venezuela, Birds of Costa Rica
Passeriformes	Tyrannidae	*Pseudelaenia leucospodia*	2.4	2	HBW9, BSA
Passeriformes	Tyrannidae	*Elaenia martinica*	2.4	2	HBW9, BSA, Birds of the West Indies

Order	Family	Species	Clutch	Range	Traits
Passeriformes	Tyrannidae	*Elaenia flavogaster*	1.7	2	HBW9, BSA, Birds of Venezuela, Birds of Costa Rica
Passeriformes	Tyrannidae	*Elaenia albiceps*	2.4	2	HBW9, BSA, BHA
Passeriformes	Tyrannidae	*Elaenia parvirostris*	2.4	2	HBW9, BSA, BHA
Passeriformes	Tyrannidae	*Elaenia mesoleuca*	2.4	2	HBW9, BSA, BHA
Passeriformes	Tyrannidae	*Elaenia chiriquensis*	2.0	2	HBW9, BSA, Birds of Venezuela, Birds of Costa Rica
Passeriformes	Tyrannidae	*Elaenia frantzii*	2.0	2	HBW9, BSA, Birds of Venezuela, Birds of Costa Rica
Passeriformes	Tyrannidae	*Elaenia fallax*	2.0	2	HBW9, Birds of the West Indies
Passeriformes	Tyrannidae	*Serpophaga cinerea*	2.0	2	HBW9, BSA, Birds of Costa Rica
Passeriformes	Tyrannidae	*Serpophaga subcristata*	2.4	2	HBW9, BSA
Passeriformes	Tyrannidae	*Serpophaga munda*	2.4	2	HBW9, BSA
Passeriformes	Tyrannidae	*Stigmatura budytoides*	2.0	2	HBW9, BSA
Passeriformes	Tyrannidae	*Anairetes flavirostris*	2.4	2	HBW9, BSA, BHA
Passeriformes	Tyrannidae	*Anairetes parulus*	2.4	2	HBW9, BSA, BHA
Passeriformes	Tyrannidae	*Tachuris rubrigastra*	3.5	2	HBW9, BSA
Passeriformes	Tyrannidae	*Polystictus pectoralis*	3.0	2	HBW9, BSA
Passeriformes	Tyrannidae	*Polystictus superciliaris*	1.4	2	HBW9, BSA
Passeriformes	Tyrannidae	*Pseudocolopteryx sclateri*	2.0	2	HBW9, BSA
Passeriformes	Tyrannidae	*Pseudocolopteryx dinellianus*	3.0	2	HBW9, BSA
Passeriformes	Tyrannidae	*Pseudocolopteryx acutipennis*	3.0	2	HBW9, BSA
Passeriformes	Tyrannidae	*Pseudocolopteryx flaviventris*	2.8	2	HBW9, BSA
Passeriformes	Tyrannidae	*Euscarthmus meloryphus*	2.0	2	HBW9, BSA, Birds of Venezuela
Passeriformes	Tyrannidae	*Myiornis atricapillus*	2.0	2	HBW9, BSA, Birds of Costa Rica
Passeriformes	Tyrannidae	*Lophotriccus pileatus*	2.0	2	HBW9, BSA, Birds of Costa Rica
Passeriformes	Tyrannidae	*Lophotriccus galeatus*	2.4	2	HBW9, BSA, Birds of Venezuela
Passeriformes	Tyrannidae	*Oncostoma cinereigulare*	2.0	2	HBW9, BSA, Birds of Costa Rica
Passeriformes	Tyrannidae	*Oncostoma olivaceum*	2.0	2	HBW9, BSA
Passeriformes	Tyrannidae	*Rhynchocyclus brevirostris*	2.4	2	HBW9, Birds of Costa Rica
Passeriformes	Tyrannidae	*Rhynchocyclus olivaceus*	2.4	2	HBW9, Birds of Venezuela
Passeriformes	Tyrannidae	*Rhynchocyclus fulvipectus*	2.4	2	HBW9, HBW9
Passeriformes	Tyrannidae	*Tolmomyias sulphurescens*	2.4	2	HBW9, BSA, Birds of Venezuela, Birds of Costa Rica
Passeriformes	Tyrannidae	*Tolmomyias flaviventris*	2.4	2	HBW9, BSA
Passeriformes	Tyrannidae	*Platyrinchus cancrominus*	2.0	2	HBW9, Birds of Costa Rica
Passeriformes	Tyrannidae	*Platyrinchus mystaceus*	2.0	2	HBW9, BSA, Birds of Venezuela, Birds of Costa Rica
Passeriformes	Tyrannidae	*Platyrinchus coronatus*	2.0	2	HBW9, BSA, Birds of Venezuela, Birds of Costa Rica
Passeriformes	Tyrannidae	*Platyrinchus leucoryphus*	2.0	2	HBW9, BSA
Passeriformes	Tyrannidae	*Onychorhynchus coronatus*	2.0	2	HBW9, BSA, Birds of Venezuela, Birds of Costa Rica
Passeriformes	Tyrannidae	*Myiophobus fasciatus*	1.4	2	HBW9, BSA, Birds of Venezuela, Birds of Costa Rica
Passeriformes	Tyrannidae	*Myiobius erythrurus*	2.0	2	HBW9, BSA, Birds of Venezuela, Birds of Costa Rica
Passeriformes	Tyrannidae	*Myiobius barbatus*	2.0	2	HBW9, BSA, Birds of Venezuela, Birds of Costa Rica
Passeriformes	Tyrannidae	*Myiobius atricaudus*	2.0	2	HBW9, BSA, Birds of Costa Rica
Passeriformes	Tyrannidae	*Pyrrhomyias cinnamomea*	2.0	2	HBW9, BSA, BHA
Passeriformes	Tyrannidae	*Hirundinea ferruginea*	2.0	2	HBW9, BSA, Birds of Venezuela
Passeriformes	Tyrannidae	*Cnemotriccus fuscatus*	3.0	2	HBW9, BSA, Birds of Venezuela
Passeriformes	Tyrannidae	*Lathrotriccus euleri*	2.4	2	HBW9, BSA, Birds of Venezuela, Birds of the West Indies
Passeriformes	Tyrannidae	*Xenotriccus callizonus*	3.0	2	HBW9, BMx
Passeriformes	Tyrannidae	*Xenotriccus mexicanus*	2.4	2	HBW9, BMx
Passeriformes	Tyrannidae	*Mitrephanes phaeocercus*	2.0	2	HBW9, BSA, Birds of Costa Rica
Passeriformes	Tyrannidae	*Contopus cooperi*	3.9	2	HBW9, BSA, Birds of Costa Rica
Passeriformes	Tyrannidae	*Contopus pertinax*	2.8	2	HBW9, BMx, BH
Passeriformes	Tyrannidae	*Contopus sordidulus*	2.8	2	HBW9, BSA, Birds of Costa Rica
Passeriformes	Tyrannidae	*Contopus virens*	2.8	2	HBW9, BSA, Birds of Costa Rica
Passeriformes	Tyrannidae	*Contopus cinereus*	2.4	2	HBW9, BSA, Birds of Venezuela, Birds of Costa Rica
Passeriformes	Tyrannidae	*Contopus caribaeus*	2.8	2	HBW9, Birds of the West Indies
Passeriformes	Tyrannidae	*Contopus hispaniolensis*	2.8	2	HBW9, Birds of the West Indies
Passeriformes	Tyrannidae	*Empidonax flaviventris*	3.9	2	HBW9, Birds of Costa Rica
Passeriformes	Tyrannidae	*Empidonax virescens*	2.0	2	HBW9, BSA
Passeriformes	Tyrannidae	*Empidonax alnorum*	3.5	2	HBW9, BSA, Birds of Costa Rica
Passeriformes	Tyrannidae	*Empidonax traillii*	3.5	2	HBW9, BSA, Birds of Venezuela, Birds of Costa Rica
Passeriformes	Tyrannidae	*Empidonax albigularis*	2.4	2	HBW9, Birds of Costa Rica
Passeriformes	Tyrannidae	*Empidonax minimus*	4.0	2	HBW9, Birds of Costa Rica
Passeriformes	Tyrannidae	*Empidonax hammondii*	3.5	2	HBW9, BMx, BH
Passeriformes	Tyrannidae	*Empidonax wrightii*	3.5	2	HBW9, BMx, BH
Passeriformes	Tyrannidae	*Empidonax oberholseri*	3.5	2	HBW9, BMx, BH

Order	Family	*Species*	Clutch	Range	Traits
Passeriformes	Tyrannidae	*Empidonax difficilis*	2.2	2	HBW9, BMx, BH
Passeriformes	Tyrannidae	*Empidonax occidentalis*	3.5	2	HBW9, BMx, BH
Passeriformes	Tyrannidae	*Empidonax flavescens*	2.4	2	HBW9, Birds of Costa Rica
Passeriformes	Tyrannidae	*Empidonax fulvifrons*	3.2	2	HBW9, BMx, BH
Passeriformes	Tyrannidae	*Empidonax atriceps*	2.0	2	HBW9, Birds of Costa Rica
Passeriformes	Tyrannidae	*Sayornis phoebe*	4.5	2	HBW9, Birds of the West Indies
Passeriformes	Tyrannidae	*Sayornis saya*	4.5	2	HBW9, BMx, BH
Passeriformes	Tyrannidae	*Sayornis nigricans*	2.4	2	HBW9, BSA, Birds of Costa Rica
Passeriformes	Tyrannidae	*Pyrocephalus rubinus*	2.4	2	HBW9, BSA
Passeriformes	Tyrannidae	*Silvicultrix diadema*	4.0	2	HBW9, BSA, Birds of Venezuela
Passeriformes	Tyrannidae	*Ochthoeca leucophrys*	1.0	2	HBW9, BSA, BHA
Passeriformes	Tyrannidae	*Colorhamphus parvirostris*	3.0	2	HBW9, BSA
Passeriformes	Tyrannidae	*Ochthornis littoralis*	3.5	2	HBW9, BSA
Passeriformes	Tyrannidae	*Xolmis pyrope*	2.4	2	HBW9, BSA
Passeriformes	Tyrannidae	*Xolmis cinerea*	2.4	2	HBW9, BSA
Passeriformes	Tyrannidae	*Xolmis coronata*	2.4	2	HBW9, BSA
Passeriformes	Tyrannidae	*Xolmis irupero*	3.5	2	HBW9, BSA
Passeriformes	Tyrannidae	*Xolmis dominicana*	3.5	2	HBW9, BSA
Passeriformes	Tyrannidae	*Xolmis rubetra*	2.0	2	HBW9, BSA
Passeriformes	Tyrannidae	*Xolmis rufiventris*	2.4	2	HBW9, BSA, BHA
Passeriformes	Tyrannidae	*Agriornis montana*	2.4	2	HBW9, BSA, BHA
Passeriformes	Tyrannidae	*Agriornis livida*	2.8	2	HBW9, BSA, BHA
Passeriformes	Tyrannidae	*Agriornis microptera*	3.5	2	HBW9, BSA, BHA
Passeriformes	Tyrannidae	*Agriornis murina*	3.0	2	HBW9, BSA, BHA
Passeriformes	Tyrannidae	*Muscisaxicola maculirostris*	2.8	2	HBW9, BSA, BHA
Passeriformes	Tyrannidae	*Muscisaxicola fluviatilis*	2.0	2	HBW9, BSA, BHA
Passeriformes	Tyrannidae	*Muscisaxicola macloviana*	2.4	2	HBW9, BSA, BHA
Passeriformes	Tyrannidae	*Muscisaxicola capistrata*	3.0	2	HBW9, BSA, BHA
Passeriformes	Tyrannidae	*Muscisaxicola rufivertex*	3.0	2	HBW9, BSA, BHA
Passeriformes	Tyrannidae	*Muscisaxicola albilora*	2.4	2	HBW9, BSA, BHA
Passeriformes	Tyrannidae	*Muscisaxicola alpina*	4.0	2	HBW9, BSA, BHA
Passeriformes	Tyrannidae	*Muscisaxicola cinerea*	2.0	2	HBW9, BSA, BHA
Passeriformes	Tyrannidae	*Muscisaxicola flavinucha*	2.8	2	HBW9, BSA, BHA
Passeriformes	Tyrannidae	*Muscigralla brevicauda*	3.9	2	HBW9, BSA
Passeriformes	Tyrannidae	*Lessonia oreas*	3.5	2	HBW9, BSA, BHA
Passeriformes	Tyrannidae	*Lessonia rufa*	2.8	2	HBW9, BSA, BHA
Passeriformes	Tyrannidae	*Knipolegus signatus*	1.7	2	HBW9, BSA
Passeriformes	Tyrannidae	*Knipolegus cyanirostris*	1.7	2	HBW9, BSA
Passeriformes	Tyrannidae	*Knipolegus aterrimus*	2.4	2	HBW9, BSA
Passeriformes	Tyrannidae	*Hymenops perspicillatus*	2.8	2	HBW9, BSA
Passeriformes	Tyrannidae	*Fluvicola pica*	2.4	2	HBW9, BSA
Passeriformes	Tyrannidae	*Fluvicola albiventer*	2.4	2	HBW9, BSA
Passeriformes	Tyrannidae	*Arundinicola leucocephala*	2.8	2	HBW9, BSA
Passeriformes	Tyrannidae	*Alectrurus risora*	3.0	2	HBW9, BSA
Passeriformes	Tyrannidae	*Satrapa icterophrys*	2.8	2	HBW9, BSA, BHA
Passeriformes	Tyrannidae	*Colonia colonus*	2.4	2	HBW9, BSA, Birds of Costa Rica
Passeriformes	Tyrannidae	*Machetornis rixosus*	3.5	2	HBW9, BSA, Birds of Venezuela
Passeriformes	Tyrannidae	*Muscipipra vetula*	3.0	2	HBW9, BSA
Passeriformes	Tyrannidae	*Attila cinnamomeus*	2.4	2	HBW9, BSA, Birds of Venezuela
Passeriformes	Tyrannidae	*Attila bolivianus*	2.0	2	HBW9, BSA
Passeriformes	Tyrannidae	*Attila spadiceus*	2.8	2	HBW9, BSA, Birds of Venezuela, Birds of Costa Rica
Passeriformes	Tyrannidae	*Laniocera hypopyrra*	2.0	14	HBW9, BSA, Birds of Venezuela
Passeriformes	Tyrannidae	*Myiarchus tuberculifer*	3.5	2	HBW9, BSA, Birds of Costa Rica
Passeriformes	Tyrannidae	*Myiarchus barbirostris*	3.5	2	HBW9, Birds of the West Indies
Passeriformes	Tyrannidae	*Myiarchus swainsoni*	3.5	2	HBW9, BSA
Passeriformes	Tyrannidae	*Myiarchus panamensis*	2.4	2	HBW9, BSA, Birds of Costa Rica
Passeriformes	Tyrannidae	*Myiarchus ferox*	2.4	2	HBW9, BSA
Passeriformes	Tyrannidae	*Myiarchus cinerascens*	4.5	2	HBW9, Birds of Costa Rica
Passeriformes	Tyrannidae	*Myiarchus nuttingi*	3.9	2	HBW9, Birds of Costa Rica
Passeriformes	Tyrannidae	*Myiarchus crinitus*	5.7	2	HBW9, BSA, Birds of Costa Rica
Passeriformes	Tyrannidae	*Myiarchus tyrannulus*	3.9	2	HBW9, BSA, Birds of Costa Rica
Passeriformes	Tyrannidae	*Myiarchus magnirostris*	3.9	2	HBW9, BGA
Passeriformes	Tyrannidae	*Myiarchus validus*	3.9	2	HBW9, Birds of the West Indies

Order	Family	*Species*	Clutch	Range	Traits
Passeriformes	Tyrannidae	*Myiarchus sagrae*	4.0	2	HBW9, Birds of the West Indies
Passeriformes	Tyrannidae	*Myiarchus stolidus*	3.5	2	HBW9, Birds of the West Indies
Passeriformes	Tyrannidae	*Myiarchus antillarum*	3.9	2	HBW9, Birds of the West Indies
Passeriformes	Tyrannidae	*Ramphotrigon megacephala*	2.0	2	HBW9, BSA
Passeriformes	Tyrannidae	*Ramphotrigon fuscicauda*	2.0	2	HBW9, BSA
Passeriformes	Tyrannidae	*Ramphotrigon ruficauda*	3.0	2	HBW9, BSA, Birds of Venezuela
Passeriformes	Tyrannidae	*Tyrannus niveigularis*	2.8	2	HBW9, BSA
Passeriformes	Tyrannidae	*Tyrannus melancholicus*	3.2	2	HBW9, BSA, Birds of Venezuela, Birds of Costa Rica
Passeriformes	Tyrannidae	*Tyrannus couchii*	3.5	2	HBW9, BMx, BH
Passeriformes	Tyrannidae	*Tyrannus vociferans*	3.5	2	HBW9, BMx, BH
Passeriformes	Tyrannidae	*Tyrannus crassirostris*	3.5	2	HBW9, BMx, BH
Passeriformes	Tyrannidae	*Tyrannus verticalis*	3.5	2	HBW9, Birds of Costa Rica
Passeriformes	Tyrannidae	*Tyrannus forficatus*	4.2	2	HBW9, Birds of Costa Rica
Passeriformes	Tyrannidae	*Tyrannus savana*	2.4	2	HBW9, BSA, Birds of Venezuela, Birds of Costa Rica
Passeriformes	Tyrannidae	*Tyrannus tyrannus*	3.9	2	HBW9, BSA, Birds of Costa Rica
Passeriformes	Tyrannidae	*Tyrannus dominicensis*	2.8	2	HBW9, BSA, Birds of Venezuela, Birds of Costa Rica, Birds of the West
Passeriformes	Tyrannidae	*Tyrannus caudifasciatus*	2.8	2	HBW9, Birds of the West Indies
Passeriformes	Tyrannidae	*Tyrannus cubensis*	2.4	2	HBW9, Birds of the West Indies
Passeriformes	Tyrannidae	*Empidonomus varius*	2.4	2	HBW9, BSA, Birds of Venezuela
Passeriformes	Tyrannidae	*Griseotyrannus aurantioatrocri*	2.8	2	HBW9, BSA, Birds of Venezuela
Passeriformes	Tyrannidae	*Tyrannopsis sulphurea*	2.0	2	HBW9, BSA, Birds of Venezuela
Passeriformes	Tyrannidae	*Megarynchus pitangua*	2.4	2	HBW9, BSA, Birds of Venezuela, Birds of Costa Rica
Passeriformes	Tyrannidae	*Conopias albovittata*	2.0	2	HBW9, BSA, Birds of Costa Rica
Passeriformes	Tyrannidae	*Conopias parva*	2.0	2	HBW9, BSA
Passeriformes	Tyrannidae	*Myiodynastes hemichrysus*	3.0	2	HBW9, Birds of Costa Rica
Passeriformes	Tyrannidae	*Myiodynastes chrysocephalus*	2.0	2	HBW9, BSA, BHA
Passeriformes	Tyrannidae	*Myiodynastes bairdii*	3.9	2	HBW9, BSA, BHA
Passeriformes	Tyrannidae	*Myiodynastes maculatus*	2.8	2	HBW9, BSA, Birds of Venezuela, Birds of Costa Rica
Passeriformes	Tyrannidae	*Myiodynastes luteiventris*	2.8	2	HBW9, BSA, Birds of Venezuela, Birds of Costa Rica
Passeriformes	Tyrannidae	*Myiozetetes cayanensis*	2.4	2	HBW9, BSA, Birds of Venezuela
Passeriformes	Tyrannidae	*Myiozetetes similis*	2.8	2	HBW9, BSA, Birds of Venezuela, Birds of Costa Rica
Passeriformes	Tyrannidae	*Myiozetetes granadensis*	2.8	2	HBW9, BSA, Birds of Venezuela, Birds of Costa Rica
Passeriformes	Tyrannidae	*Legatus leucophaius*	2.8	2	HBW9, BSA, Birds of Venezuela, Birds of Costa Rica
Passeriformes	Tyrannidae	*Philohydor lictor*	2.4	2	HBW9, BSA
Passeriformes	Tyrannidae	*Pitangus sulphuratus*	3.9	2,13	HBW9, BSA, Birds of Venezuela, Birds of Costa Rica
Passeriformes	Tyrannidae	*Phelpsia inornata*	2.0	2	HBW9, BSA, Birds of Venezuela
Passeriformes	Tyrannidae	*Schiffornis turdinus*	2.0	2	HBW9, BSA, Birds of Costa Rica
Passeriformes	Tyrannidae	*Schiffornis virescens*	2.0	2	HBW9, BSA
Passeriformes	Tyrannidae	*Xenopsaris albinucha*	3.0	2	HBW9, BSA
Passeriformes	Tyrannidae	*Pachyramphus viridis*	2.8	2	HBW9, BSA
Passeriformes	Tyrannidae	*Pachyramphus versicolor*	2.4	2	HBW9, BSA, Birds of Venezuela, Birds of Costa Rica
Passeriformes	Tyrannidae	*Pachyramphus cinnamomeus*	3.5	2	HBW9, BSA, Birds of Costa Rica
Passeriformes	Tyrannidae	*Pachyramphus polychopterus*	2.8	2	HBW9, BSA, Birds of Venezuela, Birds of Costa Rica
Passeriformes	Tyrannidae	*Pachyramphus rufus*	3.2	2	HBW9, BSA, Birds of Venezuela
Passeriformes	Tyrannidae	*Pachyramphus aglaiae*	3.5	2	HBW9, Birds of Costa Rica
Passeriformes	Tyrannidae	*Pachyramphus minor*	4.9	2	HBW9, BSA, Birds of Venezuela, Birds of Costa Rica
Passeriformes	Tyrannidae	*Pachyramphus niger*	3.0	2	HBW9, Birds of the West Indies
Passeriformes	Tyrannidae	*Pachyramphus validus*	4.0	2	HBW9, BSA
Passeriformes	Tyrannidae	*Tityra cayana*	3.0	2	HBW9, BSA
Passeriformes	Tyrannidae	*Tityra semifasciata*	2.4	2	HBW9, BSA, Birds of Venezuela, Birds of Costa Rica
Passeriformes	Tyrannidae	*Tityra inquisitor*	3.0	2	HBW9, BSA, Birds of Costa Rica
Passeriformes	Tyrannidae	*Phibalura flavirostris*	2.4	2	HBW9, BSA
Passeriformes	Tyrannidae	*Phytotoma raimondii*	2.8	2	HBW9, BSA
Passeriformes	Tyrannidae	*Phytotoma rutila*	2.8	2	HBW9+E3472, BSA
Passeriformes	Tyrannidae	*Phytotoma rara*	2.8	2	HBW9, BSA
Passeriformes	Tyrannidae	*Zaratornis stresemanni*	3.0	2	HBW9, BSA
Passeriformes	Tyrannidae	*Pipreola riefferii*	2.0	2	HBW9, BSA
Passeriformes	Tyrannidae	*Pipreola jucunda*	2.0	2	HBW9, BSA
Passeriformes	Tyrannidae	*Iodopleura pipra*	1.0	2	HBW9, BSA
Passeriformes	Tyrannidae	*Lipaugus unirufus*	1.0	2	HBW9, BSA, Birds of Costa Rica
Passeriformes	Tyrannidae	*Lipaugus lanioides*	1.0	2	HBW9, BSA
Passeriformes	Tyrannidae	*Cotinga ridgwayi*	2.0	2	HBW9, Birds of Costa Rica
Passeriformes	Tyrannidae	*Cotinga nattererii*	2.0	2	HBW9, BSA

Order	Family	*Species*	Clutch	Range	Traits
Passeriformes	Tyrannidae	*Xipholena punicea*	1.0	2	HBW9, BSA, Birds of Venezuela
Passeriformes	Tyrannidae	*Xipholena lamellipennis*	1.0	2	HBW9, BSA
Passeriformes	Tyrannidae	*Xipholena atropurpurea*	1.0	2	HBW9, BSA
Passeriformes	Tyrannidae	*Carpodectes nitidus*	1.0	2	HBW9, Birds of Costa Rica
Passeriformes	Tyrannidae	*Carpodectes antoniae*	1.0	2	HBW9, Birds of Costa Rica
Passeriformes	Tyrannidae	*Gymnoderus foetidus*	1.0	2	HBW9, BSA, Birds of Venezuela
Passeriformes	Tyrannidae	*Querula purpurata*	1.0	2	HBW9, BSA, Birds of Venezuela
Passeriformes	Tyrannidae	*Pyroderus scutatus*	1.0	2	HBW9, BSA, Birds of Venezuela
Passeriformes	Tyrannidae	*Cephalopterus glabricollis*	1.0	2	HBW9, Birds of Costa Rica
Passeriformes	Tyrannidae	*Cephalopterus penduliger*	1.0	2	HBW9, BSA
Passeriformes	Tyrannidae	*Cephalopterus ornatus*	1.0	2	HBW9, BSA, Birds of Venezuela
Passeriformes	Tyrannidae	*Perissocephalus tricolor*	1.0	2	HBW9, BSA, Birds of Venezuela
Passeriformes	Tyrannidae	*Procnias averano*	1.0	2	HBW9, BSA
Passeriformes	Tyrannidae	*Procnias nudicollis*	1.0	2	HBW9, BSA
Passeriformes	Tyrannidae	*Oxyruncus cristatus*	2.0	2	HBW9, BSA
Passeriformes	Tyrannidae	*Pipra aureola*	2.0	2	HBW9, BSA
Passeriformes	Tyrannidae	*Pipra fasciicauda*	2.0	2	HBW9, BSA
Passeriformes	Tyrannidae	*Pipra filicauda*	2.0	2	HBW9, BSA, Birds of Venezuela
Passeriformes	Tyrannidae	*Pipra mentalis*	2.0	2	HBW9, BSA, Birds of Costa Rica
Passeriformes	Tyrannidae	*Pipra erythrocephala*	2.0	2	HBW9, BSA
Passeriformes	Tyrannidae	*Pipra rubrocapilla*	2.0	2	HBW9, BSA
Passeriformes	Tyrannidae	*Pipra pipra*	2.0	2	HBW9, BSA, Birds of Costa Rica
Passeriformes	Tyrannidae	*Lepidothrix coronata*	2.0	2	HBW9, BSA, Birds of Costa Rica
Passeriformes	Tyrannidae	*Lepidothrix serena*	2.0	2	HBW9, BSA
Passeriformes	Tyrannidae	*Antilophia galeata*	2.0	2	HBW9, BSA
Passeriformes	Tyrannidae	*Chiroxiphia linearis*	2.0	2	HBW9, Birds of Costa Rica
Passeriformes	Tyrannidae	*Chiroxiphia lanceolata*	2.0	2	HBW9, BSA, Birds of Costa Rica
Passeriformes	Tyrannidae	*Chiroxiphia pareola*	2.0	2	HBW9, BSA
Passeriformes	Tyrannidae	*Chiroxiphia caudata*	2.0	2	HBW9, BSA
Passeriformes	Tyrannidae	*Masius chrysopterus*	2.0	2	HBW9, BSA
Passeriformes	Tyrannidae	*Corapipo leucorrhoa*	2.0	2	HBW9, BSA, Birds of Venezuela, Birds of Costa Rica
Passeriformes	Tyrannidae	*Manacus candei*	2.0	2	HBW9, Birds of Costa Rica
Passeriformes	Tyrannidae	*Manacus aurantiacus*	2.0	2	HBW9, Birds of Costa Rica
Passeriformes	Tyrannidae	*Manacus vitellinus*	2.0	2	HBW9, BSA
Passeriformes	Tyrannidae	*Machaeropterus pyrocephalus*	2.0	2	HBW9, BSA
Passeriformes	Tyrannidae	*Machaeropterus deliciosus*	2.0	2	HBW9, BSA
Passeriformes	Tyrannidae	*Heterocercus aurantiivertex*	2.0	2	HBW9, BSA
Passeriformes	Thamnophilidae	*Cymbilaimus lineatus*	2.0	2	HBW8, BSA, Birds of Costa Rica
Passeriformes	Thamnophilidae	*Batara cinerea*	2.4	2	HBW8, BSA
Passeriformes	Thamnophilidae	*Frederickena viridis*	2.0	2	HBW8, BSA
Passeriformes	Thamnophilidae	*Taraba major*	2.4	2	HBW8, BSA, Birds of Costa Rica
Passeriformes	Thamnophilidae	*Sakesphorus canadensis*	2.4	2	HBW8, BSA
Passeriformes	Thamnophilidae	*Sakesphorus bernardi*	2.4	2	HBW8, BSA
Passeriformes	Thamnophilidae	*Sakesphorus melanonotus*	2.0	2	HBW8, BSA
Passeriformes	Thamnophilidae	*Thamnophilus doliatus*	1.7	2	HBW8, BSA, Birds of Costa Rica
Passeriformes	Thamnophilidae	*Thamnophilus palliatus*	2.0	2	HBW8, BSA
Passeriformes	Thamnophilidae	*Thamnophilus tenuepunctatus*	2.0	2	HBW8, BSA
Passeriformes	Thamnophilidae	*Thamnophilus bridgesi*	2.4	2	HBW8, Birds of Costa Rica
Passeriformes	Thamnophilidae	*Thamnophilus nigrocinereus*	2.0	2	HBW8, BSA
Passeriformes	Thamnophilidae	*Thamnophilus aethiops*	2.0	2	HBW8, BSA
Passeriformes	Thamnophilidae	*Thamnophilus schistaceus*	2.0	2	HBW8, BSA
Passeriformes	Thamnophilidae	*Thamnophilus murinus*	2.0	2	HBW8, BSA
Passeriformes	Thamnophilidae	*Thamnophilus punctatus*	2.0	2	HBW8, BSA, Birds of Costa Rica
Passeriformes	Thamnophilidae	*Thamnophilus atrinucha*	2.0	2	HBW8, BSA
Passeriformes	Thamnophilidae	*Thamnophilus amazonicus*	2.0	2	HBW8, BSA
Passeriformes	Thamnophilidae	*Thamnophilus caerulescens*	2.4	2	HBW8, BSA
Passeriformes	Thamnophilidae	*Thamnophilus torquatus*	2.4	2	HBW8, BSA
Passeriformes	Thamnophilidae	*Thamnophilus ruficapillus*	2.4	2	HBW8, BSA
Passeriformes	Thamnophilidae	*Megastictus margaritatus*	2.0	2	HBW8, BSA
Passeriformes	Thamnophilidae	*Xenornis setifrons*	2.0	2	HBW8, BSA
Passeriformes	Thamnophilidae	*Thamnistes anabatinus*	2.0	2	HBW8, BSA, Birds of Costa Rica
Passeriformes	Thamnophilidae	*Dysithamnus mentalis*	2.0	2	HBW8, BSA, Birds of Costa Rica
Passeriformes	Thamnophilidae	*Dysithamnus striaticeps*	2.0	2	HBW8, Birds of Costa Rica

Order	Family	*Species*	Clutch	Range	Traits
Passeriformes	Thamnophilidae	*Dysithamnus puncticeps*	2.0	2	HBW8, BSA, Birds of Costa Rica
Passeriformes	Thamnophilidae	*Dysithamnus xanthopterus*	2.0	2	HBW8, BSA
Passeriformes	Thamnophilidae	*Dysithamnus plumbeus*	2.0	2	HBW8, BSA
Passeriformes	Thamnophilidae	*Thamnomanes ardesiacus*	2.0	2	HBW8, BSA
Passeriformes	Thamnophilidae	*Thamnomanes caesius*	2.0	2	HBW8, BSA
Passeriformes	Thamnophilidae	*Thamnomanes schistogynus*	2.0	2	HBW8, BSA
Passeriformes	Thamnophilidae	*Myrmotherula surinamensis*	2.0	2	HBW8, BSA
Passeriformes	Thamnophilidae	*Myrmotherula hauxwelli*	2.0	2	HBW8, BSA
Passeriformes	Thamnophilidae	*Myrmotherula guttata*	2.0	2	HBW8, BSA, Birds of Venezuela
Passeriformes	Thamnophilidae	*Myrmotherula gutturalis*	2.0	2	HBW8, BSA
Passeriformes	Thamnophilidae	*Myrmotherula fulviventris*	2.0	2	HBW8, BSA, Birds of Costa Rica
Passeriformes	Thamnophilidae	*Myrmotherula axillaris*	2.0	2	HBW8, BSA, Birds of Venezuela, Birds of Costa Rica
Passeriformes	Thamnophilidae	*Myrmotherula schisticolor*	2.0	2	HBW8, BSA, Birds of Costa Rica
Passeriformes	Thamnophilidae	*Myrmorchilus strigilatus*	2.0	2	HBW8, BSA
Passeriformes	Thamnophilidae	*Microrhopias quixensis*	2.0	2	HBW8, BSA, Birds of Costa Rica
Passeriformes	Thamnophilidae	*Formicivora grisea*	2.0	2	HBW8, BSA
Passeriformes	Thamnophilidae	*Formicivora littoralis*	2.0	2	HBW8, BSA
Passeriformes	Thamnophilidae	*Formicivora erythronotos*	2.0	2	HBW8, BSA
Passeriformes	Thamnophilidae	*Formicivora rufa*	2.0	2	HBW8, BSA
Passeriformes	Thamnophilidae	*Drymophila squamata*	2.0	2	HBW8, BSA
Passeriformes	Thamnophilidae	*Cercomacra tyrannina*	2.0	2	HBW8, BSA, Birds of Costa Rica
Passeriformes	Thamnophilidae	*Cercomacra nigricans*	2.0	2	HBW8, BSA
Passeriformes	Thamnophilidae	*Cercomacra melanaria*	2.0	2	HBW8, BSA
Passeriformes	Thamnophilidae	*Pyriglena leuconota*	1.4	2	HBW8, BSA
Passeriformes	Thamnophilidae	*Pyriglena leucoptera*	2.0	2	HBW8, BSA
Passeriformes	Thamnophilidae	*Myrmoborus leucophrys*	2.0	2	HBW8, BSA
Passeriformes	Thamnophilidae	*Myrmoborus myotherinus*	2.0	2	HBW8, BSA
Passeriformes	Thamnophilidae	*Hypocnemis cantator*	2.0	2	HBW8, BSA
Passeriformes	Thamnophilidae	*Hypocnemoides melanopogon*	2.0	2	HBW8, BSA
Passeriformes	Thamnophilidae	*Gymnocichla nudiceps*	2.0	2	HBW8, BSA, Birds of Costa Rica
Passeriformes	Thamnophilidae	*Percnostola rufifrons*	1.4	2	HBW8, BSA, Birds of Venezuela
Passeriformes	Thamnophilidae	*Myrmeciza longipes*	2.0	2	HBW8, BSA
Passeriformes	Thamnophilidae	*Myrmeciza exsul*	2.0	2	HBW8, BSA, Birds of Costa Rica
Passeriformes	Thamnophilidae	*Myrmeciza ferruginea*	2.0	2	HBW8, BSA
Passeriformes	Thamnophilidae	*Myrmeciza ruficauda*	2.0	2	HBW8, BSA
Passeriformes	Thamnophilidae	*Myrmeciza loricata*	2.0	2	HBW8, BSA
Passeriformes	Thamnophilidae	*Myrmeciza laemosticta*	2.0	2	HBW8, BSA, Birds of Costa Rica
Passeriformes	Thamnophilidae	*Myrmeciza hemimelaena*	2.0	2	HBW8, BSA
Passeriformes	Thamnophilidae	*Myrmeciza hyperythra*	2.0	2	HBW8, BSA
Passeriformes	Thamnophilidae	*Myrmeciza melanoceps*	2.0	2	HBW8, BSA
Passeriformes	Thamnophilidae	*Myrmeciza goeldii*	1.4	2	HBW8, BSA
Passeriformes	Thamnophilidae	*Myrmeciza fortis*	2.0	2	HBW8, BSA
Passeriformes	Thamnophilidae	*Pithys albifrons*	2.0	2	HBW8, BSA
Passeriformes	Thamnophilidae	*Gymnopithys rufigula*	2.0	2	HBW8, BSA, Birds of Venezuela
Passeriformes	Thamnophilidae	*Gymnopithys leucaspis*	2.0	2	HBW8, BSA, Birds of Costa Rica
Passeriformes	Thamnophilidae	*Gymnopithys salvini*	2.0	2	HBW8, BSA
Passeriformes	Thamnophilidae	*Myrmornis torquata*	1.0	2	HBW8, BSA
Passeriformes	Thamnophilidae	*Rhegmatorhina melanosticta*	2.0	2	HBW8, BSA
Passeriformes	Thamnophilidae	*Hylophylax naevioides*	2.0	2	HBW8, BSA, Birds of Costa Rica
Passeriformes	Thamnophilidae	*Hylophylax naevia*	1.7	2	HBW8, BSA
Passeriformes	Thamnophilidae	*Hylophylax punctulata*	2.0	2	HBW8, BSA
Passeriformes	Thamnophilidae	*Phlegopsis nigromaculata*	2.0	2	HBW8, BSA
Passeriformes	Thamnophilidae	*Phaenostictus mcleannani*	1.4	2	HBW8, BSA, Birds of Costa Rica
Passeriformes	Furnariidae	*Geobates poecilopterus*	3.0	2	HBW8, BSA, BHA
Passeriformes	Furnariidae	*Geositta cunicularia*	2.4	2	HBW8, BSA, BHA
Passeriformes	Furnariidae	*Geositta peruviana*	2.0	2	HBW8, BSA, BHA
Passeriformes	Furnariidae	*Geositta punensis*	2.4	2	HBW8, BSA, BHA
Passeriformes	Furnariidae	*Geositta isabellina*	3.0	2	HBW8, BSA, BHA
Passeriformes	Furnariidae	*Geositta antarctica*	3.0	2	HBW8, BSA, BHA
Passeriformes	Furnariidae	*Geositta rufipennis*	2.4	2	HBW8, BSA, BHA
Passeriformes	Furnariidae	*Geositta tenuirostris*	2.0	2	HBW8, BSA, BHA
Passeriformes	Furnariidae	*Upucerthia certhioides*	3.5	2	HBW8, BSA
Passeriformes	Furnariidae	*Upucerthia ruficauda*	2.0	2	HBW8, BSA

Order	Family	Species	Clutch	Range	Traits
Passeriformes	Furnariidae	*Upucerthia andaecola*	2.0	2	HBW8, BSA
Passeriformes	Furnariidae	*Upucerthia dumetaria*	2.8	2	HBW8, BSA
Passeriformes	Furnariidae	*Upucerthia validirostris*	2.0	2	HBW8, BSA
Passeriformes	Furnariidae	*Cinclodes fuscus*	2.4	2	HBW8, BSA, Birds of Venezuela
Passeriformes	Furnariidae	*Cinclodes comechingonus*	2.0	2	HBW8, BSA
Passeriformes	Furnariidae	*Cinclodes oustaleti*	2.4	2	HBW8, BSA
Passeriformes	Furnariidae	*Cinclodes excelsior*	2.0	2	HBW8, BSA, BHA
Passeriformes	Furnariidae	*Cinclodes patagonicus*	2.4	2	HBW8, BSA, BHA
Passeriformes	Furnariidae	*Cinclodes nigrofumosus*	3.5	2	HBW8, BSA
Passeriformes	Furnariidae	*Cinclodes antarcticus*	2.4	2	HBW8, BSA
Passeriformes	Furnariidae	*Cinclodes atacamensis*	2.0	2	HBW8, BSA
Passeriformes	Furnariidae	*Chilia melanura*	3.5	2	HBW8, BSA
Passeriformes	Furnariidae	*Furnarius minor*	4.0	2	HBW8, BSA
Passeriformes	Furnariidae	*Furnarius figulus*	2.0	2	HBW8, BSA
Passeriformes	Furnariidae	*Furnarius leucopus*	2.0	2	HBW8, BSA
Passeriformes	Furnariidae	*Furnarius torridus*	4.0	2	HBW8, BSA
Passeriformes	Furnariidae	*Furnarius rufus*	3.2	2	HBW8, BSA
Passeriformes	Furnariidae	*Furnarius cristatus*	4.0	2	HBW8, BSA
Passeriformes	Furnariidae	*Sylviorthorhynchus desmursii*	3.5	2	HBW8, BSA, BHA
Passeriformes	Furnariidae	*Aphrastura spinicauda*	3.5	2	HBW8, BSA, BHA
Passeriformes	Furnariidae	*Leptasthenura fuliginiceps*	2.4	2	HBW8, BSA
Passeriformes	Furnariidae	*Leptasthenura platensis*	2.8	2	HBW8, BSA
Passeriformes	Furnariidae	*Leptasthenura aegithaloides*	2.8	2	HBW8, BSA
Passeriformes	Furnariidae	*Leptasthenura andicola*	2.0	2	HBW8, BSA
Passeriformes	Furnariidae	*Schizoeaca fuliginosa*	2.0	2	HBW8, BSA
Passeriformes	Furnariidae	*Schizoeaca harterti*	2.0	2	HBW8, BSA, BHA
Passeriformes	Furnariidae	*Schoeniophylax phryganophila*	4.5	2	HBW8, BSA
Passeriformes	Furnariidae	*Synallaxis infuscata*	2.0	2	HBW8, BSA
Passeriformes	Furnariidae	*Synallaxis ruficapilla*	2.4	2	HBW8, BSA
Passeriformes	Furnariidae	*Synallaxis frontalis*	3.5	2	HBW8, BSA
Passeriformes	Furnariidae	*Synallaxis azarae*	2.8	2	HBW8, BSA
Passeriformes	Furnariidae	*Synallaxis albescens*	2.8	2	HBW8, BSA, Birds of Costa Rica
Passeriformes	Furnariidae	*Synallaxis spixi*	3.9	2	HBW8, BSA
Passeriformes	Furnariidae	*Synallaxis brachyura*	2.4	2	HBW8, BSA, Birds of Costa Rica
Passeriformes	Furnariidae	*Synallaxis subpudica*	1.0	2	HBW8, BSA
Passeriformes	Furnariidae	*Synallaxis cinerascens*	3.0	2	HBW8, BSA
Passeriformes	Furnariidae	*Synallaxis gujanensis*	2.4	2	HBW8, BSA
Passeriformes	Furnariidae	*Synallaxis rutilans*	3.5	2	HBW8, BSA
Passeriformes	Furnariidae	*Synallaxis zimmeri*	2.0	2	HBW8, BSA
Passeriformes	Furnariidae	*Synallaxis erythrothorax*	2.8	2	HBW8, BMx
Passeriformes	Furnariidae	*Synallaxis cinnamomea*	3.0	2	HBW8, BSA
Passeriformes	Furnariidae	*Synallaxis candei*	3.5	2	HBW8, BSA
Passeriformes	Furnariidae	*Synallaxis scutata*	2.4	2	HBW8, BSA
Passeriformes	Furnariidae	*Cranioleuca pyrrhophia*	2.4	2	HBW8, BSA
Passeriformes	Furnariidae	*Cranioleuca vulpina*	2.4	2	HBW8, BSA
Passeriformes	Furnariidae	*Cranioleuca sulphurifera*	2.8	2	HBW8, BSA
Passeriformes	Furnariidae	*Certhiaxis cinnamomea*	3.2	2	HBW8, BSA
Passeriformes	Furnariidae	*Asthenes pyrrholeuca*	2.8	2	HBW8, BSA
Passeriformes	Furnariidae	*Asthenes baeri*	3.9	2	HBW8, BSA
Passeriformes	Furnariidae	*Asthenes modesta*	2.8	2	HBW8, BSA
Passeriformes	Furnariidae	*Asthenes dorbignyi*	2.0	2	HBW8, BSA, BHA
Passeriformes	Furnariidae	*Asthenes steinbachi*	2.4	2	HBW8, BSA
Passeriformes	Furnariidae	*Asthenes humicola*	3.5	2	HBW8, BSA
Passeriformes	Furnariidae	*Asthenes patagonica*	3.5	2	HBW8, BSA
Passeriformes	Furnariidae	*Asthenes wyatti*	2.4	2	HBW8, BSA
Passeriformes	Furnariidae	*Asthenes sclateri*	2.4	2	HBW8, BSA
Passeriformes	Furnariidae	*Asthenes hudsoni*	3.2	2	HBW8, BSA
Passeriformes	Furnariidae	*Thripophaga macroura*	3.0	2	HBW8, BSA
Passeriformes	Furnariidae	*Spartonoica maluroides*	3.5	2	HBW8, BSA
Passeriformes	Furnariidae	*Phleocryptes melanops*	2.8	2	HBW8, BSA, BHA
Passeriformes	Furnariidae	*Limnornis curvirostris*	2.0	2	HBW8, BSA
Passeriformes	Furnariidae	*Limnornis rectirostris*	2.4	2	HBW8, BSA
Passeriformes	Furnariidae	*Anumbius annumbi*	3.9	2	HBW8, BSA

Order	Family	*Species*	Clutch	Range	Traits
Passeriformes	Furnariidae	*Coryphistera alaudina*	3.9	2	HBW8, BSA
Passeriformes	Furnariidae	*Eremobius phoenicurus*	2.8	2	HBW8, BSA
Passeriformes	Furnariidae	*Premnoplex brunnescens*	2.0	2	HBW8, BSA, Birds of Venezuela, Birds of Costa Rica
Passeriformes	Furnariidae	*Lochmias nematura*	2.0	2	HBW8, BSA
Passeriformes	Furnariidae	*Pseudoseisura lophotes*	2.8	2	HBW8, BSA
Passeriformes	Furnariidae	*Pseudoseisura gutturalis*	3.9	2	HBW8, BSA
Passeriformes	Furnariidae	*Pseudocolaptes lawrencii*	1.0	2	HBW8, BSA, Birds of Costa Rica
Passeriformes	Furnariidae	*Syndactyla rufosuperciliata*	2.8	2	HBW8, BSA
Passeriformes	Furnariidae	*Anabacerthia variegaticeps*	2.0	2	HBW8, BSA, Birds of Costa Rica
Passeriformes	Furnariidae	*Anabacerthia striaticollis*	2.0	2	HBW8, BSA
Passeriformes	Furnariidae	*Philydor erythrocercus*	2.0	2	HBW8, BSA
Passeriformes	Furnariidae	*Philydor rufus*	2.4	2	HBW8, BSA, Birds of Venezuela, Birds of Costa Rica
Passeriformes	Furnariidae	*Thripadectes rufobrunneus*	2.0	2	HBW8, Birds of Costa Rica
Passeriformes	Furnariidae	*Thripadectes virgaticeps*	2.0	2	HBW8, BSA, BHA
Passeriformes	Furnariidae	*Thripadectes melanorhynchus*	3.0	2	HBW8, BSA
Passeriformes	Furnariidae	*Automolus ochrolaemus*	2.4	2	HBW8, BSA, Birds of Venezuela, Birds of Costa Rica
Passeriformes	Furnariidae	*Automolus leucophthalmus*	3.5	2	HBW8, BSA
Passeriformes	Furnariidae	*Automolus rubiginosus*	2.0	2	HBW8, BSA, Birds of Costa Rica
Passeriformes	Furnariidae	*Sclerurus mexicanus*	2.0	2	HBW8, BSA, Birds of Costa Rica
Passeriformes	Furnariidae	*Sclerurus albigularis*	2.0	2	HBW8, BSA, Birds of Costa Rica
Passeriformes	Furnariidae	*Sclerurus caudacutus*	2.0	2	HBW8, BSA
Passeriformes	Furnariidae	*Sclerurus scansor*	2.4	2	HBW8, BSA
Passeriformes	Furnariidae	*Sclerurus guatemalensis*	2.0	2	HBW8, BSA, Birds of Costa Rica
Passeriformes	Furnariidae	*Xenops minutus*	2.0	2	HBW8, BSA, Birds of Costa Rica
Passeriformes	Furnariidae	*Xenops rutilans*	2.4	2	HBW8, BSA, Birds of Costa Rica
Passeriformes	Furnariidae	*Pygarrhichas albogularis*	2.4	2	HBW8, BSA
Passeriformes	Furnariidae	*Drymornis bridgesii*	2.4	2	HBW8, BSA
Passeriformes	Furnariidae	*Dendrocincla fuliginosa*	1.7	2	HBW8, BSA, Birds of Venezuela, Birds of Costa Rica
Passeriformes	Furnariidae	*Dendrocincla turdina*	2.0	2	HBW8, BSA
Passeriformes	Furnariidae	*Dendrocincla anabatina*	2.0	2	HBW8, Birds of Costa Rica
Passeriformes	Furnariidae	*Dendrocincla homochroa*	2.4	2	HBW8, BSA, Birds of Costa Rica
Passeriformes	Furnariidae	*Deconychura longicauda*	2.0	2	HBW8, BSA, Birds of Costa Rica
Passeriformes	Furnariidae	*Sittasomus griseicapillus*	3.0	2	HBW8, BSA, Birds of Venezuela, Birds of Costa Rica
Passeriformes	Furnariidae	*Glyphorynchus spirurus*	1.7	2	HBW8, BSA, Birds of Costa Rica
Passeriformes	Furnariidae	*Dendrexetastes rufigula*	2.4	2	HBW8, BSA
Passeriformes	Furnariidae	*Xiphocolaptes albicollis*	2.0	2	HBW8, BSA
Passeriformes	Furnariidae	*Xiphocolaptes major*	2.4	2	HBW8, BSA
Passeriformes	Furnariidae	*Dendrocolaptes certhia*	1.0	2	HBW8, BSA, Birds of Venezuela, Birds of Costa Rica
Passeriformes	Furnariidae	*Dendrocolaptes picumnus*	2.0	2	HBW8, BSA, Birds of Venezuela, Birds of Costa Rica
Passeriformes	Furnariidae	*Xiphorhynchus picus*	1.7	2	HBW8, BSA
Passeriformes	Furnariidae	*Xiphorhynchus obsoletus*	2.0	2	HBW8, BSA, Birds of Venezuela
Passeriformes	Furnariidae	*Xiphorhynchus spixii*	2.0	2	HBW8, BSA
Passeriformes	Furnariidae	*Xiphorhynchus pardalotus*	1.0	2	HBW8, BSA, Birds of Venezuela
Passeriformes	Furnariidae	*Xiphorhynchus guttatus*	1.7	2	HBW8, BSA, Birds of Venezuela, Birds of Costa Rica
Passeriformes	Furnariidae	*Xiphorhynchus susurrans*	2.0	2	HBW8, BSA
Passeriformes	Furnariidae	*Xiphorhynchus flavigaster*	2.4	2	HBW8, Birds of Costa Rica
Passeriformes	Furnariidae	*Xiphorhynchus lachrymosus*	2.0	2	HBW8, BSA, Birds of Costa Rica
Passeriformes	Furnariidae	*Xiphorhynchus erythropygius*	2.0	2	HBW8, BSA, Birds of Costa Rica
Passeriformes	Furnariidae	*Lepidocolaptes souleyetii*	2.0	2	HBW8, BSA, Birds of Costa Rica
Passeriformes	Furnariidae	*Lepidocolaptes angustirostris*	3.5	2	HBW8, BSA
Passeriformes	Furnariidae	*Lepidocolaptes affinis*	2.4	2	HBW8, Birds of Venezuela, Birds of Costa Rica
Passeriformes	Furnariidae	*Lepidocolaptes fuscus*	2.4	2	HBW8, BSA
Passeriformes	Formicariidae	*Formicarius colma*	2.0	2	HBW8, BSA
Passeriformes	Formicariidae	*Formicarius moniliger*	2.0	2	HBW8, BSA, Birds of Costa Rica
Passeriformes	Formicariidae	*Formicarius analis*	2.0	2	HBW8, BSA, Birds of Costa Rica
Passeriformes	Formicariidae	*Formicarius nigricapillus*	2.0	2	HBW8, BSA, Birds of Costa Rica
Passeriformes	Formicariidae	*Chamaeza campanisona*	2.0	2	HBW8, BSA
Passeriformes	Formicariidae	*Pittasoma michleri*	2.0	2	HBW8, BSA, Birds of Costa Rica
Passeriformes	Formicariidae	*Grallaria varia*	2.0	2	HBW8, BSA
Passeriformes	Formicariidae	*Grallaria guatimalensis*	2.0	2	HBW8, BSA, Birds of Costa Rica
Passeriformes	Formicariidae	*Grallaria haplonota*	2.0	2	HBW8, BSA
Passeriformes	Formicariidae	*Grallaria ruficapilla*	2.0	2	HBW8, BSA, BHA
Passeriformes	Formicariidae	*Grallaria rufula*	1.0	2	HBW8, BSA

Order	Family	*Species*	Clutch	Range	Traits
Passeriformes	Formicariidae	*Grallaria erythrotis*	2.0	2	HBW8, BSA
Passeriformes	Formicariidae	*Hylopezus perspicillatus*	2.0	2	HBW8, BSA, Birds of Costa Rica
Passeriformes	Formicariidae	*Myrmothera simplex*	2.0	2	HBW8, BSA
Passeriformes	Formicariidae	*Grallaricula flavirostris*	1.4	2	HBW8, BSA, Birds of Costa Rica, BHA
Passeriformes	Formicariidae	*Grallaricula ferrugineipectus*	2.0	2	HBW8, BSA, BHA
Passeriformes	Conopophagidae	*Conopophaga lineata*	2.0	2	HBW8, BSA
Passeriformes	Conopophagidae	*Conopophaga aurita*	2.0	2	HBW8, BSA
Passeriformes	Conopophagidae	*Conopophaga melanops*	2.0	2	HBW8, BSA
Passeriformes	Rhinocryptidae	*Pteroptochos tarnii*	2.0	2	HBW8, BSA
Passeriformes	Rhinocryptidae	*Pteroptochos castaneus*	2.0	2	HBW8, BSA
Passeriformes	Rhinocryptidae	*Pteroptochos megapodius*	2.0	2	HBW8, BSA
Passeriformes	Rhinocryptidae	*Scelorchilus albicollis*	2.4	2	HBW8, BSA
Passeriformes	Rhinocryptidae	*Scelorchilus rubecula*	2.4	2	HBW8, BSA
Passeriformes	Rhinocryptidae	*Rhinocrypta lanceolata*	1.7	2	HBW8, BSA
Passeriformes	Rhinocryptidae	*Teledromas fuscus*	2.0	2	HBW8, BSA
Passeriformes	Rhinocryptidae	*Eugralla paradoxa*	2.4	2	HBW8, BSA
Passeriformes	Rhinocryptidae	*Scytalopus unicolor*	2.0	2	HBW8, BSA
Passeriformes	Rhinocryptidae	*Scytalopus parvirostris*	2.0	2	HBW8, BHA
Passeriformes	Rhinocryptidae	*Scytalopus macropus*	2.0	2	HBW8, BSA
Passeriformes	Rhinocryptidae	*Scytalopus femoralis*	2.0	2	HBW8, BSA
Passeriformes	Rhinocryptidae	*Scytalopus bolivianus*	2.0	2	HBW8, BSA
Passeriformes	Rhinocryptidae	*Scytalopus atratus*	2.0	2	HBW8, BSA
Passeriformes	Rhinocryptidae	*Scytalopus sanctaemartae*	2.0	2	HBW8, BSA
Passeriformes	Rhinocryptidae	*Scytalopus panamensis*	2.0	2	HBW8, BSA
Passeriformes	Rhinocryptidae	*Scytalopus vicinior*	2.0	2	HBW8, BSA
Passeriformes	Rhinocryptidae	*Scytalopus argentifrons*	2.0	2	HBW8, Birds of Costa Rica
Passeriformes	Rhinocryptidae	*Scytalopus latebricola*	2.0	2	HBW8, BSA
Passeriformes	Rhinocryptidae	*Scytalopus meridanus*	2.0	2	HBW8, BHA
Passeriformes	Rhinocryptidae	*Scytalopus caracae*	2.0	2	HBW8, BSA
Passeriformes	Rhinocryptidae	*Scytalopus spillmani*	2.0	14	HBW8, BSA
Passeriformes	Rhinocryptidae	*Scytalopus zimmeri*	2.4	2	HBW8, HBW
Passeriformes	Rhinocryptidae	*Scytalopus simonsi*	2.0	2	HBW8, BHA
Passeriformes	Rhinocryptidae	*Scytalopus urubambae*	2.4	14	HBW8, HBW
Passeriformes	Rhinocryptidae	*Scytalopus altirostris*	2.4	2	HBW8, HBW
Passeriformes	Rhinocryptidae	*Scytalopus affinis*	2.4	2	HBW8, HBW
Passeriformes	Rhinocryptidae	*Scytalopus canus*	2.0	2	HBW8, BHA
Passeriformes	Rhinocryptidae	*Scytalopus magellanicus*	2.4	2	HBW8, HBW
Passeriformes	Rhinocryptidae	*Scytalopus griseicollis*	2.0	2	HBW8, BSA
Passeriformes	Rhinocryptidae	*Scytalopus superciliaris*	2.0	2	HBW8, BSA
Passeriformes	Rhinocryptidae	*Scytalopus fuscus*	2.0	2	HBW8, BSA
Passeriformes	Rhinocryptidae	*Scytalopus acutirostris*	2.0	2	HBW8, BSA
Passeriformes	Rhinocryptidae	*Scytalopus speluncae*	2.0	2	HBW8, BSA
Passeriformes	Rhinocryptidae	*Scytalopus novacapitalis*	2.0	2	HBW8, BSA
Passeriformes	Rhinocryptidae	*Scytalopus psychopompus*	2.0	14	HBW8, BSA
Passeriformes	Rhinocryptidae	*Scytalopus indigoticus*	2.0	2	HBW8, BSA
Passeriformes	Rhinocryptidae	*Scytalopus schulenbergi*	2.0	2	HBW8, WILSON BULLETIN 106 (4): 585-614 DEC 1994
Passeriformes	Rhinocryptidae	*Scytalopus chocoensis*	2.0	2	HBW8, Orn. Mon. 48: 47
Passeriformes	Climacteridae	*Cormobates leucophaeus*	2.4	10	Handbook of Australian, New Zeeland and Antarctic Birds
Passeriformes	Climacteridae	*Climacteris affinis*	1.7	10	Handbook of Australian, New Zeeland and Antarctic Birds
Passeriformes	Climacteridae	*Climacteris erythrops*	2.0	10	Handbook of Australian, New Zeeland and Antarctic Birds
Passeriformes	Climacteridae	*Climacteris picumnus*	3.0	10	Handbook of Australian, New Zeeland and Antarctic Birds
Passeriformes	Climacteridae	*Climacteris melanura*	2.0	10	Handbook of Australian, New Zeeland and Antarctic Birds
Passeriformes	Climacteridae	*Climacteris rufa*	2.0	10	Handbook of Australian, New Zeeland and Antarctic Birds
Passeriformes	Menuridae	*Menura alberti*	1.0	10	HBW9, Handbook of Australian, New Zeeland and Antarctic Birds
Passeriformes	Menuridae	*Menura novaehollandiae*	1.0	10	HBW9, Handbook of Australian, New Zeeland and Antarctic Birds
Passeriformes	Menuridae	*Atrichornis rufescens*	2.0	10	HBW9, Handbook of Australian, New Zeeland and Antarctic Birds
Passeriformes	Menuridae	*Atrichornis clamosus*	1.0	10	HBW9, Handbook of Australian, New Zeeland and Antarctic Birds
Passeriformes	Ptilonorhynchida	*Ailuroedus buccoides*	1.0	8	BPNG
Passeriformes	Ptilonorhynchida	*Ailuroedus melanotis*	1.4	8,10	BPNG
Passeriformes	Ptilonorhynchida	*Ailuroedus crassirostris*	1.7	10	Complete Book of Australian Birds
Passeriformes	Ptilonorhynchida	*Scenopooetes dentirostris*	2.0	10	Complete Book of Australian Birds
Passeriformes	Ptilonorhynchida	*Prionodura newtoniana*	2.0	10	Complete Book of Australian Birds
Passeriformes	Ptilonorhynchida	*Sericulus chrysocephalus*	2.0	10	Complete Book of Australian Birds

Order	Family	*Species*	Clutch	Range	Traits
Passeriformes	Ptilonorhynchida	*Ptilonorhynchus violaceus*	1.7	10	Complete Book of Australian Birds
Passeriformes	Ptilonorhynchida	*Chlamydera guttata*	2.0	10	Complete Book of Australian Birds
Passeriformes	Ptilonorhynchida	*Chlamydera maculata*	2.4	10	Complete Book of Australian Birds
Passeriformes	Ptilonorhynchida	*Chlamydera nuchalis*	1.4	10	Complete Book of Australian Birds
Passeriformes	Ptilonorhynchida	*Chlamydera lauterbachi*	1.0	8	BPNG
Passeriformes	Ptilonorhynchida	*Chlamydera cerviniventris*	1.0	8,10	BPNG
Passeriformes	Maluridae	*Malurus alboscapulatus*	2.8	8	Fairy-wrens and grasswrens
Passeriformes	Maluridae	*Malurus melanocephalus*	3.5	10	Fairy-wrens and grasswrens
Passeriformes	Maluridae	*Malurus leucopterus*	3.5	10	Fairy-wrens and grasswrens
Passeriformes	Maluridae	*Malurus cyaneus*	2.8	10	Fairy-wrens and grasswrens
Passeriformes	Maluridae	*Malurus splendens*	3.0	10	Fairy-wrens and grasswrens
Passeriformes	Maluridae	*Malurus lamberti*	3.5	10	Fairy-wrens and grasswrens
Passeriformes	Maluridae	*Malurus amabilis*	3.0	10	Fairy-wrens and grasswrens
Passeriformes	Maluridae	*Malurus elegans*	2.4	10	Fairy-wrens and grasswrens
Passeriformes	Maluridae	*Malurus pulcherrimus*	3.0	10	Fairy-wrens and grasswrens
Passeriformes	Maluridae	*Malurus coronatus*	2.4	10	Fairy-wrens and grasswrens
Passeriformes	Maluridae	*Stipiturus ruficeps*	2.4	10	Fairy-wrens and grasswrens
Passeriformes	Maluridae	*Stipiturus malachurus*	3.0	10	Fairy-wrens and grasswrens
Passeriformes	Maluridae	*Stipiturus mallee*	3.0	10	Fairy-wrens and grasswrens
Passeriformes	Maluridae	*Amytornis barbatus*	2.4	10	Fairy-wrens and grasswrens
Passeriformes	Maluridae	*Amytornis woodwardi*	2.0	10	Fairy-wrens and grasswrens
Passeriformes	Maluridae	*Amytornis dorotheae*	2.0	10	Fairy-wrens and grasswrens
Passeriformes	Maluridae	*Amytornis striatus*	2.4	10	Fairy-wrens and grasswrens
Passeriformes	Maluridae	*Amytornis goyderi*	2.4	10	Fairy-wrens and grasswrens
Passeriformes	Maluridae	*Amytornis textilis*	2.0	10	Fairy-wrens and grasswrens
Passeriformes	Maluridae	*Amytornis purnelli*	2.4	10	Fairy-wrens and grasswrens
Passeriformes	Maluridae	*Amytornis housei*	2.0	10	Fairy-wrens and grasswrens
Passeriformes	Meliphagidae	*Myzomela obscura*	2.0	7,8,10	Handbook of Australian, New Zeeland and Antarctic Birds
Passeriformes	Meliphagidae	*Myzomela erythrocephala*	2.0	8,10	Handbook of Australian, New Zeeland and Antarctic Birds
Passeriformes	Meliphagidae	*Myzomela sanguinolenta*	2.0	10	Handbook of Australian, New Zeeland and Antarctic Birds, BWAL
Passeriformes	Meliphagidae	*Myzomela cardinalis*	3.9	9,11	BHTP
Passeriformes	Meliphagidae	*Myzomela jugularis*	2.0	11	BHTP
Passeriformes	Meliphagidae	*Myzomela rosenbergii*	2.0	8	Handbook of Australian, New Zeeland and Antarctic Birds
Passeriformes	Meliphagidae	*Certhionyx pectoralis*	2.0	10	Handbook of Australian, New Zeeland and Antarctic Birds
Passeriformes	Meliphagidae	*Certhionyx niger*	2.0	10	Handbook of Australian, New Zeeland and Antarctic Birds
Passeriformes	Meliphagidae	*Certhionyx variegatus*	2.4	10	Handbook of Australian, New Zeeland and Antarctic Birds
Passeriformes	Meliphagidae	*Timeliopsis fulvigula*	1.0	8	BPNG
Passeriformes	Meliphagidae	*Meliphaga albonotata*	1.4	8	BPNG
Passeriformes	Meliphagidae	*Meliphaga aruensis*	1.4	8	BPNG
Passeriformes	Meliphagidae	*Meliphaga gracilis*	2.0	8,10	Handbook of Australian, New Zeeland and Antarctic Birds
Passeriformes	Meliphagidae	*Meliphaga notata*	2.0	10	Handbook of Australian, New Zeeland and Antarctic Birds
Passeriformes	Meliphagidae	*Meliphaga lewinii*	2.0	10	Handbook of Australian, New Zeeland and Antarctic Birds
Passeriformes	Meliphagidae	*Meliphaga albilineata*	2.0	10	Handbook of Australian, New Zeeland and Antarctic Birds
Passeriformes	Meliphagidae	*Lichenostomus obscurus*	2.0	8	BPNG
Passeriformes	Meliphagidae	*Lichenostomus frenatus*	2.0	10	Handbook of Australian, New Zeeland and Antarctic Birds
Passeriformes	Meliphagidae	*Lichenostomus hindwoodi*	2.0	10	Handbook of Australian, New Zeeland and Antarctic Birds
Passeriformes	Meliphagidae	*Lichenostomus chrysops*	2.4	10	Handbook of Australian, New Zeeland and Antarctic Birds
Passeriformes	Meliphagidae	*Lichenostomus versicolor*	2.0	8,10	Handbook of Australian, New Zeeland and Antarctic Birds
Passeriformes	Meliphagidae	*Lichenostomus fasciogularis*	2.0	10	Handbook of Australian, New Zeeland and Antarctic Birds
Passeriformes	Meliphagidae	*Lichenostomus virescens*	2.0	10	Handbook of Australian, New Zeeland and Antarctic Birds
Passeriformes	Meliphagidae	*Lichenostomus flavus*	1.4	10	Handbook of Australian, New Zeeland and Antarctic Birds
Passeriformes	Meliphagidae	*Lichenostomus unicolor*	2.0	10	Handbook of Australian, New Zeeland and Antarctic Birds
Passeriformes	Meliphagidae	*Lichenostomus leucotis*	2.0	10	Handbook of Australian, New Zeeland and Antarctic Birds
Passeriformes	Meliphagidae	*Lichenostomus flavicollis*	2.4	10	Handbook of Australian, New Zeeland and Antarctic Birds
Passeriformes	Meliphagidae	*Lichenostomus melanops*	2.0	10	Handbook of Australian, New Zeeland and Antarctic Birds
Passeriformes	Meliphagidae	*Lichenostomus cratitius*	2.0	10	Handbook of Australian, New Zeeland and Antarctic Birds
Passeriformes	Meliphagidae	*Lichenostomus keartlandi*	2.0	10	Handbook of Australian, New Zeeland and Antarctic Birds
Passeriformes	Meliphagidae	*Lichenostomus flavescens*	1.4	8,10	Handbook of Australian, New Zeeland and Antarctic Birds
Passeriformes	Meliphagidae	*Lichenostomus fuscus*	1.7	10	Handbook of Australian, New Zeeland and Antarctic Birds
Passeriformes	Meliphagidae	*Lichenostomus plumulus*	2.0	10	Handbook of Australian, New Zeeland and Antarctic Birds
Passeriformes	Meliphagidae	*Lichenostomus ornatus*	2.0	10	Handbook of Australian, New Zeeland and Antarctic Birds
Passeriformes	Meliphagidae	*Lichenostomus penicillatus*	2.4	10	Handbook of Australian, New Zeeland and Antarctic Birds
Passeriformes	Meliphagidae	*Xanthotis flaviventer*	2.0	8,10	Handbook of Australian, New Zeeland and Antarctic Birds

Order	Family	*Species*	Clutch	Range	Traits
Passeriformes	Meliphagidae	*Xanthotis macleayana*	2.0	10	Handbook of Australian, New Zeeland and Antarctic Birds
Passeriformes	Meliphagidae	*Melithreptus lunatus*	2.4	10	Handbook of Australian, New Zeeland and Antarctic Birds
Passeriformes	Meliphagidae	*Melithreptus affinis*	3.0	10	Handbook of Australian, New Zeeland and Antarctic Birds
Passeriformes	Meliphagidae	*Melithreptus albogularis*	2.0	8,10	Handbook of Australian, New Zeeland and Antarctic Birds
Passeriformes	Meliphagidae	*Melithreptus gularis*	2.4	10	Handbook of Australian, New Zeeland and Antarctic Birds
Passeriformes	Meliphagidae	*Melithreptus validirostris*	3.0	10	Handbook of Australian, New Zeeland and Antarctic Birds
Passeriformes	Meliphagidae	*Melithreptus brevirostris*	3.0	10	Handbook of Australian, New Zeeland and Antarctic Birds
Passeriformes	Meliphagidae	*Philemon citreogularis*	2.4	8,10	Handbook of Australian, New Zeeland and Antarctic Birds
Passeriformes	Meliphagidae	*Philemon buceroides*	3.5	7,8,10	Handbook of Australian, New Zeeland and Antarctic Birds
Passeriformes	Meliphagidae	*Philemon argenticeps*	2.4	10	Handbook of Australian, New Zeeland and Antarctic Birds
Passeriformes	Meliphagidae	*Philemon corniculatus*	2.8	8,10	Handbook of Australian, New Zeeland and Antarctic Birds
Passeriformes	Meliphagidae	*Ptiloprora perstriata*	1.0	8	BPNG
Passeriformes	Meliphagidae	*Phylidonyris pyrrhoptera*	2.0	10	Handbook of Australian, New Zeeland and Antarctic Birds
Passeriformes	Meliphagidae	*Phylidonyris novaehollandiae*	2.4	10	Handbook of Australian, New Zeeland and Antarctic Birds
Passeriformes	Meliphagidae	*Phylidonyris nigra*	2.0	10	Handbook of Australian, New Zeeland and Antarctic Birds
Passeriformes	Meliphagidae	*Phylidonyris albifrons*	2.0	10	Handbook of Australian, New Zeeland and Antarctic Birds
Passeriformes	Meliphagidae	*Phylidonyris melanops*	2.0	10	Handbook of Australian, New Zeeland and Antarctic Birds
Passeriformes	Meliphagidae	*Ramsayornis modestus*	2.0	8,10	Handbook of Australian, New Zeeland and Antarctic Birds
Passeriformes	Meliphagidae	*Ramsayornis fasciatus*	1.7	10	Handbook of Australian, New Zeeland and Antarctic Birds
Passeriformes	Meliphagidae	*Plectorhyncha lanceolata*	3.0	10	Handbook of Australian, New Zeeland and Antarctic Birds
Passeriformes	Meliphagidae	*Conopophila albogularis*	2.0	8,10	Handbook of Australian, New Zeeland and Antarctic Birds
Passeriformes	Meliphagidae	*Conopophila rufogularis*	2.4	10	Handbook of Australian, New Zeeland and Antarctic Birds
Passeriformes	Meliphagidae	*Conopophila whitei*	2.0	10	Handbook of Australian, New Zeeland and Antarctic Birds
Passeriformes	Meliphagidae	*Grantiella picta*	2.0	10	Handbook of Australian, New Zeeland and Antarctic Birds
Passeriformes	Meliphagidae	*Xanthomyza phrygia*	2.0	10	Handbook of Australian, New Zeeland and Antarctic Birds
Passeriformes	Meliphagidae	*Acanthorhynchus tenuirostris*	2.0	10	Handbook of Australian, New Zeeland and Antarctic Birds
Passeriformes	Meliphagidae	*Acanthorhynchus superciliosus*	1.4	10	Handbook of Australian, New Zeeland and Antarctic Birds
Passeriformes	Meliphagidae	*Entomyzon cyanotis*	2.0	8,10	Handbook of Australian, New Zeeland and Antarctic Birds
Passeriformes	Meliphagidae	*Manorina melanophrys*	2.0	10	Handbook of Australian, New Zeeland and Antarctic Birds
Passeriformes	Meliphagidae	*Manorina melanocephala*	2.8	10	Handbook of Australian, New Zeeland and Antarctic Birds
Passeriformes	Meliphagidae	*Manorina flavigula*	2.8	10	Handbook of Australian, New Zeeland and Antarctic Birds
Passeriformes	Meliphagidae	*Manorina melanotis*	2.4	10	Handbook of Australian, New Zeeland and Antarctic Birds
Passeriformes	Meliphagidae	*Acanthagenys rufogularis*	2.4	10	Handbook of Australian, New Zeeland and Antarctic Birds
Passeriformes	Meliphagidae	*Anthochaera chrysoptera*	2.0	10	Handbook of Australian, New Zeeland and Antarctic Birds
Passeriformes	Meliphagidae	*Anthochaera carunculata*	2.0	10	Handbook of Australian, New Zeeland and Antarctic Birds
Passeriformes	Meliphagidae	*Anthochaera paradoxa*	2.4	10	Handbook of Australian, New Zeeland and Antarctic Birds
Passeriformes	Meliphagidae	*Epthianura tricolor*	3.5	10	Handbook of Australian, New Zeeland and Antarctic Birds
Passeriformes	Meliphagidae	*Epthianura aurifrons*	2.4	10	Handbook of Australian, New Zeeland and Antarctic Birds
Passeriformes	Meliphagidae	*Epthianura crocea*	3.0	10	Handbook of Australian, New Zeeland and Antarctic Birds
Passeriformes	Meliphagidae	*Epthianura albifrons*	3.0	10	Handbook of Australian, New Zeeland and Antarctic Birds
Passeriformes	Meliphagidae	*Ashbyia lovensis*	2.8	10	Handbook of Australian, New Zeeland and Antarctic Birds
Passeriformes	Pardalotidae	*Pardalotus punctatus*	4.2	10	Complete Book of Australian Birds
Passeriformes	Pardalotidae	*Pardalotus quadragintus*	3.9	10	Complete Book of Australian Birds
Passeriformes	Pardalotidae	*Pardalotus rubricatus*	3.5	10	Complete Book of Australian Birds
Passeriformes	Pardalotidae	*Pardalotus striatus*	3.9	10	Complete Book of Australian Birds
Passeriformes	Pardalotidae	*Dasyornis longirostris*	2.0	10	Complete Book of Australian Birds
Passeriformes	Pardalotidae	*Dasyornis brachypterus*	2.0	10	Complete Book of Australian Birds
Passeriformes	Pardalotidae	*Dasyornis broadbenti*	2.0	10	Complete Book of Australian Birds
Passeriformes	Pardalotidae	*Pycnoptilus floccosus*	1.4	10	Complete Book of Australian Birds
Passeriformes	Pardalotidae	*Origma solitaria*	3.0	10	Complete Book of Australian Birds
Passeriformes	Pardalotidae	*Oreoscopus gutturalis*	2.0	10	Complete Book of Australian Birds
Passeriformes	Pardalotidae	*Sericornis citreogularis*	3.0	10	Complete Book of Australian Birds
Passeriformes	Pardalotidae	*Sericornis frontalis*	2.4	10	Complete Book of Australian Birds
Passeriformes	Pardalotidae	*Sericornis humilis*	2.4	10	EBA
Passeriformes	Pardalotidae	*Sericornis keri*	2.0	10	Complete Book of Australian Birds
Passeriformes	Pardalotidae	*Sericornis beccarii*	2.8	8,10	Complete Book of Australian Birds
Passeriformes	Pardalotidae	*Sericornis magnirostris*	3.5	10	Complete Book of Australian Birds
Passeriformes	Pardalotidae	*Sericornis papuensis*	1.0	8	BPNG
Passeriformes	Pardalotidae	*Acanthornis magnus*	3.5	10	Complete Book of Australian Birds
Passeriformes	Pardalotidae	*Pyrrholaemus brunneus*	3.5	10	Complete Book of Australian Birds
Passeriformes	Pardalotidae	*Chthonicola sagittatus*	3.5	10	Complete Book of Australian Birds
Passeriformes	Pardalotidae	*Calamanthus campestris*	3.5	10	Complete Book of Australian Birds
Passeriformes	Pardalotidae	*Calamanthus fuliginosus*	3.5	10	Complete Book of Australian Birds

Order	Family	*Species*	Clutch	Range	Traits
Passeriformes	Pardalotidae	*Hylacola pyrrhopygia*	2.4	10	Complete Book of Australian Birds
Passeriformes	Pardalotidae	*Hylacola cautus*	2.4	10	Complete Book of Australian Birds
Passeriformes	Pardalotidae	*Acanthiza murina*	2.0	8	BPNG
Passeriformes	Pardalotidae	*Acanthiza katherina*	2.0	10	Complete Book of Australian Birds
Passeriformes	Pardalotidae	*Acanthiza pusilla*	2.4	10	Complete Book of Australian Birds
Passeriformes	Pardalotidae	*Acanthiza ewingii*	3.5	10	Complete Book of Australian Birds
Passeriformes	Pardalotidae	*Acanthiza reguloides*	3.9	10	Complete Book of Australian Birds
Passeriformes	Pardalotidae	*Acanthiza inornata*	4.0	10	Complete Book of Australian Birds
Passeriformes	Pardalotidae	*Acanthiza iredalei*	3.0	10	Complete Book of Australian Birds
Passeriformes	Pardalotidae	*Acanthiza chrysorrhoa*	3.5	10	Complete Book of Australian Birds
Passeriformes	Pardalotidae	*Acanthiza uropygialis*	3.0	10	Complete Book of Australian Birds
Passeriformes	Pardalotidae	*Acanthiza nana*	3.0	10	Complete Book of Australian Birds
Passeriformes	Pardalotidae	*Acanthiza lineata*	3.0	10	Complete Book of Australian Birds
Passeriformes	Pardalotidae	*Acanthiza robustirostris*	2.4	10	Complete Book of Australian Birds
Passeriformes	Pardalotidae	*Smicrornis brevirostris*	2.4	10	Complete Book of Australian Birds
Passeriformes	Pardalotidae	*Gerygone chloronotus*	2.4	8,10	Complete Book of Australian Birds
Passeriformes	Pardalotidae	*Gerygone palpebrosa*	2.4	8,10	Complete Book of Australian Birds
Passeriformes	Pardalotidae	*Gerygone olivacea*	2.4	8,10	Complete Book of Australian Birds
Passeriformes	Pardalotidae	*Gerygone chrysogaster*	3.0	8	BPNG
Passeriformes	Pardalotidae	*Gerygone magnirostris*	2.4	8,10	Complete Book of Australian Birds
Passeriformes	Pardalotidae	*Gerygone tenebrosa*	2.4	10	Complete Book of Australian Birds
Passeriformes	Pardalotidae	*Gerygone sulphurea*	2.4	7,8	Birds of Southeast Asia
Passeriformes	Pardalotidae	*Gerygone inornata*	2.4	7	BWAL
Passeriformes	Pardalotidae	*Gerygone dorsalis*	2.4	7	BWAL
Passeriformes	Pardalotidae	*Gerygone levigaster*	2.4	8,10	Complete Book of Australian Birds
Passeriformes	Pardalotidae	*Gerygone fusca*	2.4	10	Complete Book of Australian Birds
Passeriformes	Pardalotidae	*Gerygone mouki*	2.4	10	Complete Book of Australian Birds
Passeriformes	Pardalotidae	*Gerygone igata*	3.2	12,13	BNZ
Passeriformes	Pardalotidae	*Aphelocephala leucopsis*	3.2	10	Complete Book of Australian Birds
Passeriformes	Pardalotidae	*Aphelocephala pectoralis*	3.0	10	Complete Book of Australian Birds
Passeriformes	Pardalotidae	*Aphelocephala nigricincta*	2.8	10	Complete Book of Australian Birds
Passeriformes	Petroicidae	*Amalocichla incerta*	1.0	8	HBW10, BPNG
Passeriformes	Petroicidae	*Microeca fascinans*	2.4	8,10	Complete Book of Australian Birds
Passeriformes	Petroicidae	*Microeca flavigaster*	1.0	8,10	Complete Book of Australian Birds
Passeriformes	Petroicidae	*Microeca griseoceps*	2.0	8,10	Complete Book of Australian Birds
Passeriformes	Petroicidae	*Microeca flavovirescens*	2.0	8	BPNG
Passeriformes	Petroicidae	*Microeca papuana*	2.0	8	BPNG
Passeriformes	Petroicidae	*Petroica multicolor*	3.5	9,10,11	Complete Book of Australian Birds
Passeriformes	Petroicidae	*Petroica macrocephala*	3.9	12,13	BNZ
Passeriformes	Petroicidae	*Petroica goodenovii*	2.4	10	Complete Book of Australian Birds
Passeriformes	Petroicidae	*Petroica phoenicea*	3.5	10	Complete Book of Australian Birds
Passeriformes	Petroicidae	*Petroica rosea*	2.4	10	Complete Book of Australian Birds
Passeriformes	Petroicidae	*Petroica rodinogaster*	3.5	10	Complete Book of Australian Birds
Passeriformes	Petroicidae	*Petroica australis*	2.8	12,13	BNZ
Passeriformes	Petroicidae	*Melanodryas cucullata*	2.0	10	Complete Book of Australian Birds
Passeriformes	Petroicidae	*Melanodryas vittata*	2.4	10	Complete Book of Australian Birds
Passeriformes	Petroicidae	*Tregellasia capito*	2.0	10	Complete Book of Australian Birds
Passeriformes	Petroicidae	*Tregellasia leucops*	1.4	8,10	Complete Book of Australian Birds
Passeriformes	Petroicidae	*Eopsaltria australis*	2.4	10	Complete Book of Australian Birds
Passeriformes	Petroicidae	*Eopsaltria griseogularis*	2.0	10	Complete Book of Australian Birds
Passeriformes	Petroicidae	*Eopsaltria georgiana*	2.0	10	Complete Book of Australian Birds
Passeriformes	Petroicidae	*Eopsaltria pulverulenta*	2.0	8,10	Complete Book of Australian Birds
Passeriformes	Petroicidae	*Poecilodryas superciliosa*	2.0	10	Complete Book of Australian Birds
Passeriformes	Petroicidae	*Pachycephalopsis poliosoma*	1.0	8	BPNG
Passeriformes	Petroicidae	*Drymodes superciliaris*	1.4	8,10	HBW10, Complete Book of Australian Birds
Passeriformes	Petroicidae	*Drymodes brunneopygia*	1.0	10	HBW10, Complete Book of Australian Birds
Passeriformes	Irenidae	*Irena puella*	2.4	7	HBW10, BIP
Passeriformes	Irenidae	*Chloropsis cochinchinensis*	2.4	7	HBW10, BIP
Passeriformes	Irenidae	*Chloropsis aurifrons*	2.4	7	HBW10, BIP
Passeriformes	Irenidae	*Chloropsis hardwickii*	2.4	7	HBW10, BIP, Birds of Southeast Asia
Passeriformes	Orthonychidae	*Orthonyx temminckii*	1.4	8,10	Complete Book of Australian Birds
Passeriformes	Orthonychidae	*Orthonyx spaldingii*	1.0	10	Complete Book of Australian Birds
Passeriformes	Pomatostomidae	*Pomatostomus temporalis*	2.4	8,10	Complete Book of Australian Birds

Order	Family	*Species*	Clutch	Range	Traits
Passeriformes	Pomatostomidae	*Pomatostomus superciliosus*	2.4	10	Complete Book of Australian Birds
Passeriformes	Pomatostomidae	*Pomatostomus halli*	1.4	10	Complete Book of Australian Birds
Passeriformes	Pomatostomidae	*Pomatostomus ruficeps*	3.9	10	Complete Book of Australian Birds
Passeriformes	Laniidae	*Lanius tigrinus*	5.0	7	HBW10, Shrikes&Bush-shrikes
Passeriformes	Laniidae	*Lanius bucephalus*	5.3	7	HBW10, Shrikes&Bush-shrikes
Passeriformes	Laniidae	*Lanius collurio*	5.5	3,4,5,7	HBW10, Shrikes&Bush-shrikes
Passeriformes	Laniidae	*Lanius isabellinus*	4.5	4,5,7	HBW10, Shrikes&Bush-shrikes
Passeriformes	Laniidae	*Lanius cristatus*	4.9	7,8	HBW10, Shrikes&Bush-shrikes
Passeriformes	Laniidae	*Lanius collurioides*	4.2	7	HBW10, Shrikes&Bush-shrikes
Passeriformes	Laniidae	*Lanius gubernator*	3.5	5	HBW10, Shrikes&Bush-shrikes; EMN
Passeriformes	Laniidae	*Lanius souzae*	3.5	5	HBW10, Shrikes&Bush-shrikes
Passeriformes	Laniidae	*Lanius vittatus*	4.0	4,7	HBW10, Shrikes&Bush-shrikes
Passeriformes	Laniidae	*Lanius schach*	3.5	4,7,8	HBW10, Shrikes&Bush-shrikes
Passeriformes	Laniidae	*Lanius tephronotus*	4.9	7	HBW10, Shrikes&Bush-shrikes
Passeriformes	Laniidae	*Lanius minor*	5.5	3,4,5,7	HBW10, Shrikes&Bush-shrikes
Passeriformes	Laniidae	*Lanius ludovicianus*	4.5	2	HBW10, Shrikes&Bush-shrikes
Passeriformes	Laniidae	*Lanius excubitor*	5.2	2,3,4,7	HBW10, Shrikes&Bush-shrikes; EMN
Passeriformes	Laniidae	*Lanius meridionalis*	5.5	3,4,5	HBW10, Shrikes&Bush-shrikes; EMN
Passeriformes	Laniidae	*Lanius sphenocercus*	6.9	7	HBW10, Shrikes&Bush-shrikes
Passeriformes	Laniidae	*Lanius excubitoroides*	3.0	5	HBW10, Shrikes&Bush-shrikes
Passeriformes	Laniidae	*Lanius cabanisi*	3.5	5	HBW10, Shrikes&Bush-shrikes
Passeriformes	Laniidae	*Lanius dorsalis*	3.5	5	HBW10, Shrikes&Bush-shrikes
Passeriformes	Laniidae	*Lanius somalicus*	3.5	5	HBW10, Shrikes&Bush-shrikes
Passeriformes	Laniidae	*Lanius mackinnoni*	2.4	5	HBW10, Shrikes&Bush-shrikes
Passeriformes	Laniidae	*Lanius collaris*	3.5	5	HBW10, Shrikes&Bush-shrikes
Passeriformes	Laniidae	*Lanius senator*	5.5	3,4,5	HBW10, Shrikes&Bush-shrikes
Passeriformes	Laniidae	*Lanius nubicus*	4.6	3,4,5	HBW10, Shrikes&Bush-shrikes
Passeriformes	Laniidae	*Corvinella corvina*	3.9	5	HBW10, Shrikes&Bush-shrikes
Passeriformes	Laniidae	*Corvinella melanoleuca*	3.9	5	HBW10, Shrikes&Bush-shrikes
Passeriformes	Laniidae	*Eurocephalus rueppelli*	2.8	5	HBW10, Shrikes&Bush-shrikes
Passeriformes	Laniidae	*Eurocephalus anguitimens*	3.5	5	HBW10, Shrikes&Bush-shrikes
Passeriformes	Vireonidae	*Vireo bellii*	3.9	2	BMx
Passeriformes	Vireonidae	*Vireo atricapillus*	3.9	2	BMx
Passeriformes	Vireonidae	*Vireo huttoni*	3.9	2	BMx
Passeriformes	Vireonidae	*Vireo carmioli*	2.0	2	Birds of Costa Rica
Passeriformes	Vireonidae	*Vireo griseus*	3.9	2,13	Birds of Costa Rica, Birds of the West Indies
Passeriformes	Vireonidae	*Vireo pallens*	2.4	2	Birds of Costa Rica
Passeriformes	Vireonidae	*Vireo gundlachii*	3.0	2	Birds of the West Indies
Passeriformes	Vireonidae	*Vireo crassirostris*	2.4	2	Birds of the West Indies
Passeriformes	Vireonidae	*Vireo vicinior*	3.9	2	BMx
Passeriformes	Vireonidae	*Vireo modestus*	2.4	2	Birds of the West Indies
Passeriformes	Vireonidae	*Vireo nanus*	2.0	2	Birds of the West Indies
Passeriformes	Vireonidae	*Vireo latimeri*	3.0	2	Birds of the West Indies
Passeriformes	Vireonidae	*Vireo cassinii*	3.9	2	BMx
Passeriformes	Vireonidae	*Vireo plumbeus*	3.9	2	BMx
Passeriformes	Vireonidae	*Vireo solitarius*	3.9	2	Birds of Costa Rica
Passeriformes	Vireonidae	*Vireo flavifrons*	3.9	2	EMN, Birds of Costa Rica
Passeriformes	Vireonidae	*Vireo philadelphicus*	3.9	2	EMN, Birds of Costa Rica
Passeriformes	Vireonidae	*Vireo olivaceus*	3.9	2	EMN, Birds of Costa Rica
Passeriformes	Vireonidae	*Vireo flavoviridis*	2.4	2	BSA, Birds of Costa Rica
Passeriformes	Vireonidae	*Vireo altiloquus*	2.4	2	Birds of Venezuela; BSA, Birds of Costa Rica, Birds of the West Indies
Passeriformes	Vireonidae	*Vireo magister*	2.0	2	Birds of the West Indies
Passeriformes	Vireonidae	*Vireo gilvus*	3.9	2	Birds of Venezuela, Birds of Costa Rica
Passeriformes	Corvidae	*Psophodes olivaceus*	2.0	10	Complete Book of Australian Birds
Passeriformes	Corvidae	*Psophodes nigrogularis*	2.0	10	Complete Book of Australian Birds
Passeriformes	Corvidae	*Psophodes occidentalis*	2.4	10	Complete Book of Australian Birds
Passeriformes	Corvidae	*Psophodes cristatus*	2.4	10	Complete Book of Australian Birds
Passeriformes	Corvidae	*Cinclosoma punctatum*	2.4	10	Complete Book of Australian Birds
Passeriformes	Corvidae	*Cinclosoma castanotus*	2.0	10	Complete Book of Australian Birds
Passeriformes	Corvidae	*Cinclosoma castaneothorax*	2.4	10	Complete Book of Australian Birds
Passeriformes	Corvidae	*Cinclosoma cinnamomeum*	2.4	10	Complete Book of Australian Birds
Passeriformes	Corvidae	*Corcorax melanorhamphos*	3.9	10	Complete Book of Australian Birds
Passeriformes	Corvidae	*Struthidea cinerea*	3.2	10	Complete Book of Australian Birds

Order	Family	*Species*	Clutch	Range	Traits
Passeriformes	Corvidae	*Falcunculus frontatus*	2.4	10	Complete Book of Australian Birds
Passeriformes	Corvidae	*Oreoica gutturalis*	3.5	10	Complete Book of Australian Birds
Passeriformes	Corvidae	*Pachycare flavogrisea*	2.0	8	BPNG
Passeriformes	Corvidae	*Pachycephala olivacea*	2.4	10	Complete Book of Australian Birds
Passeriformes	Corvidae	*Pachycephala rufogularis*	2.4	10	Complete Book of Australian Birds
Passeriformes	Corvidae	*Pachycephala inornata*	2.8	10	Complete Book of Australian Birds
Passeriformes	Corvidae	*Pachycephala grisola*	2.0	7	BIP
Passeriformes	Corvidae	*Pachycephala hyperythra*	2.0	8	BPNG
Passeriformes	Corvidae	*Pachycephala philippinensis*	2.4	7	Birds of Phillippines
Passeriformes	Corvidae	*Pachycephala simplex*	2.0	10	Complete Book of Australian Birds
Passeriformes	Corvidae	*Pachycephala pectoralis*	1.4	8,9,10,11	Complete Book of Australian Birds, BWAL
Passeriformes	Corvidae	*Pachycephala soror*	1.4	8	BPNG
Passeriformes	Corvidae	*Pachycephala melanura*	2.4	8,9,10	Complete Book of Australian Birds
Passeriformes	Corvidae	*Pachycephala schlegelii*	2.0	8	BPNG
Passeriformes	Corvidae	*Pachycephala rufiventris*	2.4	8,9,10	Complete Book of Australian Birds
Passeriformes	Corvidae	*Pachycephala lanioides*	1.4	10	Complete Book of Australian Birds
Passeriformes	Corvidae	*Colluricincla megarhyncha*	2.4	8,10	Complete Book of Australian Birds
Passeriformes	Corvidae	*Colluricincla boweri*	2.0	10	Complete Book of Australian Birds
Passeriformes	Corvidae	*Colluricincla woodwardi*	2.4	10	Complete Book of Australian Birds
Passeriformes	Corvidae	*Colluricincla harmonica*	2.8	8,10	Complete Book of Australian Birds
Passeriformes	Corvidae	*Platylophus galericulatus*	1.4	7	Crows&Jays; Birds of Southeast Asia
Passeriformes	Corvidae	*Platysmurus leucopterus*	2.4	7	Crows&Jays
Passeriformes	Corvidae	*Gymnorhinus cyanocephalus*	3.5	2	Crows&Jays
Passeriformes	Corvidae	*Cyanocitta cristata*	4.5	2	Crows&Jays
Passeriformes	Corvidae	*Cyanocitta stelleri*	4.0	2	Crows&Jays
Passeriformes	Corvidae	*Aphelocoma californica*	3.5	2	Crows&Jays
Passeriformes	Corvidae	*Aphelocoma coerulescens*	3.5	2	Crows&Jays
Passeriformes	Corvidae	*Aphelocoma ultramarina*	4.0	2	Crows&Jays
Passeriformes	Corvidae	*Aphelocoma unicolor*	3.5	2	Crows&Jays
Passeriformes	Corvidae	*Garrulus glandarius*	4.6	3,4,5,7	Crows&Jays
Passeriformes	Corvidae	*Garrulus lanceolatus*	3.5	7	Crows&Jays
Passeriformes	Corvidae	*Perisoreus infaustus*	3.9	3,7	Crows&Jays
Passeriformes	Corvidae	*Perisoreus canadensis*	4.0	2	Crows&Jays
Passeriformes	Corvidae	*Cyanopica cyana*	5.9	3,7	Crows&Jays
Passeriformes	Corvidae	*Temnurus temnurus*	2.0	7	Crows&Jays; Birds of Southeast Asia
Passeriformes	Corvidae	*Pica pica*	5.3	3,4,5,7	Crows&Jays
Passeriformes	Corvidae	*Pica nuttalli*	6.3	2	Crows&Jays
Passeriformes	Corvidae	*Nucifraga columbiana*	3.0	2	Crows&Jays
Passeriformes	Corvidae	*Nucifraga caryocatactes*	3.5	3,4,7	Crows&Jays
Passeriformes	Corvidae	*Pyrrhocorax pyrrhocorax*	3.9	3,4,5,7	Crows&Jays
Passeriformes	Corvidae	*Pyrrhocorax graculus*	4.2	3,4,5,7	Crows&Jays
Passeriformes	Corvidae	*Ptilostomus afer*	4.6	5	Crows&Jays
Passeriformes	Corvidae	*Corvus monedula*	4.0	3,4,5,7	Crows&Jays
Passeriformes	Corvidae	*Corvus dauuricus*	4.9	7	Crows&Jays
Passeriformes	Corvidae	*Corvus splendens*	4.5	4,5,7,13	Crows&Jays
Passeriformes	Corvidae	*Corvus moneduloides*	2.4	9	Crows&Jays
Passeriformes	Corvidae	*Corvus enca*	2.0	7	Crows&Jays
Passeriformes	Corvidae	*Corvus tristis*	2.0	8	Crows&Jays, BPNG
Passeriformes	Corvidae	*Corvus capensis*	4.2	5	Crows&Jays
Passeriformes	Corvidae	*Corvus frugilegus*	3.7	3,4,7,12,13	Crows&Jays
Passeriformes	Corvidae	*Corvus caurinus*	4.5	2	Crows&Jays
Passeriformes	Corvidae	*Corvus brachyrhynchos*	4.5	2,13	Crows&Jays
Passeriformes	Corvidae	*Corvus ossifragus*	4.5	2	Crows&Jays
Passeriformes	Corvidae	*Corvus imparatus*	4.0	2	Crows&Jays
Passeriformes	Corvidae	*Corvus sinaloae*	4.5	2	Crows&Jays
Passeriformes	Corvidae	*Corvus palmarum*	4.0	2	Crows&Jays
Passeriformes	Corvidae	*Corvus jamaicensis*	3.0	2	Crows&Jays, Birds of the West Indies
Passeriformes	Corvidae	*Corvus nasicus*	4.0	2	Crows&Jays, Birds of the West Indies
Passeriformes	Corvidae	*Corvus leucognaphalus*	3.5	2	Crows&Jays, Birds of the West Indies
Passeriformes	Corvidae	*Corvus corone*	4.5	3,4,5,7	Crows&Jays
Passeriformes	Corvidae	*Corvus macrorhynchos*	4.9	4,7	Crows&Jays
Passeriformes	Corvidae	*Corvus orru*	3.2	7,8,10	Crows&Jays
Passeriformes	Corvidae	*Corvus bennetti*	4.9	10	Crows&Jays

Order	Family	*Species*	Clutch	Range	Traits
Passeriformes	Corvidae	*Corvus coronoides*	3.9	10	Crows&Jays
Passeriformes	Corvidae	*Corvus mellori*	3.9	10	Crows&Jays
Passeriformes	Corvidae	*Corvus tasmanicus*	4.2	10	Crows&Jays
Passeriformes	Corvidae	*Corvus torquatus*	3.5	7	Crows&Jays
Passeriformes	Corvidae	*Corvus hawaiiensis*	5.0	11	Crows&Jays, BPNG
Passeriformes	Corvidae	*Corvus cryptoleucus*	5.9	2	Crows&Jays
Passeriformes	Corvidae	*Corvus albus*	2.6	4,5,6,13	Crows&Jays
Passeriformes	Corvidae	*Corvus ruficollis*	3.7	3,4,5,7	Crows&Jays
Passeriformes	Corvidae	*Corvus corax*	4.6	2,3,4,5,7	Crows&Jays
Passeriformes	Corvidae	*Corvus rhipidurus*	3.5	4,5	Crows&Jays
Passeriformes	Corvidae	*Corvus albicollis*	2.4	5	Crows&Jays
Passeriformes	Corvidae	*Corvus crassirostris*	4.0	5	Crows&Jays
Passeriformes	Corvidae	*Loboparadisea sericea*	1.0	8	Birds of Paradise
Passeriformes	Corvidae	*Lycocorax pyrrhopterus*	1.0	7	Birds of Paradise
Passeriformes	Corvidae	*Epimachus meyeri*	1.0	8	Birds of Paradise
Passeriformes	Corvidae	*Epimachus albertisi*	1.4	8	Birds of Paradise
Passeriformes	Corvidae	*Lophorina superba*	2.0	8	Birds of Paradise
Passeriformes	Corvidae	*Ptiloris magnificus*	2.0	8,10	Birds of Paradise
Passeriformes	Corvidae	*Ptiloris intercedens*	2.0	8	Birds of Paradise
Passeriformes	Corvidae	*Ptiloris victoriae*	2.0	10	Birds of Paradise
Passeriformes	Corvidae	*Ptiloris paradiseus*	2.0	10	Birds of Paradise
Passeriformes	Corvidae	*Seleucidis melanoleuca*	1.0	8	Birds of Paradise
Passeriformes	Corvidae	*Cracticus mentalis*	2.4	8,10	BPNG
Passeriformes	Corvidae	*Cracticus torquatus*	3.9	10	CBAB
Passeriformes	Corvidae	*Cracticus cassicus*	2.4	8	BPNG
Passeriformes	Corvidae	*Cracticus nigrogularis*	3.9	10	Complete Book of Australian Birds
Passeriformes	Corvidae	*Cracticus quoyi*	2.0	8,10	BPNG
Passeriformes	Corvidae	*Strepera graculina*	3.0	10	Complete Book of Australian Birds
Passeriformes	Corvidae	*Strepera fuliginosa*	2.8	10	Complete Book of Australian Birds
Passeriformes	Corvidae	*Strepera versicolor*	2.4	10	Complete Book of Australian Birds
Passeriformes	Corvidae	*Artamus fuscus*	2.4	7	Birds of Southeast Asia, BIP
Passeriformes	Corvidae	*Artamus leucorynchus*	3.5	7,8,9,10,11	Complete Book of Australian Birds; Birds of Southeast Asia, BWAL
Passeriformes	Corvidae	*Artamus mentalis*	2.4	14	EBA
Passeriformes	Corvidae	*Artamus personatus*	2.4	10,12	Complete Book of Australian Birds
Passeriformes	Corvidae	*Artamus superciliosus*	2.4	10,12	Complete Book of Australian Birds
Passeriformes	Corvidae	*Artamus cinereus*	3.5	7,8,10	Complete Book of Australian Birds
Passeriformes	Corvidae	*Artamus cyanopterus*	3.5	10	Complete Book of Australian Birds
Passeriformes	Corvidae	*Artamus minor*	3.0	10	Complete Book of Australian Birds
Passeriformes	Corvidae	*Oriolus szalayi*	1.4	8	BPNG
Passeriformes	Corvidae	*Oriolus sagittatus*	2.8	8,10	Complete Book of Australian Birds
Passeriformes	Corvidae	*Oriolus flavocinctus*	2.4	7,8,10	Complete Book of Australian Birds
Passeriformes	Corvidae	*Oriolus xanthonotus*	2.0	7	Birds of Southeast Asia
Passeriformes	Corvidae	*Oriolus oriolus*	3.5	3,4,5,6,7	BA, EMN
Passeriformes	Corvidae	*Oriolus auratus*	1.7	5	BA
Passeriformes	Corvidae	*Oriolus chinensis*	2.0	7	Birds of Southeast Asia, BIP
Passeriformes	Corvidae	*Oriolus tenuirostris*	2.8	7	Birds of Southeast Asia
Passeriformes	Corvidae	*Oriolus brachyrhynchus*	2.0	5	BA
Passeriformes	Corvidae	*Oriolus larvatus*	2.0	5	BA
Passeriformes	Corvidae	*Oriolus xanthornus*	2.8	7	Birds of Southeast Asia, BIP
Passeriformes	Corvidae	*Oriolus traillii*	2.4	7	Birds of Southeast Asia, BIP
Passeriformes	Corvidae	*Coracina maxima*	2.4	10	HBW10, Compete Book of Australian Birds
Passeriformes	Corvidae	*Coracina macei*	2.4	7	HBW10, Birds of Southeast Asia
Passeriformes	Corvidae	*Coracina javensis*	2.0	7	HBW10, Birds of Southeast Asia
Passeriformes	Corvidae	*Coracina novaehollandiae*	2.4	8,10,12	HBW10, Compete Book of Australian Birds
Passeriformes	Corvidae	*Coracina caeruleogrisea*	1.0	8	HBW10, BPNG
Passeriformes	Corvidae	*Coracina bicolor*	2.0	7	HBW10, BIP
Passeriformes	Corvidae	*Coracina lineata*	2.0	8,9,10	HBW10, Compete Book of Australian Birds
Passeriformes	Corvidae	*Coracina boyeri*	1.0	8	HBW10, BPNG
Passeriformes	Corvidae	*Coracina papuensis*	2.4	7,8,9,10	HBW10, Compete Book of Australian Birds
Passeriformes	Corvidae	*Coracina longicauda*	1.0	8	HBW10, BPNG
Passeriformes	Corvidae	*Coracina pectoralis*	1.4	5	HBW10, BA
Passeriformes	Corvidae	*Coracina caesia*	1.4	5	HBW10, BA
Passeriformes	Corvidae	*Coracina cinerea*	1.0	6,13	HBW10, Guide to the Birds of Madagascar

Order	Family	*Species*	Clutch	Range	Traits
Passeriformes	Corvidae	*Coracina typica*	2.0	13	HBW10, Threatened Birds of Africa and Related Islands
Passeriformes	Corvidae	*Coracina tenuirostris*	1.0	7,8,9,10,11	HBW10, Compete Book of Australian Birds
Passeriformes	Corvidae	*Coracina ostenta*	2.4	7	HBW10, Birds of Phillippines
Passeriformes	Corvidae	*Coracina polioptera*	2.4	7	HBW10, Birds of Southeast Asia
Passeriformes	Corvidae	*Coracina melaschistos*	3.0	7	HBW10, BIP
Passeriformes	Corvidae	*Coracina fimbriata*	2.0	7	HBW10, Birds of Southeast Asia
Passeriformes	Corvidae	*Coracina melanoptera*	2.4	7	HBW10, BIP
Passeriformes	Corvidae	*Lalage nigra*	2.4	7	HBW10, Birds of Southeast Asia
Passeriformes	Corvidae	*Lalage sueurii*	2.0	7,10	HBW10, BBSJB, BPNG
Passeriformes	Corvidae	*Lalage tricolor*	2.0	8,12	HBW10, Compete Book of Australian Birds
Passeriformes	Corvidae	*Lalage leucomela*	1.0	8,10	HBW10, Compete Book of Australian Birds
Passeriformes	Corvidae	*Lalage maculosa*	1.4	9,11	HBW10, BSWP
Passeriformes	Corvidae	*Lalage leucopyga*	2.0	9	HBW10, BSWP
Passeriformes	Corvidae	*Campephaga flava*	1.7	5,8	HBW10, BA
Passeriformes	Corvidae	*Campephaga phoenicea*	2.0	5	HBW10, BA
Passeriformes	Corvidae	*Campephaga quiscalina*	2.0	14	HBW10, BA
Passeriformes	Corvidae	*Pericrocotus roseus*	3.5	7	HBW10, BIP
Passeriformes	Corvidae	*Pericrocotus cinnamomeus*	3.0	7	HBW10, BIP, Birds of the Phillippines
Passeriformes	Corvidae	*Pericrocotus igneus*	2.0	7	HBW10, Birds of Southeast Asia
Passeriformes	Corvidae	*Pericrocotus erythropygius*	3.0	7	HBW10, BIP
Passeriformes	Corvidae	*Pericrocotus solaris*	3.0	7	HBW10, BIP
Passeriformes	Corvidae	*Pericrocotus ethologus*	3.5	7	HBW10, BIP
Passeriformes	Corvidae	*Pericrocotus flammeus*	2.4	7	HBW10, BIP
Passeriformes	Corvidae	*Hemipus picatus*	2.4	7	HBW10, BIP
Passeriformes	Corvidae	*Hemipus hirundinaceus*	2.0	7	HBW10, Birds of Southeast Asia
Passeriformes	Corvidae	*Rhipidura hypoxantha*	3.0	7	HBW11, BIP
Passeriformes	Corvidae	*Rhipidura cyaniceps*	2.0	7	HBW11, Birds of Phillippines
Passeriformes	Corvidae	*Rhipidura albicollis*	3.0	7	HBW11, BIP
Passeriformes	Corvidae	*Rhipidura aureola*	3.0	7	HBW11, BIP
Passeriformes	Corvidae	*Rhipidura javanica*	2.0	7	HBW11, Birds of Southeast Asia
Passeriformes	Corvidae	*Rhipidura perlata*	2.0	7	HBW11, Birds of Southeast Asia
Passeriformes	Corvidae	*Rhipidura leucophrys*	2.8	8,9,10	HBW11, Complete Book of Australian Birds
Passeriformes	Corvidae	*Rhipidura rufiventris*	2.4	7,8,10	HBW11, Complete Book of Australian Birds
Passeriformes	Corvidae	*Rhipidura leucothorax*	2.0	8	HBW11, BPNG
Passeriformes	Corvidae	*Rhipidura atra*	1.0	8	HBW11, BPNG
Passeriformes	Corvidae	*Rhipidura hyperythra*	2.0	8	HBW11, BPNG
Passeriformes	Corvidae	*Rhipidura phasiana*	2.4	8,10	HBW11, Complete Book of Australian Birds
Passeriformes	Corvidae	*Rhipidura fuliginosa*	2.2	8,9,10,12,13	HBW11, Complete Book of Australian Birds, BWAL
Passeriformes	Corvidae	*Rhipidura spilodera*	2.0	9,11	HBW11, BHTP
Passeriformes	Corvidae	*Rhipidura superflua*	2.4	7	HBW11, Complete Book of Australian Birds, BWAL
Passeriformes	Corvidae	*Rhipidura rufidorsa*	2.0	8	HBW11, BPNG
Passeriformes	Corvidae	*Rhipidura rufifrons*	2.4	7,8,9,10,11	HBW11, Complete Book of Australian Birds, BWAL
Passeriformes	Corvidae	*Dicrurus ludwigii*	2.4	5	BA
Passeriformes	Corvidae	*Dicrurus atripennis*	2.0	5	BA
Passeriformes	Corvidae	*Dicrurus adsimilis*	2.4	4,5	BA
Passeriformes	Corvidae	*Dicrurus forficatus*	2.8	6,13	Guide to the Birds of Madagascar
Passeriformes	Corvidae	*Dicrurus macrocercus*	3.2	4,7,11	Birds of Southeast Asia, BIP
Passeriformes	Corvidae	*Dicrurus leucophaeus*	3.5	7	Birds of Southeast Asia, BIP
Passeriformes	Corvidae	*Dicrurus caerulescens*	2.4	7	BIP
Passeriformes	Corvidae	*Dicrurus annectans*	3.5	7	Birds of Southeast Asia, BIP
Passeriformes	Corvidae	*Dicrurus aeneus*	2.8	7	Birds of Southeast Asia, BIP
Passeriformes	Corvidae	*Dicrurus remifer*	2.8	7	Birds of Southeast Asia, BIP
Passeriformes	Corvidae	*Dicrurus hottentottus*	3.2	7,8,10	Birds of Southeast Asia, BIP
Passeriformes	Corvidae	*Dicrurus bracteatus*	3.9	7,8,9	BPNG
Passeriformes	Corvidae	*Dicrurus andamanensis*	2.4	7	Birds of Southeast Asia, BIP
Passeriformes	Corvidae	*Dicrurus paradiseus*	3.5	7	Birds of Southeast Asia, BIP
Passeriformes	Corvidae	*Stenostira scita*	2.4	5	HBW11, BA
Passeriformes	Corvidae	*Erythrocercus mccallii*	2.0	5	HBW11, BA
Passeriformes	Corvidae	*Erythrocercus livingstonei*	2.4	5	HBW11, BA
Passeriformes	Corvidae	*Elminia longicauda*	1.4	5	HBW11, BA
Passeriformes	Corvidae	*Elminia albicauda*	2.0	5	HBW11, BA
Passeriformes	Corvidae	*Elminia nigromitrata*	2.0	5	HBW11, BA
Passeriformes	Corvidae	*Elminia albiventris*	2.0	5	HBW11, BA

Order	Family	*Species*	Clutch	Range	Traits
Passeriformes	Corvidae	*Elminia albonotata*	1.7	5	HBW11, BA
Passeriformes	Corvidae	*Trochocercus nitens*	2.0	5	HBW11, BA
Passeriformes	Corvidae	*Trochocercus cyanomelas*	2.4	5	HBW11, BA
Passeriformes	Corvidae	*Hypothymis azurea*	2.8	7	HBW11, BIP, BWAL
Passeriformes	Corvidae	*Terpsiphone rufiventer*	2.4	5	HBW11, BA
Passeriformes	Corvidae	*Terpsiphone bedfordi*	2.0	5	HBW11, BA
Passeriformes	Corvidae	*Terpsiphone rufocinerea*	1.4	5	HBW11, BA
Passeriformes	Corvidae	*Terpsiphone viridis*	2.2	4,5	HBW11, BA
Passeriformes	Corvidae	*Terpsiphone mutata*	3.0	6,13	HBW11, Guide to the Birds of Madagascar
Passeriformes	Corvidae	*Terpsiphone bourbonnensis*	2.4	13	HBW11, Guide to the Birds of Madagascar
Passeriformes	Corvidae	*Terpsiphone paradisi*	3.5	7	HBW11, BIP, BWAL
Passeriformes	Corvidae	*Terpsiphone atrocaudata*	3.9	7	HBW11, Birds of Southeast Asia
Passeriformes	Corvidae	*Terpsiphone cinnamomea*	3.0	7	HBW11, Birds of Phillippines
Passeriformes	Corvidae	*Chasiempis sandwichensis*	1.7	11	Birds of North America
Passeriformes	Corvidae	*Mayrornis lessoni*	1.4	11	HBW11, BHTP
Passeriformes	Corvidae	*Clytorhynchus vitiensis*	2.0	11	HBW11, BHTP
Passeriformes	Corvidae	*Monarcha axillaris*	1.0	8	HBW11, BPNG
Passeriformes	Corvidae	*Monarcha rubiensis*	1.0	8	HBW11, BPNG
Passeriformes	Corvidae	*Monarcha cinerascens*	1.4	7,8,9	HBW11, BPNG
Passeriformes	Corvidae	*Monarcha frater*	2.4	8,10	HBW11, Complete Book of Australian Birds
Passeriformes	Corvidae	*Monarcha melanopsis*	2.4	8,10	HBW11, Complete Book of Australian Birds
Passeriformes	Corvidae	*Monarcha castaneiventris*	3.0	9	HBW11, BPNG
Passeriformes	Corvidae	*Monarcha leucotis*	2.0	10	HBW11, Complete Book of Australian Birds
Passeriformes	Corvidae	*Monarcha guttulus*	2.0	8	HBW11, BPNG
Passeriformes	Corvidae	*Monarcha trivirgatus*	2.0	7,8,10	HBW11, Complete Book of Australian Birds
Passeriformes	Corvidae	*Monarcha loricatus*	1.4	7	HBW11, Complete Book of Australian Birds
Passeriformes	Corvidae	*Monarcha manadensis*	2.0	8	HBW11, BPNG
Passeriformes	Corvidae	*Monarcha verticalis*	2.0	8	HBW11, BPNG
Passeriformes	Corvidae	*Monarcha barbatus*	2.0	9	HBW11, BPNG
Passeriformes	Corvidae	*Arses telescophthalmus*	2.0	8,10	HBW11, Complete Book of Australian Birds
Passeriformes	Corvidae	*Arses kaupi*	2.0	10	HBW11, Complete Book of Australian Birds
Passeriformes	Corvidae	*Myiagra rubecula*	2.4	8,10	HBW11, Complete Book of Australian Birds
Passeriformes	Corvidae	*Myiagra caledonica*	2.4	9	HBW11, Complete Book of Australian Birds
Passeriformes	Corvidae	*Myiagra vanikorensis*	1.4	9,11	HBW11, BHTP
Passeriformes	Corvidae	*Myiagra azureocapilla*	1.0	11	HBW11, BHTP
Passeriformes	Corvidae	*Myiagra ruficollis*	2.4	7,8,10	HBW11, Complete Book of Australian Birds
Passeriformes	Corvidae	*Myiagra cyanoleuca*	3.0	8,10,12	HBW11, Complete Book of Australian Birds
Passeriformes	Corvidae	*Myiagra inquieta*	2.8	8,10	HBW11, Complete Book of Australian Birds
Passeriformes	Corvidae	*Myiagra alecto*	2.4	8,10	HBW11, Complete Book of Australian Birds
Passeriformes	Corvidae	*Myiagra hebetior*	2.0	8	HBW11, BPNG
Passeriformes	Corvidae	*Lamprolia victoriae*	1.0	11	TBW
Passeriformes	Corvidae	*Machaerirhynchus flaviventer*	2.0	8,10	HBW11, Complete Book of Australian Birds
Passeriformes	Corvidae	*Machaerirhynchus nigripectus*	2.0	8	HBW11, BPNG
Passeriformes	Corvidae	*Aegithina tiphia*	2.8	7	HBW10, BIP
Passeriformes	Corvidae	*Aegithina nigrolutea*	2.4	7	HBW10, BIP
Passeriformes	Corvidae	*Aegithina viridissima*	2.0	7	HBW10, Birds of Southeast Asia
Passeriformes	Corvidae	*Aegithina lafresnayei*	3.0	7	HBW10, Birds of Southeast Asia
Passeriformes	Corvidae	*Nilaus afer*	2.0	5	HBW10, Shrikes&Bush-shrikes
Passeriformes	Corvidae	*Dryoscopus gambensis*	2.0	5	HBW10, Shrikes&Bush-shrikes
Passeriformes	Corvidae	*Dryoscopus pringlii*	2.0	5	HBW10, Shrikes&Bush-shrikes
Passeriformes	Corvidae	*Dryoscopus cubla*	2.0	5	HBW10, Shrikes&Bush-shrikes
Passeriformes	Corvidae	*Dryoscopus senegalensis*	2.0	5	HBW10, Shrikes&Bush-shrikes
Passeriformes	Corvidae	*Tchagra minuta*	2.4	5	HBW10, Shrikes&Bush-shrikes
Passeriformes	Corvidae	*Tchagra senegala*	2.4	3,4,5	HBW10, Shrikes&Bush-shrikes
Passeriformes	Corvidae	*Tchagra australis*	2.4	5	HBW10, Shrikes&Bush-shrikes
Passeriformes	Corvidae	*Tchagra jamesi*	2.4	5	HBW10, Shrikes&Bush-shrikes
Passeriformes	Corvidae	*Tchagra tchagra*	2.0	5	HBW10, Shrikes&Bush-shrikes
Passeriformes	Corvidae	*Laniarius ruficeps*	2.4	5	HBW10, Shrikes&Bush-shrikes
Passeriformes	Corvidae	*Laniarius luehderi*	2.0	5	HBW10, Shrikes&Bush-shrikes
Passeriformes	Corvidae	*Laniarius turatii*	2.0	5	HBW10, Shrikes&Bush-shrikes
Passeriformes	Corvidae	*Laniarius aethiopicus*	3.0	5	HBW10, Shrikes&Bush-shrikes
Passeriformes	Corvidae	*Laniarius bicolor*	2.0	5	HBW10, Shrikes&Bush-shrikes
Passeriformes	Corvidae	*Laniarius ferrugineus*	2.0	5	HBW10, Shrikes&Bush-shrikes

Order	Family	*Species*	Clutch	Range	Traits
Passeriformes	Corvidae	*Laniarius barbarus*	2.0	5	HBW10, Shrikes&Bush-shrikes
Passeriformes	Corvidae	*Laniarius erythrogaster*	2.0	5	HBW10, Shrikes&Bush-shrikes
Passeriformes	Corvidae	*Laniarius atrococcineus*	2.4	5	HBW10, Shrikes&Bush-shrikes
Passeriformes	Corvidae	*Laniarius mufumbiri*	2.0	5	HBW10, Shrikes&Bush-shrikes
Passeriformes	Corvidae	*Laniarius funebris*	2.0	5	HBW10, Shrikes&Bush-shrikes
Passeriformes	Corvidae	*Laniarius leucorhynchus*	2.0	5	HBW10, Shrikes&Bush-shrikes
Passeriformes	Corvidae	*Laniarius poensis*	2.0	5	HBW10, Shrikes&Bush-shrikes
Passeriformes	Corvidae	*Laniarius fuelleborni*	2.0	5	HBW10, Shrikes&Bush-shrikes
Passeriformes	Corvidae	*Rhodophoneus cruentus*	2.4	5	HBW10, Shrikes&Bush-shrikes
Passeriformes	Corvidae	*Telophorus zeylonus*	3.0	5	HBW10, Shrikes&Bush-shrikes
Passeriformes	Corvidae	*Telophorus bocagei*	2.0	5	HBW10, Shrikes&Bush-shrikes
Passeriformes	Corvidae	*Telophorus sulfureopectus*	2.0	5	HBW10, Shrikes&Bush-shrikes
Passeriformes	Corvidae	*Telophorus olivaceus*	2.0	5	HBW10, Shrikes&Bush-shrikes
Passeriformes	Corvidae	*Telophorus viridis*	2.0	5	HBW10, Shrikes&Bush-shrikes
Passeriformes	Corvidae	*Telophorus dohertyi*	3.0	5	HBW10, Shrikes&Bush-shrikes
Passeriformes	Corvidae	*Malaconotus cruentus*	2.4	5	HBW10, Shrikes&Bush-shrikes
Passeriformes	Corvidae	*Malaconotus lagdeni*	2.0	5	HBW10, Shrikes&Bush-shrikes
Passeriformes	Corvidae	*Malaconotus blanchoti*	3.5	5	HBW10, Shrikes&Bush-shrikes
Passeriformes	Corvidae	*Prionops plumatus*	4.0	5	HBW10, Shrikes&Bush-shrikes
Passeriformes	Corvidae	*Prionops poliolophus*	1.0	5	HBW10, Shrikes&Bush-shrikes
Passeriformes	Corvidae	*Prionops retzii*	3.5	5	HBW10, Shrikes&Bush-shrikes
Passeriformes	Corvidae	*Prionops scopifrons*	3.0	5	HBW10, Shrikes&Bush-shrikes
Passeriformes	Corvidae	*Bias musicus*	2.0	5	HBW11, Shrikes&Bush-shrikes
Passeriformes	Corvidae	*Batis mixta*	2.0	5	HBW11, Shrikes&Bush-shrikes
Passeriformes	Corvidae	*Batis capensis*	2.0	5	HBW11, Shrikes&Bush-shrikes
Passeriformes	Corvidae	*Batis fratrum*	2.4	5	HBW11, Shrikes&Bush-shrikes
Passeriformes	Corvidae	*Batis molitor*	2.0	5	HBW11, Shrikes&Bush-shrikes
Passeriformes	Corvidae	*Batis soror*	2.0	5	HBW11, Shrikes&Bush-shrikes
Passeriformes	Corvidae	*Batis pririt*	2.0	5	HBW11, Shrikes&Bush-shrikes
Passeriformes	Corvidae	*Batis senegalensis*	2.0	5	HBW11, Shrikes&Bush-shrikes
Passeriformes	Corvidae	*Batis orientalis*	2.4	5	HBW11, Shrikes&Bush-shrikes
Passeriformes	Corvidae	*Batis minor*	2.0	5	HBW11, Shrikes&Bush-shrikes
Passeriformes	Corvidae	*Batis minulla*	2.0	5	HBW11, Shrikes&Bush-shrikes
Passeriformes	Corvidae	*Platysteira cyanea*	2.0	5	HBW11, Shrikes&Bush-shrikes
Passeriformes	Corvidae	*Platysteira peltata*	2.0	5	HBW11, Shrikes&Bush-shrikes
Passeriformes	Corvidae	*Platysteira castanea*	2.0	14	HBW11, Shrikes&Bush-shrikes
Passeriformes	Corvidae	*Platysteira blissetti*	2.0	14	HBW11, Shrikes&Bush-shrikes
Passeriformes	Corvidae	*Platysteira concreta*	2.0	14	HBW11, Shrikes&Bush-shrikes
Passeriformes	Corvidae	*Philentoma pyrhopterum*	2.0	7	HBW11, Shrikes&Bush-shrikes
Passeriformes	Corvidae	*Philentoma velatum*	2.0	7	HBW11, Shrikes&Bush-shrikes
Passeriformes	Corvidae	*Tephrodornis gularis*	2.8	7	HBW10, Shrikes&Bush-shrikes
Passeriformes	Corvidae	*Tephrodornis pondicerianus*	3.0	7	HBW10, Shrikes&Bush-shrikes
Passeriformes	Corvidae	*Calicalicus madagascariensis*	2.0	6	HBW10, Guide to the Birds of Madagascar
Passeriformes	Corvidae	*Falculea palliata*	4.0	6	HBW10, Guide to the Birds of Madagascar
Passeriformes	Corvidae	*Artamella viridis*	3.0	6	HBW10, Guide to the Birds of Madagascar
Passeriformes	Corvidae	*Leptopterus chabert*	3.0	6	HBW10, Guide to the Birds of Madagascar
Passeriformes	Corvidae	*Tylas eduardi*	2.0	6	HBW10, Guide to the Birds of Madagascar
Passeriformes	Picathartidae	*Chaetops frenatus*	2.0	5	HBW10, BA
Passeriformes	Bombycillidae	*Ptilogonys cinereus*	2.0	2	HBW10, BMx
Passeriformes	Bombycillidae	*Ptilogonys caudatus*	2.0	2	HBW10, Birds of Costa Rica
Passeriformes	Bombycillidae	*Phainopepla nitens*	2.8	2	HBW10, BMx
Passeriformes	Bombycillidae	*Phainoptila melanoxantha*	2.0	2	HBW10, Birds of Costa Rica
Passeriformes	Bombycillidae	*Bombycilla garrulus*	5.5	2,3,4,7	HBW10, BA, EMN
Passeriformes	Bombycillidae	*Bombycilla cedrorum*	3.9	2	HBW10, BA, EMN
Passeriformes	Cinclidae	*Cinclus cinclus*	4.5	3,4,7	HBW10, EMN
Passeriformes	Cinclidae	*Cinclus pallasii*	4.5	7	HBW10, Birds of Southeast Asia
Passeriformes	Cinclidae	*Cinclus mexicanus*	3.2	2	HBW10, Birds of Costa Rica
Passeriformes	Cinclidae	*Cinclus leucocephalus*	2.0	2	HBW10, Birds of Venezuela; BSA
Passeriformes	Cinclidae	*Cinclus schulzi*	2.0	2	HBW10, BSA
Passeriformes	Muscicapidae	*Neocossyphus fraseri*	1.4	5	HBW10, Thrushes
Passeriformes	Muscicapidae	*Neocossyphus rufus*	2.0	5	HBW10, Thrushes
Passeriformes	Muscicapidae	*Pseudocossyphus imerinus*	2.0	6	HBW10, Thrushes
Passeriformes	Muscicapidae	*Monticola rupestris*	2.4	5	HBW10, Thrushes

Order	Family	*Species*	Clutch	Range	Traits
Passeriformes	Muscicapidae	*Monticola explorator*	2.4	5	HBW10, Thrushes
Passeriformes	Muscicapidae	*Monticola brevipes*	2.4	5	HBW10, Thrushes
Passeriformes	Muscicapidae	*Monticola angolensis*	3.5	5	HBW10, Thrushes
Passeriformes	Muscicapidae	*Monticola saxatilis*	3.5	3,4,5,7	HBW10, Thrushes
Passeriformes	Muscicapidae	*Monticola rufocinereus*	3.0	4,5	HBW10, Thrushes
Passeriformes	Muscicapidae	*Monticola cinclorhynchus*	3.5	7	HBW10, Thrushes
Passeriformes	Muscicapidae	*Monticola gularis*	3.9	7	HBW10, Thrushes
Passeriformes	Muscicapidae	*Monticola rufiventris*	3.5	7	HBW10, Thrushes
Passeriformes	Muscicapidae	*Monticola solitarius*	4.2	3,4,5,7,8	HBW10, Thrushes
Passeriformes	Muscicapidae	*Myophonus blighi*	1.4	7	HBW10, Thrushes
Passeriformes	Muscicapidae	*Myophonus glaucinus*	2.0	7	HBW10, Thrushes
Passeriformes	Muscicapidae	*Myophonus robinsoni*	1.4	7	HBW10, Thrushes
Passeriformes	Muscicapidae	*Myophonus horsfieldii*	2.4	7	HBW10, Thrushes
Passeriformes	Muscicapidae	*Myophonus caeruleus*	3.5	7	HBW10, Thrushes
Passeriformes	Muscicapidae	*Myophonus insularis*	2.8	7	HBW10, Thrushes
Passeriformes	Muscicapidae	*Zoothera interpres*	2.4	7	HBW10, Thrushes
Passeriformes	Muscicapidae	*Zoothera wardii*	3.5	7	HBW10, Thrushes
Passeriformes	Muscicapidae	*Zoothera citrina*	3.5	7	HBW10, Thrushes
Passeriformes	Muscicapidae	*Zoothera sibirica*	4.5	4,7	HBW10, Thrushes
Passeriformes	Muscicapidae	*Zoothera naevia*	3.0	2	HBW10, Thrushes
Passeriformes	Muscicapidae	*Zoothera pinicola*	2.0	2	HBW10, Thrushes
Passeriformes	Muscicapidae	*Zoothera piaggiae*	2.0	5	HBW10, Thrushes
Passeriformes	Muscicapidae	*Zoothera gurneyi*	2.4	5	HBW10, Thrushes
Passeriformes	Muscicapidae	*Zoothera princei*	1.7	5	HBW10, Thrushes
Passeriformes	Muscicapidae	*Zoothera guttata*	2.4	5	HBW10, Thrushes
Passeriformes	Muscicapidae	*Zoothera spiloptera*	2.4	7	HBW10, Thrushes
Passeriformes	Muscicapidae	*Zoothera andromedae*	2.4	7	HBW10, Thrushes
Passeriformes	Muscicapidae	*Zoothera mollissima*	4.0	7	HBW10, Thrushes
Passeriformes	Muscicapidae	*Zoothera dixoni*	3.0	7	HBW10, Thrushes
Passeriformes	Muscicapidae	*Zoothera dauma*	3.5	7,8	HBW10, Thrushes
Passeriformes	Muscicapidae	*Zoothera lunulata*	2.4	8,9,10	HBW10, Thrushes
Passeriformes	Muscicapidae	*Zoothera heinei*	2.4	10	HBW10, Thrushes
Passeriformes	Muscicapidae	*Zoothera talaseae*	2.0	8,9	HBW10, Thrushes
Passeriformes	Muscicapidae	*Zoothera monticola*	2.8	7	HBW10, Thrushes
Passeriformes	Muscicapidae	*Zoothera marginata*	3.5	7	HBW10, Thrushes
Passeriformes	Muscicapidae	*Sialia sialis*	4.5	2,13	HBW10, Birds of the West Indies
Passeriformes	Muscicapidae	*Sialia mexicana*	4.9	2	HBW10, BH, BMx
Passeriformes	Muscicapidae	*Sialia currucoides*	5.5	2	HBW10, BH, BMx
Passeriformes	Muscicapidae	*Myadestes obscurus*	1.4	11	HBW10, Thrushes
Passeriformes	Muscicapidae	*Myadestes townsendi*	3.5	2	HBW10, Thrushes
Passeriformes	Muscicapidae	*Myadestes occidentalis*	2.4	2	HBW10, Thrushes
Passeriformes	Muscicapidae	*Myadestes elisabeth*	3.0	2	HBW10, Thrushes
Passeriformes	Muscicapidae	*Myadestes genibarbis*	2.0	2	HBW10, Thrushes
Passeriformes	Muscicapidae	*Myadestes melanops*	2.4	2	HBW10, Thrushes
Passeriformes	Muscicapidae	*Myadestes ralloides*	2.0	2	HBW10, Thrushes
Passeriformes	Muscicapidae	*Entomodestes coracinus*	2.0	2	HBW10, Thrushes
Passeriformes	Muscicapidae	*Catharus gracilirostris*	2.0	2	HBW10, Thrushes
Passeriformes	Muscicapidae	*Catharus aurantiirostris*	2.0	2	HBW10, Thrushes
Passeriformes	Muscicapidae	*Catharus fuscater*	2.0	2	HBW10, Thrushes
Passeriformes	Muscicapidae	*Catharus occidentalis*	2.4	2	HBW10, Thrushes
Passeriformes	Muscicapidae	*Catharus frantzii*	2.0	2	HBW10, Thrushes
Passeriformes	Muscicapidae	*Catharus mexicanus*	2.0	2	HBW10, Thrushes
Passeriformes	Muscicapidae	*Catharus fuscescens*	3.5	2	HBW10, Thrushes
Passeriformes	Muscicapidae	*Catharus minimus*	3.5	2,7	HBW10, Thrushes
Passeriformes	Muscicapidae	*Catharus bicknelli*	3.5	2	HBW10, Thrushes
Passeriformes	Muscicapidae	*Catharus ustulatus*	3.5	2	HBW10, Thrushes
Passeriformes	Muscicapidae	*Catharus guttatus*	3.5	2	HBW10, Thrushes
Passeriformes	Muscicapidae	*Catharus mustelinus*	3.5	2	HBW10, Thrushes
Passeriformes	Muscicapidae	*Psophocichla litsipsirupa*	2.4	5	HBW10, Thrushes
Passeriformes	Muscicapidae	*Turdus pelios*	2.4	5	HBW10, Thrushes
Passeriformes	Muscicapidae	*Turdus tephronotus*	2.4	5	HBW10, Thrushes
Passeriformes	Muscicapidae	*Turdus libonyanus*	2.4	5	HBW10, Thrushes
Passeriformes	Muscicapidae	*Turdus olivaceus*	2.4	5	HBW10, Thrushes

Order	Family	Species	Clutch	Range	Traits
Passeriformes	Muscicapidae	*Turdus ludoviciae*	2.4	14	HBW10, Thrushes
Passeriformes	Muscicapidae	*Turdus helleri*	2.4	14	HBW10, Thrushes
Passeriformes	Muscicapidae	*Turdus menachensis*	2.0	4	HBW10, Thrushes
Passeriformes	Muscicapidae	*Turdus hortulorum*	4.5	7	HBW10, Thrushes
Passeriformes	Muscicapidae	*Turdus unicolor*	3.5	7	HBW10, Thrushes
Passeriformes	Muscicapidae	*Turdus dissimilis*	3.5	7	HBW10, Thrushes
Passeriformes	Muscicapidae	*Turdus cardis*	4.0	7	HBW10, Thrushes
Passeriformes	Muscicapidae	*Turdus albocinctus*	3.5	7	HBW10, Thrushes
Passeriformes	Muscicapidae	*Turdus torquatus*	4.5	3,4,5	HBW10, Thrushes
Passeriformes	Muscicapidae	*Turdus boulboul*	3.5	7	HBW10, Thrushes
Passeriformes	Muscicapidae	*Turdus merula*	3.9	3,4,7,10,12,13	HBW10, Thrushes
Passeriformes	Muscicapidae	*Turdus poliocephalus*	1.7	7,8,9,11	HBW10, Thrushes
Passeriformes	Muscicapidae	*Turdus rubrocanus*	2.8	7	HBW10, Thrushes
Passeriformes	Muscicapidae	*Turdus feae*	4.5	7	HBW10, Thrushes
Passeriformes	Muscicapidae	*Turdus obscurus*	4.9	4,7	HBW10, Thrushes
Passeriformes	Muscicapidae	*Turdus pallidus*	4.5	7	HBW10, Thrushes
Passeriformes	Muscicapidae	*Turdus chrysolaus*	3.2	7	HBW10, Thrushes
Passeriformes	Muscicapidae	*Turdus ruficollis*	4.5	4,7	HBW10, Thrushes
Passeriformes	Muscicapidae	*Turdus naumanni*	3.9	4,7	HBW10, Thrushes
Passeriformes	Muscicapidae	*Turdus pilaris*	5.5	3,4,5,7	HBW10, Thrushes
Passeriformes	Muscicapidae	*Turdus iliacus*	4.9	3,4,7	HBW10, Thrushes
Passeriformes	Muscicapidae	*Turdus philomelos*	3.9	3,4,7,10,12,13	HBW10, Thrushes
Passeriformes	Muscicapidae	*Turdus mupinensis*	2.8	7	HBW10, Thrushes
Passeriformes	Muscicapidae	*Turdus viscivorus*	3.9	3,4,5,7	HBW10, Thrushes
Passeriformes	Muscicapidae	*Turdus aurantius*	2.8	2	HBW10, Thrushes
Passeriformes	Muscicapidae	*Turdus plumbeus*	3.9	2	HBW10, Thrushes
Passeriformes	Muscicapidae	*Turdus nigrescens*	2.0	2	HBW10, Thrushes
Passeriformes	Muscicapidae	*Turdus fuscater*	2.0	2	HBW10, Thrushes
Passeriformes	Muscicapidae	*Turdus serranus*	2.0	2	HBW10, Thrushes
Passeriformes	Muscicapidae	*Turdus nigriceps*	3.0	2	HBW10, Thrushes
Passeriformes	Muscicapidae	*Turdus olivater*	2.4	2	HBW10, Thrushes
Passeriformes	Muscicapidae	*Turdus rufiventris*	2.4	2	HBW10, Thrushes
Passeriformes	Muscicapidae	*Turdus falcklandii*	2.4	2	HBW10, Thrushes
Passeriformes	Muscicapidae	*Turdus leucomelas*	2.4	2	HBW10, Thrushes
Passeriformes	Muscicapidae	*Turdus amaurochalinus*	3.0	2	HBW10, Thrushes
Passeriformes	Muscicapidae	*Turdus plebejus*	2.4	2	HBW10, Thrushes
Passeriformes	Muscicapidae	*Turdus ignobilis*	2.0	2	HBW10, Thrushes
Passeriformes	Muscicapidae	*Turdus obsoletus*	2.4	2	HBW10, Thrushes
Passeriformes	Muscicapidae	*Turdus fumigatus*	2.4	2	HBW10, Thrushes
Passeriformes	Muscicapidae	*Turdus grayi*	2.4	2	HBW10, Thrushes
Passeriformes	Muscicapidae	*Turdus nudigenis*	2.4	2	HBW10, Thrushes
Passeriformes	Muscicapidae	*Turdus maculirostris*	3.0	2	HBW10, Thrushes
Passeriformes	Muscicapidae	*Turdus jamaicensis*	2.4	2	HBW10, Thrushes
Passeriformes	Muscicapidae	*Turdus assimilis*	2.4	2	HBW10, Thrushes
Passeriformes	Muscicapidae	*Turdus albicollis*	2.4	2	HBW10, Thrushes
Passeriformes	Muscicapidae	*Turdus swalesi*	2.4	2	HBW10, Thrushes
Passeriformes	Muscicapidae	*Turdus migratorius*	3.5	2	HBW10, Thrushes
Passeriformes	Muscicapidae	*Turdus rufitorques*	2.4	2	HBW10, Thrushes
Passeriformes	Muscicapidae	*Brachypteryx major*	2.0	7	HBW10, BIS
Passeriformes	Muscicapidae	*Brachypteryx leucophrys*	2.8	7	HBW10, Birds of Southeast Asia
Passeriformes	Muscicapidae	*Brachypteryx montana*	2.8	7	HBW10, Birds of Southeast Asia
Passeriformes	Muscicapidae	*Alethe poliocephala*	1.7	5	HBW10, BA
Passeriformes	Muscicapidae	*Alethe poliophrys*	2.0	5	HBW10, BA
Passeriformes	Muscicapidae	*Alethe fuelleborni*	2.0	5	HBW10, BA
Passeriformes	Muscicapidae	*Alethe diademata*	2.0	5	HBW10, BA
Passeriformes	Muscicapidae	*Empidornis semipartitus*	2.0	5	HBW11, BA
Passeriformes	Muscicapidae	*Bradornis pallidus*	2.4	14	HBW11, BA
Passeriformes	Muscicapidae	*Bradornis infuscatus*	2.4	14	HBW11, BA
Passeriformes	Muscicapidae	*Bradornis mariquensis*	3.0	14	HBW11, BA
Passeriformes	Muscicapidae	*Bradornis microrhynchus*	2.0	14	HBW11, BA
Passeriformes	Muscicapidae	*Dioptrornis chocolatinus*	3.0	5	HBW11, BA
Passeriformes	Muscicapidae	*Melaenornis edolioides*	2.4	5	HBW11, BA
Passeriformes	Muscicapidae	*Melaenornis pammelaina*	2.8	5	HBW11, BA

Order	Family	*Species*	Clutch	Range	Traits
Passeriformes	Muscicapidae	*Melaenornis ardesiacus*	2.0	5	HBW11, BA
Passeriformes	Muscicapidae	*Fraseria ocreata*	2.4	5	HBW11, BA
Passeriformes	Muscicapidae	*Fraseria cinerascens*	1.4	5	HBW11, BA
Passeriformes	Muscicapidae	*Sigelus silens*	3.0	5	HBW11, BA
Passeriformes	Muscicapidae	*Rhinomyias addita*	2.4	7	HBW11, BWAL
Passeriformes	Muscicapidae	*Rhinomyias oscillans*	2.4	7	HBW11, BWAL
Passeriformes	Muscicapidae	*Rhinomyias brunneata*	2.4	7	HBW11, BIP
Passeriformes	Muscicapidae	*Rhinomyias olivacea*	2.4	7	HBW11, Birds of Southeast Asia
Passeriformes	Muscicapidae	*Rhinomyias umbratilis*	2.4	7	HBW11, Birds of Southeast Asia
Passeriformes	Muscicapidae	*Rhinomyias ruficauda*	2.4	7	HBW11, Birds of Phillippines
Passeriformes	Muscicapidae	*Rhinomyias colonus*	2.4	7	HBW11, BWAL
Passeriformes	Muscicapidae	*Rhinomyias gularis*	2.4	7	HBW11, BBSJB
Passeriformes	Muscicapidae	*Rhinomyias insignis*	2.4	7	HBW11, Birds of Phillippines
Passeriformes	Muscicapidae	*Rhinomyias albigularis*	2.4	7	HBW11, Birds of Phillippines
Passeriformes	Muscicapidae	*Rhinomyias goodfellowi*	2.4	7	HBW11, Birds of Phillippines
Passeriformes	Muscicapidae	*Muscicapa striata*	3.5	3,4,5,7	HBW11, BA
Passeriformes	Muscicapidae	*Muscicapa gambagae*	2.0	4,5	HBW11, BA
Passeriformes	Muscicapidae	*Muscicapa griseisticta*	3.9	7,8	HBW11, Birds of Southeast Asia
Passeriformes	Muscicapidae	*Muscicapa sibirica*	3.5	7	HBW11, BIP
Passeriformes	Muscicapidae	*Muscicapa dauurica*	2.8	7	HBW11, EMN, BIP
Passeriformes	Muscicapidae	*Muscicapa ruficauda*	3.5	7	HBW11, BIP
Passeriformes	Muscicapidae	*Muscicapa muttui*	4.5	7	HBW11, BIP
Passeriformes	Muscicapidae	*Muscicapa ferruginea*	2.4	7	HBW11, BIP
Passeriformes	Muscicapidae	*Muscicapa infuscata*	2.4	5	HBW11, BA
Passeriformes	Muscicapidae	*Muscicapa boehmi*	4.0	5	HBW11, BA
Passeriformes	Muscicapidae	*Muscicapa aquatica*	2.4	5	HBW11, BA
Passeriformes	Muscicapidae	*Muscicapa lendu*	2.0	5	HBW11, BA
Passeriformes	Muscicapidae	*Muscicapa adusta*	2.4	5	HBW11, BA
Passeriformes	Muscicapidae	*Muscicapa epulata*	2.0	5	HBW11, BA
Passeriformes	Muscicapidae	*Muscicapa sethsmithi*	2.0	5	HBW11, BA
Passeriformes	Muscicapidae	*Muscicapa comitata*	1.4	5	HBW11, BA
Passeriformes	Muscicapidae	*Muscicapa cassini*	1.7	5	HBW11, BA
Passeriformes	Muscicapidae	*Muscicapa caerulescens*	2.4	5	HBW11, BA
Passeriformes	Muscicapidae	*Myioparus griseigularis*	2.0	5	HBW11, BA
Passeriformes	Muscicapidae	*Myioparus plumbeus*	2.0	5	HBW11, BA
Passeriformes	Muscicapidae	*Ficedula hypoleuca*	5.7	3,4,5	HBW11, BA
Passeriformes	Muscicapidae	*Ficedula albicollis*	5.9	3,4,5	HBW11, BA, EMN
Passeriformes	Muscicapidae	*Ficedula semitorquata*	5.5	3,4,5	HBW11, BA, EMN
Passeriformes	Muscicapidae	*Ficedula zanthopygia*	5.7	7	HBW11
Passeriformes	Muscicapidae	*Ficedula narcissina*	3.9	7	HBW11, Birds of Southeast Asia
Passeriformes	Muscicapidae	*Ficedula mugimaki*	5.7	7	HBW11, Birds of Southeast Asia
Passeriformes	Muscicapidae	*Ficedula hodgsonii*	4.5	7	HBW11, Birds of Southeast Asia
Passeriformes	Muscicapidae	*Ficedula strophiata*	3.5	7	HBW11, BIP
Passeriformes	Muscicapidae	*Ficedula parva*	5.5	3,4,7	HBW11, BA, EMN, BIP
Passeriformes	Muscicapidae	*Ficedula subrubra*	4.5	7	HBW11, BIP
Passeriformes	Muscicapidae	*Ficedula monileger*	4.0	7	HBW11, BIP
Passeriformes	Muscicapidae	*Ficedula solitaris*	2.4	7	HBW11, Birds of Southeast Asia
Passeriformes	Muscicapidae	*Ficedula hyperythra*	2.8	7	HBW11, BIP
Passeriformes	Muscicapidae	*Ficedula dumetoria*	2.0	7	HBW11, Birds of Southeast Asia
Passeriformes	Muscicapidae	*Ficedula rufigula*	2.4	7	HBW11, BWAL
Passeriformes	Muscicapidae	*Ficedula buruensis*	2.4	7	HBW11, BWAL
Passeriformes	Muscicapidae	*Ficedula basilanica*	2.0	7	HBW11, Birds of Phillippines
Passeriformes	Muscicapidae	*Ficedula harterti*	2.4	7	HBW11, BWAL
Passeriformes	Muscicapidae	*Ficedula bonthaina*	2.4	7	NO EGG?
Passeriformes	Muscicapidae	*Ficedula westermanni*	2.0	7	HBW11, BIP
Passeriformes	Muscicapidae	*Ficedula superciliaris*	4.0	7	HBW11, BIP
Passeriformes	Muscicapidae	*Ficedula tricolor*	3.5	7	HBW11, BIP
Passeriformes	Muscicapidae	*Ficedula sapphira*	4.0	7	HBW11, BIP
Passeriformes	Muscicapidae	*Ficedula timorensis*	2.4	7	HBW11, BWAL
Passeriformes	Muscicapidae	*Eumyias thalassina*	3.5	7	HBW11, BIP
Passeriformes	Muscicapidae	*Eumyias sordida*	2.4	7	HBW11, BIP
Passeriformes	Muscicapidae	*Eumyias albicaudata*	3.0	7	HBW11, BIP
Passeriformes	Muscicapidae	*Niltava grandis*	4.0	7	HBW11, BIP

Order	Family	*Species*	Clutch	Range	Traits
Passeriformes	Muscicapidae	*Niltava macgrigoriae*	4.0	7	HBW11, BIP
Passeriformes	Muscicapidae	*Niltava sundara*	4.0	7	HBW11, BIP
Passeriformes	Muscicapidae	*Cyornis concretus*	2.0	7	HBW11, BIP, Birds of Southeast Asia
Passeriformes	Muscicapidae	*Cyornis pallipes*	4.0	7	HBW11, BIP
Passeriformes	Muscicapidae	*Cyornis poliogenys*	3.9	7	HBW11, BIP
Passeriformes	Muscicapidae	*Cyornis unicolor*	4.0	7	HBW11, BIP
Passeriformes	Muscicapidae	*Cyornis rubeculoides*	4.5	7	HBW11, BIP
Passeriformes	Muscicapidae	*Cyornis banyumas*	4.5	7	HBW11, BIP
Passeriformes	Muscicapidae	*Cyornis tickelliae*	3.5	7	HBW11, BIP
Passeriformes	Muscicapidae	*Cyornis rufigastra*	2.0	7	HBW11, Birds of Southeast Asia, Birds of Phillippines
Passeriformes	Muscicapidae	*Culicicapa ceylonensis*	3.5	7	HBW11, BIP
Passeriformes	Muscicapidae	*Pogonocichla stellata*	2.4	5	HBW10, BA
Passeriformes	Muscicapidae	*Swynnertonia swynnertoni*	2.0	5	HBW10, BA
Passeriformes	Muscicapidae	*Stiphrornis erythrothorax*	2.0	5	HBW10, BA
Passeriformes	Muscicapidae	*Sheppardia bocagei*	2.0	5	HBW10, BA
Passeriformes	Muscicapidae	*Sheppardia cyornithopsis*	2.0	5	HBW10, BA
Passeriformes	Muscicapidae	*Sheppardia aequatorialis*	2.0	5	HBW10, BA
Passeriformes	Muscicapidae	*Sheppardia sharpei*	2.0	5	HBW10, BA
Passeriformes	Muscicapidae	*Erithacus rubecula*	4.9	3,4	HBW10, BA, EMN
Passeriformes	Muscicapidae	*Luscinia luscinia*	4.5	3,4,5,7	HBW10, BA, EMN
Passeriformes	Muscicapidae	*Luscinia megarhynchos*	4.5	3,4,5,7	HBW10, BA, EMN
Passeriformes	Muscicapidae	*Luscinia calliope*	4.9	7,8	HBW10, EMN
Passeriformes	Muscicapidae	*Luscinia pectoralis*	3.5	7	HBW10, Birds of Southeast Asia
Passeriformes	Muscicapidae	*Luscinia svecica*	5.5	2,3,4,5,7	HBW10, BA, EMN
Passeriformes	Muscicapidae	*Luscinia ruficeps*	4.0	7	HBW10, Birds of Southeast Asia
Passeriformes	Muscicapidae	*Luscinia obscura*	4.0	7	HBW10, Birds of Southeast Asia
Passeriformes	Muscicapidae	*Luscinia brunnea*	3.5	7	HBW10, Birds of Southeast Asia
Passeriformes	Muscicapidae	*Tarsiger cyanurus*	5.9	3,4,7	HBW10, EMN
Passeriformes	Muscicapidae	*Tarsiger chrysaeus*	3.5	7	HBW10, Birds of Southeast Asia
Passeriformes	Muscicapidae	*Tarsiger indicus*	3.5	7	HBW10, Birds of Southeast Asia
Passeriformes	Muscicapidae	*Irania gutturalis*	4.5	3,4,5	HBW10, EMN
Passeriformes	Muscicapidae	*Cossypha isabellae*	2.0	5	HBW10, BA
Passeriformes	Muscicapidae	*Cossypha archeri*	2.0	5	HBW10, BA
Passeriformes	Muscicapidae	*Cossypha anomala*	2.0	5	HBW10, BA
Passeriformes	Muscicapidae	*Cossypha caffra*	2.4	5	HBW10, BA
Passeriformes	Muscicapidae	*Cossypha humeralis*	3.0	5	HBW10, BA
Passeriformes	Muscicapidae	*Cossypha cyanocampter*	2.0	5	HBW10, BA
Passeriformes	Muscicapidae	*Cossypha semirufa*	2.4	5	HBW10, BA
Passeriformes	Muscicapidae	*Cossypha heuglini*	2.0	5	HBW10, BA
Passeriformes	Muscicapidae	*Cossypha natalensis*	3.0	5	HBW10, BA
Passeriformes	Muscicapidae	*Cossypha dichroa*	3.0	5	HBW10, BA
Passeriformes	Muscicapidae	*Cossypha niveicapilla*	2.4	5	HBW10, BA
Passeriformes	Muscicapidae	*Cossypha albicapilla*	2.0	5	HBW10, BA
Passeriformes	Muscicapidae	*Cichladusa arquata*	2.4	5	HBW10, BA
Passeriformes	Muscicapidae	*Cichladusa ruficauda*	2.0	5	HBW10, BA
Passeriformes	Muscicapidae	*Cichladusa guttata*	2.4	5	HBW10, BA
Passeriformes	Muscicapidae	*Cercotrichas quadrivirgata*	2.4	5	HBW10, BA
Passeriformes	Muscicapidae	*Cercotrichas barbata*	2.4	5	HBW10, BA
Passeriformes	Muscicapidae	*Cercotrichas signata*	2.4	5	HBW10, BA
Passeriformes	Muscicapidae	*Cercotrichas hartlaubi*	2.4	5	HBW10, BA
Passeriformes	Muscicapidae	*Cercotrichas leucophrys*	2.8	5	HBW10, BA
Passeriformes	Muscicapidae	*Cercotrichas galactotes*	3.5	3,4,5	HBW10, BA, EMN
Passeriformes	Muscicapidae	*Cercotrichas paena*	2.8	5	HBW10, BA
Passeriformes	Muscicapidae	*Cercotrichas coryphaeus*	3.0	5	HBW10, BA
Passeriformes	Muscicapidae	*Cercotrichas podobe*	2.8	4,5	HBW10, BA, EMN
Passeriformes	Muscicapidae	*Namibornis herero*	2.0	5	HBW10, BA
Passeriformes	Muscicapidae	*Copsychus albospecularis*	3.0	6	HBW10, Guide to the Birds of Madagascar
Passeriformes	Muscicapidae	*Copsychus saularis*	3.2	7	HBW10, Birds of Southeast Asia
Passeriformes	Muscicapidae	*Copsychus malabaricus*	3.9	7,11	HBW10, Birds of Southeast Asia
Passeriformes	Muscicapidae	*Copsychus luzoniensis*	2.0	7	HBW10, Birds of Phillippines
Passeriformes	Muscicapidae	*Copsychus cebuensis*	2.4	7	HBW10, Birds of Phillippines
Passeriformes	Muscicapidae	*Phoenicurus ochruros*	4.9	3,4,5,7	HBW10, BA, EMN
Passeriformes	Muscicapidae	*Phoenicurus phoenicurus*	5.9	3,4,5,7	HBW10, BA, EMN

Order	Family	Species	Clutch	Range	Traits
Passeriformes	Muscicapidae	*Phoenicurus schisticeps*	3.0	7	HBW10, Birds of Southeast Asia
Passeriformes	Muscicapidae	*Phoenicurus auroreus*	3.5	7	HBW10, Birds of Southeast Asia
Passeriformes	Muscicapidae	*Phoenicurus moussieri*	4.5	3,4	HBW10, BA, EMN
Passeriformes	Muscicapidae	*Phoenicurus erythrogaster*	4.0	3,4,7	HBW10, EMN
Passeriformes	Muscicapidae	*Phoenicurus frontalis*	3.5	7	HBW10, Birds of Southeast Asia
Passeriformes	Muscicapidae	*Chaimarrornis leucocephalus*	3.9	7	HBW10, Birds of Southeast Asia
Passeriformes	Muscicapidae	*Rhyacornis fuliginosus*	3.9	7	HBW10, Birds of Southeast Asia
Passeriformes	Muscicapidae	*Rhyacornis bicolor*	2.8	7	HBW10, Birds of Phillippines
Passeriformes	Muscicapidae	*Hodgsonius phaenicuroides*	2.8	7	HBW10, Birds of Southeast Asia
Passeriformes	Muscicapidae	*Cinclidium leucurum*	3.2	7	HBW10, Birds of Southeast Asia
Passeriformes	Muscicapidae	*Grandala coelicolor*	2.0	7	HBW10, Birds of Southeast Asia
Passeriformes	Muscicapidae	*Enicurus scouleri*	2.8	7	HBW10, Birds of Southeast Asia
Passeriformes	Muscicapidae	*Enicurus ruficapillus*	2.0	7	HBW10, Birds of Southeast Asia
Passeriformes	Muscicapidae	*Enicurus immaculatus*	3.0	7	HBW10, Birds of Southeast Asia
Passeriformes	Muscicapidae	*Enicurus schistaceus*	2.8	7	HBW10, Birds of Southeast Asia
Passeriformes	Muscicapidae	*Enicurus leschenaulti*	2.8	7	HBW10, Birds of Southeast Asia
Passeriformes	Muscicapidae	*Enicurus maculatus*	3.5	7	HBW10, Birds of Southeast Asia
Passeriformes	Muscicapidae	*Cochoa purpurea*	2.8	7	HBW10, Birds of Southeast Asia
Passeriformes	Muscicapidae	*Cochoa viridis*	2.8	7	HBW10, Birds of Southeast Asia
Passeriformes	Muscicapidae	*Cochoa azurea*	2.0	7	HBW10
Passeriformes	Muscicapidae	*Saxicola rubetra*	5.3	3,4,5,7	HBW10, BA, EMN
Passeriformes	Muscicapidae	*Saxicola insignis*	4.5	7	HBW10, BIS
Passeriformes	Muscicapidae	*Saxicola torquata*	4.9	3,4,5,6,7	HBW10, BA, EMN
Passeriformes	Muscicapidae	*Saxicola leucura*	3.9	7	HBW10, Birds of Southeast Asia
Passeriformes	Muscicapidae	*Saxicola caprata*	3.9	4,7,8	HBW10, EMN, Birds of Southeast Asia, BPNG
Passeriformes	Muscicapidae	*Saxicola jerdoni*	3.5	7	HBW10, Birds of Southeast Asia
Passeriformes	Muscicapidae	*Saxicola ferrea*	4.9	7	HBW10, Birds of Southeast Asia
Passeriformes	Muscicapidae	*Oenanthe leucopyga*	3.9	3,4,5	HBW10, BA, EMN
Passeriformes	Muscicapidae	*Oenanthe monacha*	3.0	3,4,5	HBW10, BA, EMN
Passeriformes	Muscicapidae	*Oenanthe alboniger*	4.5	4,7	HBW10, EMN
Passeriformes	Muscicapidae	*Oenanthe leucura*	3.9	3,4,5	HBW10, BA, EMN
Passeriformes	Muscicapidae	*Oenanthe monticola*	2.8	5	HBW10, BA
Passeriformes	Muscicapidae	*Oenanthe phillipsi*	3.5	5	HBW10, BA
Passeriformes	Muscicapidae	*Oenanthe oenanthe*	5.3	2,3,4,5,7	HBW10, BA, EMN
Passeriformes	Muscicapidae	*Oenanthe lugens*	4.5	3,4,5	HBW10, BA, EMN
Passeriformes	Muscicapidae	*Oenanthe lugentoides*	4.5	4	HBW10, EMN
Passeriformes	Muscicapidae	*Oenanthe finschii*	5.0	3,4,5	HBW10, BA, EMN
Passeriformes	Muscicapidae	*Oenanthe moesta*	4.5	3,4	HBW10, BA, EMN
Passeriformes	Muscicapidae	*Oenanthe hispanica*	4.5	3,4,5	HBW10, BA, EMN
Passeriformes	Muscicapidae	*Oenanthe pleschanka*	4.9	3,4,5,7	HBW10, BA, EMN
Passeriformes	Muscicapidae	*Oenanthe cypriaca*	4.9	4	HBW10, BA
Passeriformes	Muscicapidae	*Oenanthe xanthoprymna*	4.9	3,4,5	HBW10, BA, EMN
Passeriformes	Muscicapidae	*Oenanthe deserti*	4.5	3,4,5,7	HBW10, BA, EMN
Passeriformes	Muscicapidae	*Oenanthe pileata*	3.5	5	HBW10, BA
Passeriformes	Muscicapidae	*Oenanthe isabellina*	5.5	3,4,5,7	HBW10, BA, EMN
Passeriformes	Muscicapidae	*Oenanthe bottae*	3.0	4,5	HBW10, BA
Passeriformes	Muscicapidae	*Cercomela sinuata*	2.4	5	HBW10, BA
Passeriformes	Muscicapidae	*Cercomela schlegelii*	2.8	5	HBW10, BA
Passeriformes	Muscicapidae	*Cercomela tractrac*	2.4	5	HBW10, BA
Passeriformes	Muscicapidae	*Cercomela familiaris*	3.0	4,5	HBW10, BA
Passeriformes	Muscicapidae	*Cercomela melanura*	3.5	3,4,5	HBW10, BA, EMN
Passeriformes	Muscicapidae	*Cercomela sordida*	3.0	5	HBW10, BA
Passeriformes	Muscicapidae	*Myrmecocichla tholloni*	2.0	5	HBW10, BA
Passeriformes	Muscicapidae	*Myrmecocichla formicivora*	3.5	5	HBW10, BA
Passeriformes	Muscicapidae	*Myrmecocichla nigra*	3.2	5	HBW10, BA
Passeriformes	Muscicapidae	*Myrmecocichla albifrons*	2.4	5	HBW10, BA
Passeriformes	Muscicapidae	*Myrmecocichla arnotti*	2.8	5	HBW10, BA
Passeriformes	Muscicapidae	*Thamnolaea cinnamomeiventri*	2.8	5	HBW10, BA
Passeriformes	Muscicapidae	*Thamnolaea semirufa*	3.0	5	HBW10, BA
Passeriformes	Muscicapidae	*Pinarornis plumosus*	2.4	5	HBW10, BA
Passeriformes	Sturnidae	*Aplonis tabuensis*	2.4	9,11	Starlings & Mynas
Passeriformes	Sturnidae	*Aplonis cantoroides*	2.4	8,9	Starlings & Mynas
Passeriformes	Sturnidae	*Aplonis panayensis*	3.5	7	Starlings & Mynas

Order	Family	*Species*	Clutch	Range	Traits
Passeriformes	Sturnidae	*Aplonis metallica*	1.7	7,8,9,10	Starlings & Mynas
Passeriformes	Sturnidae	*Onychognathus walleri*	1.7	5	Starlings & Mynas
Passeriformes	Sturnidae	*Onychognathus nabouroup*	3.2	5	Starlings & Mynas
Passeriformes	Sturnidae	*Onychognathus tristramii*	2.8	3,4	Starlings & Mynas
Passeriformes	Sturnidae	*Onychognathus morio*	3.0	5	Starlings & Mynas
Passeriformes	Sturnidae	*Onychognathus blythii*	4.5	4,5	Starlings & Mynas
Passeriformes	Sturnidae	*Onychognathus tenuirostris*	2.8	5	Starlings & Mynas
Passeriformes	Sturnidae	*Onychognathus albirostris*	2.4	5	Starlings & Mynas
Passeriformes	Sturnidae	*Lamprotornis cupreocauda*	3.0	5	Starlings & Mynas
Passeriformes	Sturnidae	*Lamprotornis purpureiceps*	3.0	5	Starlings & Mynas
Passeriformes	Sturnidae	*Lamprotornis corruscus*	2.8	5	Starlings & Mynas
Passeriformes	Sturnidae	*Lamprotornis purpureus*	3.0	5	Starlings & Mynas
Passeriformes	Sturnidae	*Lamprotornis nitens*	2.4	5	Starlings & Mynas
Passeriformes	Sturnidae	*Lamprotornis chalcurus*	4.0	5	Starlings & Mynas
Passeriformes	Sturnidae	*Lamprotornis chalybaeus*	3.9	5	Starlings & Mynas
Passeriformes	Sturnidae	*Lamprotornis chloropterus*	3.0	5	Starlings & Mynas
Passeriformes	Sturnidae	*Lamprotornis acuticaudus*	3.0	5	Starlings & Mynas
Passeriformes	Sturnidae	*Lamprotornis splendidus*	2.0	5	Starlings & Mynas
Passeriformes	Sturnidae	*Lamprotornis australis*	2.8	5	Starlings & Mynas
Passeriformes	Sturnidae	*Lamprotornis mevesii*	3.5	5	Starlings & Mynas
Passeriformes	Sturnidae	*Lamprotornis caudatus*	3.5	5	Starlings & Mynas
Passeriformes	Sturnidae	*Lamprotornis purpuropterus*	3.0	5	Starlings & Mynas
Passeriformes	Sturnidae	*Lamprotornis superbus*	4.0	5	Starlings & Mynas
Passeriformes	Sturnidae	*Lamprotornis pulcher*	3.9	5	Starlings & Mynas
Passeriformes	Sturnidae	*Lamprotornis shelleyi*	4.2	5	Starlings & Mynas
Passeriformes	Sturnidae	*Lamprotornis hildebrandti*	3.5	5	Starlings & Mynas
Passeriformes	Sturnidae	*Cinnyricinclus sharpii*	3.0	5	Starlings & Mynas
Passeriformes	Sturnidae	*Cinnyricinclus leucogaster*	2.8	4,5	Starlings & Mynas
Passeriformes	Sturnidae	*Spreo fischeri*	4.2	5	Starlings & Mynas
Passeriformes	Sturnidae	*Spreo bicolor*	3.5	5	Starlings & Mynas
Passeriformes	Sturnidae	*Spreo albicapillus*	5.5	5	Starlings & Mynas
Passeriformes	Sturnidae	*Cosmopsarus regius*	3.5	5	Starlings & Mynas
Passeriformes	Sturnidae	*Cosmopsarus unicolor*	3.0	5	Starlings & Mynas
Passeriformes	Sturnidae	*Creatophora cinerea*	3.2	4,5	Starlings & Mynas
Passeriformes	Sturnidae	*Sturnus senex*	2.0	7	Starlings & Mynas
Passeriformes	Sturnidae	*Sturnus malabaricus*	3.9	7	Starlings & Mynas
Passeriformes	Sturnidae	*Sturnus erythropygius*	4.0	7	Starlings & Mynas
Passeriformes	Sturnidae	*Sturnus pagodarum*	3.9	7	Starlings & Mynas, Birds of Southeast Asia
Passeriformes	Sturnidae	*Sturnus sturninus*	4.6	4,7	Starlings & Mynas, Birds of Southeast Asia
Passeriformes	Sturnidae	*Sturnus philippensis*	4.6	7	Starlings & Mynas
Passeriformes	Sturnidae	*Sturnus sinensis*	4.0	7	Starlings & Mynas
Passeriformes	Sturnidae	*Sturnus roseus*	4.2	3,4,5,7	Starlings & Mynas
Passeriformes	Sturnidae	*Sturnus vulgaris*	5.9	5,7,8,10,11,12,13	Starlings & Mynas, Birds of the West Indies
Passeriformes	Sturnidae	*Sturnus unicolor*	3.2	3,4,5	Starlings & Mynas
Passeriformes	Sturnidae	*Sturnus cineraceus*	3.2	7	Starlings & Mynas
Passeriformes	Sturnidae	*Sturnus contra*	4.5	7	Starlings & Mynas, Birds of Southeast Asia
Passeriformes	Sturnidae	*Sturnus nigricollis*	3.9	7	Starlings & Mynas, Birds of Southeast Asia
Passeriformes	Sturnidae	*Sturnus melanopterus*	3.5	7	Starlings & Mynas, Birds of Southeast Asia
Passeriformes	Sturnidae	*Acridotheres tristis*	3.5	5,7,9,10,11,12,13	Starlings & Mynas
Passeriformes	Sturnidae	*Acridotheres ginginianus*	4.9	4,7	Starlings & Mynas
Passeriformes	Sturnidae	*Acridotheres fuscus*	4.2	7,11	Starlings & Mynas
Passeriformes	Sturnidae	*Acridotheres grandis*	4.9	7	Starlings & Mynas, Birds of Southeast Asia
Passeriformes	Sturnidae	*Acridotheres albocinctus*	4.0	7	Starlings & Mynas, Birds of Southeast Asia
Passeriformes	Sturnidae	*Acridotheres cristatellus*	5.3	2,7	Starlings & Mynas
Passeriformes	Sturnidae	*Mino anais*	2.0	8	Starlings & Mynas
Passeriformes	Sturnidae	*Mino dumontii*	1.4	7,8,9	Starlings & Mynas
Passeriformes	Sturnidae	*Gracula ptilogenys*	2.0	7	Starlings & Mynas
Passeriformes	Sturnidae	*Gracula religiosa*	1.7	7	Starlings & Mynas
Passeriformes	Sturnidae	*Buphagus africanus*	2.4	5	Starlings & Mynas
Passeriformes	Sturnidae	*Buphagus erythrorhynchus*	3.2	5	Starlings & Mynas
Passeriformes	Sturnidae	*Dumetella carolinensis*	3.5	2,13	HBW10, BMx
Passeriformes	Sturnidae	*Melanoptila glabrirostris*	2.0	2	HBW10, BMx
Passeriformes	Sturnidae	*Melanotis caerulescens*	2.0	2	HBW10, Wrens, dippers, thrashers

Order	Family	Species	Clutch	Range	Traits
Passeriformes	Sturnidae	*Melanotis hypoleucus*	2.4	2	HBW10, Wrens, dippers, thrashers
Passeriformes	Sturnidae	*Mimus polyglottos*	3.5	2,11	HBW10, Birds of the West Indies
Passeriformes	Sturnidae	*Mimus gilvus*	2.8	2	HBW10, BSA
Passeriformes	Sturnidae	*Mimus gundlachii*	2.0	2	HBW10, Birds of the West Indies
Passeriformes	Sturnidae	*Mimus saturninus*	3.5	2	HBW10, BSA
Passeriformes	Sturnidae	*Mimus triurus*	4.0	2	HBW10, BSA
Passeriformes	Sturnidae	*Mimus thenca*	3.5	2	HBW10, BSA
Passeriformes	Sturnidae	*Nesomimus parvulus*	3.5	2	HBW10, EBW
Passeriformes	Sturnidae	*Oreoscoptes montanus*	4.5	2	HBW10, Wrens, dippers, thrashers
Passeriformes	Sturnidae	*Toxostoma rufum*	3.9	2	HBW10, Birds of the West Indies
Passeriformes	Sturnidae	*Toxostoma longirostre*	3.9	2	HBW10, Wrens, dippers, thrashers
Passeriformes	Sturnidae	*Toxostoma bendirei*	3.5	2	HBW10, Wrens, dippers, thrashers
Passeriformes	Sturnidae	*Toxostoma cinereum*	2.4	2	HBW10, Wrens, dippers, thrashers
Passeriformes	Sturnidae	*Toxostoma curvirostre*	3.9	2	HBW10, Wrens, dippers, thrashers
Passeriformes	Sturnidae	*Toxostoma ocellatum*	2.0	2	HBW10, Wrens, dippers, thrashers
Passeriformes	Sturnidae	*Toxostoma lecontei*	3.2	2	HBW10, Wrens, dippers, thrashers
Passeriformes	Sturnidae	*Toxostoma redivivum*	3.5	2	HBW10, Wrens, dippers, thrashers
Passeriformes	Sturnidae	*Toxostoma crissale*	2.4	2	HBW10, Wrens, dippers, thrashers
Passeriformes	Sturnidae	*Margarops fuscatus*	2.4	2	HBW10, BSA
Passeriformes	Sittidae	*Sitta europaea*	7.3	3,4,5,7	Chickades, tits, nuthatches & Treecreepers
Passeriformes	Sittidae	*Sitta nagaensis*	3.2	7	Chickades, tits, nuthatches & Treecreepers
Passeriformes	Sittidae	*Sitta cashmirensis*	5.9	7	Chickades, tits, nuthatches & Treecreepers
Passeriformes	Sittidae	*Sitta castanea*	4.9	7	Chickades, tits, nuthatches & Treecreepers
Passeriformes	Sittidae	*Sitta himalayensis*	5.3	7	Chickades, tits, nuthatches & Treecreepers
Passeriformes	Sittidae	*Sitta victoriae*	4.2	7	Chickades, tits, nuthatches & Treecreepers; Birds of Southeast Asia
Passeriformes	Sittidae	*Sitta pygmaea*	7.3	2	Chickades, tits, nuthatches & Treecreepers
Passeriformes	Sittidae	*Sitta pusilla*	4.9	2	Chickades, tits, nuthatches & Treecreepers
Passeriformes	Sittidae	*Sitta whiteheadi*	5.5	3	Chickades, tits, nuthatches & Treecreepers
Passeriformes	Sittidae	*Sitta ledanti*	2.8	3,5	Chickades, tits, nuthatches & Treecreepers
Passeriformes	Sittidae	*Sitta krueperi*	5.9	3,4	Chickades, tits, nuthatches & Treecreepers
Passeriformes	Sittidae	*Sitta villosa*	5.5	7	Chickades, tits, nuthatches & Treecreepers
Passeriformes	Sittidae	*Sitta canadensis*	5.5	2	Chickades, tits, nuthatches & Treecreepers
Passeriformes	Sittidae	*Sitta leucopsis*	6.9	7	Chickades, tits, nuthatches & Treecreepers
Passeriformes	Sittidae	*Sitta carolinensis*	6.7	2	Chickades, tits, nuthatches & Treecreepers
Passeriformes	Sittidae	*Sitta neumayer*	6.3	3,4	Chickades, tits, nuthatches & Treecreepers
Passeriformes	Sittidae	*Sitta tephronota*	5.9	3,4,7	Chickades, tits, nuthatches & Treecreepers
Passeriformes	Sittidae	*Sitta frontalis*	4.5	7	Chickades, tits, nuthatches & Treecreepers
Passeriformes	Sittidae	*Sitta azurea*	3.5	7	Chickades, tits, nuthatches & Treecreepers
Passeriformes	Sittidae	*Sitta magna*	3.0	7	Chickades, tits, nuthatches & Treecreepers; Birds of Southeast Asia
Passeriformes	Sittidae	*Sitta formosa*	4.9	7	Chickades, tits, nuthatches & Treecreepers; Birds of Southeast Asia
Passeriformes	Sittidae	*Tichodroma muraria*	4.5	3,4,7	Chickades, tits, nuthatches & Treecreepers; EMN
Passeriformes	Certhiidae	*Certhia familiaris*	4.2	3,4,7	Chickades, tits, nuthatches & Treecreepers
Passeriformes	Certhiidae	*Certhia americana*	5.5	2	Chickades, tits, nuthatches & Treecreepers
Passeriformes	Certhiidae	*Certhia brachydactyla*	5.9	3,4,5	Chickades, tits, nuthatches & Treecreepers
Passeriformes	Certhiidae	*Certhia himalayana*	4.9	7	Chickades, tits, nuthatches & Treecreepers
Passeriformes	Certhiidae	*Certhia discolor*	3.9	7	Chickades, tits, nuthatches & Treecreepers
Passeriformes	Certhiidae	*Donacobius atricapillus*	2.4	2	HBW10, BSA
Passeriformes	Certhiidae	*Campylorhynchus gularis*	4.0	2	HBW10
Passeriformes	Certhiidae	*Campylorhynchus brunneicapil*	3.9	2	HBW10
Passeriformes	Certhiidae	*Campylorhynchus jocosus*	3.5	2	HBW10
Passeriformes	Certhiidae	*Campylorhynchus chiapensis*	3.0	2	HBW10
Passeriformes	Certhiidae	*Campylorhynchus griseus*	3.9	2	HBW10, Birds of Venezuela; BSA
Passeriformes	Certhiidae	*Campylorhynchus rufinucha*	4.5	2	HBW10, Birds of Costa Rica
Passeriformes	Certhiidae	*Campylorhynchus zonatus*	3.9	2	HBW10, BSA, Birds of Costa Rica
Passeriformes	Certhiidae	*Campylorhynchus nuchalis*	4.0	2	HBW10, Birds of Venezuela; BSA
Passeriformes	Certhiidae	*Campylorhynchus fasciatus*	3.5	2	HBW10, BSA
Passeriformes	Certhiidae	*Salpinctes obsoletus*	4.9	2	HBW10, Birds of Costa Rica
Passeriformes	Certhiidae	*Catherpes mexicanus*	6.0	2	HBW10, BNA
Passeriformes	Certhiidae	*Hylorchilus sumichrasti*	3.0	2	HBW10
Passeriformes	Certhiidae	*Cistothorus platensis*	7.0	2	HBW10, BSA, Birds of Costa Rica
Passeriformes	Certhiidae	*Cistothorus palustris*	4.9	2	HBW10, BNA
Passeriformes	Certhiidae	*Thryomanes bewickii*	5.5	2	HBW10, Wrens, dippers, thrashers
Passeriformes	Certhiidae	*Ferminia cerverai*	2.0	2	HBW10, Birds of the West Indies

Order	Family	*Species*	Clutch	Range	Traits
Passeriformes	Certhiidae	*Thryothorus spadix*	2.0	2	HBW10, BSA
Passeriformes	Certhiidae	*Thryothorus coraya*	2.0	2	HBW10, BSA
Passeriformes	Certhiidae	*Thryothorus felix*	5.0	2	HBW10, Wrens, dippers, thrashers
Passeriformes	Certhiidae	*Thryothorus maculipectus*	3.5	2	HBW10, Birds of Costa Rica
Passeriformes	Certhiidae	*Thryothorus rutilus*	2.8	2	HBW10, BSA, Birds of Costa Rica
Passeriformes	Certhiidae	*Thryothorus semibadius*	2.0	2	HBW10, Birds of Costa Rica
Passeriformes	Certhiidae	*Thryothorus nigricapillus*	3.0	2	HBW10, BSA, Birds of Costa Rica
Passeriformes	Certhiidae	*Thryothorus thoracicus*	2.4	2	HBW10, Birds of Costa Rica
Passeriformes	Certhiidae	*Thryothorus pleurostictus*	3.2	2	HBW10, Birds of Costa Rica
Passeriformes	Certhiidae	*Thryothorus ludovicianus*	4.2	2	HBW10
Passeriformes	Certhiidae	*Thryothorus rufalbus*	2.8	2	HBW10, Birds of Venezuela; BSA, Birds of Costa Rica
Passeriformes	Certhiidae	*Thryothorus sinaloa*	5.0	2	HBW10, Wrens, dippers, thrashers
Passeriformes	Certhiidae	*Thryothorus modestus*	2.4	2	HBW10, Birds of Costa Rica
Passeriformes	Certhiidae	*Thryothorus leucotis*	2.4	2	HBW10, Birds of Venezuela; BSA
Passeriformes	Certhiidae	*Thryothorus superciliaris*	2.4	2	HBW10, BSA
Passeriformes	Certhiidae	*Troglodytes troglodytes*	6.3	2,3,4,7	HBW10, BA, EMN
Passeriformes	Certhiidae	*Troglodytes aedon*	5.7	2	HBW10, BSA, Birds of Costa Rica
Passeriformes	Certhiidae	*Troglodytes cobbi*	3.5	2	HBW10
Passeriformes	Certhiidae	*Troglodytes rufociliatus*	3.0	2	HBW10
Passeriformes	Certhiidae	*Troglodytes ochraceus*	2.0	2	HBW10, Birds of Costa Rica
Passeriformes	Certhiidae	*Troglodytes solstitialis*	2.0	2	HBW10, BSA
Passeriformes	Certhiidae	*Thryorchilus browni*	2.0	2	HBW10, Birds of Costa Rica
Passeriformes	Certhiidae	*Uropsila leucogastra*	4.0	2	HBW10
Passeriformes	Certhiidae	*Henicorhina leucosticta*	2.0	2	HBW10, BSA, Birds of Costa Rica
Passeriformes	Certhiidae	*Henicorhina leucophrys*	2.0	2	HBW10, BSA, Birds of Costa Rica
Passeriformes	Certhiidae	*Microcerculus marginatus*	2.4	2	HBW10, BSA
Passeriformes	Certhiidae	*Microcerculus bambla*	1.0	2	HBW10, BSA
Passeriformes	Certhiidae	*Cyphorhinus phaeocephalus*	2.4	2	HBW10, BSA, Birds of Costa Rica
Passeriformes	Certhiidae	*Cyphorhinus thoracicus*	2.0	2	HBW10, BSA
Passeriformes	Certhiidae	*Cyphorhinus aradus*	2.0	2	HBW10, BSA
Passeriformes	Certhiidae	*Auriparus flaviceps*	3.5	2	Chickades, tits, nuthatches & Treecreepers
Passeriformes	Certhiidae	*Microbates cinereiventris*	2.0	2	BSA, Birds of Costa Rica
Passeriformes	Certhiidae	*Ramphocaenus melanurus*	2.0	2	Birds of Venezuela; BSA, Birds of Costa Rica
Passeriformes	Certhiidae	*Polioptila caerulea*	3.0	2	Birds of the West Indies
Passeriformes	Certhiidae	*Polioptila californica*	3.9	2	BMx
Passeriformes	Certhiidae	*Polioptila melanura*	3.9	2	BMx
Passeriformes	Certhiidae	*Polioptila lembeyei*	3.9	2	Birds of the West Indies
Passeriformes	Certhiidae	*Polioptila albiloris*	2.4	2	Birds of Costa Rica
Passeriformes	Certhiidae	*Polioptila plumbea*	2.4	2	Birds of Venezuela; BSA, Birds of Costa Rica
Passeriformes	Certhiidae	*Polioptila maranonica*	2.4	2	Birds of Venezuela; BSA, Birds of Costa Rica
Passeriformes	Certhiidae	*Polioptila dumicola*	3.9	2	BMx
Passeriformes	Paridae	*Remiz pendulinus*	3.7	3,4,7	Chickades, tits, nuthatches & Treecreepers
Passeriformes	Paridae	*Remiz coronatus*	5.2	4,7	Chickades, tits, nuthatches & Treecreepers
Passeriformes	Paridae	*Remiz consobrinus*	7.1	7	Chickades, tits, nuthatches & Treecreepers
Passeriformes	Paridae	*Anthoscopus parvulus*	2.0	5	Chickades, tits, nuthatches & Treecreepers
Passeriformes	Paridae	*Anthoscopus musculus*	4.0	5	Chickades, tits, nuthatches & Treecreepers
Passeriformes	Paridae	*Anthoscopus caroli*	4.9	5	Chickades, tits, nuthatches & Treecreepers
Passeriformes	Paridae	*Anthoscopus minutus*	4.2	5	Chickades, tits, nuthatches & Treecreepers
Passeriformes	Paridae	*Parus palustris*	7.1	3,4,7	Chickades, tits, nuthatches & Treecreepers; EMN
Passeriformes	Paridae	*Parus lugubris*	5.9	3,4	Chickades, tits, nuthatches & Treecreepers; EMN
Passeriformes	Paridae	*Parus montanus*	7.3	3,7	Chickades, tits, nuthatches & Treecreepers
Passeriformes	Paridae	*Parus carolinensis*	5.5	2	Chickades, tits, nuthatches & Treecreepers
Passeriformes	Paridae	*Parus atricapillus*	6.9	2	Chickades, tits, nuthatches & Treecreepers
Passeriformes	Paridae	*Parus gambeli*	6.7	2	Chickades, tits, nuthatches & Treecreepers
Passeriformes	Paridae	*Parus sclateri*	6.7	2	Chickades, tits, nuthatches & Treecreepers
Passeriformes	Paridae	*Parus cinctus*	7.7	2,3,7	Chickades, tits, nuthatches & Treecreepers
Passeriformes	Paridae	*Parus hudsonicus*	6.5	2	Chickades, tits, nuthatches & Treecreepers
Passeriformes	Paridae	*Parus rufescens*	6.9	2	Chickades, tits, nuthatches & Treecreepers
Passeriformes	Paridae	*Parus rufonuchalis*	4.9	7	Chickades, tits, nuthatches & Treecreepers
Passeriformes	Paridae	*Parus rubidiventris*	2.4	7	Chickades, tits, nuthatches & Treecreepers
Passeriformes	Paridae	*Parus melanolophus*	5.5	7	Chickades, tits, nuthatches & Treecreepers
Passeriformes	Paridae	*Parus ater*	9.4	3,4,5,7	Chickades, tits, nuthatches & Treecreepers
Passeriformes	Paridae	*Parus venustulus*	5.9	7	Chickades, tits, nuthatches & Treecreepers

Order	Family	*Species*	Clutch	Range	Traits
Passeriformes	Paridae	*Parus cristatus*	6.3	3,4	Chickades, tits, nuthatches & Treecreepers
Passeriformes	Paridae	*Parus dichrous*	4.5	7	Chickades, tits, nuthatches & Treecreepers
Passeriformes	Paridae	*Parus leucomelas*	3.9	5	Chickades, tits, nuthatches & Treecreepers
Passeriformes	Paridae	*Parus niger*	4.5	5	Chickades, tits, nuthatches & Treecreepers
Passeriformes	Paridae	*Parus albiventris*	3.9	5	Chickades, tits, nuthatches & Treecreepers
Passeriformes	Paridae	*Parus funereus*	3.0	5	Chickades, tits, nuthatches & Treecreepers
Passeriformes	Paridae	*Parus rufiventris*	3.2	5	Chickades, tits, nuthatches & Treecreepers
Passeriformes	Paridae	*Parus fringillinus*	3.0	5	Chickades, tits, nuthatches & Treecreepers
Passeriformes	Paridae	*Parus fasciiventer*	3.0	5	Chickades, tits, nuthatches & Treecreepers
Passeriformes	Paridae	*Parus griseiventris*	3.9	5	Chickades, tits, nuthatches & Treecreepers
Passeriformes	Paridae	*Parus cinerascens*	4.2	5	Chickades, tits, nuthatches & Treecreepers
Passeriformes	Paridae	*Parus afer*	3.5	5	Chickades, tits, nuthatches & Treecreepers
Passeriformes	Paridae	*Parus major*	7.7	3,4,5,7	Chickades, tits, nuthatches & Treecreepers
Passeriformes	Paridae	*Parus monticolus*	5.7	7	Chickades, tits, nuthatches & Treecreepers
Passeriformes	Paridae	*Parus xanthogenys*	4.5	7	Chickades, tits, nuthatches & Treecreepers
Passeriformes	Paridae	*Parus spilonotus*	4.9	7	Chickades, tits, nuthatches & Treecreepers
Passeriformes	Paridae	*Parus holsti*	3.5	7	Chickades, tits, nuthatches & Treecreepers
Passeriformes	Paridae	*Parus caeruleus*	5.1	3,4,5	Chickades, tits, nuthatches & Treecreepers
Passeriformes	Paridae	*Parus ultramarinus*	5.1	13	Chickades, tits, nuthatches & Treecreepers
Passeriformes	Paridae	*Parus teneriffae*	5.1	13	Chickades, tits, nuthatches & Treecreepers
Passeriformes	Paridae	*Parus cyanus*	8.8	3,4,7	Chickades, tits, nuthatches & Treecreepers
Passeriformes	Paridae	*Parus flavipectus*	7.3	7	Chickades, tits, nuthatches & Treecreepers
Passeriformes	Paridae	*Parus varius*	6.9	7	Chickades, tits, nuthatches & Treecreepers
Passeriformes	Paridae	*Parus wollweberi*	5.9	2	Chickades, tits, nuthatches & Treecreepers
Passeriformes	Paridae	*Parus inornatus*	6.9	2	Chickades, tits, nuthatches & Treecreepers
Passeriformes	Paridae	*Parus ridgwayi*	6.9	2	Chickades, tits, nuthatches & Treecreepers
Passeriformes	Paridae	*Parus bicolor*	5.9	2	Chickades, tits, nuthatches & Treecreepers
Passeriformes	Paridae	*Parus atricristatus*	6.0	2	Chickades, tits, nuthatches & Treecreepers
Passeriformes	Aegithalidae	*Aegithalos caudatus*	9.8	3,4,7	Chickades, tits, nuthatches & Treecreepers
Passeriformes	Aegithalidae	*Aegithalos leucogenys*	6.3	7	Chickades, tits, nuthatches & Treecreepers
Passeriformes	Aegithalidae	*Aegithalos concinnus*	4.2	7	Chickades, tits, nuthatches & Treecreepers
Passeriformes	Aegithalidae	*Aegithalos niveogularis*	4.0	7	Chickades, tits, nuthatches & Treecreepers
Passeriformes	Aegithalidae	*Aegithalos iouschistos*	4.5	7	Chickades, tits, nuthatches & Treecreepers; Birds of Southeast Asia
Passeriformes	Aegithalidae	*Psaltriparus minimus*	5.9	2	Chickades, tits, nuthatches & Treecreepers
Passeriformes	Aegithalidae	*Psaltria exilis*	2.4	7	Chickades, tits, nuthatches & Treecreepers
Passeriformes	Hirundinidae	*Pseudochelidon eurystomina*	3.0	5	HBW9, BA
Passeriformes	Hirundinidae	*Tachycineta bicolor*	5.5	2	HBW9, BSA, Birds of Costa Rica, Swallows & Martins
Passeriformes	Hirundinidae	*Tachycineta albilinea*	3.9	2	HBW9, BSA, Birds of Costa Rica, Swallows & Martins
Passeriformes	Hirundinidae	*Tachycineta albiventer*	4.2	2	HBW9, Birds of Venezuela; BSA, Swallows & Martins
Passeriformes	Hirundinidae	*Tachycineta leucorrhoa*	5.3	2	HBW9, BSA, Swallows & Martins
Passeriformes	Hirundinidae	*Tachycineta meyeni*	4.2	2	HBW9, BSA, Swallows & Martins
Passeriformes	Hirundinidae	*Tachycineta thalassina*	4.9	2	HBW9, Birds of Costa Rica, Swallows & Martins
Passeriformes	Hirundinidae	*Tachycineta cyaneoviridis*	3.0	2	HBW9, Birds of the West Indies, Swallows & Martins
Passeriformes	Hirundinidae	*Tachycineta euchrysea*	3.0	2	HBW9, Birds of the West Indies, Swallows & Martins
Passeriformes	Hirundinidae	*Progne subis*	4.9	2	HBW9, Birds of Venezuela; BSA, Birds of Costa Rica, Swallows & Mart
Passeriformes	Hirundinidae	*Progne cryptoleuca*	4.9	2	HBW9, Birds of the West Indies
Passeriformes	Hirundinidae	*Progne dominicensis*	4.9	2	HBW9, BSA, Birds of the West Indies, Swallows & Martins
Passeriformes	Hirundinidae	*Progne chalybea*	3.2	2	HBW9, Birds of Venezuela, Birds of Costa Rica, Swallows & Martins
Passeriformes	Hirundinidae	*Progne modesta*	3.9	2	HBW9, BSA, Swallows & Martins
Passeriformes	Hirundinidae	*Progne tapera*	4.0	2	HBW9, Birds of Venezuela; BSA, Birds of Costa Rica, Swallows & Mart
Passeriformes	Hirundinidae	*Notiochelidon murina*	2.4	2	HBW9, Birds of Venezuela; BSA, Swallows & Martins
Passeriformes	Hirundinidae	*Notiochelidon cyanoleuca*	2.8	2	HBW9, BSA, Birds of Costa Rica, Swallows & Martins
Passeriformes	Hirundinidae	*Notiochelidon pileata*	4.0	2	HBW9, Swallows & Martins
Passeriformes	Hirundinidae	*Atticora fasciata*	4.5	2	HBW9, Birds of Venezuela; BSA, Swallows & Martins
Passeriformes	Hirundinidae	*Atticora melanoleuca*	3.0	2	HBW9, Birds of Venezuela, Swallows & Martins
Passeriformes	Hirundinidae	*Stelgidopteryx fucata*	4.5	2	HBW9, Birds of Venezuela, Swallows & Martins
Passeriformes	Hirundinidae	*Stelgidopteryx serripennis*	5.7	2	HBW9, BSA, Birds of Costa Rica, Swallows & Martins
Passeriformes	Hirundinidae	*Stelgidopteryx ruficollis*	4.9	2	HBW9, Birds of Venezuela, Birds of Costa Rica, Swallows & Martins
Passeriformes	Hirundinidae	*Cheramoeca leucosternus*	4.9	10	HBW9, Compete Book of Australian Birds
Passeriformes	Hirundinidae	*Riparia riparia*	4.2	2,3,4,5,6,7,8	HBW9, BA, EMN
Passeriformes	Hirundinidae	*Riparia paludicola*	3.2	3,4,5,6,7	HBW9, BA, EMN, HBW9
Passeriformes	Hirundinidae	*Riparia cincta*	3.2	4,5	HBW9, BA
Passeriformes	Hirundinidae	*Phedina borbonica*	3.5	5,6,13	HBW9, BA,Swallows & Martins

Order	Family	*Species*	Clutch	Range	Traits
Passeriformes	Hirundinidae	*Phedina brazzae*	3.0	5	HBW9, BA,Swallows & Martins
Passeriformes	Hirundinidae	*Hirundo rupestris*	3.2	3,4,5,7	HBW9, BA, EMN, Swallows & Martins
Passeriformes	Hirundinidae	*Hirundo obsoleta*	2.4	4	HBW9, BA
Passeriformes	Hirundinidae	*Hirundo fuligula*	3.9	3,4,5,7	HBW9, BA, EMN
Passeriformes	Hirundinidae	*Hirundo concolor*	2.8	7	HBW9, Birds of Southeast Asia
Passeriformes	Hirundinidae	*Hirundo rustica*	4.5	3,4,5,6,7,8,10,11	HBW9, BA, EMN
Passeriformes	Hirundinidae	*Hirundo lucida*	3.5	5	HBW9, BA
Passeriformes	Hirundinidae	*Hirundo aethiopica*	2.8	4,5	HBW9, BA
Passeriformes	Hirundinidae	*Hirundo angolensis*	2.4	5	HBW9, BA
Passeriformes	Hirundinidae	*Hirundo albigularis*	3.0	5	HBW9, BA
Passeriformes	Hirundinidae	*Hirundo domicola*	3.5	7	HBW9, Swallows & Martins
Passeriformes	Hirundinidae	*Hirundo tahitica*	2.0	7,8,9,11	HBW9, Birds of Southeast Asia, HBW9
Passeriformes	Hirundinidae	*Hirundo neoxena*	3.5	8,10,12	HBW9, Compete Book of Australian Birds
Passeriformes	Hirundinidae	*Hirundo smithii*	3.2	4,5,7	HBW9, BA, Swallows & Martins
Passeriformes	Hirundinidae	*Hirundo nigrita*	2.4	5	HBW9, BA, Swallows & Martins
Passeriformes	Hirundinidae	*Hirundo nigrorufa*	3.0	5	HBW9, BA, Swallows & Martins
Passeriformes	Hirundinidae	*Hirundo atrocaerulea*	2.4	5	HBW9, BA, Swallows & Martins
Passeriformes	Hirundinidae	*Hirundo leucosoma*	4.0	5	HBW9, BA, Swallows & Martins
Passeriformes	Hirundinidae	*Hirundo dimidiata*	2.4	5	HBW9, BA, Swallows & Martins
Passeriformes	Hirundinidae	*Hirundo cucullata*	3.0	5	HBW9, BA, Swallows & Martins
Passeriformes	Hirundinidae	*Hirundo abyssinica*	3.0	4,5	HBW9, BA, Swallows & Martins
Passeriformes	Hirundinidae	*Hirundo semirufa*	3.0	5	HBW9, BA, Swallows & Martins
Passeriformes	Hirundinidae	*Hirundo senegalensis*	2.8	5	HBW9, BA, Swallows & Martins
Passeriformes	Hirundinidae	*Hirundo daurica*	3.7	3,4,5,7,8,10	HBW9, BA, EMN, Swallows & Martins
Passeriformes	Hirundinidae	*Hirundo striolata*	3.9	7,8	HBW9, Birds of Southeast Asia, Swallows & Martins, HBW9
Passeriformes	Hirundinidae	*Hirundo preussi*	2.4	5	HBW9, BA, Swallows & Martins
Passeriformes	Hirundinidae	*Hirundo rufigula*	2.0	5	HBW9, BA, Swallows & Martins
Passeriformes	Hirundinidae	*Hirundo spilodera*	3.0	5	HBW9, BA, Swallows & Martins
Passeriformes	Hirundinidae	*Hirundo pyrrhonota*	2.4	2	HBW9, Birds of Venezuela; BSA, Birds of Costa Rica, Swallows & Mart
Passeriformes	Hirundinidae	*Hirundo fulva*	2.4	2	HBW9, Birds of the West Indies, Swallows & Martins
Passeriformes	Hirundinidae	*Hirundo nigricans*	3.9	8,9,10,12	HBW9, Compete Book of Australian Birds, Swallows & Martins
Passeriformes	Hirundinidae	*Hirundo fluvicola*	3.5	7	HBW9, Swallows & Martins
Passeriformes	Hirundinidae	*Hirundo ariel*	4.5	8,10,12	HBW9, BPNG
Passeriformes	Hirundinidae	*Hirundo fuliginosa*	2.4	5	HBW9, BA,Swallows & Martins
Passeriformes	Hirundinidae	*Delichon urbica*	4.5	3,4,5,7	HBW9, BA, EMN
Passeriformes	Hirundinidae	*Delichon dasypus*	3.5	4,7	HBW9, Birds of Southeast Asia, Swallows & Martins
Passeriformes	Hirundinidae	*Delichon nipalensis*	3.9	7	HBW9, Birds of Southeast Asia, Swallows & Martins
Passeriformes	Hirundinidae	*Psalidoprocne nitens*	2.0	5	HBW9, BA, Swallows & Martins
Passeriformes	Hirundinidae	*Psalidoprocne fuliginosa*	2.0	5	HBW9, BA
Passeriformes	Hirundinidae	*Psalidoprocne albiceps*	2.8	5	HBW9, BA
Passeriformes	Hirundinidae	*Psalidoprocne pristoptera*	2.0	5	HBW9, BA
Passeriformes	Hirundinidae	*Psalidoprocne obscura*	2.0	5	HBW9, BA
Passeriformes	Regulidae	*Regulus calendula*	7.9	2	HBW11, EMN, Birds of the West Indies
Passeriformes	Regulidae	*Regulus regulus*	9.9	3,4,5,7	HBW11, Warblers of Europe, Asia and North Africa; EMN
Passeriformes	Regulidae	*Regulus ignicapillus*	6.9	3,4	HBW11, Warblers of Europe, Asia and North Africa
Passeriformes	Regulidae	*Regulus satrapa*	8.5	2	HBW11, EMN
Passeriformes	Pycnonotidae	*Spizixos canifrons*	2.4	7	HBW10, BIP
Passeriformes	Pycnonotidae	*Spizixos semitorques*	3.5	7	HBW10, Birds of Southeast Asia
Passeriformes	Pycnonotidae	*Pycnonotus zeylanicus*	2.0	7	HBW10, Birds of Southeast Asia
Passeriformes	Pycnonotidae	*Pycnonotus striatus*	3.0	7	HBW10, BIP
Passeriformes	Pycnonotidae	*Pycnonotus priocephalus*	1.0	7	HBW10, BIP
Passeriformes	Pycnonotidae	*Pycnonotus atriceps*	2.4	7	HBW10, BIP
Passeriformes	Pycnonotidae	*Pycnonotus melanicterus*	2.8	7	HBW10, BIP
Passeriformes	Pycnonotidae	*Pycnonotus squamatus*	2.0	7	HBW10, Birds of Southeast Asia
Passeriformes	Pycnonotidae	*Pycnonotus cyaniventris*	2.4	7	HBW10, Birds of Southeast Asia
Passeriformes	Pycnonotidae	*Pycnonotus jocosus*	3.0	4,7,10,11,13	HBW10, BIP
Passeriformes	Pycnonotidae	*Pycnonotus xanthorrhous*	2.4	7	HBW10, Birds of Southeast Asia
Passeriformes	Pycnonotidae	*Pycnonotus sinensis*	3.5	7	HBW10, Birds of Southeast Asia
Passeriformes	Pycnonotidae	*Pycnonotus barbatus*	2.4	3,4,5	HBW10, BA, EMN
Passeriformes	Pycnonotidae	*Pycnonotus nigricans*	3.0	5	HBW10, BA
Passeriformes	Pycnonotidae	*Pycnonotus capensis*	2.4	5	HBW10, BA
Passeriformes	Pycnonotidae	*Pycnonotus xanthopygos*	3.0	3,4	HBW10, BA, EMN
Passeriformes	Pycnonotidae	*Pycnonotus leucogenys*	3.0	3,4,7	HBW10, EMN

Order	Family	Species	Clutch	Range	Traits
Passeriformes	Pycnonotidae	*Pycnonotus cafer*	2.4	4,7,11,12,13	HBW10, BIP
Passeriformes	Pycnonotidae	*Pycnonotus aurigaster*	3.5	7,8	HBW10, Birds of Southeast Asia
Passeriformes	Pycnonotidae	*Pycnonotus eutilotus*	2.0	7	HBW10, Birds of Southeast Asia
Passeriformes	Pycnonotidae	*Pycnonotus urostictus*	3.0	7	HBW10, Birds of Phillippines
Passeriformes	Pycnonotidae	*Pycnonotus finlaysoni*	2.8	7	HBW10, Birds of Southeast Asia
Passeriformes	Pycnonotidae	*Pycnonotus xantholaemus*	2.4	7	HBW10, BSI
Passeriformes	Pycnonotidae	*Pycnonotus penicillatus*	2.0	7	HBW10, BIP
Passeriformes	Pycnonotidae	*Pycnonotus flavescens*	2.4	7	HBW10, BIP
Passeriformes	Pycnonotidae	*Pycnonotus luteolus*	2.0	7	HBW10, BIP
Passeriformes	Pycnonotidae	*Pycnonotus goiavier*	2.8	7	HBW10, Birds of Southeast Asia, Birds of Phillippines
Passeriformes	Pycnonotidae	*Pycnonotus plumosus*	2.0	7	HBW10, Birds of Southeast Asia
Passeriformes	Pycnonotidae	*Pycnonotus blanfordi*	2.4	7	HBW10, Birds of Southeast Asia
Passeriformes	Pycnonotidae	*Pycnonotus simplex*	2.0	7	HBW10, Birds of Southeast Asia
Passeriformes	Pycnonotidae	*Pycnonotus brunneus*	2.0	7	HBW10, Birds of Southeast Asia
Passeriformes	Pycnonotidae	*Pycnonotus erythropthalmos*	2.0	7	HBW10, Birds of Southeast Asia
Passeriformes	Pycnonotidae	*Andropadus montanus*	1.4	5	HBW10, BA
Passeriformes	Pycnonotidae	*Andropadus masukuensis*	1.4	5	HBW10, BA
Passeriformes	Pycnonotidae	*Andropadus virens*	2.0	5	HBW10, BA
Passeriformes	Pycnonotidae	*Andropadus gracilis*	2.0	5	HBW10, BA
Passeriformes	Pycnonotidae	*Andropadus importunus*	1.7	5	HBW10, BA
Passeriformes	Pycnonotidae	*Andropadus latirostris*	2.0	5	HBW10, BA
Passeriformes	Pycnonotidae	*Andropadus tephrolaemus*	2.0	5	HBW10, BA
Passeriformes	Pycnonotidae	*Andropadus milanjensis*	2.0	5	HBW10, BA
Passeriformes	Pycnonotidae	*Baeopogon indicator*	2.0	5	HBW10, BA
Passeriformes	Pycnonotidae	*Ixonotus guttatus*	2.0	5	HBW10, BA
Passeriformes	Pycnonotidae	*Chlorocichla simplex*	2.0	5	HBW10, BA
Passeriformes	Pycnonotidae	*Chlorocichla flavicollis*	2.0	5	HBW10, BA
Passeriformes	Pycnonotidae	*Chlorocichla falkensteini*	2.0	5	HBW10, BA
Passeriformes	Pycnonotidae	*Chlorocichla flaviventris*	2.0	5	HBW10, BA
Passeriformes	Pycnonotidae	*Pyrrhurus scandens*	2.4	5	HBW10, BA
Passeriformes	Pycnonotidae	*Phyllastrephus cabanisi*	2.0	5	HBW10, BA
Passeriformes	Pycnonotidae	*Phyllastrephus fischeri*	2.0	5	HBW10, Guide to the Birds of Madagascar
Passeriformes	Pycnonotidae	*Phyllastrephus terrestris*	2.0	5	HBW10, BA
Passeriformes	Pycnonotidae	*Phyllastrephus fulviventris*	2.0	5	HBW10, Guide to the Birds of Madagascar
Passeriformes	Pycnonotidae	*Phyllastrephus cerviniventris*	2.0	5	HBW10, BA
Passeriformes	Pycnonotidae	*Phyllastrephus baumanni*	2.0	5	HBW10, Guide to the Birds of Madagascar
Passeriformes	Pycnonotidae	*Phyllastrephus poensis*	2.0	5	HBW10, BA
Passeriformes	Pycnonotidae	*Phyllastrephus flavostriatus*	2.0	5	HBW10, BA
Passeriformes	Pycnonotidae	*Phyllastrephus debilis*	2.0	5	HBW10, BA
Passeriformes	Pycnonotidae	*Phyllastrephus albigularis*	2.0	5	HBW10, BA
Passeriformes	Pycnonotidae	*Phyllastrephus icterinus*	2.0	5	HBW10, BA
Passeriformes	Pycnonotidae	*Phyllastrephus xavieri*	2.0	5	HBW10, BA
Passeriformes	Pycnonotidae	*Phyllastrephus zosterops*	3.5	6	HBW10, Guide to the Birds of Madagascar
Passeriformes	Pycnonotidae	*Phyllastrephus cinereiceps*	3.0	6	HBW10, Guide to the Birds of Madagascar
Passeriformes	Pycnonotidae	*Bleda syndactyla*	2.0	5	HBW10, BA
Passeriformes	Pycnonotidae	*Bleda eximia*	2.0	5	HBW10, BA
Passeriformes	Pycnonotidae	*Bleda canicapilla*	2.0	5	HBW10, BA
Passeriformes	Pycnonotidae	*Nicator chloris*	1.0	5	HBW10, BA
Passeriformes	Pycnonotidae	*Nicator gularis*	2.4	5	HBW10, BA
Passeriformes	Pycnonotidae	*Nicator vireo*	2.0	5	HBW10, BA
Passeriformes	Pycnonotidae	*Criniger barbatus*	2.0	5	HBW10, BA
Passeriformes	Pycnonotidae	*Criniger chloronotus*	2.0	5	HBW10, BA
Passeriformes	Pycnonotidae	*Criniger calurus*	2.0	5	HBW10, BA
Passeriformes	Pycnonotidae	*Alophoixus flaveolus*	3.5	7	HBW10, BIP
Passeriformes	Pycnonotidae	*Alophoixus ochraceus*	2.0	7	HBW10, Birds of Southeast Asia
Passeriformes	Pycnonotidae	*Alophoixus bres*	2.0	7	HBW10, Birds of Southeast Asia, Birds of Phillippines
Passeriformes	Pycnonotidae	*Alophoixus phaeocephalus*	2.0	7	HBW10, Birds of Southeast Asia
Passeriformes	Pycnonotidae	*Iole virescens*	2.4	7	HBW10, Birds of Southeast Asia
Passeriformes	Pycnonotidae	*Iole olivacea*	2.0	7	HBW10, Birds of Southeast Asia
Passeriformes	Pycnonotidae	*Iole indica*	2.4	7	HBW10, BIP
Passeriformes	Pycnonotidae	*Ixos philippinus*	2.4	7	HBW10, Birds of Phillippines
Passeriformes	Pycnonotidae	*Ixos amaurotis*	3.9	7	HBW10, BIP
Passeriformes	Pycnonotidae	*Ixos malaccensis*	2.0	7	HBW10, Birds of Southeast Asia

Order	Family	*Species*	Clutch	Range	Traits
Passeriformes	Pycnonotidae	*Hemixos flavala*	2.8	7	HBW10, Birds of Southeast Asia
Passeriformes	Pycnonotidae	*Hemixos castanonotus*	3.0	7	HBW10, Birds of Southeast Asia
Passeriformes	Pycnonotidae	*Hypsipetes mcclellandii*	2.0	7	HBW10, BIP
Passeriformes	Pycnonotidae	*Hypsipetes madagascariensis*	3.0	6,13	HBW10, Guide to the Birds of Madagascar
Passeriformes	Pycnonotidae	*Hypsipetes leucocephalus*	2.8	4,7	HBW10, Birds of Southeast Asia
Passeriformes	Pycnonotidae	*Hypsipetes thompsoni*	2.4	7	HBW10, Birds of Southeast Asia
Passeriformes	Pycnonotidae	*Neolestes torquatus*	2.0	5	HBW10, BA
Passeriformes	Hypocoliidae	*Hypocolius ampelinus*	3.9	3,4	HBW10, BA, EMN
Passeriformes	Priniidae	*Cisticola erythrops*	2.8	5	HBW11, BA
Passeriformes	Priniidae	*Cisticola cantans*	3.0	5	HBW11, BA
Passeriformes	Priniidae	*Cisticola lateralis*	2.4	5	HBW11, BA
Passeriformes	Priniidae	*Cisticola anonymus*	2.4	5	HBW11, BA
Passeriformes	Priniidae	*Cisticola woosnami*	2.4	5	HBW11, BA
Passeriformes	Priniidae	*Cisticola bulliens*	3.0	5	HBW11, BA
Passeriformes	Priniidae	*Cisticola chubbi*	2.4	5	HBW11, BA
Passeriformes	Priniidae	*Cisticola hunteri*	2.0	5	HBW11, BA
Passeriformes	Priniidae	*Cisticola nigriloris*	2.4	5	HBW11, BA
Passeriformes	Priniidae	*Cisticola aberrans*	2.4	5	HBW11, BA
Passeriformes	Priniidae	*Cisticola bodessa*	4.0	5	HBW11, BA
Passeriformes	Priniidae	*Cisticola chiniana*	3.2	5	HBW11, BA
Passeriformes	Priniidae	*Cisticola cinereolus*	4.0	5	HBW11, BA
Passeriformes	Priniidae	*Cisticola ruficeps*	3.5	5	HBW11, BA
Passeriformes	Priniidae	*Cisticola rufilatus*	3.0	5	HBW11, BA
Passeriformes	Priniidae	*Cisticola subruficapillus*	2.8	5	HBW11, BA
Passeriformes	Priniidae	*Cisticola lais*	2.0	5	HBW11, BA
Passeriformes	Priniidae	*Cisticola njombe*	2.4	5	HBW11, BA
Passeriformes	Priniidae	*Cisticola galactotes*	3.5	5	HBW11, BA
Passeriformes	Priniidae	*Cisticola pipiens*	3.5	5	HBW11, BA
Passeriformes	Priniidae	*Cisticola carruthersi*	2.8	5	HBW11, BA
Passeriformes	Priniidae	*Cisticola tinniens*	3.0	5	HBW11, BA
Passeriformes	Priniidae	*Cisticola robustus*	2.8	5	HBW11, BA
Passeriformes	Priniidae	*Cisticola aberdare*	2.0	5	HBW11, BA
Passeriformes	Priniidae	*Cisticola natalensis*	2.4	5	HBW11, BA
Passeriformes	Priniidae	*Cisticola fulvicapillus*	3.5	5	HBW11, BA
Passeriformes	Priniidae	*Cisticola angusticauda*	3.5	5	HBW11, BA
Passeriformes	Priniidae	*Cisticola brachypterus*	3.0	5	HBW11, BA
Passeriformes	Priniidae	*Cisticola nanus*	3.5	5	HBW11, BA
Passeriformes	Priniidae	*Cisticola juncidis*	3.7	3,4,5,7,8,10	HBW11, Warblers of Europe, Asia and North Africa
Passeriformes	Priniidae	*Cisticola cherinus*	3.9	6,13	HBW11, Guide to the Birds of Madagascar
Passeriformes	Priniidae	*Cisticola aridulus*	2.8	5	HBW11, BA
Passeriformes	Priniidae	*Cisticola textrix*	2.8	5	HBW11, BA
Passeriformes	Priniidae	*Cisticola eximius*	3.5	5	HBW11, BA
Passeriformes	Priniidae	*Cisticola dambo*	2.0	5	HBW11, BA
Passeriformes	Priniidae	*Cisticola brunnescens*	2.8	5	HBW11, BA
Passeriformes	Priniidae	*Cisticola ayresii*	2.2	5	HBW11, BA
Passeriformes	Priniidae	*Cisticola exilis*	3.2	7,8,10	HBW11, Warblers of Europe, Asia and North Africa
Passeriformes	Priniidae	*Scotocerca inquieta*	3.5	3,4,5	HBW11, Warblers of Europe, Asia and North Africa
Passeriformes	Priniidae	*Prinia criniger*	4.6	7	HBW11, Warblers of Europe, Asia and North Africa; Birds of Southeast
Passeriformes	Priniidae	*Prinia polychroa*	4.0	7	HBW11, Warblers of Europe, Asia and North Africa; Birds of Southeast
Passeriformes	Priniidae	*Prinia atrogularis*	3.9	7	HBW11, Warblers of Europe, Asia and North Africa; Birds of Southeast
Passeriformes	Priniidae	*Prinia buchanani*	4.5	7	HBW11, Warblers of Europe, Asia and North Africa; Birds of Southeast
Passeriformes	Priniidae	*Prinia rufescens*	3.5	7	HBW11, Warblers of Europe, Asia and North Africa; Birds of Southeast
Passeriformes	Priniidae	*Prinia hodgsonii*	3.5	7	HBW11, Warblers of Europe, Asia and North Africa; Birds of Southeast
Passeriformes	Priniidae	*Prinia gracilis*	3.9	3,4,5,7	HBW11, Warblers of Europe, Asia and North Africa
Passeriformes	Priniidae	*Prinia sylvatica*	3.9	7	HBW11, Warblers of Europe, Asia and North Africa; Birds of Southeast
Passeriformes	Priniidae	*Prinia familiaris*	2.8	7	HBW11, Warblers of Europe, Asia and North Africa; Birds of Southeast
Passeriformes	Priniidae	*Prinia flaviventris*	3.5	7	HBW11, Warblers of Europe, Asia and North Africa; Birds of Southeast
Passeriformes	Priniidae	*Prinia socialis*	3.9	7	HBW11, Warblers of Europe, Asia and North Africa; Birds of Southeast
Passeriformes	Priniidae	*Prinia subflava*	3.5	5	HBW11, Warblers of Europe, Asia and North Africa
Passeriformes	Priniidae	*Prinia somalica*	4.0	5	HBW11, BA
Passeriformes	Priniidae	*Prinia inornata*	4.2	7	HBW11, Warblers of Europe, Asia and North Africa; Birds of Southeast
Passeriformes	Priniidae	*Prinia fluviatilis*	4.0	5	HBW11, BA
Passeriformes	Priniidae	*Prinia flavicans*	3.5	5	HBW11, BA

Order	Family	*Species*	Clutch	Range	Traits
Passeriformes	Priniidae	*Prinia maculosa*	3.0	5	HBW11, BA
Passeriformes	Priniidae	*Prinia robertsi*	2.4	14	HBW11, BA
Passeriformes	Priniidae	*Prinia leucopogon*	2.0	14	HBW11, BA
Passeriformes	Priniidae	*Prinia bairdii*	2.4	5	HBW11, BA
Passeriformes	Priniidae	*Phragmacia substriata*	3.0	5	HBW11, BA
Passeriformes	Priniidae	*Heliolais erythroptera*	2.4	5	HBW11, BA
Passeriformes	Priniidae	*Malcorus pectoralis*	3.9	5	HBW11, BA
Passeriformes	Priniidae	*Spiloptila clamans*	3.5	5	HBW11, BA
Passeriformes	Priniidae	*Spiloptila rufifrons*	3.5	14	HBW11, BA
Passeriformes	Priniidae	*Phyllolais pulchella*	2.4	5	HBW11, BA
Passeriformes	Priniidae	*Apalis pulchra*	2.4	5	HBW11, BA
Passeriformes	Priniidae	*Apalis ruwenzorii*	2.0	5	HBW11, BA
Passeriformes	Priniidae	*Apalis thoracica*	3.0	5	HBW11, BA
Passeriformes	Priniidae	*Apalis jacksoni*	2.0	5	HBW11, BA
Passeriformes	Priniidae	*Apalis chariessa*	2.4	5	HBW11, BA
Passeriformes	Priniidae	*Apalis binotata*	1.4	5	HBW11, BA
Passeriformes	Priniidae	*Apalis flavida*	2.8	5	HBW11, BA
Passeriformes	Priniidae	*Apalis ruddi*	2.0	5	HBW11, BA
Passeriformes	Priniidae	*Apalis sharpii*	3.0	5	HBW11, BA
Passeriformes	Priniidae	*Apalis melanocephala*	2.4	5	HBW11, BA
Passeriformes	Priniidae	*Apalis cinerea*	3.0	5	HBW11, BA
Passeriformes	Priniidae	*Hypergerus atriceps*	2.4	5	HBW11, BA
Passeriformes	Priniidae	*Eminia lepida*	2.4	5	HBW11, BA
Passeriformes	Priniidae	*Camaroptera brachyura*	3.0	5	HBW11, BA
Passeriformes	Priniidae	*Camaroptera superciliaris*	3.0	5	HBW11, BA
Passeriformes	Priniidae	*Camaroptera chloronota*	2.0	5	HBW11, BA
Passeriformes	Priniidae	*Calamonastes simplex*	3.5	5	HBW11, BA
Passeriformes	Priniidae	*Calamonastes undosus*	2.4	5	HBW11, BA
Passeriformes	Priniidae	*Calamonastes fasciolatus*	2.0	5	HBW11, BA
Passeriformes	Priniidae	*Poliolais lopezi*	1.4	5	HBW11, BA
Passeriformes	Priniidae	*Euryptila subcinnamomea*	2.4	5	HBW11, BA
Passeriformes	Zosteropidae	*Zosterops senegalensis*	2.0	5	BA
Passeriformes	Zosteropidae	*Zosterops vaughani*	2.0	5	BA
Passeriformes	Zosteropidae	*Zosterops poliogaster*	2.4	5	BA
Passeriformes	Zosteropidae	*Zosterops abyssinicus*	2.8	4,5	BA
Passeriformes	Zosteropidae	*Zosterops pallidus*	2.8	5	BA
Passeriformes	Zosteropidae	*Zosterops maderaspatanus*	2.4	6,13	Guide to the Birds of Madagascar
Passeriformes	Zosteropidae	*Zosterops palpebrosus*	2.8	7	Birds of Southeast Asia
Passeriformes	Zosteropidae	*Zosterops japonicus*	2.8	7,11	Birds of Southeast Asia
Passeriformes	Zosteropidae	*Zosterops citrinellus*	2.8	7,10	Complete Book of Australian Birds, BWAL
Passeriformes	Zosteropidae	*Zosterops hypoxanthus*	2.4	8	BPNG
Passeriformes	Zosteropidae	*Zosterops novaeguineae*	2.0	8	BPNG
Passeriformes	Zosteropidae	*Zosterops luteus*	3.5	10	Complete Book of Australian Birds
Passeriformes	Zosteropidae	*Zosterops griseotinctus*	2.0	8,9	BPNG
Passeriformes	Zosteropidae	*Zosterops lateralis*	2.8	9,10,11,12,13	Complete Book of Australian Birds
Passeriformes	Sylviidae	*Tesia castaneocoronata*	2.0	7	HBW11, Warblers of Europe, Asia and North Africa; Birds of Southeast
Passeriformes	Sylviidae	*Tesia cyaniventer*	3.9	7	HBW11, Warblers of Europe, Asia and North Africa; Birds of Southeast
Passeriformes	Sylviidae	*Cettia pallidipes*	3.5	7	HBW11, Warblers of Europe, Asia and North Africa; Birds of Southeast
Passeriformes	Sylviidae	*Cettia diphone*	4.2	7,11	HBW11, Warblers of Europe, Asia and North Africa
Passeriformes	Sylviidae	*Cettia ruficapilla*	2.0	11	HBW11, BHTP
Passeriformes	Sylviidae	*Cettia fortipes*	3.9	7	HBW11, Warblers of Europe, Asia and North Africa; Birds of Southeast
Passeriformes	Sylviidae	*Cettia flavolivacea*	3.5	7	HBW11, Warblers of Europe, Asia and North Africa; Birds of Southeast
Passeriformes	Sylviidae	*Cettia acanthizoides*	2.4	7	HBW11, Warblers of Europe, Asia and North Africa; Birds of Southeast
Passeriformes	Sylviidae	*Cettia brunnifrons*	3.9	7	HBW11, Warblers of Europe, Asia and North Africa; Birds of Southeast
Passeriformes	Sylviidae	*Cettia cetti*	4.5	3,4,5,7	HBW11, Warblers of Europe, Asia and North Africa
Passeriformes	Sylviidae	*Bradypterus baboecala*	2.4	5	HBW11, BA
Passeriformes	Sylviidae	*Bradypterus sylvaticus*	2.4	5	HBW11, BA
Passeriformes	Sylviidae	*Bradypterus lopezi*	2.4	5	HBW11, BA
Passeriformes	Sylviidae	*Bradypterus barratti*	2.0	5	HBW11, BA
Passeriformes	Sylviidae	*Bradypterus cinnamomeus*	2.4	5	HBW11, BA
Passeriformes	Sylviidae	*Bradypterus victorini*	2.4	5	HBW11, BA
Passeriformes	Sylviidae	*Bradypterus thoracicus*	3.5	7	HBW11, Warblers of Europe, Asia and North Africa; Birds of Southeast
Passeriformes	Sylviidae	*Bradypterus major*	3.5	7	HBW11, Warblers of Europe, Asia and North Africa; Birds of Southeast

Order	Family	*Species*	Clutch	Range	Traits
Passeriformes	Sylviidae	*Bradypterus luteoventris*	3.9	7	HBW11, Warblers of Europe, Asia and North Africa; Birds of Southeast
Passeriformes	Sylviidae	*Bathmocercus cerviniventris*	2.0	5	HBW11, BA
Passeriformes	Sylviidae	*Bathmocercus rufus*	2.0	5	HBW11, BA
Passeriformes	Sylviidae	*Nesillas typica*	2.0	6,13	HBW11, Guide to the Birds of Madagascar
Passeriformes	Sylviidae	*Thamnornis chloropetoides*	3.0	6	HBW11, Guide to the Birds of Madagascar
Passeriformes	Sylviidae	*Melocichla mentalis*	2.4	5	HBW11, BA
Passeriformes	Sylviidae	*Sphenoeacus afer*	2.4	5	HBW11, BA
Passeriformes	Sylviidae	*Locustella lanceolata*	3.9	7	HBW11, Warblers of Europe, Asia and North Africa; EMN
Passeriformes	Sylviidae	*Locustella naevia*	5.5	3,4,5,7	HBW11, Warblers of Europe, Asia and North Africa; EMN
Passeriformes	Sylviidae	*Locustella certhiola*	4.9	4,7	HBW11, Warblers of Europe, Asia and North Africa; EMN
Passeriformes	Sylviidae	*Locustella ochotensis*	5.5	7	HBW11, Warblers of Europe, Asia and North Africa
Passeriformes	Sylviidae	*Locustella pleskei*	3.9	7	HBW11, Warblers of Europe, Asia and North Africa
Passeriformes	Sylviidae	*Locustella fluviatilis*	4.9	3,4,5,7	HBW11, Warblers of Europe, Asia and North Africa; EMN
Passeriformes	Sylviidae	*Locustella luscinioides*	4.5	3,4,5,7	HBW11, Warblers of Europe, Asia and North Africa
Passeriformes	Sylviidae	*Locustella fasciolata*	3.5	7,8	HBW11, Warblers of Europe, Asia and North Africa
Passeriformes	Sylviidae	*Locustella pryeri*	5.5	7	HBW11, Warblers of Europe, Asia and North Africa
Passeriformes	Sylviidae	*Acrocephalus melanopogon*	3.9	3,4,5,7	HBW11, Warblers of Europe, Asia and North Africa
Passeriformes	Sylviidae	*Acrocephalus paludicola*	4.9	3,4,5	HBW11, Warblers of Europe, Asia and North Africa; EMN
Passeriformes	Sylviidae	*Acrocephalus schoenobaenus*	5.5	3,4,5,7	HBW11, Warblers of Europe, Asia and North Africa; EMN
Passeriformes	Sylviidae	*Acrocephalus bistrigiceps*	4.9	7	HBW11, Warblers of Europe, Asia and North Africa; EMN
Passeriformes	Sylviidae	*Acrocephalus agricola*	4.2	3,4,7	HBW11, Warblers of Europe, Asia and North Africa; EMN
Passeriformes	Sylviidae	*Acrocephalus concinens*	3.5	7	HBW11, Warblers of Europe, Asia and North Africa; EMN
Passeriformes	Sylviidae	*Acrocephalus scirpaceus*	3.9	3,4,5,7	HBW11, Warblers of Europe, Asia and North Africa
Passeriformes	Sylviidae	*Acrocephalus baeticatus*	2.4	4,5	HBW11, Warblers of Europe, Asia and North Africa; EMN
Passeriformes	Sylviidae	*Acrocephalus dumetorum*	4.2	3,4,7	HBW11, Warblers of Europe, Asia and North Africa; EMN
Passeriformes	Sylviidae	*Acrocephalus palustris*	3.9	3,4,5	HBW11, Warblers of Europe, Asia and North Africa; EMN
Passeriformes	Sylviidae	*Acrocephalus arundinaceus*	4.2	3,4,5,7,8	HBW11, Warblers of Europe, Asia and North Africa; EMN
Passeriformes	Sylviidae	*Acrocephalus stentoreus*	4.2	3,4,5,7,8,10	HBW11, Warblers of Europe, Asia and North Africa
Passeriformes	Sylviidae	*Acrocephalus australis*	2.4	8,9	HBW11, BPNG
Passeriformes	Sylviidae	*Acrocephalus rufescens*	2.4	5	HBW11, BA
Passeriformes	Sylviidae	*Acrocephalus gracilirostris*	2.4	5	HBW11, BA
Passeriformes	Sylviidae	*Acrocephalus newtoni*	3.0	6	HBW11, Guide to the Birds of Madagascar
Passeriformes	Sylviidae	*Acrocephalus aedon*	4.9	7	HBW11, Warblers of Europe, Asia and North Africa; EMN
Passeriformes	Sylviidae	*Hippolais caligata*	4.9	3,4,7	HBW11, Warblers of Europe, Asia and North Africa; EMN
Passeriformes	Sylviidae	*Hippolais rama*	4.9	4	HBW11, Warblers of Europe, Asia and North Africa; EMN
Passeriformes	Sylviidae	*Hippolais pallida*	3.0	3,4,5	HBW11, Warblers of Europe, Asia and North Africa
Passeriformes	Sylviidae	*Hippolais languida*	4.5	3,4,5,7	HBW11, Warblers of Europe, Asia and North Africa; EMN
Passeriformes	Sylviidae	*Hippolais olivetorum*	3.5	3,4,5	HBW11, Warblers of Europe, Asia and North Africa; EMN
Passeriformes	Sylviidae	*Hippolais polyglotta*	3.9	3,4,5	HBW11, Warblers of Europe, Asia and North Africa
Passeriformes	Sylviidae	*Hippolais icterina*	4.5	3,4,5,7	HBW11, Warblers of Europe, Asia and North Africa; EMN
Passeriformes	Sylviidae	*Chloropeta natalensis*	2.0	5	HBW11, BA
Passeriformes	Sylviidae	*Chloropeta similis*	2.0	5	HBW11, BA
Passeriformes	Sylviidae	*Chloropeta gracilirostris*	2.4	5	HBW11, BA
Passeriformes	Sylviidae	*Orthotomus metopias*	2.0	14	HBW11, BA
Passeriformes	Sylviidae	*Orthotomus cuculatus*	3.5	7	HBW11, Warblers of Europe, Asia and North Africa; Birds of Southeast
Passeriformes	Sylviidae	*Orthotomus sutorius*	3.5	7	HBW11, Warblers of Europe, Asia and North Africa; Birds of Southeast
Passeriformes	Sylviidae	*Orthotomus atrogularis*	3.2	7	HBW11, Warblers of Europe, Asia and North Africa; Birds of Southeast
Passeriformes	Sylviidae	*Orthotomus castaneiceps*	2.4	7	HBW11, Birds of Phillippines
Passeriformes	Sylviidae	*Orthotomus frontalis*	2.4	7	HBW11, Birds of Phillippines
Passeriformes	Sylviidae	*Orthotomus sericeus*	2.4	7	HBW11, Warblers of Europe, Asia and North Africa; Birds of Southeast
Passeriformes	Sylviidae	*Orthotomus ruficeps*	2.4	7	HBW11, Warblers of Europe, Asia and North Africa; Birds of Southeast
Passeriformes	Sylviidae	*Eremomela icteropygialis*	1.7	5	HBW11, BA
Passeriformes	Sylviidae	*Eremomela flavicrissalis*	1.4	5	HBW11, BA
Passeriformes	Sylviidae	*Eremomela pusilla*	2.0	5	HBW11, BA
Passeriformes	Sylviidae	*Eremomela scotops*	2.0	5	HBW11, BA
Passeriformes	Sylviidae	*Eremomela gregalis*	3.5	5	HBW11, BA
Passeriformes	Sylviidae	*Eremomela atricollis*	2.0	5	HBW11, BA
Passeriformes	Sylviidae	*Eremomela usticollis*	2.0	5	HBW11, BA
Passeriformes	Sylviidae	*Sylvietta virens*	2.0	5	HBW11, BA
Passeriformes	Sylviidae	*Sylvietta denti*	1.0	5	HBW11, BA
Passeriformes	Sylviidae	*Sylvietta leucophrys*	2.0	5	HBW11, BA
Passeriformes	Sylviidae	*Sylvietta brachyura*	2.0	5	HBW11, BA
Passeriformes	Sylviidae	*Sylvietta ruficapilla*	2.0	5	HBW11, BA

Order	Family	Species	Clutch	Range	Traits
Passeriformes	Sylviidae	*Sylvietta whytii*	2.0	5	HBW11, BA
Passeriformes	Sylviidae	*Sylvietta isabellina*	2.0	5	HBW11, BA
Passeriformes	Sylviidae	*Sylvietta rufescens*	2.0	5	HBW11, BA
Passeriformes	Sylviidae	*Hylia prasina*	1.0	5	HBW11, BA
Passeriformes	Sylviidae	*Phylloscopus laetus*	2.0	5	HBW11, BA
Passeriformes	Sylviidae	*Phylloscopus ruficapillus*	2.8	5	HBW11, BA
Passeriformes	Sylviidae	*Phylloscopus herberti*	3.0	5	HBW11, Warblers of Europe, Asia and North Africa; EMN
Passeriformes	Sylviidae	*Phylloscopus budongoensis*	3.0	5	HBW11, BA
Passeriformes	Sylviidae	*Phylloscopus umbrovirens*	2.4	4,5	HBW11, Warblers of Europe, Asia and North Africa
Passeriformes	Sylviidae	*Phylloscopus trochilus*	5.7	3,4,5,7	HBW11, Warblers of Europe, Asia and North Africa; EMN
Passeriformes	Sylviidae	*Phylloscopus collybita*	5.5	3,4,5,7	HBW11, Warblers of Europe, Asia and North Africa
Passeriformes	Sylviidae	*Phylloscopus sindianus*	3.2	4	HBW11, my.ort.org.il/holon/birds/bf45.html
Passeriformes	Sylviidae	*Phylloscopus neglectus*	3.9	4,7	HBW11, Warblers of Europe, Asia and North Africa; EMN
Passeriformes	Sylviidae	*Phylloscopus bonelli*	3.9	3,4,5	HBW11, Warblers of Europe, Asia and North Africa
Passeriformes	Sylviidae	*Phylloscopus orientalis*	4.9	3,4	HBW11, Warblers of Europe, Asia and North Africa; EMN
Passeriformes	Sylviidae	*Phylloscopus sibilatrix*	5.9	3,4,5,7	HBW11, Warblers of Europe, Asia and North Africa; EMN
Passeriformes	Sylviidae	*Phylloscopus fuscatus*	4.9	4,7	HBW11, Warblers of Europe, Asia and North Africa; EMN
Passeriformes	Sylviidae	*Phylloscopus affinis*	3.9	7	HBW11, Warblers of Europe, Asia and North Africa; EMN
Passeriformes	Sylviidae	*Phylloscopus subaffinis*	4.0	7	HBW11, Warblers of Europe, Asia and North Africa; Birds of Southeast
Passeriformes	Sylviidae	*Phylloscopus griseolus*	4.5	4,7	HBW11, Warblers of Europe, Asia and North Africa; EMN
Passeriformes	Sylviidae	*Phylloscopus armandii*	4.5	7	HBW11, Warblers of Europe, Asia and North Africa; EMN
Passeriformes	Sylviidae	*Phylloscopus pulcher*	3.5	7	HBW11, Warblers of Europe, Asia and North Africa; Birds of Southeast
Passeriformes	Sylviidae	*Phylloscopus proregulus*	4.9	4,7	HBW11, Warblers of Europe, Asia and North Africa; EMN
Passeriformes	Sylviidae	*Phylloscopus chloronotus*	3.9	7	HBW11, Warblers of Europe, Asia and North Africa; Birds of Southeast
Passeriformes	Sylviidae	*Phylloscopus sichuanensis*	3.9	14	HBW11, Warblers of Europe, Asia and North Africa; Birds of Southeast
Passeriformes	Sylviidae	*Phylloscopus subviridis*	3.5	4,7	HBW11, Warblers of Europe, Asia and North Africa; EMN
Passeriformes	Sylviidae	*Phylloscopus inornatus*	5.3	4,7	HBW11, Warblers of Europe, Asia and North Africa; EMN
Passeriformes	Sylviidae	*Phylloscopus humei*	4.5	4	HBW11, Warblers of Europe, Asia and North Africa; EMN
Passeriformes	Sylviidae	*Phylloscopus borealis*	5.9	2,3,4,7,8	HBW11, Warblers of Europe, Asia and North Africa; EMN
Passeriformes	Sylviidae	*Phylloscopus trochiloides*	4.6	3,4,7	HBW11, Warblers of Europe, Asia and North Africa; EMN
Passeriformes	Sylviidae	*Phylloscopus nitidus*	4.5	4	HBW11, Warblers of Europe, Asia and North Africa; EMN
Passeriformes	Sylviidae	*Phylloscopus tenellipes*	5.5	7	HBW11, Warblers of Europe, Asia and North Africa; EMN
Passeriformes	Sylviidae	*Phylloscopus borealoides*	4.9	7	HBW11, Warblers of Europe, Asia and North Africa; EMN
Passeriformes	Sylviidae	*Phylloscopus magnirostris*	3.9	7	HBW11, Warblers of Europe, Asia and North Africa; Birds of Southeast
Passeriformes	Sylviidae	*Phylloscopus tytleri*	4.5	7	HBW11, Warblers of Europe, Asia and North Africa; EMN
Passeriformes	Sylviidae	*Phylloscopus occipitalis*	3.5	4,7	HBW11, Warblers of Europe, Asia and North Africa; EMN
Passeriformes	Sylviidae	*Phylloscopus coronatus*	5.3	7	HBW11, Warblers of Europe, Asia and North Africa; EMN
Passeriformes	Sylviidae	*Phylloscopus reguloides*	4.5	7	HBW11, Warblers of Europe, Asia and North Africa; Birds of Southeast
Passeriformes	Sylviidae	*Phylloscopus cantator*	3.5	7	HBW11, Warblers of Europe, Asia and North Africa; Birds of Southeast
Passeriformes	Sylviidae	*Phylloscopus trivirgatus*	2.0	7,8	HBW11, Warblers of Europe, Asia and North Africa
Passeriformes	Sylviidae	*Seicercus xanthoschistos*	3.9	7	HBW11, Warblers of Europe, Asia and North Africa; Birds of Southeast
Passeriformes	Sylviidae	*Seicercus affinis*	4.5	7	HBW11, Warblers of Europe, Asia and North Africa; Birds of Southeast
Passeriformes	Sylviidae	*Seicercus poliogenys*	4.0	7	HBW11, Warblers of Europe, Asia and North Africa; Birds of Southeast
Passeriformes	Sylviidae	*Seicercus castaniceps*	3.9	7	HBW11, Warblers of Europe, Asia and North Africa; Birds of Southeast
Passeriformes	Sylviidae	*Seicercus montis*	2.0	7	HBW11, Warblers of Europe, Asia and North Africa; Birds of Southeast
Passeriformes	Sylviidae	*Abroscopus albogularis*	3.9	7	HBW11, Warblers of Europe, Asia and North Africa; Birds of Southeast
Passeriformes	Sylviidae	*Abroscopus schisticeps*	4.5	7	HBW11, Warblers of Europe, Asia and North Africa; Birds of Southeast
Passeriformes	Sylviidae	*Abroscopus superciliaris*	3.2	7	HBW11, Warblers of Europe, Asia and North Africa; Birds of Southeast
Passeriformes	Sylviidae	*Hyliota flavigaster*	2.0	5	HBW11, BA
Passeriformes	Sylviidae	*Hyliota australis*	3.0	5	HBW11, BA
Passeriformes	Sylviidae	*Megalurus timoriensis*	2.8	7,8,10	HBW11, Complete Book of Australian Birds
Passeriformes	Sylviidae	*Megalurus palustris*	3.5	7	HBW11, Warblers of Europe, Asia and North Africa; Birds of Southeast
Passeriformes	Sylviidae	*Megalurus albolimbatus*	3.0	8	HBW11, BPNG
Passeriformes	Sylviidae	*Megalurus gramineus*	3.9	8,10	HBW11, Complete Book of Australian Birds
Passeriformes	Sylviidae	*Megalurus punctatus*	1.7	12,13	HBW11, BNZ
Passeriformes	Sylviidae	*Cincloramphus cruralis*	3.5	10	HBW11, Complete Book of Australian Birds
Passeriformes	Sylviidae	*Cincloramphus mathewsi*	3.5	10	HBW11, Complete Book of Australian Birds
Passeriformes	Sylviidae	*Eremiornis carteri*	2.0	10	HBW11, Complete Book of Australian Birds
Passeriformes	Sylviidae	*Megalurulus mariei*	1.7	9	HBW11, BPNG
Passeriformes	Sylviidae	*Megalurulus whitneyi*	2.0	9	HBW11, BPNG
Passeriformes	Sylviidae	*Megalurulus rubiginosus*	2.0	8	HBW11, BPNG
Passeriformes	Sylviidae	*Chaetornis striatus*	3.9	7	HBW11, Warblers of Europe, Asia and North Africa
Passeriformes	Sylviidae	*Graminicola bengalensis*	4.0	7	HBW11, Warblers of Europe, Asia and North Africa; Birds of Southeast

Order	Family	*Species*	Clutch	Range	Traits
Passeriformes	Sylviidae	*Schoenicola brevirostris*	2.4	5	HBW11, BA
Passeriformes	Sylviidae	*Schoenicola platyura*	2.4	7	HBW11, Warblers of Europe, Asia and North Africa
Passeriformes	Sylviidae	*Garrulax cinereifrons*	3.0	7	BIP
Passeriformes	Sylviidae	*Garrulax perspicillatus*	3.2	7	Birds of Southeast Asia
Passeriformes	Sylviidae	*Garrulax albogularis*	3.0	7	BIP
Passeriformes	Sylviidae	*Garrulax leucolophus*	4.0	7	BIP
Passeriformes	Sylviidae	*Garrulax monileger*	4.5	7	BIP
Passeriformes	Sylviidae	*Garrulax pectoralis*	3.9	7,11	BIP
Passeriformes	Sylviidae	*Garrulax lugubris*	2.0	7	Birds of Southeast Asia
Passeriformes	Sylviidae	*Garrulax striatus*	2.0	7	BIP
Passeriformes	Sylviidae	*Garrulax ruficollis*	3.5	7	BIP
Passeriformes	Sylviidae	*Garrulax nuchalis*	3.0	7	BIP
Passeriformes	Sylviidae	*Garrulax chinensis*	3.9	7	Birds of Southeast Asia
Passeriformes	Sylviidae	*Garrulax galbanus*	3.0	7	BIP
Passeriformes	Sylviidae	*Garrulax delesserti*	3.0	7	BIP
Passeriformes	Sylviidae	*Garrulax gularis*	3.0	7	BIP
Passeriformes	Sylviidae	*Garrulax cineraceus*	2.4	7	BIP
Passeriformes	Sylviidae	*Garrulax rufogularis*	3.0	7	BIP
Passeriformes	Sylviidae	*Garrulax ocellatus*	2.0	7	BIP
Passeriformes	Sylviidae	*Garrulax caerulatus*	3.0	7,11	BIP
Passeriformes	Sylviidae	*Garrulax mitratus*	2.0	7	Birds of Southeast Asia
Passeriformes	Sylviidae	*Garrulax merulinus*	2.4	7	BIP
Passeriformes	Sylviidae	*Garrulax canorus*	3.2	7,11	Birds of Southeast Asia
Passeriformes	Sylviidae	*Garrulax sannio*	3.5	7	BIP
Passeriformes	Sylviidae	*Garrulax cachinnans*	2.4	7	BIP
Passeriformes	Sylviidae	*Garrulax jerdoni*	2.0	7	BIP
Passeriformes	Sylviidae	*Garrulax lineatus*	3.0	7	BIP
Passeriformes	Sylviidae	*Garrulax virgatus*	2.4	7	BIP
Passeriformes	Sylviidae	*Garrulax austeni*	3.5	7	BIP
Passeriformes	Sylviidae	*Garrulax squamatus*	2.4	7	BIP
Passeriformes	Sylviidae	*Garrulax subunicolor*	3.5	7	BIP
Passeriformes	Sylviidae	*Garrulax variegatus*	3.0	7	BIP
Passeriformes	Sylviidae	*Garrulax henrici*	2.4	7	BIP
Passeriformes	Sylviidae	*Garrulax affinis*	2.4	7	BIP
Passeriformes	Sylviidae	*Garrulax erythrocephalus*	2.4	7	BIP
Passeriformes	Sylviidae	*Garrulax milnei*	2.4	7	Birds of Southeast Asia
Passeriformes	Sylviidae	*Modulatrix stictigula*	2.0	14	BA
Passeriformes	Sylviidae	*Trichastoma rostratum*	2.0	7	Birds of Southeast Asia
Passeriformes	Sylviidae	*Trichastoma bicolor*	2.0	7	Birds of Southeast Asia
Passeriformes	Sylviidae	*Malacocincla abbotti*	3.9	7	BIP
Passeriformes	Sylviidae	*Malacocincla sepiarium*	2.0	7	Birds of Southeast Asia
Passeriformes	Sylviidae	*Malacocincla malaccensis*	2.4	7	Birds of Southeast Asia
Passeriformes	Sylviidae	*Malacopteron magnirostre*	2.0	7	Birds of Southeast Asia
Passeriformes	Sylviidae	*Malacopteron affine*	2.0	7	Birds of Southeast Asia
Passeriformes	Sylviidae	*Malacopteron cinereum*	2.0	7	Birds of Southeast Asia
Passeriformes	Sylviidae	*Malacopteron magnum*	2.0	7	Birds of Southeast Asia
Passeriformes	Sylviidae	*Illadopsis cleaveri*	2.0	5	BA
Passeriformes	Sylviidae	*Illadopsis albipectus*	2.0	5	BA
Passeriformes	Sylviidae	*Illadopsis puveli*	2.0	5	BA
Passeriformes	Sylviidae	*Illadopsis rufipennis*	2.0	5	BA
Passeriformes	Sylviidae	*Illadopsis fulvescens*	2.0	5	BA
Passeriformes	Sylviidae	*Illadopsis pyrrhoptera*	2.0	5	BA
Passeriformes	Sylviidae	*Kenopia striata*	2.0	7	Birds of Southeast Asia
Passeriformes	Sylviidae	*Neomixis tenella*	2.0	6	Guide to the Birds of Madagascar
Passeriformes	Sylviidae	*Neomixis striatigula*	3.9	6	Guide to the Birds of Madagascar
Passeriformes	Sylviidae	*Neomixis flavoviridis*	2.0	6	Guide to the Birds of Madagascar
Passeriformes	Sylviidae	*Macronous gularis*	3.5	7	BIP
Passeriformes	Sylviidae	*Macronous kelleyi*	4.0	7	Birds of Southeast Asia
Passeriformes	Sylviidae	*Macronous striaticeps*	3.0	7	Birds of Phillippines
Passeriformes	Sylviidae	*Macronous ptilosus*	2.4	7	Birds of Southeast Asia
Passeriformes	Sylviidae	*Turdoides nipalensis*	3.5	7	BIP
Passeriformes	Sylviidae	*Turdoides altirostris*	3.5	3,4	EMN
Passeriformes	Sylviidae	*Turdoides caudatus*	3.5	3,4,7	BIP, EMN

Order	Family	*Species*	Clutch	Range	Traits
Passeriformes	Sylviidae	*Turdoides earlei*	3.5	7	BIP
Passeriformes	Sylviidae	*Turdoides gularis*	3.9	7	Birds of Southeast Asia
Passeriformes	Sylviidae	*Turdoides longirostris*	3.9	7	Birds of Southeast Asia
Passeriformes	Sylviidae	*Turdoides malcolmi*	3.5	7	BIP
Passeriformes	Sylviidae	*Turdoides squamiceps*	3.9	3,4	EMN
Passeriformes	Sylviidae	*Turdoides fulvus*	4.5	3,4,5	BA, EMN
Passeriformes	Sylviidae	*Turdoides aylmeri*	2.0	5	BA
Passeriformes	Sylviidae	*Turdoides rubiginosus*	2.4	5	BA
Passeriformes	Sylviidae	*Turdoides subrufus*	4.0	7	BIP
Passeriformes	Sylviidae	*Turdoides striatus*	4.9	7	BIP
Passeriformes	Sylviidae	*Turdoides rufescens*	2.4	7	BIP
Passeriformes	Sylviidae	*Turdoides affinis*	3.5	7	BIP
Passeriformes	Sylviidae	*Turdoides reinwardii*	2.4	5	BA
Passeriformes	Sylviidae	*Turdoides tenebrosus*	2.4	5	BA
Passeriformes	Sylviidae	*Turdoides melanops*	3.2	5	BA
Passeriformes	Sylviidae	*Turdoides leucopygius*	3.2	5	BA
Passeriformes	Sylviidae	*Turdoides hartlaubii*	3.2	5	BA
Passeriformes	Sylviidae	*Turdoides bicolor*	3.2	5	BA
Passeriformes	Sylviidae	*Turdoides sharpei*	3.2	5	BA
Passeriformes	Sylviidae	*Turdoides hypoleucus*	3.0	5	BA
Passeriformes	Sylviidae	*Turdoides hindei*	2.4	5	BA
Passeriformes	Sylviidae	*Turdoides plebejus*	2.8	5	BA
Passeriformes	Sylviidae	*Turdoides jardineii*	3.2	5	BA
Passeriformes	Sylviidae	*Turdoides gymnogenys*	2.0	5	BA
Passeriformes	Sylviidae	*Lioptilus nigricapillus*	2.0	5	BA
Passeriformes	Sylviidae	*Oxylabes madagascariensis*	2.0	6	Guide to the Birds of Madagascar
Passeriformes	Sylviidae	*Crossleyia xanthophrys*	3.0	5,6	Guide to the Birds of Madagascar
Passeriformes	Sylviidae	*Panurus biarmicus*	5.7	3,4,7	BA
Passeriformes	Sylviidae	*Chamaea fasciata*	3.9	2	BH
Passeriformes	Sylviidae	*Sylvia buryi*	3.0	4	HBW11, Warblers of Europe, Asia and North Africa; Sylvia warblers
Passeriformes	Sylviidae	*Sylvia lugens*	2.0	14	HBW11, Sylvia warblers; BA
Passeriformes	Sylviidae	*Sylvia boehmi*	2.4	14	HBW11, Sylvia warblers; BA
Passeriformes	Sylviidae	*Sylvia layardi*	3.0	14	HBW11, Sylvia warblers; BA
Passeriformes	Sylviidae	*Sylvia subcaeruleum*	2.8	14	HBW11, Sylvia warblers; BA
Passeriformes	Sylviidae	*Sylvia atricapilla*	4.5	3,4,5,7	HBW11, Warblers of Europe, Asia and North Africa; Sylvia warblers
Passeriformes	Sylviidae	*Sylvia borin*	4.5	3,4,5,7	HBW11, Warblers of Europe, Asia and North Africa; Sylvia warblers
Passeriformes	Sylviidae	*Sylvia communis*	4.5	3,4,5,7	HBW11, Warblers of Europe, Asia and North Africa; Sylvia warblers
Passeriformes	Sylviidae	*Sylvia curruca*	4.6	3,4,5,7	HBW11, Warblers of Europe, Asia and North Africa; Sylvia warblers
Passeriformes	Sylviidae	*Sylvia althaea*	4.5	4	HBW11, Warblers of Europe, Asia and North Africa; Sylvia warblers
Passeriformes	Sylviidae	*Sylvia nana*	4.9	3,4,5,7	HBW11, Warblers of Europe, Asia and North Africa; Sylvia warblers
Passeriformes	Sylviidae	*Sylvia nisoria*	4.2	3,4,5,7	HBW11, Warblers of Europe, Asia and North Africa; Sylvia warblers
Passeriformes	Sylviidae	*Sylvia hortensis*	4.2	3,4,5	HBW11, Warblers of Europe, Asia and North Africa
Passeriformes	Sylviidae	*Sylvia leucomelaena*	2.8	3,4,5	HBW11, Warblers of Europe, Asia and North Africa; Sylvia warblers
Passeriformes	Sylviidae	*Sylvia rueppelli*	4.5	3,4,5	HBW11, Warblers of Europe, Asia and North Africa; Sylvia warblers
Passeriformes	Sylviidae	*Sylvia melanocephala*	4.5	3,4,5	HBW11, Warblers of Europe, Asia and North Africa; Sylvia warblers
Passeriformes	Sylviidae	*Sylvia melanothorax*	4.5	3,4	HBW11, Warblers of Europe, Asia and North Africa; Sylvia warblers
Passeriformes	Sylviidae	*Sylvia cantillans*	3.9	3,4,5	HBW11, Warblers of Europe, Asia and North Africa; Sylvia warblers
Passeriformes	Sylviidae	*Sylvia mystacea*	4.5	3,4,5	HBW11, Warblers of Europe, Asia and North Africa; Sylvia warblers
Passeriformes	Sylviidae	*Sylvia conspicillata*	5.0	3,4,5	HBW11, Warblers of Europe, Asia and North Africa; Sylvia warblers
Passeriformes	Sylviidae	*Sylvia deserticola*	3.9	3,4,5	HBW11, Warblers of Europe, Asia and North Africa; Sylvia warblers
Passeriformes	Sylviidae	*Sylvia undata*	4.0	3,4	HBW11, Warblers of Europe, Asia and North Africa; Sylvia warblers
Passeriformes	Sylviidae	*Sylvia sarda*	3.5	3,4,5	HBW11, Warblers of Europe, Asia and North Africa; Sylvia warblers
Passeriformes	Alaudidae	*Mirafra passerina*	2.8	5	HBW9, BA
Passeriformes	Alaudidae	*Mirafra cantillans*	2.8	4,5,7,8	HBW9, BA, BIP
Passeriformes	Alaudidae	*Mirafra javanica*	2.8	7,8,10	HBW9, Compete Book of Australian Birds; Birds of Southeast Asia; BP
Passeriformes	Alaudidae	*Mirafra cheniana*	2.8	5	HBW9, BA
Passeriformes	Alaudidae	*Mirafra albicauda*	2.0	5	HBW9, BA
Passeriformes	Alaudidae	*Mirafra hova*	2.0	6	HBW9, Guide to the Birds of Madagascar
Passeriformes	Alaudidae	*Mirafra hypermetra*	2.0	5	HBW9, BA
Passeriformes	Alaudidae	*Mirafra somalica*	3.5	5	HBW9, BA
Passeriformes	Alaudidae	*Mirafra africana*	2.8	5	HBW9, BA
Passeriformes	Alaudidae	*Mirafra angolensis*	3.0	5	HBW9, BA
Passeriformes	Alaudidae	*Mirafra rufocinnamomea*	2.4	5	HBW9, BA

Order	Family	*Species*	Clutch	Range	Traits
Passeriformes	Alaudidae	*Mirafra apiata*	2.4	5	HBW9, BA
Passeriformes	Alaudidae	*Mirafra collaris*	2.4	5	HBW9, BA
Passeriformes	Alaudidae	*Mirafra africanoides*	2.8	5	HBW9, BA
Passeriformes	Alaudidae	*Mirafra erythroptera*	2.8	7	HBW9, BIP
Passeriformes	Alaudidae	*Mirafra assamica*	3.5	7	HBW9, Birds of Southeast Asia, BIP
Passeriformes	Alaudidae	*Mirafra gilletti*	3.0	5	HBW9, BA
Passeriformes	Alaudidae	*Mirafra degodiensis*	3.0	14	HBW9, Guide to the Birds of Madagascar
Passeriformes	Alaudidae	*Mirafra poecilosterna*	2.0	5	HBW9, BA
Passeriformes	Alaudidae	*Mirafra sabota*	2.8	5	HBW9, BA
Passeriformes	Alaudidae	*Pinarocorys erythropygia*	2.0	5	HBW9, BA
Passeriformes	Alaudidae	*Pinarocorys nigricans*	2.0	5	HBW9, BA
Passeriformes	Alaudidae	*Heteromirafra archeri*	3.0	5	HBW9, BA
Passeriformes	Alaudidae	*Heteromirafra ruddi*	2.4	5	HBW9, BA
Passeriformes	Alaudidae	*Certhilauda curvirostris*	2.4	5	HBW9, BA
Passeriformes	Alaudidae	*Certhilauda erythrochlamys*	1.4	5	HBW9, BA
Passeriformes	Alaudidae	*Certhilauda albescens*	2.8	5	HBW9, BA
Passeriformes	Alaudidae	*Certhilauda burra*	2.4	5	HBW9, BA
Passeriformes	Alaudidae	*Chersomanes albofasciata*	2.4	5	HBW9, BA
Passeriformes	Alaudidae	*Eremopterix leucotis*	1.4	5	HBW9, BA
Passeriformes	Alaudidae	*Eremopterix australis*	2.8	5	HBW9, BA
Passeriformes	Alaudidae	*Eremopterix verticalis*	2.4	5	HBW9, BA
Passeriformes	Alaudidae	*Eremopterix leucopareia*	2.4	5	HBW9, BA
Passeriformes	Alaudidae	*Eremopterix signata*	4.0	4,5	HBW9, BA
Passeriformes	Alaudidae	*Eremopterix nigriceps*	2.8	3,4,5,7	HBW9, EMN
Passeriformes	Alaudidae	*Eremopterix grisea*	2.4	7	HBW9, BIP
Passeriformes	Alaudidae	*Ammomanes cincturus*	2.8	3,4,5,7	HBW9, BA, EMN
Passeriformes	Alaudidae	*Ammomanes phoenicurus*	2.8	7	HBW9, BIP
Passeriformes	Alaudidae	*Ammomanes deserti*	2.2	3,4,5,7	HBW9, BA, EMN
Passeriformes	Alaudidae	*Ammomanes grayi*	2.0	5	HBW9, BA
Passeriformes	Alaudidae	*Alaemon alaudipes*	2.8	3,4,5,7	HBW9, BA, EMN
Passeriformes	Alaudidae	*Alaemon hamertoni*	2.4	5	HBW9, BA
Passeriformes	Alaudidae	*Ramphocoris clotbey*	3.9	3,4	HBW9, BA, EMN
Passeriformes	Alaudidae	*Melanocorypha calandra*	4.2	3,4,7	HBW9, BA, EMN
Passeriformes	Alaudidae	*Melanocorypha bimaculata*	4.2	3,4,5,7	HBW9, BA, EMN
Passeriformes	Alaudidae	*Melanocorypha maxima*	2.8	7	HBW9, BIP
Passeriformes	Alaudidae	*Melanocorypha mongolica*	3.9	7	HBW9, BCh
Passeriformes	Alaudidae	*Melanocorypha leucoptera*	4.9	3,4,7	HBW9, EMN
Passeriformes	Alaudidae	*Melanocorypha yeltoniensis*	4.0	3,4,7	HBW9, EMN
Passeriformes	Alaudidae	*Calandrella brachydactyla*	3.5	3,4,5,7	HBW9, BA, EMN
Passeriformes	Alaudidae	*Calandrella cinerea*	2.8	4,5	HBW9, BA
Passeriformes	Alaudidae	*Calandrella acutirostris*	2.8	4,7	HBW9, BIP
Passeriformes	Alaudidae	*Calandrella rufescens*	3.5	3,4,5	HBW9, BA, EMN
Passeriformes	Alaudidae	*Calandrella raytal*	2.4	4,7	HBW9, Birds of Southeast Asia, BIP
Passeriformes	Alaudidae	*Calandrella somalica*	3.2	5	HBW9, BA
Passeriformes	Alaudidae	*Spizocorys conirostris*	2.4	5	HBW9, BA
Passeriformes	Alaudidae	*Spizocorys sclateri*	1.0	5	HBW9, BA
Passeriformes	Alaudidae	*Spizocorys obbiensis*	2.4	5	HBW9, BA
Passeriformes	Alaudidae	*Spizocorys fringillaris*	2.4	5	HBW9, BA
Passeriformes	Alaudidae	*Eremalauda starki*	2.4	5	HBW9, BA
Passeriformes	Alaudidae	*Eremalauda dunni*	2.4	3,4,5	HBW9, BA, EMN
Passeriformes	Alaudidae	*Galerida cristata*	3.9	3,4,5,7	HBW9, BA, EMN
Passeriformes	Alaudidae	*Galerida theklae*	3.5	3,4,5	HBW9, BA, EMN
Passeriformes	Alaudidae	*Galerida malabarica*	2.4	7	HBW9, BA
Passeriformes	Alaudidae	*Galerida deva*	2.8	7	HBW9, BIP
Passeriformes	Alaudidae	*Galerida modesta*	1.0	5	HBW9, BA
Passeriformes	Alaudidae	*Galerida magnirostris*	2.8	5	HBW9, BA
Passeriformes	Alaudidae	*Lullula arborea*	3.5	3,4,5	HBW9, BA, EMN
Passeriformes	Alaudidae	*Alauda arvensis*	2.6	3,4,7,10,11,12,13	HBW9, BA, EMN
Passeriformes	Alaudidae	*Alauda japonica*	3.9	7	HBW9, BJAP
Passeriformes	Alaudidae	*Alauda gulgula*	3.2	4,7	HBW9, EMN; Birds of Southeast Asia, HBW9
Passeriformes	Alaudidae	*Eremophila alpestris*	3.2	2,3,4,7	HBW9, BA, EMN
Passeriformes	Alaudidae	*Eremophila bilopha*	2.8	3,4	HBW9, BA, EMN
Passeriformes	Nectariniidae	*Promerops gurneyi*	1.4	5	Sunbirds

Order	Family	*Species*	Clutch	Range	Traits
Passeriformes	Nectariniidae	*Promerops cafer*	2.4	5	Sunbirds
Passeriformes	Nectariniidae	*Prionochilus maculatus*	2.0	7	Sunbirds
Passeriformes	Nectariniidae	*Prionochilus percussus*	1.0	7	Sunbirds
Passeriformes	Nectariniidae	*Dicaeum agile*	2.8	7	Sunbirds
Passeriformes	Nectariniidae	*Dicaeum chrysorrheum*	2.4	7	Sunbirds
Passeriformes	Nectariniidae	*Dicaeum melanoxanthum*	3.0	7	Sunbirds
Passeriformes	Nectariniidae	*Dicaeum vincens*	2.0	7	Sunbirds
Passeriformes	Nectariniidae	*Dicaeum haematostictum*	1.0	14	Sunbirds
Passeriformes	Nectariniidae	*Dicaeum trigonostigma*	2.4	7	Sunbirds
Passeriformes	Nectariniidae	*Dicaeum erythrorhynchos*	1.7	7	Sunbirds
Passeriformes	Nectariniidae	*Dicaeum concolor*	2.4	7	Sunbirds
Passeriformes	Nectariniidae	*Dicaeum geelvinkianum*	2.4	8	Sunbirds
Passeriformes	Nectariniidae	*Dicaeum maugei*	2.0	7	Sunbirds
Passeriformes	Nectariniidae	*Dicaeum ignipectus*	2.4	7	Sunbirds
Passeriformes	Nectariniidae	*Dicaeum celebicum*	3.0	7	Sunbirds
Passeriformes	Nectariniidae	*Dicaeum hirundinaceum*	3.5	7,8,10	Sunbirds
Passeriformes	Nectariniidae	*Dicaeum cruentatum*	2.8	7	Sunbirds
Passeriformes	Nectariniidae	*Anthreptes reichenowi*	2.4	5	Sunbirds
Passeriformes	Nectariniidae	*Anthreptes anchietae*	1.4	5	Sunbirds
Passeriformes	Nectariniidae	*Anthreptes simplex*	2.0	7	Sunbirds
Passeriformes	Nectariniidae	*Anthreptes malacensis*	2.0	7	Sunbirds
Passeriformes	Nectariniidae	*Anthreptes rhodolaema*	1.0	7	Sunbirds
Passeriformes	Nectariniidae	*Anthreptes singalensis*	2.0	7	Sunbirds; Birds of Southeast Asia
Passeriformes	Nectariniidae	*Anthreptes gabonicus*	1.4	5	Sunbirds
Passeriformes	Nectariniidae	*Anthreptes longuemarei*	1.4	5	Sunbirds
Passeriformes	Nectariniidae	*Anthreptes orientalis*	1.4	5	Sunbirds
Passeriformes	Nectariniidae	*Anthreptes aurantium*	1.4	5	Sunbirds
Passeriformes	Nectariniidae	*Anthreptes pallidigaster*	3.0	5	Sunbirds
Passeriformes	Nectariniidae	*Anthreptes rectirostris*	2.0	5	Sunbirds
Passeriformes	Nectariniidae	*Anthreptes rubritorques*	2.0	5	Sunbirds
Passeriformes	Nectariniidae	*Anthreptes collaris*	2.0	5	Sunbirds
Passeriformes	Nectariniidae	*Anthreptes platurus*	1.4	4,5	Sunbirds
Passeriformes	Nectariniidae	*Anthreptes metallicus*	1.7	3,4,5	Sunbirds
Passeriformes	Nectariniidae	*Hypogramma hypogrammicum*	2.4	7	Sunbirds
Passeriformes	Nectariniidae	*Nectarinia seimundi*	2.0	5	Sunbirds
Passeriformes	Nectariniidae	*Nectarinia batesi*	1.4	5	Sunbirds
Passeriformes	Nectariniidae	*Nectarinia olivacea*	1.4	5	Sunbirds
Passeriformes	Nectariniidae	*Nectarinia violacea*	1.4	5	Sunbirds
Passeriformes	Nectariniidae	*Nectarinia veroxii*	1.4	5	Sunbirds
Passeriformes	Nectariniidae	*Nectarinia reichenbachii*	1.0	5	Sunbirds
Passeriformes	Nectariniidae	*Nectarinia oritis*	2.0	5	Sunbirds
Passeriformes	Nectariniidae	*Nectarinia alinae*	1.4	5	Sunbirds
Passeriformes	Nectariniidae	*Nectarinia verticalis*	2.0	5	Sunbirds
Passeriformes	Nectariniidae	*Nectarinia bannermani*	2.0	5	Sunbirds
Passeriformes	Nectariniidae	*Nectarinia cyanolaema*	2.0	5	Sunbirds
Passeriformes	Nectariniidae	*Nectarinia fuliginosa*	2.0	5	Sunbirds
Passeriformes	Nectariniidae	*Nectarinia amethystina*	2.0	5	Sunbirds
Passeriformes	Nectariniidae	*Nectarinia rubescens*	2.0	5	Sunbirds
Passeriformes	Nectariniidae	*Nectarinia senegalensis*	1.7	5	Sunbirds
Passeriformes	Nectariniidae	*Nectarinia hunteri*	1.4	5	Sunbirds
Passeriformes	Nectariniidae	*Nectarinia adelberti*	4.0	5	Sunbirds
Passeriformes	Nectariniidae	*Nectarinia zeylonica*	2.4	7	Sunbirds
Passeriformes	Nectariniidae	*Nectarinia minima*	2.0	7	Sunbirds
Passeriformes	Nectariniidae	*Nectarinia sperata*	2.0	7	Sunbirds
Passeriformes	Nectariniidae	*Nectarinia aspasia*	2.4	7,8	Sunbirds
Passeriformes	Nectariniidae	*Nectarinia calcostetha*	2.0	7	Sunbirds
Passeriformes	Nectariniidae	*Nectarinia jugularis*	2.4	7,8,9,10	Sunbirds
Passeriformes	Nectariniidae	*Nectarinia sovimanga*	1.7	6,13	Sunbirds
Passeriformes	Nectariniidae	*Nectarinia venusta*	1.7	5	Sunbirds
Passeriformes	Nectariniidae	*Nectarinia ursulae*	1.4	5	Sunbirds
Passeriformes	Nectariniidae	*Nectarinia talatala*	1.4	5	Sunbirds
Passeriformes	Nectariniidae	*Nectarinia bouvieri*	2.0	5	Sunbirds
Passeriformes	Nectariniidae	*Nectarinia osea*	1.7	3,4,5	Sunbirds

Order	Family	*Species*	Clutch	Range	Traits
Passeriformes	Nectariniidae	*Nectarinia asiatica*	1.7	4,7	Sunbirds
Passeriformes	Nectariniidae	*Nectarinia habessinica*	1.4	4,5	Sunbirds
Passeriformes	Nectariniidae	*Nectarinia lotenia*	2.4	7	Sunbirds
Passeriformes	Nectariniidae	*Nectarinia manoensis*	1.7	5	Sunbirds
Passeriformes	Nectariniidae	*Nectarinia chalybea*	1.7	5	Sunbirds
Passeriformes	Nectariniidae	*Nectarinia afra*	2.0	5	Sunbirds
Passeriformes	Nectariniidae	*Nectarinia preussi*	1.4	5	Sunbirds
Passeriformes	Nectariniidae	*Nectarinia mediocris*	1.4	5	Sunbirds
Passeriformes	Nectariniidae	*Nectarinia neergaardi*	2.0	5	Sunbirds
Passeriformes	Nectariniidae	*Nectarinia chloropygia*	1.7	5	Sunbirds
Passeriformes	Nectariniidae	*Nectarinia minulla*	1.4	5	Sunbirds
Passeriformes	Nectariniidae	*Nectarinia regia*	1.0	5	Sunbirds
Passeriformes	Nectariniidae	*Nectarinia cuprea*	1.7	5	Sunbirds
Passeriformes	Nectariniidae	*Nectarinia fusca*	2.4	5	Sunbirds
Passeriformes	Nectariniidae	*Nectarinia tacazze*	1.4	5	Sunbirds
Passeriformes	Nectariniidae	*Nectarinia purpureiventris*	1.4	5	Sunbirds
Passeriformes	Nectariniidae	*Nectarinia bocagii*	2.0	14	Sunbirds
Passeriformes	Nectariniidae	*Nectarinia kilimensis*	1.4	5	Sunbirds
Passeriformes	Nectariniidae	*Nectarinia reichenowi*	1.0	5	Sunbirds
Passeriformes	Nectariniidae	*Nectarinia famosa*	2.0	5	Sunbirds
Passeriformes	Nectariniidae	*Nectarinia johnstoni*	1.4	5	Sunbirds
Passeriformes	Nectariniidae	*Nectarinia shelleyi*	1.4	5	Sunbirds
Passeriformes	Nectariniidae	*Nectarinia erythrocerca*	1.7	5	Sunbirds
Passeriformes	Nectariniidae	*Nectarinia mariquensis*	1.7	5	Sunbirds
Passeriformes	Nectariniidae	*Nectarinia bifasciata*	1.4	5	Sunbirds
Passeriformes	Nectariniidae	*Nectarinia notata*	1.4	6,13	Sunbirds
Passeriformes	Nectariniidae	*Nectarinia coccinigaster*	1.4	5	Sunbirds
Passeriformes	Nectariniidae	*Nectarinia johannae*	1.4	5	Sunbirds
Passeriformes	Nectariniidae	*Nectarinia superba*	1.4	5	Sunbirds
Passeriformes	Nectariniidae	*Nectarinia pulchella*	1.4	5	Sunbirds
Passeriformes	Nectariniidae	*Nectarinia nectarinioides*	1.4	5	Sunbirds
Passeriformes	Nectariniidae	*Arachnothera longirostra*	2.4	7	Sunbirds
Passeriformes	Nectariniidae	*Arachnothera robusta*	2.0	7	Sunbirds
Passeriformes	Nectariniidae	*Arachnothera chrysogenys*	2.0	7	Sunbirds
Passeriformes	Nectariniidae	*Arachnothera affinis*	1.4	7	Sunbirds
Passeriformes	Nectariniidae	*Arachnothera everetti*	1.4	7	Sunbirds
Passeriformes	Nectariniidae	*Arachnothera magna*	2.4	7	Sunbirds
Passeriformes	Melanocharitidae	*Toxorhamphus poliopterus*	1.0	8	BPNG
Passeriformes	Melanocharitidae	*Toxorhamphus iliolophus*	1.0	8	BPNG
Passeriformes	Passeridae	*Passer domesticus*	3.7	7,8,9,10,11,12,13	Finches & sparrows; EMN
Passeriformes	Passeridae	*Passer hispaniolensis*	4.9	3,4,5,7	Finches & sparrows; EMN
Passeriformes	Passeridae	*Passer rutilans*	4.5	7	Finches & sparrows; Birds of Southeast Asia
Passeriformes	Passeridae	*Passer flaveolus*	2.8	7	Finches & sparrows; Birds of Southeast Asia
Passeriformes	Passeridae	*Passer moabiticus*	4.5	3,4	Finches & sparrows; EMN
Passeriformes	Passeridae	*Passer motitensis*	3.2	5	Finches & sparrows; EMN
Passeriformes	Passeridae	*Passer melanurus*	3.5	5	Finches & sparrows; EMN
Passeriformes	Passeridae	*Passer griseus*	3.2	5	Finches & sparrows; EMN
Passeriformes	Passeridae	*Passer simplex*	3.2	3,4,5	Finches & sparrows; EMN
Passeriformes	Passeridae	*Passer montanus*	3.7	2,3,4,5,7,8,10,11	Finches & sparrows; EMN
Passeriformes	Passeridae	*Passer luteus*	3.9	4,5	Finches & sparrows; EMN
Passeriformes	Passeridae	*Passer eminibey*	3.5	5	Finches & sparrows; EMN
Passeriformes	Passeridae	*Petronia xanthocollis*	3.5	4,7	Finches & sparrows; EMN
Passeriformes	Passeridae	*Petronia superciliaris*	3.2	3,5	Finches & sparrows; EMN
Passeriformes	Passeridae	*Petronia dentata*	3.0	4,5	Finches & sparrows; EMN
Passeriformes	Passeridae	*Petronia petronia*	5.3	3,4,5,7	Finches & sparrows; EMN
Passeriformes	Passeridae	*Montifringilla nivalis*	4.5	3,4,7	Finches & sparrows; EMN
Passeriformes	Passeridae	*Dendronanthus indicus*	4.5	4,7	HBW9, Birds of Southeast Asia
Passeriformes	Passeridae	*Motacilla alba*	4.9	2,3,4,5,7	HBW9, EMN
Passeriformes	Passeridae	*Motacilla grandis*	5.3	7	HBW9, BJAP
Passeriformes	Passeridae	*Motacilla madaraspatensis*	3.9	7	HBW9, BIS
Passeriformes	Passeridae	*Motacilla aguimp*	3.5	5	HBW9, BA, EMN
Passeriformes	Passeridae	*Motacilla capensis*	2.0	5	HBW9, BA
Passeriformes	Passeridae	*Motacilla flaviventris*	2.0	6	HBW9, Guide to the Birds of Madagascar

Order	Family	*Species*	Clutch	Range	Traits
Passeriformes	Passeridae	*Motacilla citreola*	4.2	3,4,5,7	HBW9, BA, EMN
Passeriformes	Passeridae	*Motacilla flava*	4.9	2,3,4,5,7,8,10,11	HBW9, BA, EMN
Passeriformes	Passeridae	*Motacilla cinerea*	4.6	2,3,4,5,7,8	HBW9, BA, EMN
Passeriformes	Passeridae	*Motacilla clara*	2.4	5	HBW9, BA
Passeriformes	Passeridae	*Tmetothylacus tenellus*	2.8	4,5	HBW9, BA
Passeriformes	Passeridae	*Macronyx croceus*	2.8	5	HBW9, BA
Passeriformes	Passeridae	*Macronyx fuellebornii*	2.4	5	HBW9, BA
Passeriformes	Passeridae	*Macronyx capensis*	3.2	5	HBW9, BA
Passeriformes	Passeridae	*Macronyx flavicollis*	2.8	5	HBW9, BA
Passeriformes	Passeridae	*Macronyx sharpei*	2.4	5	HBW9, BA
Passeriformes	Passeridae	*Macronyx ameliae*	2.8	5	HBW9, BA
Passeriformes	Passeridae	*Macronyx aurantiigula*	2.8	5	HBW9, BA
Passeriformes	Passeridae	*Anthus chloris*	3.0	5	HBW9, BA
Passeriformes	Passeridae	*Anthus lineiventris*	2.4	5	HBW9, BA
Passeriformes	Passeridae	*Anthus crenatus*	3.5	5	HBW9, BA
Passeriformes	Passeridae	*Anthus hoeschi*	3.5	5	HBW9, BA
Passeriformes	Passeridae	*Anthus richardi*	3.5	4,5,7,8	HBW9, BA
Passeriformes	Passeridae	*Anthus rufulus*	3.5	7,8	HBW9, Birds of Southeast Asia, HBW9
Passeriformes	Passeridae	*Anthus novaeseelandiae*	3.2	8,10,12,13	HBW9, BA, EMN, BPNG, HBW9
Passeriformes	Passeridae	*Anthus leucophrys*	2.4	5	HBW9, BA
Passeriformes	Passeridae	*Anthus vaalensis*	2.4	5	HBW9, BA
Passeriformes	Passeridae	*Anthus melindae*	2.4	5	HBW9, BA
Passeriformes	Passeridae	*Anthus campestris*	4.2	3,4,5,7	HBW9, BA, EMN
Passeriformes	Passeridae	*Anthus berthelotii*	3.2	3	HBW9, EMN
Passeriformes	Passeridae	*Anthus godlewskii*	3.9	4,7	HBW9, EMN
Passeriformes	Passeridae	*Anthus similis*	2.4	3,4,5,7	HBW9, BA, EMN
Passeriformes	Passeridae	*Anthus brachyurus*	2.4	5	HBW9, BA
Passeriformes	Passeridae	*Anthus caffer*	2.4	5	HBW9, BA
Passeriformes	Passeridae	*Anthus trivialis*	4.0	3,4,5,7	HBW9, BA, EMN
Passeriformes	Passeridae	*Anthus hodgsoni*	2.4	3,4,7	HBW9, EMN
Passeriformes	Passeridae	*Anthus gustavi*	4.9	3,4,7	HBW9, EMN
Passeriformes	Passeridae	*Anthus pratensis*	3.7	3,4,5	HBW9, BA, EMN
Passeriformes	Passeridae	*Anthus cervinus*	3.7	2,3,4,5,7	HBW9, BA, EMN
Passeriformes	Passeridae	*Anthus roseatus*	3.5	7	HBW9, Birds of Southeast Asia
Passeriformes	Passeridae	*Anthus petrosus*	4.9	3,4	HBW9, BA, EMN
Passeriformes	Passeridae	*Anthus spinoletta*	4.9	3,4,5,7	HBW9, BA, EMN
Passeriformes	Passeridae	*Anthus rubescens*	4.6	2,7	HBW9, Birds of Southeast Asia
Passeriformes	Passeridae	*Anthus sylvanus*	3.9	7	HBW9, BIS
Passeriformes	Passeridae	*Anthus nilghiriensis*	2.8	7	HBW9, BIS
Passeriformes	Passeridae	*Anthus correndera*	2.8	2	HBW9, BSA
Passeriformes	Passeridae	*Anthus spragueii*	4.5	2	HBW9, BMx
Passeriformes	Passeridae	*Anthus lutescens*	2.8	2	HBW9, Birds of Venezuela; BSA
Passeriformes	Passeridae	*Anthus nattereri*	4.0	2	HBW9, BSA
Passeriformes	Passeridae	*Prunella collaris*	3.5	3,4,7	HBW10, BA, EMN
Passeriformes	Passeridae	*Prunella strophiata*	3.9	7	HBW10, Birds of Southeast Asia
Passeriformes	Passeridae	*Prunella montanella*	4.9	3,4,7	HBW10, EMN
Passeriformes	Passeridae	*Prunella ocularis*	3.5	3,4	HBW10, EMN
Passeriformes	Passeridae	*Prunella atrogularis*	3.5	3,4,7	HBW10, EMN
Passeriformes	Passeridae	*Prunella modularis*	4.9	3,4,5,12,13	HBW10, BA, EMN
Passeriformes	Passeridae	*Prunella rubida*	3.5	7	HBW10, BJAP
Passeriformes	Passeridae	*Prunella immaculata*	3.9	7	HBW10, Birds of Southeast Asia
Passeriformes	Passeridae	*Bubalornis albirostris*	2.8	5	BA
Passeriformes	Passeridae	*Dinemellia dinemelli*	2.8	5	BA
Passeriformes	Passeridae	*Sporopipes frontalis*	3.7	5	Roberts' Web page/SA Bird Atlas
Passeriformes	Passeridae	*Sporopipes squamifrons*	3.7	5	Roberts' Web page/SA Bird Atlas
Passeriformes	Passeridae	*Plocepasser mahali*	1.7	5	BA
Passeriformes	Passeridae	*Plocepasser superciliosus*	2.0	5	BA
Passeriformes	Passeridae	*Histurgops ruficauda*	3.0	5	BA
Passeriformes	Passeridae	*Pseudonigrita arnaudi*	3.9	5	BA
Passeriformes	Passeridae	*Pseudonigrita cabanisi*	2.8	5	BA
Passeriformes	Passeridae	*Philetairus socius*	3.5	5	BA
Passeriformes	Passeridae	*Ploceus baglafecht*	2.4	5	BA
Passeriformes	Passeridae	*Ploceus pelzelni*	2.4	5	BA

Order	Family	*Species*	Clutch	Range	Traits
Passeriformes	Passeridae	*Ploceus luteolus*	2.4	5	BA
Passeriformes	Passeridae	*Ploceus intermedius*	2.8	4,5	BA
Passeriformes	Passeridae	*Ploceus ocularis*	2.8	5	BA
Passeriformes	Passeridae	*Ploceus nigricollis*	1.4	5	BA
Passeriformes	Passeridae	*Ploceus temporalis*	2.8	5	BA, Adrian Craig
Passeriformes	Passeridae	*Ploceus capensis*	2.8	5	BA
Passeriformes	Passeridae	*Ploceus subaureus*	2.8	5	BA
Passeriformes	Passeridae	*Ploceus xanthops*	2.4	5	BA
Passeriformes	Passeridae	*Ploceus aurantius*	2.0	5	BA
Passeriformes	Passeridae	*Ploceus castaneiceps*	2.4	5	BA
Passeriformes	Passeridae	*Ploceus xanthopterus*	2.8	5	BA
Passeriformes	Passeridae	*Ploceus heuglini*	2.4	5	BA
Passeriformes	Passeridae	*Ploceus velatus*	2.8	5	BA
Passeriformes	Passeridae	*Ploceus cucullatus*	2.8	2,5,13	EMN, BWA, Birds of the West Indies
Passeriformes	Passeridae	*Ploceus spekei*	1.7	5	BA
Passeriformes	Passeridae	*Ploceus nigerrimus*	2.0	5	BA
Passeriformes	Passeridae	*Ploceus melanocephalus*	2.4	5	BA
Passeriformes	Passeridae	*Ploceus jacksoni*	2.4	5	BA
Passeriformes	Passeridae	*Ploceus badius*	2.4	5	BA, Adrian Craig
Passeriformes	Passeridae	*Ploceus rubiginosus*	3.5	5	BA
Passeriformes	Passeridae	*Ploceus nelicourvi*	3.0	6	Guide to the Birds of Madagascar
Passeriformes	Passeridae	*Ploceus sakalava*	3.5	6	Guide to the Birds of Madagascar
Passeriformes	Passeridae	*Ploceus benghalensis*	3.2	7	Birds of Southeast Asia
Passeriformes	Passeridae	*Ploceus manyar*	2.4	5,7	EMN
Passeriformes	Passeridae	*Ploceus philippinus*	3.2	7	Birds of Southeast Asia
Passeriformes	Passeridae	*Ploceus hypoxanthus*	2.8	7	Birds of Southeast Asia
Passeriformes	Passeridae	*Ploceus megarhynchus*	2.8	7	BIS
Passeriformes	Passeridae	*Ploceus bicolor*	2.8	5	BA
Passeriformes	Passeridae	*Ploceus olivaceiceps*	2.4	5	BA
Passeriformes	Passeridae	*Ploceus angolensis*	2.0	5	BA, Adrian Craig
Passeriformes	Passeridae	*Pachyphantes superciliosus*	3.5	5	BA
Passeriformes	Passeridae	*Malimbus scutatus*	2.0	5	BA
Passeriformes	Passeridae	*Malimbus nitens*	2.0	5	BA
Passeriformes	Passeridae	*Malimbus malimbicus*	2.0	5	BA
Passeriformes	Passeridae	*Anaplectes rubriceps*	2.4	5	BA
Passeriformes	Passeridae	*Quelea cardinalis*	2.4	5	BA
Passeriformes	Passeridae	*Quelea erythrops*	2.0	5	BA
Passeriformes	Passeridae	*Quelea quelea*	2.2	5	BA
Passeriformes	Passeridae	*Euplectes afer*	3.2	2,5	BA, Birds of the West Indies
Passeriformes	Passeridae	*Euplectes hordeaceus*	3.0	5	BA, Birds of the West Indies
Passeriformes	Passeridae	*Euplectes franciscanus*	2.8	2,5	BA, Birds of the West Indies
Passeriformes	Passeridae	*Euplectes orix*	3.2	5	BA, Birds of the West Indies
Passeriformes	Passeridae	*Euplectes capensis*	2.8	5	BA, Birds of the West Indies
Passeriformes	Passeridae	*Euplectes axillaris*	2.8	5	BA, Birds of the West Indies
Passeriformes	Passeridae	*Euplectes macrourus*	2.4	5	BA, Birds of the West Indies
Passeriformes	Passeridae	*Euplectes albonotatus*	3.2	5	BA, Birds of the West Indies
Passeriformes	Passeridae	*Euplectes ardens*	3.5	5	BA, Birds of the West Indies
Passeriformes	Passeridae	*Euplectes progne*	2.8	5	BA, Birds of the West Indies
Passeriformes	Passeridae	*Euplectes jacksoni*	2.4	5	BA, Birds of the West Indies
Passeriformes	Passeridae	*Parmoptila woodhousei*	3.5	5	Finches & Sparrows; Estrildid finches of the world
Passeriformes	Passeridae	*Nigrita fusconota*	4.2	5	Finches & Sparrows; Estrildid finches of the world
Passeriformes	Passeridae	*Nigrita bicolor*	2.0	5	Finches & Sparrows; Estrildid finches of the world
Passeriformes	Passeridae	*Nigrita canicapilla*	4.9	5	Finches & Sparrows; Estrildid finches of the world
Passeriformes	Passeridae	*Nesocharis ansorgei*	3.0	5	Finches & Sparrows; Estrildid finches of the world
Passeriformes	Passeridae	*Nesocharis capistrata*	4.0	5	Finches & Sparrows; Estrildid finches of the world
Passeriformes	Passeridae	*Pytilia phoenicoptera*	3.5	5	Finches & Sparrows; Estrildid finches of the world
Passeriformes	Passeridae	*Pytilia afra*	3.5	5	Finches & Sparrows; Estrildid finches of the world
Passeriformes	Passeridae	*Pytilia melba*	4.2	4,5	Finches & Sparrows; Estrildid finches of the world
Passeriformes	Passeridae	*Pytilia hypogrammica*	3.5	5	Finches & Sparrows; Estrildid finches of the world
Passeriformes	Passeridae	*Mandingoa nitidula*	4.2	5	Finches & Sparrows; Estrildid finches of the world
Passeriformes	Passeridae	*Cryptospiza reichenovii*	3.5	5	Finches & Sparrows; Estrildid finches of the world
Passeriformes	Passeridae	*Cryptospiza salvadorii*	3.9	5	Finches & Sparrows; Estrildid finches of the world
Passeriformes	Passeridae	*Pyrenestes sanguineus*	3.5	5	Finches & Sparrows; Estrildid finches of the world

Order	Family	*Species*	Clutch	Range	Traits
Passeriformes	Passeridae	*Pyrenestes ostrinus*	3.9	5	Finches & Sparrows; Estrildid finches of the world
Passeriformes	Passeridae	*Spermophaga haematina*	4.2	5	Finches & Sparrows; Estrildid finches of the world
Passeriformes	Passeridae	*Spermophaga ruficapilla*	3.0	5	Finches & Sparrows; Estrildid finches of the world
Passeriformes	Passeridae	*Clytospiza monteiri*	4.9	5	Finches & Sparrows; Estrildid finches of the world
Passeriformes	Passeridae	*Hypargos niveoguttatus*	4.2	5	Finches & Sparrows; Estrildid finches of the world
Passeriformes	Passeridae	*Hypargos margaritatus*	3.5	5	Finches & Sparrows; Estrildid finches of the world
Passeriformes	Passeridae	*Lagonosticta rufopicta*	4.0	5	Finches & Sparrows; Estrildid finches of the world
Passeriformes	Passeridae	*Lagonosticta senegala*	3.5	5	Finches & Sparrows; EMN; Estrildid finches of the world
Passeriformes	Passeridae	*Lagonosticta rara*	3.5	5	Finches & Sparrows; Estrildid finches of the world
Passeriformes	Passeridae	*Lagonosticta rubricata*	3.9	5	Finches & Sparrows; Estrildid finches of the world
Passeriformes	Passeridae	*Lagonosticta virata*	4.0	5	Finches & Sparrows; Estrildid finches of the world
Passeriformes	Passeridae	*Lagonosticta rhodopareia*	3.2	5	Finches & Sparrows; Estrildid finches of the world
Passeriformes	Passeridae	*Lagonosticta larvata*	3.5	5	Finches & Sparrows; Estrildid finches of the world
Passeriformes	Passeridae	*Uraeginthus angolensis*	4.2	5	Finches & Sparrows; Estrildid finches of the world
Passeriformes	Passeridae	*Uraeginthus bengalus*	4.2	5,11	Finches & Sparrows; Estrildid finches of the world
Passeriformes	Passeridae	*Uraeginthus cyanocephalus*	4.9	5	Finches & Sparrows; Estrildid finches of the world
Passeriformes	Passeridae	*Uraeginthus granatina*	3.2	5	Finches & Sparrows; Estrildid finches of the world
Passeriformes	Passeridae	*Estrilda caerulescens*	3.2	5,11	Finches & Sparrows; Estrildid finches of the world
Passeriformes	Passeridae	*Estrilda perreini*	6.3	5	Finches & Sparrows; Estrildid finches of the world
Passeriformes	Passeridae	*Estrilda thomensis*	3.5	5	Finches & Sparrows; Estrildid finches of the world
Passeriformes	Passeridae	*Estrilda melanotis*	5.2	5	Finches & Sparrows; Estrildid finches of the world
Passeriformes	Passeridae	*Estrilda paludicola*	4.9	5	Finches & Sparrows; Estrildid finches of the world
Passeriformes	Passeridae	*Estrilda melpoda*	4.9	2,5,11,13	Finches & Sparrows; Estrildid finches of the world
Passeriformes	Passeridae	*Estrilda troglodytes*	4.5	2,5	Finches & Sparrows; Estrildid finches of the world
Passeriformes	Passeridae	*Estrilda astrild*	4.9	2,3,5,6,9,11,13	Finches & Sparrows; EMN; Estrildid finches of the world
Passeriformes	Passeridae	*Estrilda nonnula*	5.5	5	Finches & Sparrows; Estrildid finches of the world
Passeriformes	Passeridae	*Estrilda atricapilla*	2.0	5	Finches & Sparrows; Estrildid finches of the world
Passeriformes	Passeridae	*Estrilda charmosyna*	4.0	5	Finches & Sparrows; Estrildid finches of the world
Passeriformes	Passeridae	*Estrilda erythronotos*	4.2	5	Finches & Sparrows; Estrildid finches of the world
Passeriformes	Passeridae	*Ortygospiza atricollis*	4.9	5	Finches & Sparrows; Estrildid finches of the world
Passeriformes	Passeridae	*Ortygospiza locustella*	5.3	5	Finches & Sparrows; Estrildid finches of the world
Passeriformes	Passeridae	*Emblema pictum*	3.5	10	Finches & Sparrows; Estrildid finches of the world
Passeriformes	Passeridae	*Stagonopleura bella*	4.5	10	Finches & Sparrows; Estrildid finches of the world
Passeriformes	Passeridae	*Stagonopleura oculata*	4.9	10	Finches & Sparrows; Estrildid finches of the world
Passeriformes	Passeridae	*Stagonopleura guttata*	5.3	10	Finches & Sparrows; Estrildid finches of the world
Passeriformes	Passeridae	*Neochmia temporalis*	4.9	10,11	Finches & Sparrows; Estrildid finches of the world
Passeriformes	Passeridae	*Neochmia phaeton*	6.3	8,10	Finches & Sparrows; Estrildid finches of the world
Passeriformes	Passeridae	*Neochmia ruficauda*	4.2	10	Finches & Sparrows; Estrildid finches of the world
Passeriformes	Passeridae	*Neochmia modesta*	5.5	10	Finches & Sparrows; Estrildid finches of the world
Passeriformes	Passeridae	*Taeniopygia guttata*	4.5	7,10	Finches & Sparrows; Estrildid finches of the world
Passeriformes	Passeridae	*Taeniopygia bichenovii*	4.5	10	Finches & Sparrows; Estrildid finches of the world
Passeriformes	Passeridae	*Poephila personata*	4.9	10	Finches & Sparrows; Estrildid finches of the world
Passeriformes	Passeridae	*Poephila acuticauda*	4.5	10	Finches & Sparrows; Estrildid finches of the world
Passeriformes	Passeridae	*Poephila cincta*	4.5	10	Finches & Sparrows; Estrildid finches of the world
Passeriformes	Passeridae	*Erythrura hyperythra*	4.0	7	Finches & Sparrows; Estrildid finches of the world
Passeriformes	Passeridae	*Erythrura prasina*	4.9	7	Finches & Sparrows; Estrildid finches of the world
Passeriformes	Passeridae	*Erythrura viridifacies*	3.0	7	Finches & Sparrows; Estrildid finches of the world
Passeriformes	Passeridae	*Erythrura trichroa*	3.5	7,8,9,10,11	Finches & Sparrows; Estrildid finches of the world
Passeriformes	Passeridae	*Erythrura coloria*	1.4	7	Finches & Sparrows; Estrildid finches of the world
Passeriformes	Passeridae	*Erythrura psittacea*	4.0	9	Finches & Sparrows; Estrildid finches of the world
Passeriformes	Passeridae	*Erythrura pealii*	3.5	13	Finches & Sparrows; Estrildid finches of the world
Passeriformes	Passeridae	*Erythrura cyaneovirens*	3.5	9,11	Finches & Sparrows; Estrildid finches of the world
Passeriformes	Passeridae	*Erythrura regia*	2.8	9	Finches & Sparrows; Estrildid finches of the world
Passeriformes	Passeridae	*Erythrura gouldiae*	5.7	10	Finches & Sparrows; Estrildid finches of the world
Passeriformes	Passeridae	*Lemuresthes nana*	4.9	6	Finches & Sparrows; Estrildid finches of the world
Passeriformes	Passeridae	*Lonchura cantans*	4.2	4,5	Finches & Sparrows; Estrildid finches of the world
Passeriformes	Passeridae	*Lonchura malabarica*	4.6	2,4,7,11	Finches & Sparrows; Estrildid finches of the world
Passeriformes	Passeridae	*Lonchura striata*	5.0	7	Finches & Sparrows; Estrildid finches of the world
Passeriformes	Passeridae	*Lonchura leucogastroides*	5.0	7	Finches & Sparrows; Estrildid finches of the world
Passeriformes	Passeridae	*Lonchura fuscans*	4.2	7	Finches & Sparrows; Estrildid finches of the world
Passeriformes	Passeridae	*Lonchura molucca*	4.5	7	Finches & Sparrows; Estrildid finches of the world
Passeriformes	Passeridae	*Lonchura kelaarti*	5.0	7	Finches & Sparrows; Estrildid finches of the world
Passeriformes	Passeridae	*Lonchura punctulata*	6.3	2,7,10,11,13	Finches & Sparrows; Estrildid finches of the world

Order	Family	*Species*	Clutch	Range	Traits
Passeriformes	Passeridae	*Lonchura leucogastra*	4.9	7	Finches & Sparrows; Estrildid finches of the world; Birds of Southeast A
Passeriformes	Passeridae	*Lonchura malacca*	5.5	2,7,11	Finches & Sparrows; Estrildid finches of the world
Passeriformes	Passeridae	*Lonchura maja*	4.6	7	Finches & Sparrows; Estrildid finches of the world
Passeriformes	Passeridae	*Lonchura pallida*	4.5	7	Finches & Sparrows; Estrildid finches of the world
Passeriformes	Passeridae	*Lonchura grandis*	5.5	8	Finches & Sparrows; Estrildid finches of the world
Passeriformes	Passeridae	*Lonchura nevermanni*	4.5	8	Finches & Sparrows; Estrildid finches of the world
Passeriformes	Passeridae	*Lonchura spectabilis*	3.9	8	Finches & Sparrows; Estrildid finches of the world
Passeriformes	Passeridae	*Lonchura flaviprymna*	4.5	10	Finches & Sparrows; Estrildid finches of the world
Passeriformes	Passeridae	*Lonchura castaneothorax*	4.9	8,9,10,11	Finches & Sparrows; Estrildid finches of the world
Passeriformes	Passeridae	*Lonchura stygia*	4.5	8	Finches & Sparrows; Estrildid finches of the world
Passeriformes	Passeridae	*Lonchura melaena*	4.2	8,9	Finches & Sparrows; Estrildid finches of the world
Passeriformes	Passeridae	*Lonchura oryzivora*	4.9	2,5,7,11,13	Finches & Sparrows; Estrildid finches of the world
Passeriformes	Passeridae	*Lonchura fuscata*	4.2	7	Finches & Sparrows; Estrildid finches of the world
Passeriformes	Passeridae	*Heteromunia pectoralis*	4.9	10	Finches & Sparrows; Estrildid finches of the world
Passeriformes	Passeridae	*Amadina erythrocephala*	4.9	5	Finches & Sparrows; Estrildid finches of the world
Passeriformes	Fringillidae	*Peucedramus taeniatus*	3.5	2	Warblers of the Americas
Passeriformes	Fringillidae	*Fringilla coelebs*	4.5	3,4,5,7,12,13	Finches & sparrows; EMN
Passeriformes	Fringillidae	*Fringilla teydea*	2.0	13	Finches & sparrows; EMN
Passeriformes	Fringillidae	*Fringilla montifringilla*	5.9	4,5,7	Finches & sparrows; EMN
Passeriformes	Fringillidae	*Serinus pusillus*	3.9	3,4,7	Finches & sparrows; EMN
Passeriformes	Fringillidae	*Serinus serinus*	3.5	3,4,5	Finches & sparrows; EMN
Passeriformes	Fringillidae	*Serinus syriacus*	4.0	3,4	Finches & sparrows; EMN
Passeriformes	Fringillidae	*Serinus canaria*	3.5	3,11	Finches & sparrows; EMN
Passeriformes	Fringillidae	*Serinus citrinella*	4.5	3	Finches & sparrows; EMN
Passeriformes	Fringillidae	*Serinus canicollis*	3.2	5,13	Finches & sparrows; EMN
Passeriformes	Fringillidae	*Serinus citrinelloides*	2.4	5	Finches & sparrows; EMN
Passeriformes	Fringillidae	*Serinus scotops*	2.8	5	Finches & sparrows; EMN
Passeriformes	Fringillidae	*Serinus leucopygius*	2.8	5	Finches & sparrows; EMN
Passeriformes	Fringillidae	*Serinus atrogularis*	2.8	5	Finches & sparrows; EMN
Passeriformes	Fringillidae	*Serinus citrinipectus*	3.5	5	Finches & sparrows; EMN
Passeriformes	Fringillidae	*Serinus mozambicus*	3.2	2,5,11,13	Finches & sparrows; EMN
Passeriformes	Fringillidae	*Serinus donaldsoni*	3.0	5	Finches & sparrows; EMN
Passeriformes	Fringillidae	*Serinus dorsostriatus*	2.8	5	Finches & sparrows; EMN
Passeriformes	Fringillidae	*Serinus flaviventris*	2.8	5	Finches & sparrows; EMN
Passeriformes	Fringillidae	*Serinus sulphuratus*	2.8	5	Finches & sparrows; EMN
Passeriformes	Fringillidae	*Serinus albogularis*	2.8	5	Finches & sparrows; EMN
Passeriformes	Fringillidae	*Serinus gularis*	2.8	5	Finches & sparrows; EMN
Passeriformes	Fringillidae	*Serinus mennelli*	2.8	5	Finches & sparrows; EMN
Passeriformes	Fringillidae	*Serinus striolatus*	3.5	5	Finches & sparrows; EMN
Passeriformes	Fringillidae	*Serinus leucopterus*	2.8	5	Finches & sparrows; EMN
Passeriformes	Fringillidae	*Serinus totta*	3.9	5	Finches & sparrows; EMN
Passeriformes	Fringillidae	*Serinus alario*	2.8	5	Finches & sparrows; EMN
Passeriformes	Fringillidae	*Linurgus olivaceus*	2.4	5	Finches & sparrows
Passeriformes	Fringillidae	*Carduelis chloris*	4.9	3,4,5,10,12,13	Finches & sparrows; EMN
Passeriformes	Fringillidae	*Carduelis sinica*	3.2	7	Finches & sparrows; Birds of Southeast Asia
Passeriformes	Fringillidae	*Carduelis spinoides*	3.9	7	Finches & sparrows; Birds of Southeast Asia
Passeriformes	Fringillidae	*Carduelis ambigua*	3.5	7	Finches & sparrows; Birds of Southeast Asia
Passeriformes	Fringillidae	*Carduelis spinus*	3.9	3,4,5,7	Finches & sparrows; EMN
Passeriformes	Fringillidae	*Carduelis pinus*	3.5	2	Finches & sparrows
Passeriformes	Fringillidae	*Carduelis notata*	2.0	2	Finches & sparrows
Passeriformes	Fringillidae	*Carduelis xanthogastra*	2.4	2	Finches & sparrows; Birds of Costa Rica
Passeriformes	Fringillidae	*Carduelis tristis*	4.9	2	Finches & sparrows
Passeriformes	Fringillidae	*Carduelis psaltria*	3.5	2	Finches & sparrows; Birds of Costa Rica
Passeriformes	Fringillidae	*Carduelis lawrencei*	4.5	2	Finches & sparrows
Passeriformes	Fringillidae	*Carduelis dominicensis*	2.4	2	Finches & sparrows, Birds of the West Indies
Passeriformes	Fringillidae	*Carduelis carduelis*	4.9	,3,4,5,7,10,12,13	Finches & sparrows; EMN
Passeriformes	Fringillidae	*Carduelis hornemanni*	4.5	2,3,7	Finches & sparrows; EMN
Passeriformes	Fringillidae	*Carduelis flammea*	4.9	2,3,4,7,12,13	Finches & sparrows; EMN
Passeriformes	Fringillidae	*Carduelis flavirostris*	4.9	3,4,7	Finches & sparrows; EMN
Passeriformes	Fringillidae	*Carduelis cannabina*	4.9	3,4,5,7	Finches & sparrows; EMN
Passeriformes	Fringillidae	*Leucosticte nemoricola*	3.5	7	Finches & sparrows; Birds of Southeast Asia
Passeriformes	Fringillidae	*Leucosticte arctoa*	4.5	7	Finches & sparrows; Birds of Southeast Asia
Passeriformes	Fringillidae	*Leucosticte tephrocotis*	4.5	2	Finches & sparrows

Order	Family	*Species*	Clutch	Range	Traits
Passeriformes	Fringillidae	*Leucosticte atrata*	4.5	2	Finches & sparrows
Passeriformes	Fringillidae	*Leucosticte australis*	4.5	2	Finches & sparrows
Passeriformes	Fringillidae	*Rhodopechys sanguinea*	4.5	3,4,5,7	Finches & sparrows; EMN
Passeriformes	Fringillidae	*Rhodopechys githaginea*	4.9	3,4,5,7	Finches & sparrows; EMN
Passeriformes	Fringillidae	*Rhodopechys mongolica*	4.9	4,7	Finches & sparrows
Passeriformes	Fringillidae	*Rhodopechys obsoleta*	4.9	3,4,7	Finches & sparrows; EMN
Passeriformes	Fringillidae	*Carpodacus erythrinus*	4.9	3,4,7	Finches & sparrows; EMN
Passeriformes	Fringillidae	*Carpodacus purpureus*	4.5	2	Finches & sparrows
Passeriformes	Fringillidae	*Carpodacus cassinii*	4.5	2	Finches & sparrows
Passeriformes	Fringillidae	*Carpodacus mexicanus*	4.5	2,11	Finches & sparrows
Passeriformes	Fringillidae	*Carpodacus synoicus*	4.5	3,4,7	Finches & sparrows; EMN
Passeriformes	Fringillidae	*Carpodacus rubicilla*	4.5	3,4,7	Finches & sparrows; EMN
Passeriformes	Fringillidae	*Pinicola enucleator*	3.5	2,3,7	Finches & sparrows; EMN
Passeriformes	Fringillidae	*Loxia pytyopsittacus*	3.5	3	Finches & sparrows; EMN
Passeriformes	Fringillidae	*Loxia curvirostra*	3.5	2,3,4,5,7	Finches & sparrows; EMN
Passeriformes	Fringillidae	*Loxia leucoptera*	4.0	2,7	Finches & sparrows; EMN
Passeriformes	Fringillidae	*Pyrrhula nipalensis*	2.0	7	Finches & sparrows; Birds of Southeast Asia
Passeriformes	Fringillidae	*Pyrrhula pyrrhula*	4.5	3,4,7	Finches & sparrows; EMN
Passeriformes	Fringillidae	*Coccothraustes coccothraustes*	4.5	3,4,5,7	Finches & sparrows; EMN
Passeriformes	Fringillidae	*Hesperiphona vespertina*	3.5	2	Finches & sparrows
Passeriformes	Fringillidae	*Emberiza citrinella*	3.9	3,4,7,12,13	Sparrows and buntings
Passeriformes	Fringillidae	*Emberiza leucocephalos*	4.5	4,7	Sparrows and buntings
Passeriformes	Fringillidae	*Emberiza stewarti*	3.9	4,7	Sparrows and buntings
Passeriformes	Fringillidae	*Emberiza cirlus*	3.2	3,4,5,12,13	Sparrows and buntings
Passeriformes	Fringillidae	*Emberiza cia*	3.9	3,4,5,7	Sparrows and buntings
Passeriformes	Fringillidae	*Emberiza godlewskii*	3.9	7	Sparrows and buntings
Passeriformes	Fringillidae	*Emberiza cioides*	4.2	7	Sparrows and buntings
Passeriformes	Fringillidae	*Emberiza jankowskii*	4.5	7	Sparrows and buntings
Passeriformes	Fringillidae	*Emberiza buchanani*	4.5	3,4,7	Sparrows and buntings
Passeriformes	Fringillidae	*Emberiza cineracea*	4.9	3,4	Sparrows and buntings
Passeriformes	Fringillidae	*Emberiza hortulana*	4.5	3,4,5,7	Sparrows and buntings
Passeriformes	Fringillidae	*Emberiza caesia*	4.5	3,4	Sparrows and buntings
Passeriformes	Fringillidae	*Emberiza striolata*	2.8	3,4,5,7	Sparrows and buntings
Passeriformes	Fringillidae	*Emberiza impetuani*	3.5	5	Sparrows and buntings
Passeriformes	Fringillidae	*Emberiza tahapisi*	2.8	4,5	Sparrows and buntings
Passeriformes	Fringillidae	*Emberiza capensis*	2.8	5	Sparrows and buntings
Passeriformes	Fringillidae	*Emberiza tristrami*	4.5	7	Sparrows and buntings
Passeriformes	Fringillidae	*Emberiza fucata*	3.9	7	Sparrows and buntings
Passeriformes	Fringillidae	*Emberiza pusilla*	4.9	3,4,7	Sparrows and buntings
Passeriformes	Fringillidae	*Emberiza chrysophrys*	3.9	7	Sparrows and buntings
Passeriformes	Fringillidae	*Emberiza rustica*	4.9	3,4,7	Sparrows and buntings
Passeriformes	Fringillidae	*Emberiza elegans*	4.9	7	Sparrows and buntings
Passeriformes	Fringillidae	*Emberiza aureola*	3.9	3,4,7	Sparrows and buntings
Passeriformes	Fringillidae	*Emberiza rutila*	4.0	7	Sparrows and buntings
Passeriformes	Fringillidae	*Emberiza flaviventris*	2.4	5	Sparrows and buntings
Passeriformes	Fringillidae	*Emberiza poliopleura*	2.4	5	Sparrows and buntings
Passeriformes	Fringillidae	*Emberiza cabanisi*	2.4	5	Sparrows and buntings
Passeriformes	Fringillidae	*Emberiza melanocephala*	3.9	3,4	Sparrows and buntings
Passeriformes	Fringillidae	*Emberiza bruniceps*	4.5	4,7	Sparrows and buntings
Passeriformes	Fringillidae	*Emberiza sulphurata*	3.9	7	Sparrows and buntings
Passeriformes	Fringillidae	*Emberiza spodocephala*	4.5	7	Sparrows and buntings
Passeriformes	Fringillidae	*Emberiza variabilis*	3.9	7	Sparrows and buntings
Passeriformes	Fringillidae	*Emberiza pallasi*	3.9	7	Sparrows and buntings
Passeriformes	Fringillidae	*Emberiza schoeniclus*	4.5	3,4,5,7	Sparrows and buntings
Passeriformes	Fringillidae	*Emberiza yessoensis*	3.9	7	Sparrows and buntings
Passeriformes	Fringillidae	*Emberiza calandra*	4.2	3,4,5,7	Sparrows and buntings
Passeriformes	Fringillidae	*Calcarius mccownii*	3.5	2	Sparrows and buntings
Passeriformes	Fringillidae	*Calcarius lapponicus*	5.5	2,3,4,7	Sparrows and buntings
Passeriformes	Fringillidae	*Calcarius pictus*	4.0	2	Sparrows and buntings
Passeriformes	Fringillidae	*Calcarius ornatus*	4.9	2	Sparrows and buntings
Passeriformes	Fringillidae	*Plectrophenax nivalis*	4.9	2,3,4,7	Sparrows and buntings
Passeriformes	Fringillidae	*Plectrophenax hyperboreus*	4.9	2	Sparrows and buntings
Passeriformes	Fringillidae	*Calamospiza melanocorys*	4.9	2	Sparrows and buntings

Order	Family	*Species*	Clutch	Range	Traits
Passeriformes	Fringillidae	*Passerella iliaca*	3.9	2	Sparrows and buntings
Passeriformes	Fringillidae	*Melospiza melodia*	3.9	2	Sparrows and buntings
Passeriformes	Fringillidae	*Melospiza lincolnii*	4.5	2	Sparrows and buntings
Passeriformes	Fringillidae	*Melospiza georgiana*	4.5	2	Sparrows and buntings
Passeriformes	Fringillidae	*Zonotrichia capensis*	2.8	2	Sparrows and buntings
Passeriformes	Fringillidae	*Zonotrichia querula*	3.9	2	Sparrows and buntings
Passeriformes	Fringillidae	*Zonotrichia albicollis*	3.9	2	Sparrows and buntings
Passeriformes	Fringillidae	*Zonotrichia leucophrys*	3.9	2	Sparrows and buntings
Passeriformes	Fringillidae	*Zonotrichia atricapilla*	3.9	2	Sparrows and buntings
Passeriformes	Fringillidae	*Junco vulcani*	2.0	2	Sparrows and buntings
Passeriformes	Fringillidae	*Junco hyemalis*	4.5	2	Sparrows and buntings
Passeriformes	Fringillidae	*Junco phaeonotus*	3.5	2	Sparrows and buntings
Passeriformes	Fringillidae	*Passerculus sandwichensis*	4.5	2	Sparrows and buntings
Passeriformes	Fringillidae	*Ammodramus maritimus*	4.5	2	Sparrows and buntings
Passeriformes	Fringillidae	*Ammodramus nelsoni*	3.9	2	Sparrows and buntings
Passeriformes	Fringillidae	*Ammodramus caudacutus*	3.9	2	Sparrows and buntings
Passeriformes	Fringillidae	*Ammodramus leconteii*	3.9	2	Sparrows and buntings
Passeriformes	Fringillidae	*Ammodramus henslowii*	3.9	2	Sparrows and buntings
Passeriformes	Fringillidae	*Ammodramus bairdii*	4.2	2	Sparrows and buntings
Passeriformes	Fringillidae	*Ammodramus savannarum*	4.5	2	Sparrows and buntings
Passeriformes	Fringillidae	*Ammodramus humeralis*	3.5	2	Sparrows and buntings
Passeriformes	Fringillidae	*Ammodramus aurifrons*	2.4	2	Sparrows and buntings
Passeriformes	Fringillidae	*Spizella arborea*	5.0	2	Sparrows and buntings
Passeriformes	Fringillidae	*Spizella passerina*	4.0	2	Sparrows and buntings
Passeriformes	Fringillidae	*Spizella pallida*	3.9	2	Sparrows and buntings
Passeriformes	Fringillidae	*Spizella breweri*	3.5	2	Sparrows and buntings
Passeriformes	Fringillidae	*Spizella pusilla*	4.2	2	Sparrows and buntings
Passeriformes	Fringillidae	*Spizella wortheni*	3.5	2	Sparrows and buntings
Passeriformes	Fringillidae	*Spizella atrogularis*	2.8	2	Sparrows and buntings
Passeriformes	Fringillidae	*Pooecetes gramineus*	3.9	2	Sparrows and buntings
Passeriformes	Fringillidae	*Chondestes grammacus*	4.5	2	Sparrows and buntings
Passeriformes	Fringillidae	*Amphispiza bilineata*	3.5	2	Sparrows and buntings
Passeriformes	Fringillidae	*Amphispiza belli*	3.5	2	Sparrows and buntings
Passeriformes	Fringillidae	*Aimophila quinquestriata*	3.9	2	Sparrows and buntings
Passeriformes	Fringillidae	*Aimophila mystacalis*	3.5	2	Sparrows and buntings
Passeriformes	Fringillidae	*Aimophila humeralis*	3.5	2	Sparrows and buntings
Passeriformes	Fringillidae	*Aimophila ruficauda*	2.8	2	Sparrows and buntings
Passeriformes	Fringillidae	*Aimophila strigiceps*	3.5	2	Sparrows and buntings
Passeriformes	Fringillidae	*Aimophila stolzmanni*	4.0	2	Sparrows and buntings
Passeriformes	Fringillidae	*Aimophila aestivalis*	3.9	2	Sparrows and buntings
Passeriformes	Fringillidae	*Aimophila botterii*	3.2	2	Sparrows and buntings
Passeriformes	Fringillidae	*Aimophila cassinii*	4.0	2	Sparrows and buntings
Passeriformes	Fringillidae	*Aimophila carpalis*	4.0	2	Sparrows and buntings
Passeriformes	Fringillidae	*Aimophila ruficeps*	4.0	2	Sparrows and buntings
Passeriformes	Fringillidae	*Aimophila rufescens*	2.4	2	Sparrows and buntings
Passeriformes	Fringillidae	*Torreornis inexpectata*	1.4	2	Sparrows and buntings, Birds of the West Indies
Passeriformes	Fringillidae	*Oriturus superciliosus*	3.5	2	Sparrows and buntings
Passeriformes	Fringillidae	*Pipilo chlorurus*	4.0	2	Sparrows and buntings
Passeriformes	Fringillidae	*Pipilo ocai*	4.0	2	Sparrows and buntings
Passeriformes	Fringillidae	*Pipilo maculatus*	3.5	2	Sparrows and buntings
Passeriformes	Fringillidae	*Pipilo erythrophthalmus*	3.5	2	Sparrows and buntings
Passeriformes	Fringillidae	*Pipilo albicollis*	4.0	2	Sparrows and buntings
Passeriformes	Fringillidae	*Pipilo fuscus*	3.5	2	Sparrows and buntings
Passeriformes	Fringillidae	*Pipilo crissalis*	3.5	2	Sparrows and buntings
Passeriformes	Fringillidae	*Pipilo aberti*	3.5	2	Sparrows and buntings
Passeriformes	Fringillidae	*Arremon aurantiirostris*	2.0	2	BSA, Birds of Costa Rica
Passeriformes	Fringillidae	*Atlapetes gutturalis*	2.4	2	BSA, Birds of Costa Rica
Passeriformes	Fringillidae	*Atlapetes brunneinucha*	2.0	2	Birds of Venezuela, Birds of Costa Rica
Passeriformes	Fringillidae	*Atlapetes atricapillus*	2.0	2	BSA, Birds of Costa Rica
Passeriformes	Fringillidae	*Vermivora bachmanii*	3.5	2	Warblers of the Americas
Passeriformes	Fringillidae	*Vermivora pinus*	5.5	2	Warblers of the Americas
Passeriformes	Fringillidae	*Vermivora chrysoptera*	4.5	2	Warblers of the Americas
Passeriformes	Fringillidae	*Vermivora peregrina*	6.0	2	Warblers of the Americas

Order	Family	*Species*	Clutch	Range	Traits
Passeriformes	Fringillidae	*Vermivora celata*	4.5	2	Warblers of the Americas
Passeriformes	Fringillidae	*Vermivora ruficapilla*	4.5	2	Warblers of the Americas
Passeriformes	Fringillidae	*Vermivora virginiae*	4.0	2	Warblers of the Americas
Passeriformes	Fringillidae	*Vermivora crissalis*	4.0	2	Warblers of the Americas
Passeriformes	Fringillidae	*Vermivora luciae*	4.5	2	Warblers of the Americas
Passeriformes	Fringillidae	*Parula americana*	4.5	2	Warblers of the Americas
Passeriformes	Fringillidae	*Parula pitiayumi*	2.8	2	Warblers of the Americas
Passeriformes	Fringillidae	*Parula superciliosa*	2.4	2	Warblers of the Americas
Passeriformes	Fringillidae	*Parula gutturalis*	2.0	2	Warblers of the Americas
Passeriformes	Fringillidae	*Dendroica petechia*	4.5	2	Warblers of the Americas
Passeriformes	Fringillidae	*Dendroica pensylvanica*	4.0	2	Warblers of the Americas
Passeriformes	Fringillidae	*Dendroica magnolia*	4.0	2	Warblers of the Americas
Passeriformes	Fringillidae	*Dendroica tigrina*	6.5	2	Warblers of the Americas
Passeriformes	Fringillidae	*Dendroica caerulescens*	4.0	2	Warblers of the Americas
Passeriformes	Fringillidae	*Dendroica coronata*	4.0	2	Warblers of the Americas
Passeriformes	Fringillidae	*Dendroica nigrescens*	4.0	2	Warblers of the Americas
Passeriformes	Fringillidae	*Dendroica townsendi*	3.9	2	Warblers of the Americas
Passeriformes	Fringillidae	*Dendroica occidentalis*	4.0	2	Warblers of the Americas
Passeriformes	Fringillidae	*Dendroica virens*	4.0	2	Warblers of the Americas
Passeriformes	Fringillidae	*Dendroica chrysoparia*	4.0	2	Warblers of the Americas
Passeriformes	Fringillidae	*Dendroica fusca*	4.0	2	Warblers of the Americas
Passeriformes	Fringillidae	*Dendroica dominica*	4.0	2	Warblers of the Americas
Passeriformes	Fringillidae	*Dendroica graciae*	3.0	2	Warblers of the Americas
Passeriformes	Fringillidae	*Dendroica adelaidae*	2.4	2	Warblers of the Americas
Passeriformes	Fringillidae	*Dendroica pityophila*	2.0	2	Warblers of the Americas
Passeriformes	Fringillidae	*Dendroica pinus*	4.0	2	Warblers of the Americas
Passeriformes	Fringillidae	*Dendroica kirtlandii*	4.5	2	Warblers of the Americas
Passeriformes	Fringillidae	*Dendroica discolor*	4.0	2	Warblers of the Americas
Passeriformes	Fringillidae	*Dendroica palmarum*	4.5	2	Warblers of the Americas
Passeriformes	Fringillidae	*Dendroica castanea*	4.5	2	Warblers of the Americas
Passeriformes	Fringillidae	*Dendroica striata*	4.5	2	Warblers of the Americas
Passeriformes	Fringillidae	*Dendroica cerulea*	4.0	2	Warblers of the Americas
Passeriformes	Fringillidae	*Dendroica pharetra*	2.8	2	Warblers of the Americas
Passeriformes	Fringillidae	*Dendroica angelae*	2.0	2	Warblers of the Americas
Passeriformes	Fringillidae	*Mniotilta varia*	4.5	2	Warblers of the Americas
Passeriformes	Fringillidae	*Setophaga ruticilla*	4.0	2	Warblers of the Americas
Passeriformes	Fringillidae	*Protonotaria citrea*	4.9	2	Warblers of the Americas
Passeriformes	Fringillidae	*Helmitheros vermivorus*	4.5	2	Warblers of the Americas
Passeriformes	Fringillidae	*Limnothlypis swainsonii*	3.0	2	Warblers of the Americas
Passeriformes	Fringillidae	*Seiurus aurocapillus*	4.5	2	Warblers of the Americas
Passeriformes	Fringillidae	*Seiurus noveboracensis*	4.5	2	Warblers of the Americas
Passeriformes	Fringillidae	*Seiurus motacilla*	5.0	2	Warblers of the Americas
Passeriformes	Fringillidae	*Oporornis formosus*	4.5	2	Warblers of the Americas
Passeriformes	Fringillidae	*Oporornis agilis*	4.2	2	Warblers of the Americas
Passeriformes	Fringillidae	*Oporornis philadelphia*	3.9	2	Warblers of the Americas
Passeriformes	Fringillidae	*Oporornis tolmiei*	4.2	2	Warblers of the Americas
Passeriformes	Fringillidae	*Geothlypis trichas*	3.9	2	Warblers of the Americas
Passeriformes	Fringillidae	*Geothlypis beldingi*	2.8	2	Warblers of the Americas
Passeriformes	Fringillidae	*Geothlypis rostrata*	2.0	2	Warblers of the Americas
Passeriformes	Fringillidae	*Geothlypis semiflava*	1.4	2	Warblers of the Americas
Passeriformes	Fringillidae	*Geothlypis aequinoctialis*	2.0	2	Warblers of the Americas
Passeriformes	Fringillidae	*Geothlypis chiriquensis*	2.0	2	Warblers of the Americas
Passeriformes	Fringillidae	*Geothlypis auricularis*	2.0	2	Warblers of the Americas
Passeriformes	Fringillidae	*Geothlypis velata*	2.0	2	Warblers of the Americas
Passeriformes	Fringillidae	*Geothlypis poliocephala*	2.8	2	Warblers of the Americas
Passeriformes	Fringillidae	*Microligea palustris*	2.0	2	Warblers of the Americas
Passeriformes	Fringillidae	*Teretistris fernandinae*	2.4	2	Warblers of the Americas
Passeriformes	Fringillidae	*Teretistris fornsi*	2.4	2	Warblers of the Americas, Birds of the West Indies
Passeriformes	Fringillidae	*Wilsonia citrina*	3.9	2	Warblers of the Americas
Passeriformes	Fringillidae	*Wilsonia pusilla*	4.9	2	Warblers of the Americas
Passeriformes	Fringillidae	*Wilsonia canadensis*	3.9	2	Warblers of the Americas
Passeriformes	Fringillidae	*Cardellina rubrifrons*	3.5	2	Warblers of the Americas
Passeriformes	Fringillidae	*Myioborus pictus*	4.6	2	Warblers of the Americas

Order	Family	*Species*	Clutch	Range	Traits
Passeriformes	Fringillidae	*Myioborus miniatus*	1.7	2	Warblers of the Americas
Passeriformes	Fringillidae	*Myioborus torquatus*	2.4	2	Warblers of the Americas
Passeriformes	Fringillidae	*Myioborus melanocephalus*	2.0	2	Warblers of the Americas
Passeriformes	Fringillidae	*Myioborus flavivertex*	2.0	2	Warblers of the Americas
Passeriformes	Fringillidae	*Basileuterus conspicillatus*	3.5	2	Warblers of the Americas
Passeriformes	Fringillidae	*Basileuterus culicivorus*	2.8	2	Warblers of the Americas
Passeriformes	Fringillidae	*Basileuterus rufifrons*	2.4	2	Warblers of the Americas
Passeriformes	Fringillidae	*Basileuterus melanogenys*	2.0	2	Warblers of the Americas
Passeriformes	Fringillidae	*Basileuterus tristriatus*	2.0	2	Warblers of the Americas
Passeriformes	Fringillidae	*Basileuterus flaveolus*	3.0	2	Warblers of the Americas
Passeriformes	Fringillidae	*Basileuterus fulvicauda*	2.0	2	Warblers of the Americas
Passeriformes	Fringillidae	*Icteria virens*	3.9	2	Warblers of the Americas
Passeriformes	Fringillidae	*Orchesticus abeillei*	2.0	2	Tanagers
Passeriformes	Fringillidae	*Nemosia pileata*	2.0	2	Tanagers
Passeriformes	Fringillidae	*Phaenicophilus palmarum*	2.4	2	Tanagers
Passeriformes	Fringillidae	*Phaenicophilus poliocephalus*	2.8	2	Tanagers
Passeriformes	Fringillidae	*Mitrospingus cassinii*	2.0	2	Tanagers
Passeriformes	Fringillidae	*Chlorothraupis carmioli*	2.0	2	Tanagers
Passeriformes	Fringillidae	*Chlorothraupis frenata*	2.0	2	Tanagers
Passeriformes	Fringillidae	*Chlorothraupis stolzmanni*	1.4	2	Tanagers
Passeriformes	Fringillidae	*Eucometis penicillata*	2.0	2	Tanagers
Passeriformes	Fringillidae	*Lanio leucothorax*	2.0	2	Tanagers
Passeriformes	Fringillidae	*Trichothraupis melanops*	3.0	2	Tanagers
Passeriformes	Fringillidae	*Piranga bidentata*	2.4	2	Tanagers
Passeriformes	Fringillidae	*Piranga flava*	2.8	2	Tanagers
Passeriformes	Fringillidae	*Piranga rubra*	3.5	2	Tanagers
Passeriformes	Fringillidae	*Piranga olivacea*	3.5	2	Tanagers
Passeriformes	Fringillidae	*Piranga ludoviciana*	3.9	2	Tanagers
Passeriformes	Fringillidae	*Anisognathus igniventris*	2.0	2	Tanagers
Passeriformes	Fringillidae	*Stephanophorus diadematus*	4.0	2	Tanagers
Passeriformes	Fringillidae	*Euphonia jamaica*	3.5	2	Tanagers
Passeriformes	Fringillidae	*Euphonia affinis*	3.0	2	Tanagers
Passeriformes	Fringillidae	*Euphonia luteicapilla*	3.0	2	Tanagers
Passeriformes	Fringillidae	*Euphonia chlorotica*	3.9	2	Tanagers
Passeriformes	Fringillidae	*Euphonia trinitatis*	3.5	2	Tanagers
Passeriformes	Fringillidae	*Euphonia violacea*	4.0	2	Tanagers
Passeriformes	Fringillidae	*Euphonia laniirostris*	4.0	2	Tanagers
Passeriformes	Fringillidae	*Euphonia hirundinacea*	5.0	2	Tanagers
Passeriformes	Fringillidae	*Euphonia elegantissima*	3.0	2	Tanagers, Birds of Costa Rica
Passeriformes	Fringillidae	*Euphonia musica*	4.0	2	Tanagers
Passeriformes	Fringillidae	*Euphonia cyanocephala*	2.4	2	Tanagers
Passeriformes	Fringillidae	*Euphonia imitans*	3.0	2	Tanagers
Passeriformes	Fringillidae	*Euphonia gouldi*	2.8	2	Tanagers
Passeriformes	Fringillidae	*Euphonia minuta*	3.0	2	Tanagers
Passeriformes	Fringillidae	*Euphonia xanthogaster*	4.0	2	Tanagers
Passeriformes	Fringillidae	*Euphonia cayennensis*	3.9	2	Tanagers
Passeriformes	Fringillidae	*Euphonia pectoralis*	3.0	2	Tanagers
Passeriformes	Fringillidae	*Tangara inornata*	2.0	2	Tanagers
Passeriformes	Fringillidae	*Tangara mexicana*	2.4	2	Tanagers
Passeriformes	Fringillidae	*Tangara chilensis*	2.0	2	Tanagers
Passeriformes	Fringillidae	*Tangara seledon*	3.0	2	Tanagers
Passeriformes	Fringillidae	*Tangara cyanocephala*	3.0	2	Tanagers
Passeriformes	Fringillidae	*Tangara desmaresti*	3.0	2	Tanagers
Passeriformes	Fringillidae	*Tangara schrankii*	2.0	2	Tanagers
Passeriformes	Fringillidae	*Tangara florida*	2.0	2	Tanagers
Passeriformes	Fringillidae	*Tangara arthus*	1.4	2	Tanagers
Passeriformes	Fringillidae	*Tangara icterocephala*	2.0	2	Tanagers
Passeriformes	Fringillidae	*Tangara xanthocephala*	2.0	2	Tanagers
Passeriformes	Fringillidae	*Tangara guttata*	2.0	2	Tanagers
Passeriformes	Fringillidae	*Tangara gyrola*	2.0	2	Tanagers
Passeriformes	Fringillidae	*Tangara lavinia*	3.0	2	Tanagers, Birds of Costa Rica
Passeriformes	Fringillidae	*Tangara cayana*	2.0	2	Tanagers
Passeriformes	Fringillidae	*Tangara vitriolina*	2.0	2	Tanagers, BHA

Order	Family	Species	Clutch	Range	Traits
Passeriformes	Fringillidae	*Tangara ruficervix*	2.0	2	Tanagers
Passeriformes	Fringillidae	*Tangara cyanicollis*	2.0	2	Tanagers
Passeriformes	Fringillidae	*Tangara larvata*	2.0	2	Tanagers
Passeriformes	Fringillidae	*Tangara nigrocincta*	2.0	2	Tanagers
Passeriformes	Fringillidae	*Tangara dowii*	2.0	2	Tanagers, Birds of Costa Rica
Passeriformes	Fringillidae	*Iridophanes pulcherrima*	2.0	2	Tanagers
Passeriformes	Fringillidae	*Emberizoides herbicola*	2.0	2	Birds of Venezuela, Birds of Costa Rica
Passeriformes	Fringillidae	*Sporophila torqueola*	2.4	2	Birds of Costa Rica
Passeriformes	Fringillidae	*Sporophila nigricollis*	2.4	2	Birds of Venezuela, Birds of Costa Rica
Passeriformes	Fringillidae	*Sporophila minuta*	2.4	2	BSA, Birds of Costa Rica
Passeriformes	Fringillidae	*Amaurospiza concolor*	2.0	2	BSA, Birds of Costa Rica
Passeriformes	Fringillidae	*Melopyrrha nigra*	3.5	2	Birds of the West Indies
Passeriformes	Fringillidae	*Spiza americana*	3.5	2	Birds of Venezuela
Passeriformes	Fringillidae	*Pheucticus tibialis*	2.0	2	Birds of Costa Rica
Passeriformes	Fringillidae	*Pheucticus ludovicianus*	3.9	2	Birds of Venezuela; BSA, Birds of Costa Rica
Passeriformes	Fringillidae	*Pheucticus melanocephalus*	3.5	2	Birds of Costa Rica
Passeriformes	Fringillidae	*Cardinalis cardinalis*	3.5	2,11,13	BMx
Passeriformes	Fringillidae	*Cardinalis sinuatus*	2.4	2	BMx
Passeriformes	Fringillidae	*Guiraca caerulea*	3.5	2	Birds of Costa Rica
Passeriformes	Fringillidae	*Passerina amoena*	3.9	2	BMx
Passeriformes	Fringillidae	*Passerina cyanea*	3.5	2	Birds of Venezuela, Birds of Costa Rica
Passeriformes	Fringillidae	*Passerina versicolor*	3.5	2	BMx
Passeriformes	Fringillidae	*Passerina ciris*	3.5	2	Birds of Costa Rica
Passeriformes	Fringillidae	*Icterus chrysocephalus*	2.0	2	New World Blackbirds
Passeriformes	Fringillidae	*Icterus cayanensis*	2.4	2	New World Blackbirds
Passeriformes	Fringillidae	*Icterus graduacauda*	3.9	2	New World Blackbirds
Passeriformes	Fringillidae	*Icterus chrysater*	2.4	2	New World Blackbirds
Passeriformes	Fringillidae	*Icterus nigrogularis*	3.0	2	New World Blackbirds
Passeriformes	Fringillidae	*Icterus leucopteryx*	3.9	2	New World Blackbirds
Passeriformes	Fringillidae	*Icterus mesomelas*	2.4	2	New World Blackbirds
Passeriformes	Fringillidae	*Icterus graceannae*	2.0	2	New World Blackbirds
Passeriformes	Fringillidae	*Icterus pectoralis*	3.0	2	New World Blackbirds
Passeriformes	Fringillidae	*Icterus gularis*	3.5	2	New World Blackbirds
Passeriformes	Fringillidae	*Icterus pustulatus*	3.5	2	New World Blackbirds
Passeriformes	Fringillidae	*Icterus icterus*	3.0	2	New World Blackbirds
Passeriformes	Fringillidae	*Icterus galbula*	4.5	2	New World Blackbirds
Passeriformes	Fringillidae	*Icterus bullockii*	4.5	2	New World Blackbirds
Passeriformes	Fringillidae	*Icterus cucullatus*	3.9	2	New World Blackbirds
Passeriformes	Fringillidae	*Icterus spurius*	4.0	2	New World Blackbirds
Passeriformes	Fringillidae	*Icterus wagleri*	3.0	2	New World Blackbirds
Passeriformes	Fringillidae	*Icterus dominicensis*	3.0	2	New World Blackbirds
Passeriformes	Fringillidae	*Icterus parisorum*	2.8	2	New World Blackbirds
Passeriformes	Fringillidae	*Xanthocephalus xanthocephalu*	3.5	2	New World Blackbirds
Passeriformes	Fringillidae	*Agelaius flavus*	4.0	2	New World Blackbirds
Passeriformes	Fringillidae	*Agelaius thilius*	3.0	2	New World Blackbirds, BHA
Passeriformes	Fringillidae	*Agelaius cyanopus*	3.0	2	New World Blackbirds
Passeriformes	Fringillidae	*Agelaius phoeniceus*	3.0	2	New World Blackbirds
Passeriformes	Fringillidae	*Agelaius assimilis*	2.4	2	New World Blackbirds
Passeriformes	Fringillidae	*Agelaius tricolor*	4.0	2	New World Blackbirds
Passeriformes	Fringillidae	*Agelaius icterocephalus*	2.4	2	New World Blackbirds
Passeriformes	Fringillidae	*Agelaius humeralis*	3.5	2	New World Blackbirds, Birds of the West Indies
Passeriformes	Fringillidae	*Agelaius xanthomus*	3.0	2	New World Blackbirds
Passeriformes	Fringillidae	*Agelaius ruficapillus*	3.0	2	New World Blackbirds
Passeriformes	Fringillidae	*Sturnella bellicosa*	3.9	2	New World Blackbirds
Passeriformes	Fringillidae	*Sturnella militaris*	2.8	2	New World Blackbirds; Birds of Venezuela
Passeriformes	Fringillidae	*Sturnella loyca*	4.0	2	New World Blackbirds, BHA
Passeriformes	Fringillidae	*Sturnella magna*	5.0	2	New World Blackbirds, Birds of Costa Rica
Passeriformes	Fringillidae	*Sturnella neglecta*	4.2	2,11	New World Blackbirds
Passeriformes	Fringillidae	*Hypopyrrhus pyrohypogaster*	2.0	2	New World Blackbirds
Passeriformes	Fringillidae	*Oreopsar bolivianus*	3.0	14	New World Blackbirds
Passeriformes	Fringillidae	*Quiscalus mexicanus*	3.5	2	New World Blackbirds
Passeriformes	Fringillidae	*Quiscalus major*	3.5	2	New World Blackbirds
Passeriformes	Fringillidae	*Quiscalus nicaraguensis*	2.4	2	New World Blackbirds

Order	Family	*Species*	Clutch	Range	Traits
Passeriformes	Fringillidae	*Quiscalus quiscula*	4.2	2	New World Blackbirds
Passeriformes	Fringillidae	*Quiscalus niger*	3.5	2	New World Blackbirds, Birds of the West Indies
Passeriformes	Fringillidae	*Quiscalus lugubris*	2.8	2	New World Blackbirds
Passeriformes	Fringillidae	*Euphagus carolinus*	4.5	2	New World Blackbirds
Passeriformes	Fringillidae	*Euphagus cyanocephalus*	5.0	2	New World Blackbirds
Passeriformes	Fringillidae	*Dolichonyx oryzivorus*	5.0	2	New World Blackbirds
