# Supplementary material for: The Worldwide Variation in Avian Clutch Size across Species and Space
Source: PLoS Biol. 2008 Dec 9;6(12):e303. doi: 10.1371/journal.pbio.0060303 (PMC2596859; doi:10.1371/journal.pbio.0060303)
Supplement: Supplementary file 6 [file pbio.0060303.st005.pdf]

Table S5

List of main literature sources consulted, with abbreviations. Ref. refers to the numbered citation listed in Table S6.

Abbreviation	Ref. ID	Title	Authors	Volumes
HBW	62-70	Handbook of the Birds of the World	del Hoyo, J., Elliott, A. & Sargatal, J., eds.	Multiple
BA	89-91,131,227,228	Birds of Africa	Fry	Multiple
ANZAB	154	Handbook of Australian, New Zealand & Antarctic Birds	Marchant, S., Higgins, P. J. & Ambrose, S. J. (1990)	Multiple
BNA	226	Birds of North America Online	American Ornithologists' Union & Academy of Natural Sciences of Philadelphia	Multiple
BIP	2-7	Handbook of the Birds of India and Pakistan	Ali, S. & Ripley, S. D.	Multiple
EMN	49-54	Handbook of the Birds of Europe, the Middle East and North Africa	Cramp	Multiple
BPNG	41	The Birds of Papua New Guinea	Coates	
Swallows and Martins	225	A Handbook to the Swallows and Martins of the World	Turner & Rose	
BBSJB	150	Birds of Borneo, Sumatra, Java, and Bali	MacKinnon, J. R. & Phillipps	
BCh	149	A Field Guide to the Birds of China	MacKinnon, J. R. & Phillipps	
BGA	33	A guide to the birds of the Galápagos Islands	Castro, Isabel C.	
BH	82	The Birder's Handbook: A Field Guide to the Natural History of North American Birds	Ehrlich, P. R., Dobkin, D. S. & Wheye, D. (1988)	
BMx	116	A Guide to the Birds of Mexico and Northern Central America	Howell, S. N. G. & Webb, S. (1995)	
BHA	86	Birds of the High Andes	Fjeldså, J. & Krabbe, N. (1990)	
BHTP	170	The Birds of Hawaii and the Tropical Pacific	Pratt, H. D., Bruner, P. L. & Berrett, G. D. (1987)	NBB
BIOI	211	Birds of the Indian Ocean Islands	Sinclair, J. C. & Langrand, O. (1998)	BSI
	189	Guide to the Birds of Southeast Asia	Robson, C. A. (2000)	EBW
	132	A Guide to the Birds of the Philippines	Kennedy, R. S. (2000)	
BWAL	42	A Guide to the Birds of Wallacea	Coates, B. J., Bishop, K. D. & Gardner, D. (1997)	
	217	A Guide to the Birds of Costa Rica	Stiles, F. G. & Skutch, A. F. (1989)	
	25	Birds of the West Indies	Raffaele et al. (1998)	
	88	The Birds of Paradise	Frith, C. B., Beehler, B. M. & Cooper, W. T. (1998)	
	114	Birds of Venezuela	Hilty, S. L. & De Schauensee, R. M. (2003)	
BSA	184-185	The Birds of South America	Ridgely, R. S. & Tudor, G.	Multiple
BIS	112	Birds of the Indian Subcontinent	Grimmett, R., Inskipp, C. & Inskipp, T. (1998)	
BJAP	27	The Birds of Japan	Brazil, M. & Yabuuchi, M. (1991)	
BNZ	110	The Field Guide to the Birds of New Zealand	Heather, B. D. & Robertson, H. A. (1997)	
	1	The Book of Indian Birds	Ali, S. (1996)	
BP	183	A Guide to the Birds of Panama	Ridgely, R. S. & Gwynne, J. A. (1989)	
BSWP	157	Birds of the Southwest Pacific	Mayr, E. & Jaques, F. L. (1978)	
CBAB	200	Reader's Digest Complete Book of Australian Birds	Schodde, R., Tidemann, S. C. & Bell, H. L. (1986)	
	104	Chickadees, Tits, Nuthatches & Treecreepers	Harrap, S. & Quinn, D. (1995)	
	152	Crows and Jays: A Guide to the Crows, Jays, and Magpies of the World	Madge, S. & Burn, H. (1994)	
EBA	214	Endemic Bird Areas of the World	Stattersfield, A. J., Crosby, M. J., Long, A. J. & Wege, D. C. (1998)	
BWA	201	The Collins Field Guide to the Birds of West Africa	Serle, W. & Morel, G. J. (1988)	
	194	Fairy-wrens and Grasswrens: Maluridae	Rowley, I., Russell, E. M. & Marsack, P. (1997)	
	35	Finches and Sparrows: An Identification Guide	Clement, P., Harris, A. & Davis, J. (1993)	
	100	Estrildid Finches of the World	Goodwin, D. (1982) (Comstock, London)	
	141	Guide to the Birds of Madagascar	Langrand, O. (1990)	
HANZAB	154	Handbook of Australian, New Zealand & Antarctic Birds	Marchant, S., Higgins, P. J. & Ambrose, S. J. (1990)	
TBW	22	Threatened Birds of the World	BirdLife International (2000)	
	126	New World Blackbirds: The Icterids	Jaramillo, A. & Burke, P. (1999)	
PittasEtc	140	Pittas, Broadbills and Asities	Lambert, F. R. & Woodcock, M. (1996)	
	144	Shrikes: A Guide to the Shrikes of the World	Lefranc, N. & Worfolk, T. (1997)	
	32	Sparrows and Buntings	Byers, C., Curson, J. & Olsson, U. (1995)	
	84	Starlings and Mynas	Feare, C. & Craig, A. (1999)	
	34	Sunbirds	Cheke, R. A., Mann, C. F. & Allen, R. (2001)	
	203	Sylvia Warblers	Shirihai, H., Gargallo, G., Helbig, A. J., Harris, A., Kirwan, G. M., Svensson, L. (2001)	
	120	The Tanagers	Isler, M. L. & Isler, P. R. (1999)	
	47	Threatened Birds of Africa and Related Islands	Collar, N. J., Stuart, S. N., ICBP & IUCN (1985)	
	36	Thrushes	Clement, P. & Hathway, R. (2000)	
	18	Warblers of Europe, Asia, and North Africa	Baker, K. (1997)	
	57	Warblers of the Americas	Curson, J., Quinn, D. & Beadle, D. (1994)	
	29	Wrens, Dippers and Thrashers	Brewer, D. & Mackay, B. K. (2001)	
