# Supplementary material for: The Worldwide Variation in Avian Clutch Size across Species and Space
Source: PLoS Biol. 2008 Dec 9;6(12):e303. doi: 10.1371/journal.pbio.0060303 (PMC2596859; doi:10.1371/journal.pbio.0060303)
Supplement: Supplementary file 7 [file pbio.0060303.st006.doc]

Table S6

List of all literature sources consulted (with Ref. ID, linking to Table S5).

1. Ali, S. (1996) *The Book of Indian Birds* (Oxford Univ. Press, Bombay).

2. Ali, S. & Ripley, S. D. (1987) *Handbook of the Birds of India and Pakistan Together with Those of Bangladesh, Nepal, Bhutan, and Sri Lanka: Larks to the Grey Hypocolius* (Oxford Univ. Press, Delhi), Vol. 5.

3. Ali, S. & Ripley, S. D. (1996) *Handbook of the Birds of India and Pakistan Together with Those of Bangladesh, Nepal, Bhutan, and Sri Lanka: Cuckoo shrikes to Babaxes* (Oxford Univ. Press, Delhi), Vol. 6.

4. Ali, S. & Ripley, S. D. (1996) *Handbook of the Birds of India and Pakistan Together with Those of Bangladesh, Nepal, Bhutan, and Sri Lanka: Laughing thrushes to the Mangrove Whistler* (Oxford Univ. Press, Delhi), Vol. 7.

5. Ali, S. & Ripley, S. D. (1996) *Handbook of the Birds of India and Pakistan Together with Those of Bangladesh, Nepal, Bhutan, and Sri Lanka: Warblers to Redstarts* (Oxford Univ. Press, Delhi), Vol. 8.

6. Ali, S. & Ripley, S. D. (1998) *Handbook of the Birds of India and Pakistan Together with Those of Bangladesh, Nepal, Bhutan, and Sri Lanka: Robins to Wagtails* (Oxford Univ. Press, Delhi), Vol. 9.

7. Ali, S. & Ripley, S. D. (1999) *Handbook of the Birds of India and Pakistan Together with Those of Bangladesh, Nepal, Bhutan, and Sri Lanka: Flowerpeckers to Buntings* (Oxford Univ. Press, Delhi), Vol. 10.

8. Alonso, J. A. & Whitney, B. M. (2001) *Wilson Bull.* **113,** 1–9.

9. Alström, P. & Mild, K. (2003) *Pipits and Wagtails* (Princeton Univ. Press, Princeton).

10. Alström, P. & Olsson, U. (1995) *Ibis (Lond. 1859)* **137,** 459–468.

11. Alström, P. & Olsson, U. (1999) *Ibis (Lond. 1859)* **141,** 545–568.

12. Alström, P. & Olsson, U. (2000) *Ibis (Lond. 1859)* **142,** 495–500.

13. Alström, P., Olsson, U. & Colston, P. R. (1992) *Ibis (Lond. 1859)* **134,** 329–334.

14. Alström, P., Olsson, U. & Colston, P. R. (1995) *Bull. B.O.C.* **115,** 53–57.

15. Alström, P., Olsson, U. & Colston, P. R. (1997) *Bull. B.O.C.* **117,** 177–193.

16. Alström, P., Ripley, S. D. & Rasmussen, P. C. (1993) *Bull. B.O.C.* **113,** 207–209.

17. Alviola, P. L., III. (1997) *Asia Life Sciences* **6,** 51–55.

18. Baker, K. (1997) *Warblers of Europe, Asia, and North Africa* (Princeton Univ. Press, Princeton).

19. Beehler, B. M., Pratt, T. K. & Zimmerman, D. A. (1986) *Birds of New Guinea* (Princeton Univ. Press, Princeton).

20. Beresford, P. & Cracraft, J. (1999) *Am. Mus. Novit.* **3270,** 1–22.

21. Biaggi, V. (1997) *Las Aves de Puerto Rico* (Editorial de la Universidad de Puerto Rico, San Juan).

22. BirdLife International. (2000) *Threatened Birds of the World* (Lynx Edicions and BirdLife International, Barcelona and Cambridge, U.K.).

23. BirdLife International. (2001) *BirdLife's World Bird Database* (BirdLife International, Cambridge, U.K.), Version 1.0.

24. Blakers, M., Davies, S. J. J. F., Reilly, P. N. & Royal Australasian Ornithologists' Union. (1984) *The Atlas of Australian Birds* (Melbourne Univ. Press, Carlton, Australia).

25. Bond, J. (1985) *Birds of the West Indies* (Houghton-Mifflin, Boston).

26. Borrow, N. & Demey, R. (2001) *Birds of Western Africa* (Christopher Helm, London).

27. Brazil, M. & Yabuuchi, M. (1991) *The Birds of Japan* (Smithsonian Institution Press, Washington, D.C.).

28. Bretagnolle, V., Attie, C. & Mougeot, F. (2000) *Ibis (Lond. 1859)* **142,** 399–412.

29. Brewer, D. & Mackay, B. K. (2001) *Wrens, Dippers, and Thrashers* (Christopher Helm, London).

30. Brown, L., Urban, E. K. & Newman, K. B. (1982) *Birds of Africa: Ostriches to Birds of Prey* (Academic, London), Vol. 1.

31. Buckley, P. A., Foster, M. S., Morton, E. S., Ridgely R. S. & Buckley, F. G. (1985) *Neotropical Ornithology* (Amer. Ornithol. Union, Washington, D.C.).

32. Byers, C., Curson, J. & Olsson, U. (1995) *Sparrows and Buntings: A Guide to the Sparrows and Buntings of North America and the World* (Houghton-Mifflin, Boston).

33. Castro, I. C. & Phillips, A. (1996) *A Guide to the Birds of the Galápagos Islands* (Princeton Univ. Press, Princeton).

34. Cheke, R. A., Mann, C. F. & Allen, R. (2001) *Sunbirds: A Guide to the Sunbirds, Flowerpeckers, Spiderhunters, and Sugarbirds of the World* (Christopher Helm, London).

35. Clement, P., Harris, A. & Davis, J. (1993) *Finches and Sparrows: An Identification Guide* (Princeton Univ. Press, Princeton).

36. Clement, P. & Hathway, R. (2000) *Thrushes* (Christopher Helm, London).

37. Clements, J. F. (2000) *Birds of the World: A Checklist* (Ibis, Vista, CA).

38. Clements, J. F. & Santa Barbara Software Products. (2000) *Moving Your Life List from the Fourth to Fifth Edition of "Birds of the World: A Check List" by James Clements* (http://members.aol.com/sbsp/clements4thto5th.htm).

39. Clements, J. F. & Shany, N. (2001) *A Field Guide to the Birds of Peru* (Ibis, Temecula, CA).

40. Coates, B. J. (1985) *The Birds of Papua New Guinea: Nonpasserines* (Dove Publications, Alderley, Australia), Vol. 1.

41. Coates, B. J. (1990) *The Birds of Papua New Guinea: Passerines*, vol. 2 (Dove Publications, Brisbane, Australia).

42. Coates, B. J., Bishop, K. D. & Gardner, D. (1997) *A Guide to the Birds of Wallacea: Sulawesi, the Moluccas, and Lesser Sunda Islands, Indonesia* (Dove Publications, Alderley, Australia).

43. Collar, N. J., Andrew, P. & ICBP (1988) *Birds to Watch: The ICBP World Checklist of Threatened Birds* (Int. Counc. for Bird Pres., Cambridge, U.K.).

44. Collar, N. J., Crosby, M. J., Stattersfield, A. J., Andrew, P. & BirdLife International (1994) *Birds to Watch 2: The World List of Threatened Birds* (BirdLife International, Cambridge, U.K.).

45. Collar, N. J., Crosby, R., Crosby, M. J. & BirdLife International (2001) *Threatened Birds of Asia* (BirdLife International, Cambridge, U.K.).

46. Collar, N. J., International Union for Conservation of Nature and Natural Resources & International Council for Bird Preservation (1992) *Threatened Birds of the Americas* (Smithsonian Institution Press, Washington, DC).

47. Collar, N. J., Stuart, S. N., International Council for Bird Preservation & International Union for Conservation of Nature and Natural Resources (1985) *Threatened Birds of Africa and Related Islands* (Int. Counc. for Bird Pres., Cambridge, U.K.).

48. Coopmans, P. & Krabbe, N. (2000) *Wilson Bull.* **112,** 305–312.

49. Cramp, S. Ed. (1977) *Handbook of the Birds of Europe, the Middle East, and North Africa: The Birds of the Western Palearctic* (Oxford Univ. Press, Oxford).

50. Cramp, S., ed. (1977) *Handbook of the Birds of Europe, the Middle East and North Africa: Warblers*, vol. 6 (Oxford Univ. Press, Oxford).

51. Cramp, S., ed. (1977) *Handbook of the Birds of Europe, the Middle East and North Africa: Crows to Finches* (Oxford Univ. Press, Oxford), Vol. 8.

52. Cramp, S., ed. (1988) *Handbook of the Birds of Europe, the Middle East, and North Africa: Tyrant Flycatchers to Thrushes* (Oxford Univ. Press, Oxford), Vol. 5.

53. Cramp, S., ed. (1993) *Handbook of the Birds of Europe, the Middle East, and North Africa: Flycatchers to Shrikes* (Oxford Univ. Press, Oxford), Vol. 7.

54. Cramp, S., ed. (1994) *Handbook of the Birds of Europe, the Middle East and North Africa: Buntings and New World Warblers* (Oxford Univ. Press, Oxford), Vol. 9.

55. Cresswell, W., Hughes, M., Mellanby, R., Bright, S., Catry, P., Chaves, J., Freile, J., Gabela, A., Martineau, H., Macleod, R., *et al.*. (1999) *Bird Conserv. Int.* **9,** 129–145.

56. Cuervo, A. M., Salaman, P. G. W., Donegan, T. M & Ochoa, J. M. (2001) *Ibis (Lond. 1859)* **143,** 353–368.

57. Curson, J., Quinn, D. & Beadle, D. (1994) *Warblers of the Americas* (Houghton-Mifflin, Boston).

58. Daily, G. C., unpublished data.

59. De By, R. (2003) *Bird Bytes: Bird Taxonomy for Birders* (http://www.ornitaxa.com/index.html).

60. De Graaf, R. M. & Rappole, J. H.,. (1995) *Neotropical Migratory Birds: Natural History, Distribution, and Population Change* (Comstock Publishing Associates, Ithaca, NY).

61. De Schauensee, R. M. & Phelps, W. H. (1977) *A Guide to the Birds of Venezuela* (Princeton Univ. Press, Princeton).

62. del Hoyo, J., Elliott, A. & Sargatal, J., eds. (1992) *Handbook of the Birds of the World: Ostrich to Ducks* (Lynx Edicions, Barcelona), Vol. 1.

63. del Hoyo, J., Elliott, A. & Sargatal, J., eds. (1994) *Handbook of the Birds of the World: New World Vultures to Guineafowl* (Lynx Edicions, Barcelona), Vol. 2.

64. del Hoyo, J., Elliott, A. & Sargatal, J., eds. (1996) *Handbook of the Birds of the World: Hoatzin to Auks* (Lynx Edicions, Barcelona), Vol. 3.

65. del Hoyo, J., Elliott, A. & Sargatal, J., eds. (1997) *Handbook of the Birds of the World: Sandgrouse to Cuckoos* (Lynx Edicions, Barcelona), Vol. 4.

66. del Hoyo, J., Elliott, A. & Sargatal, J., eds. (1999) *Handbook of the Birds of the World: Barn owls to Hummingbirds* (Lynx Edicions, Barcelona), Vol. 5.

67. del Hoyo, J., Elliott, A. & Sargatal, J., eds. (2001) *Handbook of the Birds of the World: Mousebirds to Hornbills* (Lynx Edicions, Barcelona), Vol. 6.

68. del Hoyo, J., Elliott, A. & Sargatal, J., eds. (2002) *Handbook of the Birds of the World: Jacamars to Woodpeckers* (Lynx Edicions, Barcelona), Vol. 7.

69. del Hoyo, J., Elliott, A. & Sargatal, J., eds. (2003) *Handbook of the Birds of the World: Broadbills to Tapaculos* (Lynx Edicions, Barcelona), Vol. 8.

70. del Hoyo, J., Elliott, A. & Sargatal, J., eds. (2004) *Handbook of the Birds of the World: Cotingas to Pipits and Wagtails* (Lynx Edicions, Barcelona), Vol. 9.

71. Dinesen, L., Lehmberg, T., Svendsen, J. O., Hansen, L. A. & Fjeldsa, J. (1994) *Ibis* (*Lond. 1859)* **136,** 3–11.

72. Doughty, C., Day, N. & Plant, A. (1999) *Birds of the Solomons, Vanuatu, & New Caledonia* (Christopher Helm, London).

73. Downer, A. & Sutton, R. (1990) *Birds of Jamaica: A Photographic Field Guide* (Cambridge Univ. Press, Cambridge).

74. Duckworth, J. W., Alström, P., Davidson, P., Evans, T. D., Poole, C. M., Setha, T. & Timmins, R. J. (2001) *Bull. B.O.C.* **121,** 152–182.

75. Dunn, J. & Garrett, K. (1997) *A Field Guide to Warblers of North America* (Houghton-Mifflin, Boston).

76. Dunning, J. B. *Avian Body Mass Database*, 2nd Ed. 2, in press.

77. Dunning, J. B. (1993) *CRC Handbook of Avian Body Masses* (CRC Press, Boca Raton, FL).

78. Eames, J. C. & Eames, C. (2001) *Bull. B.O.C.* **121,** 10–23.

79. Eames, J. C., Le Trong, T., Nguyen, C. & Eve, R. (1999) *Ibis (Lond. 1859)* **141,** 1–10.

80. Eames, J. C., Trai, L. T. & Cu, N. (1999) *Bull. B.O.C.* **119,** 4–15.

81. Edden, R. & Boles, W. (1986) *Birds of the Australian Rainforests* (Reed, Sydney).

82. Ehrlich, P. R., Dobkin, D. S. & Wheye, D. (1988) *The Birder's Handbook: A Field Guide to the Natural History of North American Birds* (Simon & Schuster, New York).

83. Ehrlich, P. R., Dobkin, D. S. & Wheye, D. (1994) *The Birdwatcher's Handbook: A Guide to the Natural History of the Birds of Britain and Europe* (Oxford Univ. Press, Oxford).

84. Feare, C. & Craig, A. (1999) *Starlings and Mynas* (Princeton Univ. Press, Princeton).

85. ffrench, R. (1991) *Guide to the Birds of Trinidad and Tobago* (Comstock, London).

86. Fjeldså, J. & Krabbe, N. (1990) *Birds of the High Andes* (Zoological Museum University of Copenhagen & Apollo Books, Copenhagen).

87. Forero, M. G. & Tella, J. L. (1997) *Ibis (Lond. 1859)* **139,** 407–409.

88. Frith, C. B., Beehler, B. M. & Cooper, W. T. (1998) *The Birds of Paradise: Paradisaeidae* (Oxford Univ. Press, Oxford).

89. Fry, H. C., Keith, S. & Urban, E. K. (1988) *Birds of Africa: Parrots to Woodpeckers* (Academic, London), Vol. 3.

90. Fry, H. C., Urban, E. K. & Keith, S. (2000) *Birds of Africa: Picathartes to Oxpeckers* (Academic, London), Vol. 6.

91. Fry, H. C. & Keith, S. (2004) *Birds of Africa: Sparrows to Buntings* (Academic, London), Vol. 7.

92. Gaban-Lima, R., Raposo, M. A. & Hofling, E. (2002) *Auk* **119,** 815–819.

93. Garrido, O. H. & Kirkconnell, A. (2000) *Field Guide to the Birds of Cuba* (Comstock, Ithaca, NY).

94. Garrido, O. H., Kirwan, G. M. & Capper, D. R. (2002) *Bird Conserv. Int.* **12,** 169–187.

95. Gauthier, J. & Aubry, Y. (1996) *The Breeding Birds of Québec* (Association Québécoise des Groupes d'Ornithologoques , Montreal).

96. Gentry, A. H., ed. (1990) *Four Neotropical Rainforests* (Yale Univ. Press, New Haven, CT).

97. Gonzaga, L. P. & Pacheco, J. F. (1995) *Bull. B.O.C.* **115,** 88–97.

98. Goodman, S. M., Hawkins, A. F. A. & Domergue, C. A. (1997) *Bull. B.O.C.* **117,** 5–10.

99. Goodman, S. M., Langrand, O. & Whitney, B. M. (1996) *Ibis (Lond. 1859)* **138,** 153–159.

100. Goodwin, D. (1982) *Estrildid Finches of the World* (Comstock, London).

101. Graves, G. R. (1993) *Auk* **110,** 1–8.

102. Grimmett, R., Inskipp, C. & Inskipp, T. (1998) *Birds of the Indian Subcontinent* (Christopher Helm: A & C Black, London).

103. Hagemeijer, W. J. M. & Blair, M. J., eds. (1997) *The EBCC Atlas of European Breeding Birds* (T & A D Poyser, London).

104. Harrap, S. & Quinn, D. (1995) *Chickadees, Tits, Nuthatches, & Treecreepers* (Princeton Univ. Press, Princeton).

105. Harris, T. & Franklin, K. (2000) *Shrikes & Bush-shrikes* (Christopher Helm, London).

106. Harrison, J. A., Allan, D. G., Underhill, L. G., Herremans, M., Tree, A. J., Parker, V. & Brown, C. J., eds. (1997) *The Atlas of Southern African Birds: Nonpasserines* (BirdLife South Africa, Johannesburg), Vol. 1.

107. Harrison, J. A., Allan, D. G., Underhill, L. G., Herremans, M., Tree, A. J., Parker, V. & Brown, C. J., eds. (1997) *The Atlas of Southern African Birds: Passerines* (BirdLife South Africa, Johannesburg), Vol. 2.

108. Hazevoet, C. J. (1992) *Bird Conserv. Int.* **2,** 15–23.

109. Hazevoet, C. J. (1996) *Bird Conserv. Int.* **6,** 181–196.

110. Heather, B. D. & Robertson, H. A. (1997) *The Field Guide to the Birds of New* Zealand (Viking, Auckland).

111. Higgins, P. J. & Peter, J. M., eds. (2002) *Handbook of Australian, New Zealand, & Antarctic Birds: Pardalotes to Shrike thrushes* (Oxford Univ. Press, Melbourne), Vol. 6.

112. Higgins, P. J. & Peter, J. M. & Steele, W. K., eds. (2001) *Handbook of Australian, New Zealand, & Antarctic Birds: Tyrant-flycatchers to Chats* (Oxford Univ. Press, Melbourne), Vol. 5.

113. Hilty, S. L. & Brown, W. L. (1986) *A Guide to the Birds of Colombia* (Princeton Univ. Press, Princeton).

114. Hilty, S. L. & De Schauensee, R. M. (2003) *Birds of Venezuela* (Princeton Univ. Press, Princeton).

115. Howell, S. N. G. & Robbins, M. B. (1995) *Wilson Bull.* **107**, 7–25.

116. Howell, S. N. G. & Webb, S. (1995) *A Guide to the Birds of Mexico and Northern Central America* (Oxford Univ. Press, Oxford).

117. Imber, M. J. & Tennyson, A. J. D. (2001) *Emu* **101,** 123–127.

118. Isler, M. L. Alonso, J. A., Isler, P. R. Valqui, T., Begazo, A. & Whitney, B. M. (2002) *Auk* **119,** 362–378.

119. Isler, M. L., Alonso, J. A., Isler, P. R. & Whitney, B. M. (2001) *Wilson Bull.* **113,** 164–176.

120. Isler, M. L. & Isler, P. R. (1999) *The Tanagers: Natural History, Distribution, and Identification* (Smithsonian Institution Press, Washington, D.C.).

121. Isler, M. L., Isler, P. R. & Whitney, B. M. (1998) *Auk* **115,** 577–590.

122. Isler, M. L., Isler, P. R. & Whitney, B. M. (1999) *Auk* **116,** 83–96.

123. Isler, M. L., Isler, P. R., Whitney, B. M. & Walker, B. (2001) *Condor* **103**, 278–286.

124. International Union for Conservation of Nature and Natural Resources (2002) *2002 IUCN Red List of Threatened Species*; www.redlist.org.

125. International Union for Conservation of Nature and Natural Resources (2003) *2003 IUCN Red List of Threatened Species*; www.redlist.org.

126. Jaramillo, A. & Burke, P. (1999) *New World Blackbirds: The Icterids* (Princeton Univ. Press, Princeton).

127. Johnson, N. K. (2002) *Ibis (Lond. 1859)* **144,** 69–84.

128. Johnson, N. K. & Jones, R. E. (2001) *Auk* **118,** 334–341.

129. Jonsson, L. (1992) *Birds of Europe with North Africa and the Middle East* (Princeton Univ. Press, Princeton).

130. Karr, J. R., Robinson, S. O., Blakers, M. & Bierregaard, R. O. (1990) in *Four Neotropical Rainforests*, ed. Gentry, A. H. (Yale Univ. Press, New Haven, CT).

131. Keith, S., Urban, E. K. & Fry, H. C. (1992) *Birds of Africa: Broadbills to Chats* (Academic, London), Vol. 4.

132. Kennedy, R. S. (2000) *A Guide to the Birds of the Philippines* (Oxford Univ. Press, New York).

133. Kennedy, R. S., Gonzales, P. C. & Miranda, H. C., Jr. (1997) *Auk* **114,** 1–10.

134. King, B. F. (2002) *Bull. B.O.C.* **122,** 74–80.

135. Knox, A. G., Collinson, M., Helbig, A. J., Parkin D. T. & Sangster, G. (2002) *Ibis (Lond. 1859)* **144,** 707–710.

136. Krabbe, N., Agro, D. J., Rice, N. H., Jacome, M., Navarrete, L., & Sornoza, M. F. (1999) *Auk* **116,** 882–890.

137. Krabbe, N., Isler, M. L., Isler, P. R., Whitney, B. M., Alvarez, J., Greenfield, P. J. (1999) *Wilson Bull.* **111,** 157–165.

138. Lambert, F. R. (1998) *Bull. B.O.C.* **118,** 67–82.

139. Lambert, F. R. & Rasmussen, P. C. (1998) *Bull. B.O.C.* **118,** 204–217.

140. Lambert, F. R. & Woodcock, M. (1996) *Pittas, Broadbills, and Asities* (Pica, Mountfield).

141. Langrand, O. (1990) *Guide to the Birds of Madagascar* (Yale Univ. Press, New Haven, CT).

142. Lee, R. J. & Riley, J. (2001) *Wilson Bull.* **113,** 17–22.

143. Lee, W. S., Koo, T.H. & Park, J.Y. (2000) *A Field Guide to the Birds of Korea* (L.C. Evergreen Foundation, Seoul).

144. Lefranc, N. & Worfolk, T. (1997) *Shrikes: A Guide to the Shrikes of the World* (Yale Univ. Press, New Haven).

145. Lencioni-Neto, F. (1994) *Alauda* **62,** 241–245.

146. Lever, C. & Gillmor, R. (1987) *Naturalized Birds of the World* (Longman, Essex, U.K.).

147. Liversidge, R. (1996) *Bull. B.O.C.* **116,** 211–215.

148. Liversidge, R. & Voelker, G. (2002) *Bull. B.O.C.* **122,** 93–109.

149. MacKinnon, J. R. & Phillipps, K. (2000) *A Field Guide to the Birds of China* (Oxford Univ. Press, Oxford).

150. MacKinnon, J. R. & Phillipps, K. (1993) *A Field Guide to the Birds of Borneo, Sumatra, Java, and Bali* (Oxford Univ. Press, Oxford).

151. Mackworth-Praed, C. W. & Grant, C. H. B. (1957) *Birds of Eastern and North Eastern Africa*, (Longmans Green, London).

152. Madge, S. & Burn, H. (1994) *Crows and Jays: A Guide to the Crows, Jays, and Magpies of the World* (Houghton-Mifflin, Boston).

153. Maijer, S. & Fjeldsa, J. (1997) *Ibis (Lond. 1859)* **139,** 606–616.

154. Marchant, S., Higgins, P. J. & Ambrose, S. J. (1990) *Handbook of Australian, New Zealand, & Antarctic Birds* (Oxford Univ. Press, Melbourne).

155. Martens, J. & Eck, S. (1995) *Towards an Ornithology of the Himalayas: Systematics, Ecology, and Vocalizations of Nepal Birds* (Zoologisches Forschungsinstitut und Museum Alexander Koenig, Bonn).

156. Mayr, E. & Diamond, J. M. (2001) *The Birds of Northern Melanesia: Speciation, Ecology, & Biogeography* (Oxford Univ. Press, New York).

157. Mayr, E. & Jaques, F. L. (1978) *Birds of the Southwest Pacific: A Field Guide to the Birds of the Area between Samoa, New Caledonia, and Micronesia* (C. E. Tuttle, Rutland, VT).

158. Monroe, B. L. & Sibley, C. G. (1993) A World Checklist of Birds (Yale Univ. Press, New Haven, CT).

159. Morcombe, M. K. (2000) *Field Guide to Australian Birds* (Steve Parish Publishing, Archerfield, Australia).

160. Olsen, J., Wink, M., Sauer-Gurth, H. & Trost, S. (2002) *Emu* **102,** 223–231.

161. Olson, S. L. & Jouventin, P. (1996) *Condor* **98,** 1–9.

162. Olsson, U., Alstroem, P. & Colston, P. R. (1993) *Ibis (Lond. 1859)* **135,** 3–7.

163. O'Neill, J. P., Lane, D. F., Kratter, A. W., Capparella, A. P. & Joo, C. F. (2000) *Auk* **117,** 569–577.

164. Pacheco, J. F., Whitney, B. M. & Gonzaga, L. P. (1996) *Wilson Bull.* **108,** 397–433.

165. Parker, T. A. & Remsen, J. V. (1997) *Studies in Neotropical Ornithology Honoring Ted Parker* (AOU, Washington, D.C.).

166. Parker, T. A., Schulenberg, T. S., Graves, G. R. & Braun, M. J. (1985) in *Neotropical Ornithology*, eds. Buckley, P. A., Foster, M. S., Morton, E. S., Ridgely, R. S. & Buckley, F. G. (Am. Ornithologists' Union, Washington, D.C.).

167. Parry, S. J., Clark, W. S. & Prakash, V. (2002) *Ibis (Lond. 1859)* **144,** 665–675.

168. Payne, R. B. (1998) *Ibis (Lond. 1859)* **140,** 368–381.

169. Peters, D. S. (1996) *Senckenb. Biol.* **76,** 7–14.

170. Pratt, H. D., Bruner, P. L. & Berrett, G. D. (1987) *The Birds of Hawaii and the Tropical Pacific* (Princeton Univ. Press, Princeton).

171. Raffaele, H. A. (1998) *A Guide to the Birds of the West Indies* (Princeton Univ. Press, Princeton).

172. Rasmussen, P. C. (1991) *Condor* **93,** 825–839.

173. Rasmussen, P. C. (1998) *Bull. B.O.C.* **118,** 141–153.

174. Rasmussen, P. C. (1999) *Wilson Bull.* **111,** 457–464.

175. Rasmussen, P. C., Round, P. D., Dickinson, E. C. & Rozendaal, F. G. (2000) *Auk* **117,** 279–289.

176. Rasmussen, P. C., Schulenberg, T. S., Hawkins, F. & Voninavoko, R. (2000) *Bull. B.O.C.* **120,** 75–102.

177. Remold, H. G. & Ramos Neto, M. B. (1995) *Bull. B.O.C.* **115,** 239–240.

178. Restall, R. L. (1995) *Bull. B.O.C.* **115,** 140–157.

179. Restall, R. L. (1996) *Bull. B.O.C.* **116,** 137–142.

180. Restall, R. L. (1997) *Munias and Mannikins* (Yale Univ. Press, New Haven, CT).

181. Ridgely, R. S. & Greenfield, P. J. (2001) *The Birds of Ecuador* (Comstock, Ithaca, N.Y.).

182. Ridgely, R. S. & Greenfield, P. J. (2001) *The Birds of Ecuador: Field Guide* (Comstock, Ithaca, N.Y.).

183. Ridgely, R. S. & Gwynne, J. A. (1989) *A Guide to the Birds of Panama* (Princeton Univ. Press, Princeton).

184. Ridgely, R. S. & Tudor, G. (1994) *The Birds of South America: The Suboscine Passerines* (University of Texas Press, Austin).

185. Ridgely, R. S., Tudor, G. & Brown, W. L. (1999) *The Birds of South America: The Oscine Passerines* (University of Texas Press, Austin).

186. Robbins, M. B. & Howell, S. N. G. (1995) *Wilson Bull.* **107,** 1–7.

187. Robbins, M. B., G. H. Rosenberg & F. S. Molina,. (1994) *Auk* **111,** 1–6.

188. Robbins, M. B. & F. G. Stiles,. (1999) *Auk* **116,** 305–315.

189. Robson, C. A. (2000) *Guide to the Birds of Southeast Asia* (Princeton Univ. Press, Princeton).

190. Robson, C. (2002) *Birds of Thailand* (Princeton Univ. Press, Princeton).

191. Rodríguez, J. V. & Instituto Nacional de los Recursos Naturales Renovables y del Ambiente (1982) *Aves del Parque Nacional Natural Los Katíos, Choco, Colombia* (Inderena, Bogotá).

192. Rohwer, S., Filardi, C. E., Bostwick, K. S. & Townsend Peterson, A. (2000) *Auk* **117,** 308–320.

193. Roselaar, C. S. (1992) *Bull. B.O.C.* **112,** 225–231.

194. Rowley, I., Russell, E. M. & Marsack, P. (1997) *Fairywrens and Grasswrens: Maluridae* (Oxford Univ. Press, Oxford).

195. Saetre, G. P., Borge, T. & Moum, T. (2001) *Ibis (Lond. 1859)* **143,** 494–497.

196. Safford, R. J., Ash, J. S. & Duckworth, J. W. (1997) *Ibis (Lond. 1859)* **139,** 410–411.

197. Safford, R. J., Ash, J. S., Duckworth, J. W., Telfer, M. G. & Zewdie, C. (1995) *Ibis (Lond. 1859)* **137,** 301–307.

198. Salaman, P. G. W. & Stiles, F. G. (1996) *Ibis (Lond. 1859)* **138,** 610–619.

199. Sangster, G., Knox, A. G., Helbig, A. J. & Parkin, D. T. (2002) *Ibis (Lond. 1859)* **144,** 153–159.

200. Schodde, R., Tidemann, S. C. & Bell, H. L. (1986) *Reader's Digest Complete Book of Australian Birds* (Reader's Digest Services, Sydney).

201. Serle, W. & Morel, G. J. (1988) *The Collins Field Guide to the Birds of West Africa* (S. Greene Press, Lexington, MA).

202. Shirihai, H. (1996) *The Birds of Israel* (Academic, San Diego).

203. Shirihai, H., Gargallo, G., Helbig, A. J., Harris, A., Kirwan, G. M., Svensson, L. (2001) *Sylvia Warblers* (Princeton Univ. Press, Princeton).

204. Shirihai, H., Sinclair, I. & Colston, P. R. (1995) *Bull. B.O.C.* **115,** 75–87.

205. Sibley, D. (2000) *The Sibley Guide to Birds* (Alfred A. Knopf, New York).

206. Sibley, D., Elphick, C. & Dunning, J. B. (2001) *The Sibley Guide to Bird Life and Behavior* (Alfred A. Knopf, New York).

207. Sick, H. (1993) *Birds in Brazil* (Princeton Univ. Press, Princeton).

208. Silva, J. M. C. D., Novaes, F. C. & Oren, D. C. (1995) *Bull. B.O.C.* **115,** 200–206.

209. Sim, I. M. W. & Zefania, S. (2002) *Bull. B.O.C.* **122,** 194–196.

210. Sinclair, I., Hockey, P. A. R. & Tarboton, W. R. (2002) *Sasol Birds of Southern Africa* (Struik, Cape Town, South Africa).

211. Sinclair, J. C. & Langrand, O. (1998) *Birds of the Indian Ocean Islands* (Struik, Cape Town, SouthAfrica).

212. Skerrett, A. & Bullock, I. (2001) *Birds of the Seychelles* (Princeton Univ. Press, Princeton).

213. National Geographic Society (2002) *Field Guide to the Birds of North America* (Nat. Geograph. Soc., Washington, D.C.).

214. Stattersfield, A. J., Crosby, M. J., Long, A. J. & Wege, D. C. (1998) *Endemic Bird Areas of the World* (BirdLife International, Cambridge, U.K.).

215. Stevenson, T. & Fanshawe, J. (2002) *Field Guide to the Birds of East Africa: Kenya, Tanzania, Uganda, Rwanda, Burundi* (T & A D Poyser, London).

216. Stiles, F. G. (1996) *Wilson Bull.* **108,** 1–27.

217. Stiles, F. G. & Skutch, A. F. (1989) *A Guide to the Birds of Costa Rica* (Comstock, Ithaca, N.Y.).

218. Stotz, D. F., Parker, T. A. & Fitzpatrick, J. W. (1996) *Neotropical Birds: Ecology and Conservation* (University of Chicago Press, Chicago).

219. Stotz, D. F., Parker, T. A. & Fitzpatrick, J. W. (1996) *Database of Neotropical Birds* (University of Chicago Press, Chicago).

220. Strange, M. (2000) *A Photographic Guide to the Birds of Southeast Asia* (Periplus, Hong Kong).

221. Strange, M. (2001) *A Photographic Guide to the Birds of Indonesia* (Periplus, Hong Kong).

222. Stuart, C. & Stuart, T. (1999) *Birds of Africa: From Seabirds to Seedeaters* (Southern Book Publishers, Rivonia South Africa).

223. Summers-Smith, D. J. (1988) *The Sparrows: A Study of the Genus Passer* (T & AD Poyser, Calton. U.K.).

224. Şekercioğlu, Ç. H., unpublished data.

225. Turner, A. K. & Rose, C. (1989) *A Handbook to the Swallows and Martins of the World* (Christopher Helm, London).

226. American Ornithologists' Union & Academy of Natural Sciences of Philadelphia, eds. (1992–2002) *The Birds of North America* (Amer. Ornithol. Union, Washington, D.C.), Vols. 1–17.

227. Urban, E. K., Fry, H. C. & Keith, S. (1986) *Birds of Africa: Gamebirds to Pigeons* (Academic, London), Vol. 2.

228. Urban, E. K., Fry, H. C. & Keith, S. (1997) *Birds of Africa: Thrushes to Puffback Flycatchers* (Academic, London), Vol. 5.

229. Urquhart, E. & Bowley, A. (2002) *Stonechats: A Guide to the Genus Saxicola* (Christopher Helm, London).

230. Valqui, T. & Fjeldsa, J. (2002) *Ibis (Lond. 1859)* **144,** 347.

231. Verde, P. (2002) *Ornitologia e dintorni*, (http://digilander.libero.it/avifauna/classificazione/sibley's_index.htm).

232. Vigil, C. (1973) *Aves Argentinas y Sudamericanas* (Editorial Atlantida, Buenos Aires).

233. Watling, D., Rinke, D. R., Talbot-Kelly, C. & Rolls, I. (2001) *A Guide to the Birds of Fiji and Western Polynesia* (Environmental Consultants, Suva, Fiji).

234. Wells, D., Round, P. D. & Treesucon, U. (1999) *The Birds of the Thai-Malay Peninsula* (Academic, San Diego).

235. Wells, M. G. (1998) *World Bird Species Checklist* (Worldlist, Bushey, U.K.).

236. Whitney, B. M. (1994) *Wilson Bull.* **106,** 585–614.

237. Whitney, B. M. & Alonso, J. A. (1998) *Auk* **115,** 559–576.

238. Whitney, B. M., Pacheco, J. F., Buzzetti, D. R. C. & Parrini, R. (2000) *Auk* **117,** 869–891.

239. Whitney, B. M., Pacheo, J. F., Fonseca, P. S. M. D. & Barth, R. H. (1996) *Wilson Bull.* **108,** 434–448.

240. Williams J. G. (1988) *The Collins Field Guide to the Birds of East Africa* (S. Greene Press, Lexington, MA).

241. Williams, J. G. & Fennessy, R. (1966) *Birds of Africa* (Collins, London).

242. Willis, E. O. & Oniki, Y. (1992) *Bull. B.O.C.* **112,** 158–165.

243. Young, J. R., Braun, C. E., Oyler-McCance, S. J., Hupp, J. W. & Quinn, T. W. (2000) *Wilson Bull.* **112,** 445–453.

244. Zimmer, K. J. (2002) *Wilson Bull.* **114,** 20–37.

245. Zimmer, K. J. & Whittaker, A. (2000) *Wilson Bull.* **112,** 51–66.

246. Zimmer, K. J., Whittaker, A. & Oren, D. C. (2001) *Auk* **118,** 56–78.

247. Zimmerman, D. A., Turner, D. A. & Pearson, D. J. (1996) *Birds of Kenya and Northern Tanzania* (Princeton Univ. Press, Princeton).

248. Zook, J., unpublished data.
